# Triazine Chalcogenones from Thiocyanate or Selenocyanate Addition to Tetrazine Ligands in Ruthenium Arene Complexes

**DOI:** 10.1021/acs.inorgchem.3c00459

**Published:** 2023-05-11

**Authors:** Lorenzo Bonaldi, Marco Bortoluzzi, Stefano Zacchini, Guido Pampaloni, Fabio Marchetti, Lorenzo Biancalana

**Affiliations:** † Department of Chemistry and Industrial Chemistry, University of Pisa, Via G. Moruzzi 13, I-56124 Pisa, Italy; ‡ Department of Molecular Sciences and Nanosystems, Ca’ Foscari University of Venice, Via Torino 155, I-30175 Mestre, Venezia, Italy; § Department of Industrial Chemistry “Toso Montanari”, University of Bologna, Viale del Risorgimento 4, I-40136 Bologna, Italy

## Abstract

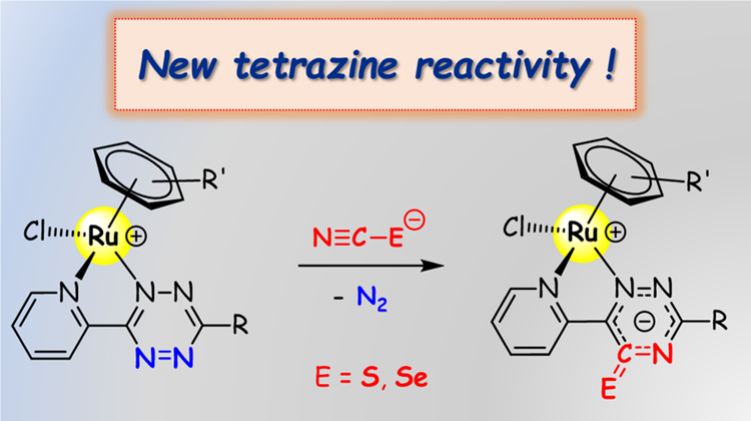

The chemistry of 1,2,4,5-tetrazines has attracted considerable interest both from a synthetic and applicative standpoint. Recently, regioselective reactions with alkynes and alkenes have been reported to be favored once the tetrazine ring is coordinated to Re(I), Ru(II), and Ir(III) centers. Aiming to further explore the effects of metal coordination, herein, we unveil the unexplored reactivity of tetrazines with chalcogenocyanate anions. Thus, ruthenium(II) tetrazine complexes, [RuCl{κ^2^*N*-3-(2-pyridyl)-6-*R*-1,2,4,5-tetrazine}(η^6^-arene)]^+^ (arene = *p*-cymene, R = H, [**1a**]^+^, R = Me, [**1b**]^+^, R = 2-pyridyl, [**1c**]^+^; arene = C_6_Me_6_, R = H, [**1d**]^+^, R = Me, [**1e**]^+^; PF_6_^–^ salts), reacted quantitatively and in mild conditions with M(ECN) salts (M = Na, K, Bu_4_N; E = O, S, Se). The addition of thiocyanate or selenocyanate to the tetrazine ligand is regioselective and afforded, *via* N_2_ release, 1,2,4-triazine-5-chalcogenone heterocycles, the one with selenium being unprecedented. The novel ruthenium complexes [RuCl{κ^2^*N*-(2-pyridyl)}{triazine chalcogenone}(η^6^-arene)] **2a**–**e** (sulfur), **3b**, **3d**, and **3e** (selenium) were characterized by analytical (CHNS analyses, conductivity), spectroscopic (IR, multinuclear and two-dimensional (2D) NMR), and spectrometric (electrospray ionization mass spectrometry (ESI-MS)) techniques. According to density functional theory (DFT) calculations, the nucleophilic attack of SCN^–^ on the tetrazine ring is kinetically driven. Compound **2b** is selectively and reversibly mono-protonated on the triazine ring by HCl or other strong acids, affording a single tautomer. When reactions of chalcogenocyanates were performed on the 2,2′-bipyridine (bpy) complex [RuCl(bpy)(η^6^-*p*-cymene)]^+^, the chloride substitution products [Ru(ECN)(bpy)(η^6^-*p*-cymene)]^+^ (E = O, [**4**]^+^; E = S, [**5**]^+^; E = Se, [**6**]^+^) were obtained in 82–90% yields (PF_6_^–^ salts). Combined spectroscopic data (IR, ^1^H/^13^C/^77^Se NMR) was revealed to be a useful tool to study the linkage isomerism of the chalcogenocyanate ligand in [**4**–**6**]^+^.

## Abstract 2

Coordination of 1,2,4,5-tetrazines to ruthenium(II) η^6^-arene scaffolds enables their unprecedented reactivity with chalcogenocyanate ions, affording triazine-thione and triazine-selone heterocycles.

## 1 Introduction

1,2,4,5-Tetrazines^1^ attracted considerable interest in the light of their physicochemical properties and reactivity, allowing their application in diverse fields such as material chemistry, supramolecular chemistry, and biochemistry.^2^ Most notably, they are known to react with alkenes and alkynes by an inverse electron demand [4 + 2] addition/elimination mechanism, allowing incorporation of the C_2_ unit within a (dihydro)pyridazine-type ring, the driving force of this process being N_2_ release.^3^ The Diels–Alder reactivity of 1,2,4,5-tetrazines was also extended to CN heterodienophiles like nitriles/cyanamides,^4^ amidines/(thio)imidates,^5,6^ hydrazones,^6,7^ and (thi)oxazolines.^8^ Instead, isocyanides^9^ react *via* a [4 + 1] cycloaddition with 1,2,4,5-tetrazines, affording an iminopyrazole. Investigations on the reactivity of tetrazines gained momentum in recent years, regarding the use of the tetrazine/alkene and tetrazine/isocyanide ligation in aqueous solution as a bioorthogonal tool.^10,11^ For instance, combinations of tetrazines tethered to fluorescent probes and alkene-derivatized biomolecules—or *vice versa*—allowed *in vivo* imaging studies of tumor sites.^12^

**Scheme 1:**
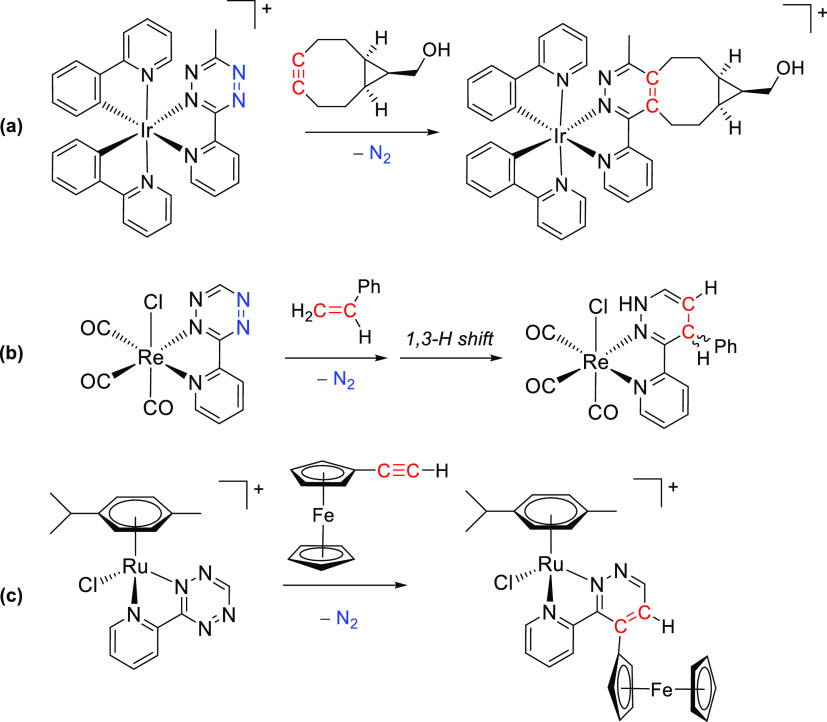
Reactions of Iridium(III) (a), Rhenium(I) (b), and Ruthenium(II) (c) Pyridyl Tetrazine Complexes with Alkenes or Alkynes Cationic complexes as PF_6_^–^ salts.

Metal complexes of tetrazines have been known for a long time.^13^ In this respect, the tetrazine nitrogen atoms are rather poor Lewis bases, requiring assistance from another coordinating unit to obtain a good chelating ligand, most commonly a 2-pyridyl substituent.^14,15^ Nevertheless, how tetrazine metalation affects its Diels–Alder reactivity has remained unexplored until very recently, when it was demonstrated that coordination of pyridyl tetrazines to {Ir^III^(N∧C)_2_} (N∧C = cyclometalated 2-phenylpyridine), {*fac*-Re^I^Cl(CO)_3_} and {Ru^II^Cl(*p*-cymene)}^+^ fragments greatly accelerates the reaction with alkynes and alkenes (Scheme 1).^16^ This result is remarkable on considering that alkynes normally require harsh conditions to react with tetrazines (*e.g.*, refluxing toluene or dimethylformamide (DMF) for 24–48 h),^17^ while the reaction of the ruthenium(II) *p*-cymene 3-pyridyl tetrazine complex and ethynylferrocene (Scheme 1c) proceeded in CH_2_Cl_2_ at room temperature.^16c^ Furthermore, reactions of Re(I) and Ru(II) tetrazine complexes with unsymmetrical alkenes/alkynes occurred in a regioselective fashion, at variance to what is commonly observed with the free tetrazines.^3b,10a^

**Figure 1:**
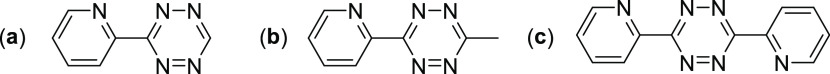
Pyridyl tetrazines employed in this work: 3-(2-pyridyl)-1,2,4,5-tetrazine (a), 3-(2-pyridyl)-6-methyl-1,2,4,5-tetrazine (b), and 3,6-di(2-pyridyl)-1,2,4,5-tetrazine (c).

These results fueled our interest in the study of the reactivity of metalated tetrazines with dienophiles, aiming to discover new pathways to heteroaromatic rings. In the light of the absence of literature information on the reactions of 1,2,4,5-tetrazines with potential heterodonor dienophiles such as chalcogenocyanate ions, we decided to extend the reactivity of the ruthenium(II) η^6^-arene tetrazine system, and three pyridyl tetrazine ligands were selected for this work (Figure 1).

## 2 Results and Discussion

### 2.1 Tetrazine Coordination and Reactivity with Thiocyanate

**Scheme 2:**
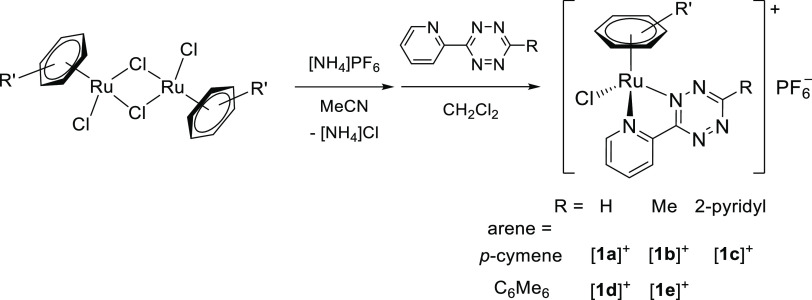
Preparation of Ruthenium(II) η^6^-Arene Pyridyl Tetrazine Complexes [**1a**–**e**]^+ ^ All reactions were carried out at room temperature in stoichiometric conditions.

Ruthenium(II) arene complexes [**1a**–**e**]PF_6_ were prepared by a two-step procedure involving the cleavage of [RuCl_2_(arene)]_2_ dimers (arene = *p*-cymene, C_6_Me_6_) with [NH_4_]PF_6_ in acetonitrile, followed by addition of the pyridyl tetrazine in CH_2_Cl_2_ (Scheme 2). The products were isolated as dark red-brown ([**1a**]PF_6_ and [**1b**]PF_6_) or red-purple ([**1d**]PF_6_ and [**1e**]PF_6_) solids in 92–96% yield. In this respect, the preparation and isolation of [**1c**]PF_6_, featuring a (2,6-dipyridyl)-1,2,4,5-tetrazine ligand, was met with unexpected difficulties (see Section 4 for details). Compounds [**1b**–**e**]PF_6_ are unprecedented, while [**1a**]PF_6_ was previously reported,^16c^ and they were characterized by CNHS analyses, solid-state IR, and multinuclear (^1^H, ^13^C, ^19^F, ^31^P) NMR in acetone-*d*_6_ or CDCl_3_. IR and NMR spectra are displayed in Figures S4–S16. Negligible changes were observed in the ^1^H NMR spectrum of CD_3_CN solutions of [**1a**]^+^ and [**1b**]^+^ at room temperature over 24 h. Instead, [**1a**]^+^ afforded a dark blue-violet solution after 14 h in acetone-*d*_6_, with the appearance of an additional ^1^H NMR sets of signals, while [**1b**]^+^ was substantially inert in acetone-*d*_6_ up to 48 h, judging by the ^1^H NMR spectra of the initial and final solutions. Moreover, partial decomposition occurred with solid [**1a**]PF_6_ kept under N_2_ for some months at room temperature, suggesting low temperature (−20 °C) storage under N_2_ as the best option.

**Scheme 3:**
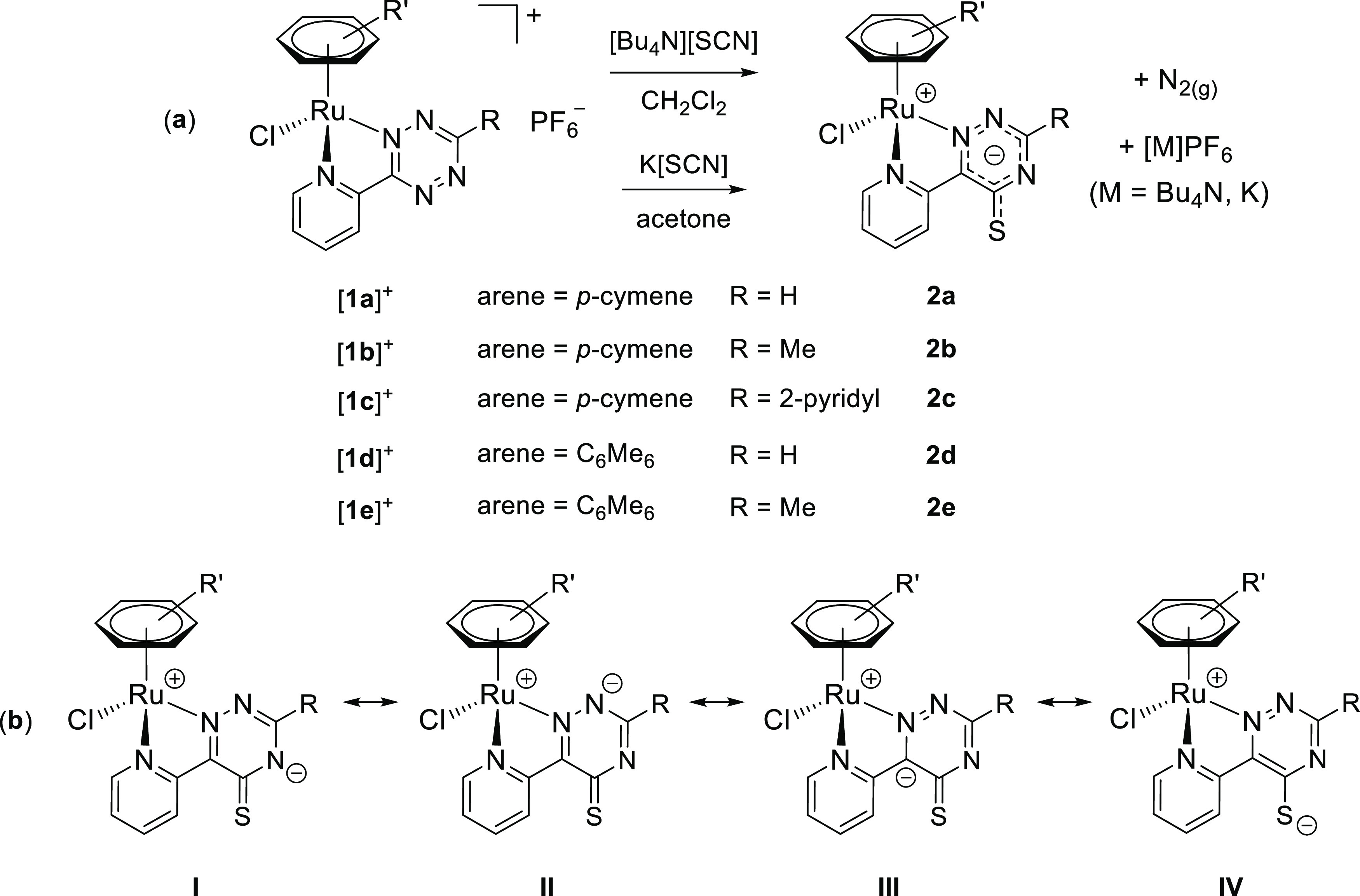
(a) Synthesis of **2a**–**e** by Reaction of the Tetrazine Complexes [**1a**–**e**]^+^ with Potassium or Tetrabutylammonium Thiocyanate; (b) Resonance Structures **I**–**IV** of the (2-Pyridyl)-5-thioxo-1,2,4-triazinide (Pyridyl–Triazine–Thione) Ligand within **2a**–**e**, above Represented by the Overall Structure with Delocalized Negative Charge Reactions were carried out at room temperature in stoichiometric conditions.

Tetrazine complexes [**1a**–**e**]PF_6_ reacted rapidly and quantitatively with potassium or tetrabutylammonium thiocyanate in CH_2_Cl_2_ or acetone at room temperature, affording neutral (formally zwitterionic) derivatives **2a**–**e** incorporating the {SCN} unit within a (2-pyridyl)-5-thioxo-1,2,4-triazinide ligand (Scheme 3a). The newly generated heterocycle is represented with a delocalized anionic charge, to better account for the electronic structure, and will be referred to as triazine-thione for simplicity, with reference to the resonance structure with a CS double bond (Scheme 3b).

The formation of **2a**, **2b**, **2d**, and **2e** proceeded with complete selectivity and gas production (N_2_). Compounds **2a** and **2b** were isolated as red-orange powders in 92% yield (50 mg scale) while **2d** and **2e** were prepared in the NMR tubes containing [**1d**]PF_6_ and [**1e**]PF_6_. In this respect, the use of KSCN is advantageous being the KPF_6_ co-product ^1^H and ^13^C NMR silent and easily removed by filtration from CH_2_Cl_2_. Differently, reactions of [**1c**]PF_6_ with M[SCN] (M = K^+^, Bu_4_N^+^) formed minor byproducts and **2c** was purified with silica chromatography.

To date, not more than 130 examples of 1,2,4-triazine-5-thione/thiolate derivatives have been reported in the literature.^18^ The most common synthetic protocol involves the preliminary multistep preparation of the corresponding carbonyl derivative followed by oxygen/sulfur exchange using P_2_S_5_.^19^ Only in one case this kind of heterocycle was assembled from a (symmetrical) 1,2,4,5-tetrazine and an external source of the {SCN} group, *i.e*., trimethylsilyl isothiocyanate, under forced conditions (20:1 molar excess of Me_3_SiNCS, DMF room temperature or reflux).^20^ Interestingly, addition of Me_3_SiNCS to the tetrazine ring was proposed as the first step of the reaction and this reagent is prone to undergo heterolytic cleavage of the Si–N bond^21^ thus mimicking the attack of a thiocyanate anion.

**Scheme 4:**
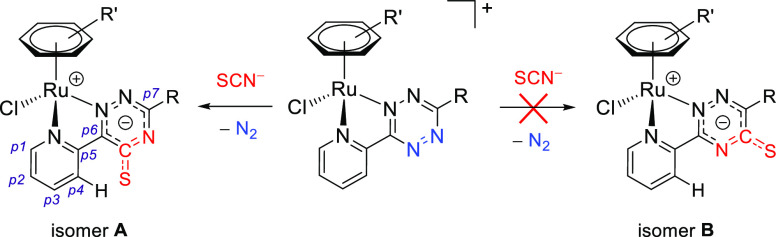
General Structure of the Triazine-Thione Isomers of **2a**–**e** Obtained by a Different Regiochemistry of the Tetrazine/Thiocyanate Reaction The C/H atom numbering (p1–p7) for the experimentally observed isomer **A** and the pyridyl hydrogen adjacent to the triazine ring are indicated (see text).

In principle, the addition of the thiocyanate ion to our unsymmetrical 1,2,4,5-tetrazine core may result in two different isomers (Scheme 4), displaying the exocyclic CS group adjacent to the pyridyl ring (**isomer A**) or to the R substituent (**isomer B**). Nevertheless, only one species was detected in solution for **2a**–**e**, indicating that the reaction proceeded with complete regioselectivity in all cases. Despite X-ray quality crystals were not obtained, multinuclear NMR studies (see below) indicate the **isomer A** structure for **2a**–**e**.

**Figure 2:**
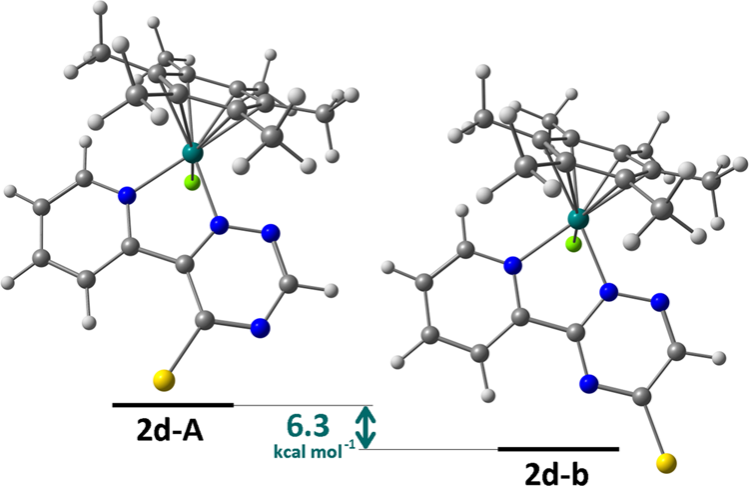
DFT-optimized structures of **2d-A** and **2d-B** (C-PCM/TPSS0/def2-TZVP calculations, acetone as continuous medium). Color map: Ru, dark green; Cl, green; S, yellow; N, blue; C, gray; H, white. Selected computed bond lengths for **2d-A** (Å): Ru–N(pyridine) 2.062, Ru–N(triazine) 2.046, Ru–Cl 2.416, Ru–C(average) 2.228. Selected computed bond lengths for **2d-B** (Å): Ru–N(pyridine) 2.098, Ru–N(triazine) 2.068, Ru–Cl 2.422, Ru–C(average) 2.218.

Density functional theory (DFT) calculations were performed on compound **2d** as a representative example. The isomeric forms **2d-A** and **2d-B** are depicted in Figure 2 with their relative Gibbs free energy. Interestingly, **2d-A** is thermodynamically less stable than **2d-B** by about 6.3 kcal·mol^–1^ (C-PCPM/TPSS0-def2-TZVP calculations, 4.7 kcal·mol^–1^ with PBEh-3c calculations); thus, its exclusive formation should be ascribed to kinetic factors.

**Figure 3:**
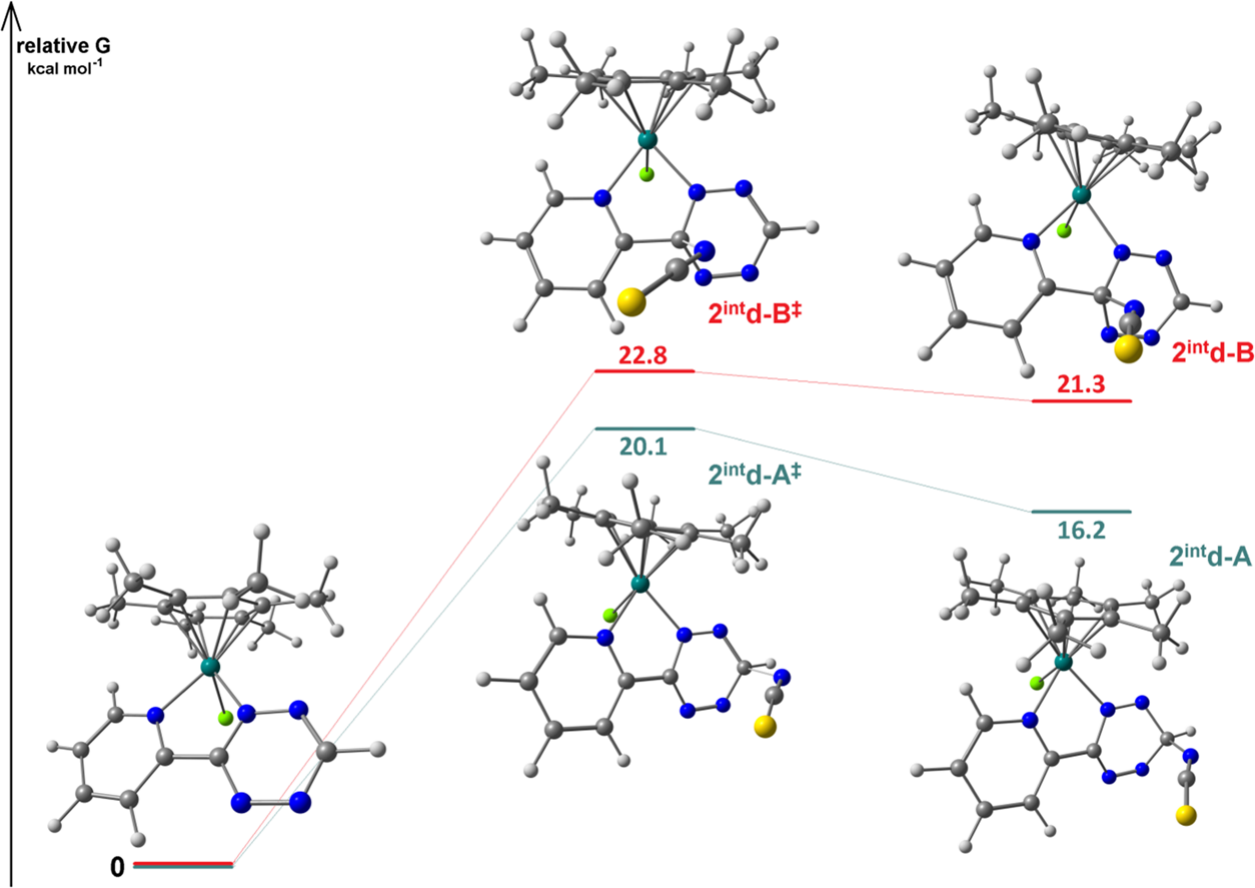
DFT-optimized structures of [**1d**]^+^, **2**^**int**^**d-A**, and **2**^**int**^**d-B** and of the related transition states (C-PCM/TPSS0/def2-TZVP, acetone as continuous medium) with relative Gibbs energy values. Color map: Ru, dark green; Cl, green; S, yellow; N, blue; C, gray; H, white. Selected computed bond lengths for [**1d**]^+^ (Å): Ru–N(pyridine) 2.092, Ru–N(triazine) 2.019, Ru–Cl 2.401, Ru–C(average) 2.236. Selected computed bond lengths for **2**^**int**^**d-A** (Å): Ru–N(pyridine) 2.102, Ru–N(triazine) 2.064, Ru–Cl 2.418, Ru–C(average) 2.224, C(triazine)–N(NCS) 1.469. Selected computed bond lengths for **2**^**int**^**d-B** (Å): Ru–N(pyridine) 2.103, Ru–N(triazine) 2.055, Ru–Cl 2.426, Ru–C(average) 2.217, C(triazine)–N(NCS) 1.490. Selected computed bond lengths for **2**^**int**^**d-A**^**‡**^ (Å): Ru–N(pyridine) 2.101, Ru–N(triazine) 2.053, Ru–Cl 2.412, Ru–C(average) 2.227, C(triazine)–N(NCS) 1.845. Selected computed bond lengths for **2**^**int**^**d-B**^**‡**^ (Å): Ru–N(pyridine) 2.104, Ru–N(triazine) 2.054, Ru–Cl 2.420, Ru–C(average) 2.220, C(triazine)–N(NCS) 1.777.

**Scheme 5:**
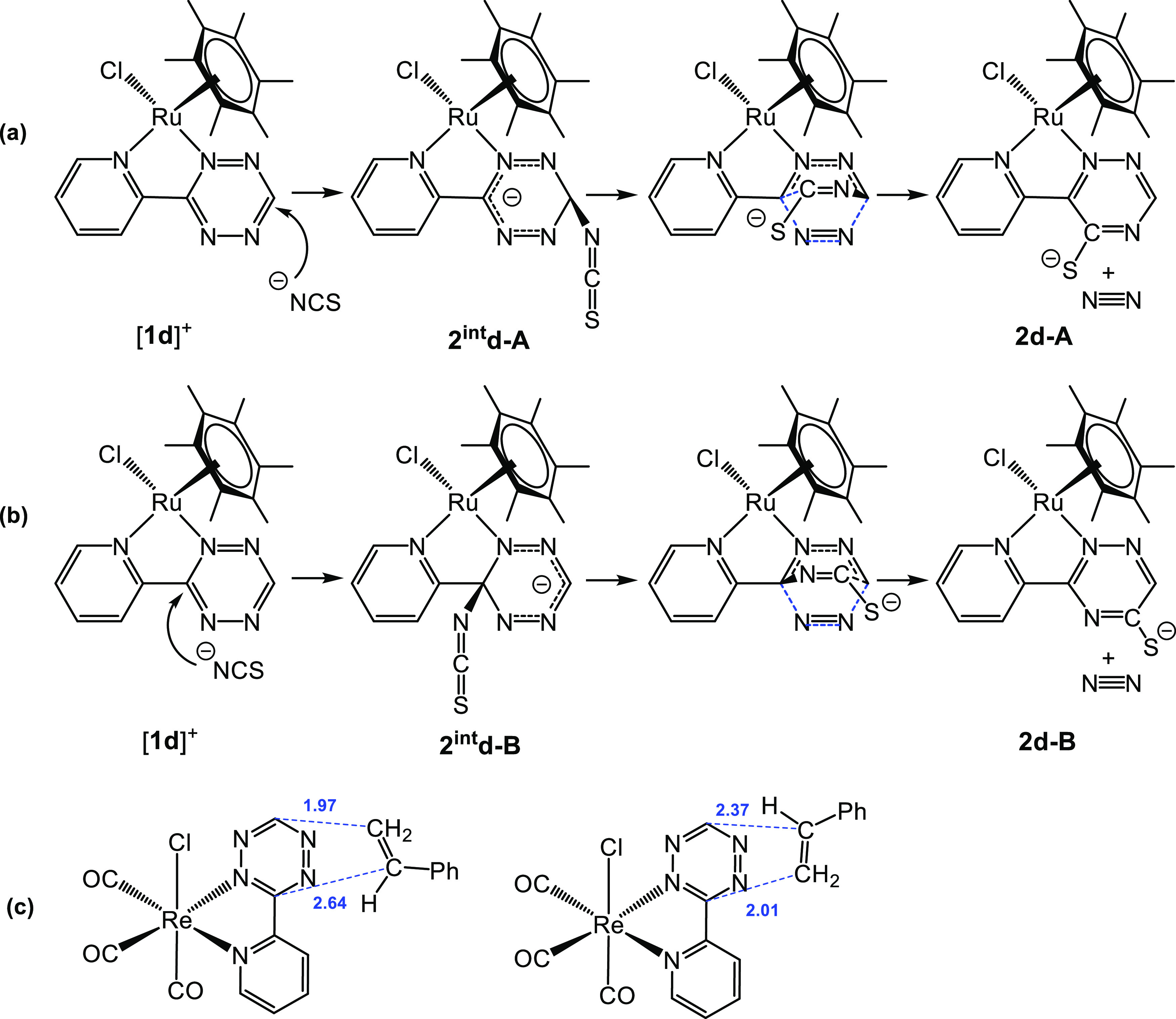
Proposed Pathways for the Reaction of [**1d**]^+^ with Thiocyanate: Nitrogen Addition on the CH or C-Pyridyl of the Tetrazine Ring, Affording the Experimentally Observed **2d-A** (a) or the Regioisomer **2d-B** (b), Respectively; Sketch of the Transition States (Computed C–C Distances in Å) for the Addition of Styrene to [ReCl(CO)_3_{κ^2^*N*-3-(2-pyridyl)-1,2,4,5-tetrazine}] (c)

It is reasonable to assume that the reaction starts with the nucleophilic attack of the thiocyanate nitrogen atom on an electron-poor carbon atom of the tetrazine moiety. On considering [**1d**]^+^ as a reactant, the interaction of the tetrazine CH with NCS^–^ should afford the intermediate **2**^**int**^**d-A**, converting to the kinetic product **2d-A** by the formation of a new C–C bond and elimination of N_2_ (Scheme 5a). On the other hand, thiocyanate attack on the pyridine-bonded *ipso*-C leads to the intermediate **2**^**int**^**d-B** and finally to **2d-B** (Scheme 5b). The structures of both intermediates were optimized and **2**^**int**^**d-A** is more stable than **2**^**int**^**d-B** by 5.1 kcal·mol^–1^ at C-PCM/TPSS0/def2-TZVP level (3.7 kcal·mol^–1^ with C-PCM/PBEh-3c calculations), accordingly to the experimental outcomes (Figure 3). The transition states for the two nucleophilic attacks were simulated both at C-PCM/TPSS0/def2-TZVP and C-PCM/PBEh-3c levels of theory. In the first case, the transition state affording **2**^**int**^**d-A** (imaginary frequency i381 cm^–1^) is more accessible than the one related to **2**^**int**^**d-B** (i353 cm^–1^) by about 2.7 kcal·mol^–1^. C-PCM/PBEh-3c outcomes resulted qualitatively comparable, with **2**^**int**^**d-A**^**‡**^ (imaginary frequency i411 cm^–1^) more stable than **2**^**int**^**d-B**^**‡**^ (i385 cm^–1^) by about 1.1 kcal·mol^–1^. All of the calculations therefore support the kinetic nature of the observed product **2d-A**. Interestingly, also in the related reaction between styrene and [ReCl(CO)_3_{κ^2^*N*-3-(2-pyridyl)-1,2,4,5-tetrazine}] (Scheme 1b), the regioselectivity is driven by a kinetically favorable interaction between the terminal styrene carbon and the less hindered tetrazine carbon (Scheme 5c).^22^ Despite the low bond polarization of the alkene, if compared to thiocyanate, the transition states are rather asynchronous and the energy difference between them is significant (4.7 kcal·mol^–1^), while the final ortho- or meta-disubstituted dihydropyridazine products are practically isoenergetic.

Compounds **2a**–**e** were characterized by spectroscopic (solid state IR, ^1^H, ^13^C and 2D NMR, UV–vis), analytical (CHNS content, molar conductivity), and spectrometric (electrospray ionization mass spectrometry (ESI-MS)) techniques; IR, NMR, and ESI-MS spectra are supplied in Figures S17–S38. Several peculiar changes in ^1^H and ^13^C NMR resonances can be noticed on moving from the tetrazine to the triazine-thione structure (Tables S1 and S2). The newly generated CS moiety manifests itself with a ^13^C NMR resonance at *ca*. 185 ppm: such chemical shift value is within the expected range for a thioketone.^23^ The characteristic singlet for the N_2_C–H proton (p7 in Scheme 4), at 10.7 ppm in the ^1^H NMR spectra of [**1a**]^+^/[**1d**]^+^, was detected around 8.3–8.4 ppm for **2a**/**2d** and showed a correlation with the sulfur-bonded carbon in the ^1^H–^13^C heteronuclear multiple bond correlation (HMBC) map (Figures S21 and S34). Instead, no correlation between the CS carbon and the N_2_C–CH_3_ protons is present in the HMBC spectra of **2b** and **2e** (Figures S27 and S38), in agreement with the small H-C coupling constant associated with a four-bond distance.^24^ Note that a strong HMBC signal (^3^*J*_CH_) would be expected for the putative isomer of **2b**/**2e** featuring the CS group next to the methyl substituent (isomer **B** in Scheme 4). The ^1^H NMR resonances of the *N*,*N*-heterocyclic and η^6^-arene ligands experienced a generalized shielding (−0.1 to −0.9 ppm), as expected for the transformation of a cation [**1**]^+^ into a neutral complex **2**. The exception to this trend is the pyridyl proton next to the triazine ring (p4 in Scheme 4) that becomes *ca*. 2 ppm downfield shifted. Such deshielding is in accordance with an intramolecular C–H···S interaction.^25^ Note that this interaction would not be present if the insertion of the {SCN} group took place with the opposite regiochemistry (isomer **B**). Similar patterns are noticed for the ^13^C{^1^H} NMR data: a generalized shielding of the η^6^-arene and *N*,*N*-heterocyclic resonances accompanies the transformation of [**1a**–**e**]^+^ into **2a**–**e**, except for the C–H group discussed above (p4) and the quaternary pyridyl carbon (p5). Interestingly, the other carbon connecting the *N*-heterocyclic rings (p6) undergoes a marked upfield shift (*ca*. −12 ppm), reflecting the change in bonding from C/N/N (tetrazine) to C/N/C (triazine-thione).

**Figure 4:**
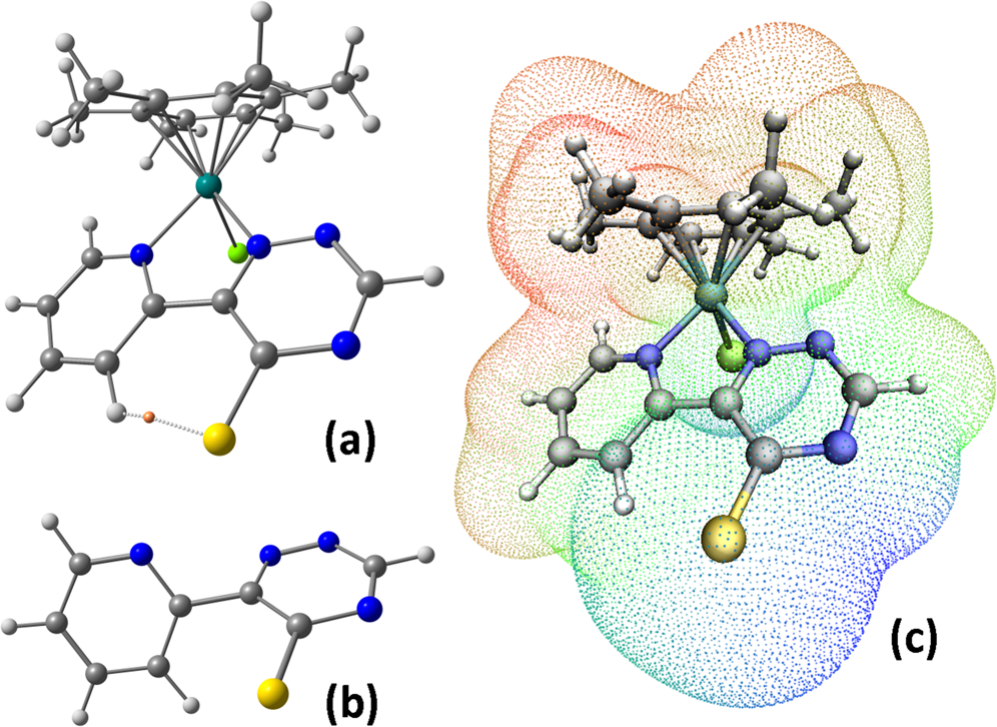
(a) DFT-optimized structure of **2d-A** with (3,–1) b.c.p. between S and CH represented by an orange sphere. (b) DFT-optimized structure of the free 6-(2-pyridyl)-1,2,4-triazine-5-thiolate ligand. (c) Electron density surface of **2d-A** (isovalue 0.001 au) with electrostatic potential (ESP) mapped. C-PCM/TPSS0-def2-TZVP calculations, acetone as continuous medium. Color map: Ru, dark green; Cl, green; S, yellow; N, blue; C, gray; H, white.

The presence of an H···S interaction was confirmed by the atoms in molecules (AIM) analysis on **2d-A**, which allowed to localize a (3,–1) bond critical point (b.c.p.) between the two attractors, as observable in Figure 4a. Selected properties at b.c.p. calculated at C-PCM/TPSS0-def2-TZVP level are electron density (ρ) 0.147 e·Å^–3^ and Laplacian of electron density (∇^2^ρ) 1.416 e·Å^–5^. The positive value of ∇^2^ρ reveals the closed-shell nature of the interaction, but it is worth noting that the ρ value is about double of that computed for the H_2_S···H_2_S hydrogen-bonded complex.^26^

To the best of our knowledge, crystal structures involving an ortho (hetero)aryl-substituted thiophenolate system are confined to 1-(2-pyridyl)pyridinium-2-thiolate^27^ and 2,2′-bipyridin-1-ium-3,3′-dithiolate.^28^ Interestingly, the former shows a 90° angle between the two aromatic rings while the latter is planar and features intermolecular C–H···S contacts. Such interaction was not detected in the DFT-optimized structure of the 6-(2-pyridyl)-1,2,4-triazine-5-thiolate ligand (Figure 4b), where the two rings are meaningfully less coplanar. In fact, the angle between the mean planes determined by the two heterocycles is 58.8° for the free ligand while it is 13.3° in **2d-A**. In this respect, the enforced planarity of the bis-heterocyclic ring due to the chelate coordination in **2a**–**e** is crucial for the C–H···S interaction.

From the map of the electrostatic potential (ESP) plotted on the electron density surface, the negative charge of the coordinated (2-pyridyl)-5-thioxo-1,2,4-triazinide ligand in **2d** is mainly localized on the sulfur atom and on the nitrogen adjacent to the {CS} unit (Figure 4c). Therefore, resonance structures **I** and **IV** in Scheme 3 best represent the electronic distribution in **2a**–**e**.

**Figure 5:**
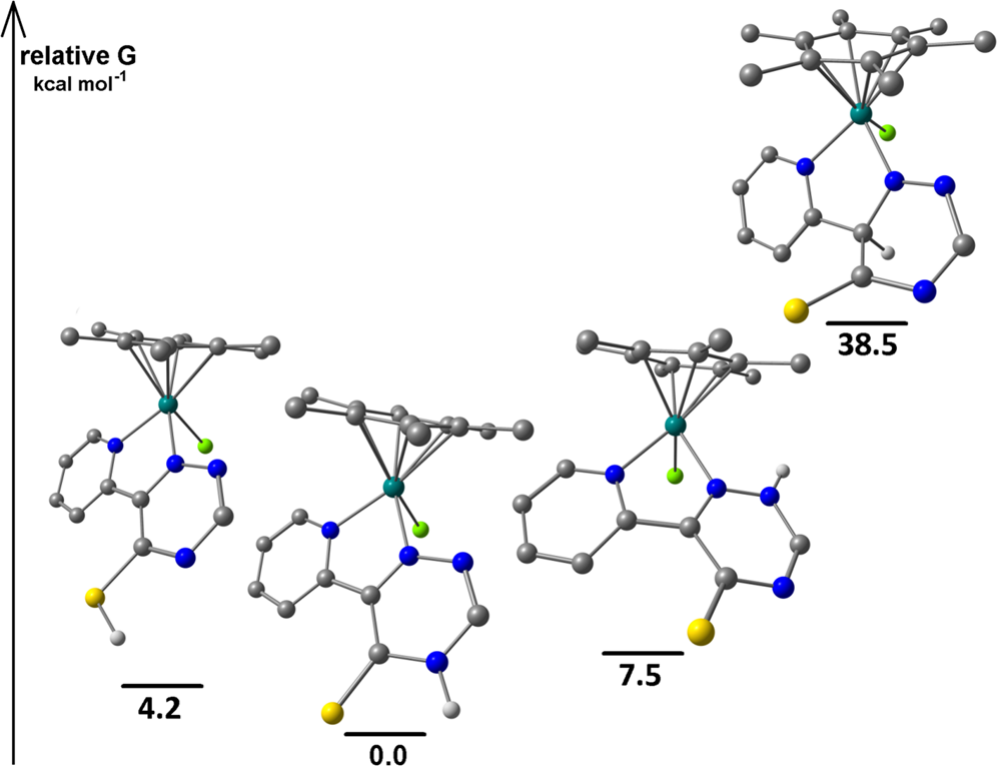
DFT-optimized structures and relative Gibbs free energies of [**2dH**]^+^ tautomers (C-PCM/TPSS0/def2-TZVP, acetone as continuous medium). Color map: Ru, dark green; Cl, green; S, yellow; N, blue; C, gray; H, white. Only the acidic proton is shown for clarity. Selected computed bond lengths for the most stable isomer: Ru–N(pyridine) 2.062, Ru–N(triazine) 2.024, Ru–Cl 2.405, Ru–C(average) 2.238, N–H 1.013.

**Scheme 6:**
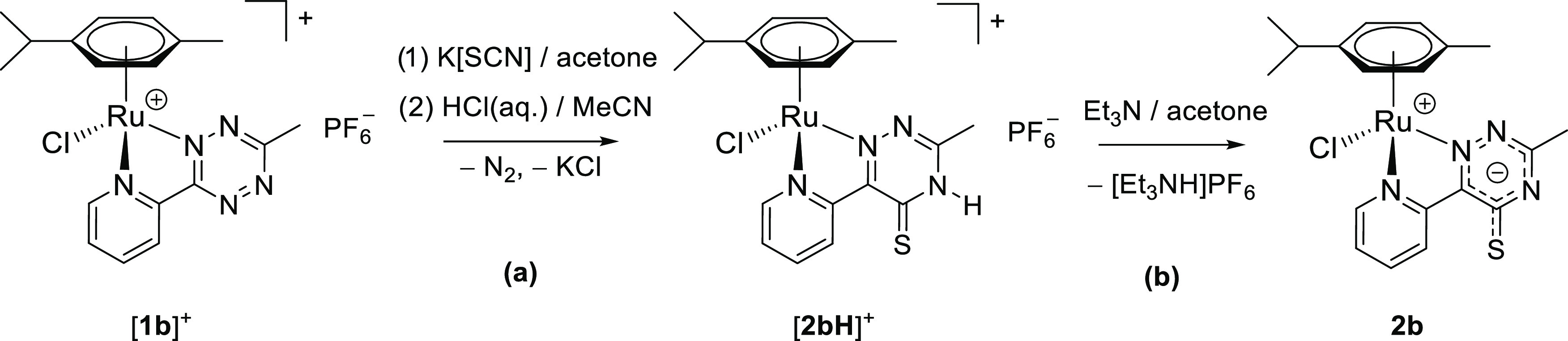
One-Pot Preparation of [**2bH**]PF_6_ from [**1b**]PF_6_ (a) and Subsequent Deprotonation (b) Reactions were carried out at room temperature in stoichiometric conditions. Complex [**2bH**]^+^ is represented as the most stable tautomer.

The stability of **2b**, as a representative compound, was checked in acetone-*d*_6_ or CD_3_CN solution at room temperature and no NMR-detectable changes were observed after 48 or 24 h, respectively. A minor decomposition of **2b** occurred in the solid state over several months under N_2_. Therefore, compounds **2a**–**e** are best stored at −20 °C under N_2_. Complex **2b** reacted straightforwardly with strong Brønsted acids (hydrochloric, triflic, and *p*-toluenesulfonic acid) to give the mono-protonated derivative [**2bH**]^+^, even under forced conditions (MeCN, 50 °C). On a preparative scale, [**2bH**]PF_6_ was isolated as a red-brown solid in 64% yield following a one-pot procedure from [**1b**]PF_6_ (Scheme 6a) and was characterized by analytical (CNHS analyses, molar conductivity) and spectroscopic (IR, NMR, UV–vis) techniques (Figures S39–S45). Compound **2b** was selectively regenerated from [**2bH**]^+^ by treatment with Et_3_N in acetone (Scheme 6b). The protonation/deprotonation can be easily monitored by UV–vis at a comparatively low ruthenium concentration (2.0 × 10^–4^ M), taking advantage of the intense absorptions of [**2bH**]^+^ around 430–465 nm (Figure S46). In principle, four different isomers of [**2bH**]^+^ can be drawn. However, only one set of signals was detected in the ^1^H and ^13^C{^1^H} NMR spectra of [**2bH**]^+^, indicating the presence of a single tautomer in solution. As a matter of fact, the relative Gibbs energy values of the DFT-optimized isomers of [**2dH**]^+^ strongly support a thioketone structure (Figure 5). Previous studies on the related 2-pyridine thione (2-mercaptopyridine) also indicated the predominance of the thioamide structure over the thiol tautomer.^29^

The NH–C=S moiety of [**2bH**]^+^ is manifested by a ^13^C NMR resonance at 177 ppm and a broad ^1^H NMR signal at 5.4 ppm in a concentrated acetone-*d*_6_ solution. All of the ^1^H NMR resonances of [**2bH**]^+^ are deshielded with respect to **2b** (protonation shift,^30^ except for the pyridyl proton adjacent to the sulfur atom (p4), which is 0.6 ppm upfield shifted) (Table S1).

### 2.2 Reactivity of Coordinated Tetrazines with Selenocyanate and Cyanate

Inspired by the results obtained with thiocyanate, we investigated the reactivity of the pyridyl tetrazine ruthenium complexes with the valence isoelectronic cyanate and selenocyanate anions.

**Scheme 7:**
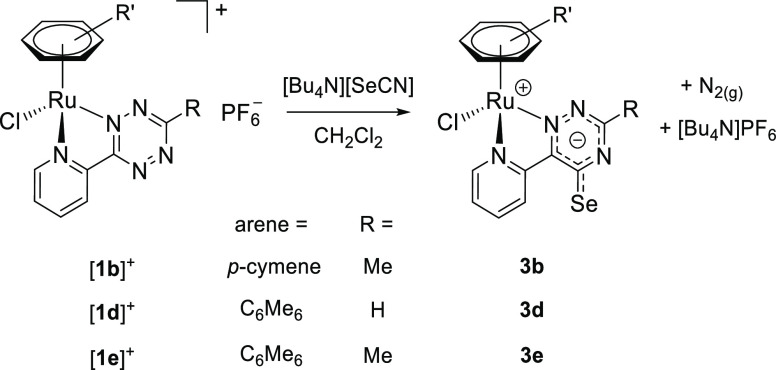
Synthesis of Pyridyl–Triazine–Selone Complexes **3b**, **3d**, and **3e** by Reaction of the Tetrazine Complexes [**1b**]^+^, [**1d**]^+^, and [**1e**]^+^ with Tetrabutylammonium Selenocyanate Reactions were carried out at room temperature in stoichiometric conditions.

The reactions of [**1b**]PF_6_, [**1d**]PF_6_, and [**1e**]PF_6_ with [Bu_4_N][SeCN] in CH_2_Cl_2_ under anhydrous conditions proceeded with quantitative conversion and led to the formation of **3b**, **3d**, and **3e** featuring a (2-pyridyl)-5-selenoxo-1,2,4-triazinide ligand (Scheme 7). As in the previous case, the new heterocycle will be referred to as triazine-selone, with reference to the resonance form with a C=Se double bond, while the structure of the zwitterionic compounds **3b**, **3d**, and **3e** is depicted with a delocalized anionic charge. In the case of [**1b**]^+^, several minor arene byproducts and a significant amount of free *p*-cymene were detected by ^1^H NMR (Figure S47). Better results were obtained with the more stable η^6^-hexamethylbenzene complexes [**1d**]^+^ and [**1e**]^+^. Following diethyl ether/acetone washings, compounds **3d** and **3e** were obtained as brown solids, in admixture with the co-product [Bu_4_N]PF_6_.^31^ To the best of our knowledge, no example of 1,2,4-triazine-5-selone or related structures has been reported so far;^32^ the closest result is represented by a selenated 6-azauracil derivative (4,5-dihydro-2,4-dimethyl-6-phenyl-5-selenoxo-1,2,4-triazin-3(2*H*)-one).^23b,33^

Compounds **3d** and **3e** were characterized by spectroscopic (IR; ^1^H, ^13^C, ^77^Se, and 2D NMR) and spectrometric (ESI-MS) techniques (Figures S48–S61). ^1^H and ^13^C NMR features of **3b**, **3d**, and **3e** resemble those described for the sulfur analogues **2b**, **2d**, and **2e** (Tables S1 and S2). In the ^13^C NMR spectra, a diagnostic signal at *ca*. 183 ppm was detected for the {CSe} moiety. The tetrazinyl proton in [**1d**]^+^ (10.66 ppm) becomes upfield shifted in **3d** (8.42 ppm) and coupled with the CSe carbon (^1^H–^13^C HMBC; Figure S52). The general trend in shielding of the ^1^H NMR resonances on moving from the tetrazine ([**1b**]^+^, [**1d**]^+^, and [**1e**]^+^) to the triazine-selone (**3b**, **3d**, and **3e**) structure is contrasted by the marked downfield shift (+2.3 ppm) of the pyridyl proton p4 (same C/H atom numbering as in Scheme 4), suggestive of an intramolecular C–H···Se interaction. Taken together, these data indicate that the addition of SeCN^–^ and SCN^–^ to the tetrazine ring occurred with the same regiochemistry (**isomer A** with reference to Scheme 4). The ^77^Se NMR spectra of **3d** and **3e** display a signal around 630 ppm or 580 ppm, respectively. By comparison, ^77^Se NMR resonances for related molecules sharing an acyclic selenamide fragment {C–C(=Se)–N} were reported between 510 and 733 ppm,^34^ while those for C=Se groups within a pyridyl or pyrimidyl ring were observed around 320 ppm.^35^

Chromatography of **3e** on a silica gel column resulted in a partial Se/O exchange, as indicated by MS data (Figure S62). In this respect, the sensitivity of the C=Se bond of related selenouracil derivatives to hydrolysis was previously reported.^35b^ Complex **3e** was reversibly protonated using HCl and Et_3_N in sequence; the protonated derivative [**3eH**]^+^ was formulated as the selenium analogue of [**2bH**]^+^ based on similar ^1^H NMR features (Table S1).

Finally, compounds [**1a**]PF_6_ and [**1b**]PF_6_ reacted very rapidly and quantitatively with a stoichiometric amount of [Bu_4_N][OCN] in acetone or CH_2_Cl_2_. However, differently from the previous cases, two sets of signals for Ru(*p*-cymene) species were found in the ^1^H NMR spectra, one of which is considerably broadened (Figures S63 and S64). Addition of excess *p*-toluenesulfonic acid to the [**1b**]^+^/OCN^–^ reaction mixture led to two new sharp sets of ^1^H NMR signals without any significant downfield shift, as would have been expected upon protonation. The IR spectrum of the [**1a**]^+^/OCN^–^ reaction ruled out the occurrence of chloride/cyanate exchange (Figure S65, see also Section 2.4). It is reasonable to assume that cyanate reacted with the tetrazine ring of [**1a**]^+^ and [**1b**]^+^ and that one of the products could be the oxygen analogue of **2a** and **2b**. Nevertheless, detailed investigations were frustrated by the marked instability of these complexes in solution and the high sensitivity of the system to the reaction conditions.

Under similar conditions, the reactions of [**1a**]^+^ and [**1b**]^+^ with cyclohexyl or xylyl isocyanide or tetrabutylammonium cyanide afforded complex mixtures of products that could not be identified.

### 2.3 Chalcogenocyanate/Tetrazine Reactivity: UV–Vis Monitoring and Control Experiments

The reactivity of pyridyl tetrazine ruthenium complexes with chalcogenocyanate anions was also investigated by monitoring the peculiar changes in the UV–vis absorptions.^36^ Therefore, *ca*. 2 × 10^–4^ M acetone solutions of [**1a**]PF_6_ or [**1b**]PF_6_ were spiked with an equimolar amount of [Bu_4_N][ECN] (E = O, S, Se) or KSCN and the UV–vis spectra were recorded over the next 14 h (Figures S66–S73). The absorption at 470 nm due to the pyridyl tetrazine complexes [**1a**]^+^ and [**1b**]^+^ was quickly replaced by new bands due to the reaction with cyanate (370 and 385 nm), thiocyanate (350 and 400 nm) or selenocyanate (375 nm). The reactions of [**1a**]^+^ were also characterized by the development of a broad, shoulder band in the 550–750 nm range. The UV–vis data allowed to delineate conversion-time profiles (Figures S74 and S75) and to calculate second-order rate constants (Table 1). The rate of the reaction decreases with the size of the chalcogen (O ≫ S > Se) for both tetrazine complexes. Even at sub-micromolar concentrations (≈2 × 10^–4^ mol·L^–1^), the reactions with cyanate were practically complete within seconds (however, the solutions are metastable, *vide supra*) and the kinetic constants could not be calculated. The introduction of a methyl substituent ([**1b**]^+^) in place of hydrogen ([**1a**]^+^) in the tetrazine ring leads to a 4- to 7-fold decrease in the reaction rate. Conversely, the change in counter cation (K^+^*vs* [Bu_4_N]^+^) of thiocyanate produces a minor effect on the kinetics. The decomposition of the tetrazine complexes [**1a**]^+^ and [**1b**]^+^ in acetone, which can be appreciated in the UV–vis spectra in Figures S76 and S77, occurs on a much longer time scale than their reactions with chalcogenocyanate ions (Table 1).

**Table 1:** Second-Order Rate Constants (*k*_2_/M^–1^·s^–1^) Determined for the Reactions of [**1a**]^+^ and [**1b**]^+^ with Thiocyanate or Selenocyanate in Acetone at Room Temperature (21 ± 1 °C)

reactant(s)	–a	K[SCN]	[Bu_4_N][SCN]	[Bu_4_N][SeCN]
[**1a**]PF_6_	0.11	59	72	4.3
[**1b**]PF_6_	3.8 × 10^–2^	8	10	1.1

a Rate constants of the decomposition of the tetrazine complexes in acetone fitted as a second-order reaction.

Finally, a series of reactions were conducted with free tetrazines and chalcogenocyanate salts, aiming to assess the role of ruthenium coordination in the observed reactivity. All of the tested combinations between 3-pyridyl, 3-pyridyl-6-methyl, 3,6-diphenyl or 3,6-dipyridyl 1,2,4,5-tetrazines and cyanate, thiocyanate or selenocyanate (K^+^, Bu_4_N^+^ or Et_3_NH^+^ salts) gave no evidence of reactivity by ^1^H NMR after 24 h in CH_2_Cl_2_ at room temperature or in refluxing acetone. Interestingly, the reactions of hydrated Fe(ClO_4_)_2_, Mn(ClO_4_)_2_ and Cu(NO_3_)_2_ with the nonchelating 3,6-bis(4-pyridyl)-1,2,4,5-tetrazine and K[ECN] (E = S, Se) or NH_4_[SCN] led to the coordination of the pyridine and chalcogenocyanate anions, leaving the noncoordinated tetrazine ring unaffected.^37^ Besides, [**1a**]^+^ was totally unreactive toward neutral chalcogenocyanate derivatives such as phenyl isocyanate and isopropyl isothiocyanate. These results demonstrate that the coordination of the pyridyl tetrazines to cationic ruthenium(II) arene scaffolds enables their reactivity with the chalcogenocyanate anions. Notably, the reactions of [**1a**]^+^ with thiocyanate or selenocyanate are (4–5) × 10^3^ or 3 × 10^2^ times faster, respectively, than the reaction with ethynylferrocene (*k*_2_ = 1.4 × 10^–2^ M^–1^·s^–1^ in CD_2_Cl_2_ at 20 °C).^16c^ The opposite ionic charges of the reactants possibly play a key (favorable) role for this reactivity in organic solutions (*e.g.*, initial formation of an ion pair).

### 2.4 Chalcogenocyanate Coordination to a Related Ruthenium(II) Arene System

**Scheme 8:**
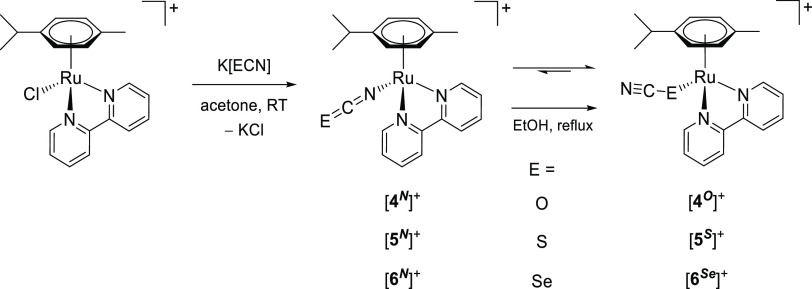
Synthesis of Chalcogenocyanato-Bipyridine Complexes [**4**–**6**]^+^ by Chloride/Chalcogenocyanate Exchange and Subsequent Thermal Treatment Reactions were carried out in stoichiometric conditions; cationic complexes as PF_6_^–^ salts.

Chalcogenocyanates, particularly thiocyanate, are well-known ambidentate ligands in coordination chemistry.^38^ As checked by the IR spectra, a competitive chalcogenocyanate coordination was generally not observed in their reactions with pyridyl tetrazine complexes [**1a**–**e**]^+^, except in some cases with more concentrated reaction mixtures (appearance of small intensity bands in the 2000–2200 cm^–1^ region). In order to collect reference spectroscopic (IR/NMR) data for chalcogenocyanate ligands in a structurally related ruthenium(II) arene system, we employed 2,2′-bipyridine as an isoelectronic analogue of the 3-(2-pyridyl)-1,2,4,5-tetrazine. Therefore, [RuCl(2,2′-bipyridine)(η^6^-*p*-cymene)]PF_6_ was prepared, X-ray characterized (Figure S82 and Table S3), and allowed to react with K[ECN] (E = S, Se) in acetone or Na[OCN] in acetone/water at room temperature (Scheme 8). Following removal of the insoluble sodium/potassium chloride, [Ru(ECN)(2,2′-bipyridine)(η^6^-*p*-cymene)]^+^ complexes were isolated as yellow (E = O, [**4**]^+^), orange (E = S, [**5**]^+^) or orange-red (E = Se, [**6**]^+^) hygroscopic hexafluorophosphate salts in 82–90% yield. Compounds [**4**–**6**]PF_6_ were (almost always) obtained as a mixture of isomers, reflecting the ambidentate character of chalcogenocyanate ligands. A comparable number of ruthenium(II) arene complexes with either κ*S* or κ*N* coordinated thiocyanate have been reported, being often isolated as mixtures of isomers.^39^ Conversely, only a dozen cyanato complexes have been described,^40^ all existing as κ*N*-coordinated isomers as established by spectroscopic and structural evidence, while no selenocyanato complex has been reported to date. The previously reported [**5**]PF_6_^39b^ and the unprecedented [**4**]PF_6_ and [**6**]PF_6_ were characterized by solid-state IR and ^1^H, ^13^C NMR in acetone-*d*_6_ (Figures S83–S98). Coordination of the chalcogenocyanate *via* the nitrogen or the chalcogen atom was assessed by the relevant spectroscopic data (Table S4). Based on IR and/or ^13^C NMR data for the C≡N moiety,^41^ the κ*N* isomer was largely predominant for thiocyanate ([**5**^***N***^]^+^: 2049 cm^–1^; 131 ppm) and selenocyanate ([**6**^***N***^]^+^: 2058 cm^–1^; 131 ppm). Instead, an almost equimolar mixture of κ*N* and κ*O* cyanate isomers ([**4**^***N***^]^+^/[**4**^***O***^]^+^: broad band at 2217 cm^–1^; 127.8/128.1 ppm) was obtained. Next, a portion of the isolated [**4**–**6**]PF_6_ was suspended in refluxing EtOH for 14 h. Isomerization to the chalcogeno-bonded isomers [**5**^***S***^]^+^ (2103 cm^–1^, 118 ppm) and [**6**^***Se***^]^+^ (2113 cm^–1^, 103 ppm) was observed, indicative of their higher thermodynamic stability. The final κ*N*/κ*S* ratio (0.23) for [**5**]^+^ is in agreement with the that previously reported in MeOH at 50 °C (0.29).^39b^ Therefore, the conditions herein employed (acetone, room temperature) allowed us to obtain a mixture of the thiocyanate and selenocyanate complexes highly enriched in the kinetic κ*N-*coordinated isomer. In one case, [**5**^***N***^]PF_6_ was selectively obtained. Conversely, no change in molar ratio was observed for cyanato complexes [**4**]^+^, suggesting that the isomeric mixture was already at equilibrium. Notably, the selenocyanate isomers can be easily distinguished by ^77^Se NMR: the resonance for [**6**^***N***^]^+^ (−303 ppm) is close to that of ionic SeCN^–^ (−300 ppm) while that of [**6**^***Se***^]^+^ is markedly downfield shifted (−106 ppm).

## 3 Conclusions

Tetrazines are reactive heterocycles that are prone to N_2_ elimination; however, their coordination to a metal center may be crucial to enable the reactions with unsaturated organic species. Herein we report the first study on the reactivity of 1,2,4,5-tetrazines with chalcogenocyanate anions, taking advantage of their coordination within bidentate pyridyl tetrazine ligands to {RuCl(η^6^-arene)}^+^ scaffolds.

The reactions with stoichiometric amounts of thiocyanate and selenocyanate salts revealed to be straightforward at room temperature, allowing the regioselective formation of a triazine chalcogenone heterocycle within a zwitterionic Ru complex. According to DFT calculations, the reaction is initiated by the nucleophilic attack of the N atom of thiocyanate to the kinetically favored tetrazine carbon. On the other hand, reactions with cyanate proceeded differently and a detailed analysis of the products was prevented by their instability. The reactions of chalcogenocyanate salts with ruthenium tetrazine complexes are relatively fast at room temperature and their kinetics depend on the chalcogen atom (O ≫ S > Se) and the tetrazine substituents (H > Me). A triazine-thione complex undergoes a fully reversible protonation on the heterocyclic ring with strong Brønsted acids in wet organic solvents, highlighting the robust coordination of the *N*,*N*-bidentate ligand to the ruthenium center.

Overall, these results are of relevance especially concerning the triazine-selone species, which are unprecedented in both organic and organometallic chemistry. On the other hand, a number of triazine-thione compounds have been previously reported by exploiting synthetic approaches different from the one described here. This work provides a further example of the synthetic opportunities offered by the coordination of an unsaturated organic compound to a transition metal. In the present case, the ruthenium arene scaffold plays a crucial role to address the reaction outcome, and in particular the net cationic charge favors the nucleophilic addition of the chalcogenocyanate anion to the less hindered carbon of the tetrazine. Notably, the alternative replacement of the chloride ligand by the chalcogenocyanate is avoided, otherwise it is viable in analogous systems lacking the N_2_-elimination route (2,2′-bipyridine). Although, in the present case, the stable metal binding of the obtained heterocycles prevents their facile dissociation and isolation, our strategy may open the door to the future development of convenient synthetic protocols to access functionalized triazines. Furthermore, this novel reactivity could be valuable for tetrazine ligation procedures.

## 4 Experimental Section

### 4.1 General Experimental Details

RuCl_3_ hydrate was purchased from Strem Chemicals, while other reactants and solvents were obtained from Merck, Apollo Scientific, or TCI Chemicals and were of the highest purity available. Compounds 3-(2-pyridyl)-1,2,4,5-tetrazine and 3-(2-pyridyl)-6-methyl-1,2,4,5-tetrazine were purchased from TTI GmbH/TGU Varimol (www.varimol.de) and stored under N_2_ at 4 °C or −20 °C (see note in the SI). Compounds [RuCl_2_(η^6^-arene)]_2_ (arene = *p*-cymene, C_6_Me_6_),^42^ [RuCl(2,2′-bipyridine)(η^6^-*p*-cymene)]PF_6_,^43^ 3,6-di(2-pyridyl)-1,2,4,5-tetrazine,^44^ and 3,6-diphenyl-1,2,4,5-tetrazine^45^ were synthesized according to literature methods. Where specified, the reactions were carried out under dry N_2_ using standard Schlenk techniques and anhydrous CH_2_CH_2_, obtained from SPS 5 solvent purifier (MBraun) and stored over 3 Å MS. All of the other reactions and operations were carried out in air with common laboratory glassware. Chromatographic separations were carried out on silica gel columns (70–230 mesh). All isolated Ru complexes were manipulated in air for short periods of time, but they were kept under N_2_ at −20 °C for long-term storage as a precaution. Carbon, hydrogen, nitrogen, and sulfur analyses were performed on a Vario MICRO cube instrument (Elementar). IR spectra of solid samples (650–4000 cm^–1^) were recorded on an Agilent Cary 630 FTIR spectrometer equipped with a UATR sampling accessory. NMR spectra were recorded on JEOL YH JNM-ECZ400S or JNM-ECZ500R instruments equipped with a Royal HFX broad band probe. CDCl_3_ was stored in the dark over K_2_CO_3_. Chemical shifts are referenced to the residual solvent peaks (^1^H, ^13^C) or to external standards (^19^F to CFCl_3_, ^31^P to 85% H_3_PO_4_, ^77^Se to Me_2_Se).^46^^1^H and ^13^C spectra were assigned with the assistance of ^1^H–^13^C *gs*-HSQC and *gs*-HMBC experiments (long range *J* = 8 Hz, Δ_2_ = 62.5 ms). UV–vis spectra were recorded on an Ultraspec 2100 Pro spectrophotometer using quartz cuvettes (1 cm pathlength). IR and UV–vis spectra were processed with Spectragryph.^47^ Conductivity measurements were carried out using an XS COND 8 instrument (cell constant = 1.0 cm^–1^)^48^ equipped with NT 55 temperature probe (measurements automatically adjusted to 25 °C). ESI-Q/ToF flow injection analyses (FIA) were carried out using a 1200 Infinity HPLC (Agilent Technologies), coupled to a Jet Stream ESI interface (Agilent) with a Quadrupole-Time of Flight tandem mass spectrometer 6530 Infinity Q-TOF (Agilent Technologies). HPLC-MS grade acetonitrile was used as mobile phase (Carlo Erba, Italy). The flow rate was 0.2 mL·min^–1^ (total run time 3 min). Samples were weighed, dissolved in HPLC-MS grade acetonitrile, and diluted to 10 ppm prior to injection. Injection volume: 0.1 μL. ESI operating conditions: drying gas (N_2_, purity >98%): 350 °C and 10 L·min^–1^; capillary voltage 4.5 KV; nozzle voltage: 1 kV; nebulizer gas 35 psig; sheath gas (N_2_, purity >98%): 375 °C and 11 L·min^–1^. The fragmentor was kept at 50 V, the skimmer at 65 V, and the OCT 1 RF at 750 V. High-resolution MS spectra were achieved in positive mode in the range 100–1700 *m*/*z*; and the mass axis was calibrated daily using the Agilent tuning mix HP0321 (Agilent Technologies) prepared in acetonitrile and water.

### 4.2 Synthesis and Characterization of Pyridyl Tetrazine Complexes

#### 4.2.1 General Procedure

An orange suspension of [RuCl_2_(η^6^-arene)]_2_ (50–150 mg; arene = *p*-cymene, C_6_Me_6_) in MeCN (3 mL) was treated with NH_4_PF_6_ (2.05 equiv) and stirred at room temperature for 1 h. The resulting suspension (yellow-orange solution + colorless solid) was filtered over a celite pad, and the filtrate was taken to dryness under vacuum. The residue was treated with the selected pyridyl tetrazine (2.0 equiv) and dry CH_2_Cl_2_ (*ca.* 5 mL). The mixture was stirred at room temperature under a N_2_ atmosphere and protected from ambient light (this step can alternatively be carried out in air for [**1a**]^+^ and [**1b**]^+^). After *ca.* 4 h, the mixture was filtered over a celite pad and taken to dryness under vacuum. The residue was triturated with Et_2_O ([**1a**–**c**]^+^) or Et_2_O/CHCl_3_ 1:1 v/v ([**1d**]^+^ and [**1e**]^+^) and filtered. The solid was washed with Et_2_O and hexane, dried under vacuum (40 °C), and maintained under N_2_ at −20 °C for long-term storage.

##### 4.2.1.1 [RuCl{κ^2^*N*-3-(2-pyridyl)-1,2,4,5-tetrazine}(η^6^-*p*-cymene)]PF_6_, [**1a**]PF_6_

**Chart 1:**
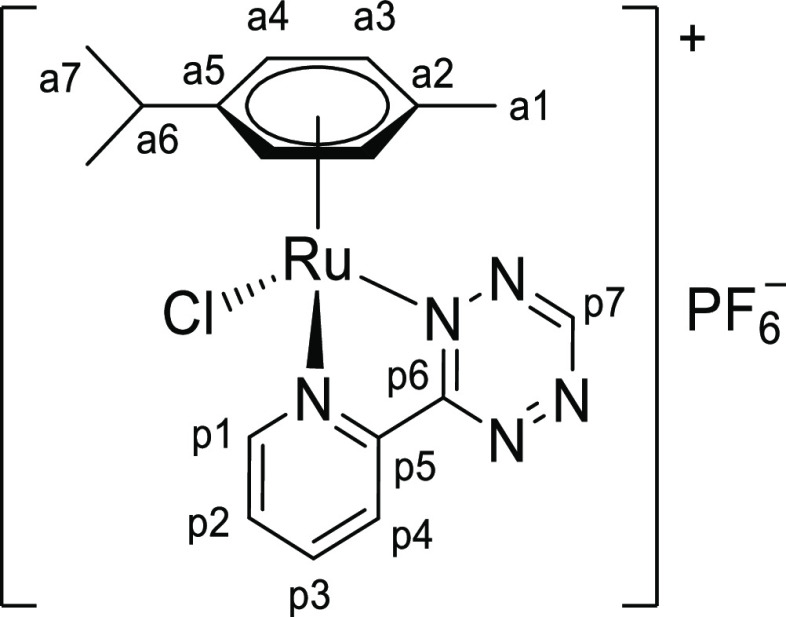
Structure of [**1a**]PF_6_ (Numbering Refers to C Atoms)^49^

Prepared from [RuCl_2_(η^6^-*p*-cymene)]_2_ (151 mg, 0.247 mmol) and 3-(2-pyridyl)-1,2,4,5-tetrazine (78 mg, 0.49 mmol). Dark red-brown solid, yield: 263 mg, 92%. Previously prepared by a similar procedure and characterized by ^1^H NMR (CD_2_Cl_2_), CNH analyses, and single-crystal X-ray diffraction (as the BAr^F^_4_^–^ salt);^16c^ new/additional data are herein reported. Soluble in CH_2_Cl_2_, MeOH, acetone, moderately soluble in CHCl_3_, poorly soluble in water, insoluble in diethyl ether. IR (solid state): ṽ/cm^–1^ = 3081w, 2966w, 1607w, 1507w, 1471w, 1453w, 1423w, 1390w, 1352m, 1295w, 1262w, 1155w, 1096w, 1061w, 1036w, 946w, 878w-sh, 834s (PF_6_), 769m-sh, 744m-sh, 690w, 674w. UV–vis (acetone, 2.2 × 10^–4^ M): λ/nm (ε/M^–1^·cm^–1^) = 471 (3.5 × 10^3^). Λ_m_ (acetone, 1.1 × 10^–3^ M) = 177 S·cm^2^·mol^–1^. ^1^H NMR (400 MHz, acetone-*d*_6_): δ/ppm = 10.70 (s, 1H, p7), 9.75 (d, ^3^*J*_HH_ = 5.3 Hz, 1H, p1), 9.00 (d, ^3^*J*_HH_ = 7.3 Hz, 1H, p4), 8.56 (td, ^3^*J*_HH_ = 7.8 Hz, ^4^*J*_HH_ = 1.1 Hz, 1H, p3), 8.15 (ddd, ^3^*J*_HH_ = 12.9, 7.0 Hz, ^4^*J*_HH_ = 4.2 Hz, 1H, p2), 6.39 (d, ^3^*J*_HH_ = 6.4 Hz, 1H, a4), 6.33 (d, ^3^*J*_HH_ = 6.5 Hz, 1H, a4′), 6.29 (d, ^3^*J*_HH_ = 6.6 Hz, 1H, a3), 6.09 (d, ^3^*J*_HH_ = 6.1 Hz, 1H, a3′), 2.96 (hept, ^3^*J*_HH_ = 6.9 Hz, 1H, a6), 2.24 (s, 3H, a1), 1.28 (d, ^3^*J*_HH_ = 6.9 Hz, 6H, a7 + a7′); an almost quantitative conversion in a mixture of unidentified products was observed after 14 h at room temperature. ^13^C{^1^H} NMR (126 MHz, acetone-*d*_6_): δ/ppm = 168.3 (p6), 159.4 (p7), 157.6 (p1), 150.0 (p5), 141.9 (p3), 132.1 (p2), 127.9 (p4), 110.8 (a5), 105.5 (a2), 92.0 (a4); 90.03, 90.01 (a4′ + a3), 89.5 (a3′), 31.4* (a6); 21.5* (a7 + a7′); 17.7* (a1). *From ^1^H–^13^C HSQC. ^19^F NMR (470 MHz, acetone-*d*_6_): δ/ppm = −144 (hept, ^1^*J*_FP_ = 711 Hz, PF_6_^–^). ^31^P NMR (202 MHz, acetone-*d*_6_): δ/ppm = −72.4 (d, ^1^*J*_PF_ = 708 Hz, PF_6_^–^). ^1^H NMR (400 MHz, CD_3_CN): δ/ppm = 10.37 (s, 1H, p7), 9.44 (d, ^3^*J*_HH_ = 5.3 Hz, 1H, p1), 8.86 (d, ^3^*J*_HH_ = 7.8 Hz, 1H, p4), 8.38 (t, ^3^*J*_HH_ = 7.7 Hz, 1H, p3), 7.99 (t, ^3^*J*_HH_ = 6.7 Hz, 1H, p2); 6.12 (d, ^3^*J*_HH_ = 6.2 Hz, 1H), 6.08 (d, ^3^*J*_HH_ = 6.3 Hz, 1H), 6.00 (d, ^3^*J*_HH_ = 6.3 Hz, 1H), 5.86 (d, ^3^*J*_HH_ = 6.2 Hz, 1H) (a3 + a3′ + a4 + a4′); 2.86 (hept, ^3^*J*_HH_ = 6.8 Hz, 1H, a6), 2.13 (s, 3H, a1), 1.21 (d, ^3^*J*_HH_ = 6.8 Hz, 6H, a7 + a7′); 5% *p*-cymene was observed after 24 h at room temperature (Chart 1).

##### 4.2.1.2 [RuCl{κ^2^*N*-3-(2-pyridyl)-6-methyl-1,2,4,5-tetrazine}(η^6^-p-cymene)]PF_6_, [**1b**]PF_6_

**Chart 2:**
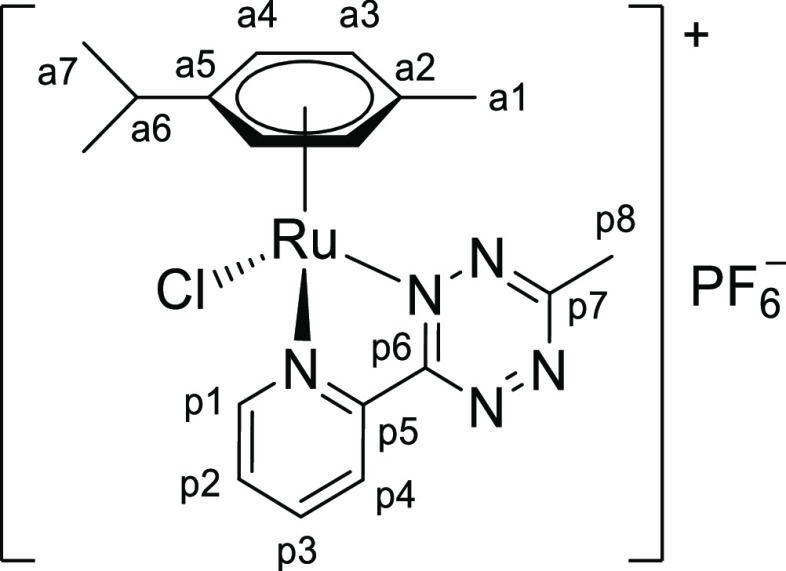
Structure of [**1b**]PF_6_ (Numbering Refers to C Atoms)^49^

Prepared from [RuCl_2_(η^6^-*p*-cymene)]_2_ (150 mg, 0.245 mmol) and 3-(2-pyridyl)-6-methyl-1,2,4,5-tetrazine (86 mg, 0.49 mmol). Dark red-brown solid, yield: 271 mg, 94%. Soluble in CH_2_Cl_2_, MeOH, acetone, moderately soluble in CHCl_3_, poorly soluble in water, and insoluble in diethyl ether. IR (solid state): ṽ/cm^–1^ = 3081w, 2970w, 2931w, 1607w, 1499w, 1471w, 1406m, 1370m, 1328w, 1283w, 1265w, 1248w, 1162w, 1135m, 1061w, 1027w, 933w, 878w, 829s (PF_6_), 768m-sh, 741m-sh, 688w. UV–vis (acetone, 2.0 × 10^–4^ M): λ/nm (ε/M^–1^·cm^–1^) = 468 (3.2 × 10^3^). Λ_m_ (acetone, 1.0 × 10^–3^ M) = 154 S·cm^2^·mol^–1^. ^1^H NMR (500 MHz, acetone-*d*_6_): δ/ppm = 9.71 (d, ^3^*J*_HH_ = 5.5 Hz, 1H, p1), 8.94 (d, ^3^*J*_HH_ = 7.9, 0.7 Hz, 1H, p4), 8.53 (td, ^3^*J*_HH_ = 7.8 Hz, ^4^*J*_HH_ = 1.2 Hz, 1H, p3), 8.11 (ddd, ^3^*J*_HH_ = 7.2, 5.7 Hz, ^4^*J*_HH_ = 1.4 Hz, 1H, p2), 6.39 (d, ^3^*J*_HH_ = 6.3 Hz, 1H, a4), 6.31 (d, ^3^*J*_HH_ = 6.4 Hz, 1H, a4′), 6.25 (d, ^3^*J*_HH_ = 6.4 Hz, 1H, a3), 6.07 (d, ^3^*J*_HH_ = 6.3 Hz, 1H, a3′), 3.29 (s, 3H, p8), 2.97 (hept, ^3^*J*_HH_ = 7.0 Hz, 1H, a6), 2.24 (s, 3H, a1), 1.263 (d, ^3^*J*_HH_ = 6.9 Hz, 3H, a7), 1.260 (d, ^3^*J*_HH_ = 7.0 Hz, 3H, a7′); no changes were observed after 48 h at room temperature. ^13^C{^1^H} NMR (126 MHz, acetone-*d*_6_): δ/ppm = 170.4 (p7), 165.7 (p6), 157.4 (p1), 150.2 (p5), 141.8 (p3), 131.6 (p2), 127.2 (p4), 110.4 (a5), 105.4 (a2), 91.8 (a4), 89.9 (a4′), 89.8 (a3), 89.1 (a3′), 31.9 (a6), 22.5 (a7), 21.9 (p8), 21.7 (a7′), 18.4 (a1). ^19^F NMR (470 MHz, acetone-*d*_6_): δ/ppm = −144 (hept, ^1^*J*_FP_ = 707 Hz, PF_6_^–^). ^31^P NMR (202 MHz, acetone-*d*_6_): δ/ppm = −73 (d, ^1^*J*_PF_ = 708 Hz, PF_6_^–^). ^1^H NMR (400 MHz, CD_3_CN): δ/ppm = 9.43 (d, ^3^*J*_HH_ = 5.1 Hz, 1H, p1), 8.81 (d, ^3^*J*_HH_ = 7.8 Hz, 1H, p4), 8.35 (t, ^3^*J*_HH_ = 7.7 Hz, 1H, p3), 7.95 (t, ^3^*J*_HH_ = 6.2 Hz, 1H, p2); 6.12 (d, ^3^*J*_HH_ = 6.0 Hz, 1H), 6.07 (d, ^3^*J*_HH_ = 6.2 Hz, 1H), 5.97 (d, ^3^*J*_HH_ = 6.2 Hz, 1H), 5.86 (d, ^3^*J*_HH_ = 6.1 Hz, 1H) (a3 + a3′ + a4 + a4′); 3.21 (s, 3H, p8), 2.86 (hept, ^3^*J*_HH_ = 6.8 Hz, 1H, a6), 2.13 (s, 3H, a1), 1.20 (d + d, ^3^*J*_HH_ = 6.8 Hz, 6H, a7 + a7′); 1% *p*-cymene was observed after 24 h at room temperature (Chart 2).

##### 4.2.1.3 [RuCl{κ^2^*N*-3,6-di(2-pyridyl)-1,2,4,5-tetrazine}(η^6^-*p*-cymene)]PF_6_, [1c]PF_6_

**Chart 3:**
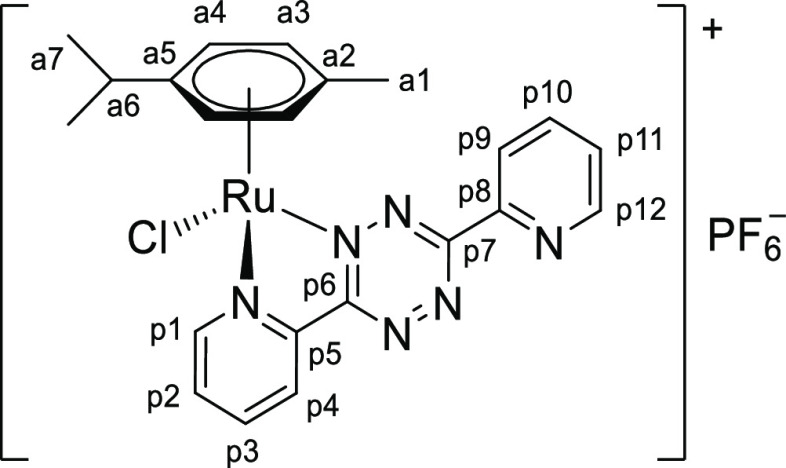
Structure of [**1c**]PF_6_ (Numbering Refers to C Atoms)^49^

Prepared from [RuCl_2_(η^6^-*p*-cymene)]_2_ (151 mg, 0.247 mmol) and 3,6-di(2-pyridyl)-1,2,4,5-tetrazine (117 mg, 0.49 mmol), according to the general procedure. Brown solid, yield: 196 mg. Alternatively obtained by the one-pot reaction of [RuCl_2_(η^6^-*p*-cymene)]_2_ (50 mg, 0.16 mmol), NaPF_6_ (29 mg, 0.16 mmol), and 3,6-di(2-pyridyl)-1,2,4,5-tetrazine (38 mg, 0.16 mmol) in acetone (8 mL); 2 h reaction time. The outcome of the reactions is highly sensitive to the reaction conditions (solvent, temperature, time). In each case, a blue solid (insoluble in CH_2_Cl_2_), containing a mixture of unidentified ruthenium arene complexes, accompanied the formation of [**1c**]^+^. Besides, [**1c**]PF_6_ was always isolated in admixture with variable amounts of 3,6-di(2-pyridyl)-1,2,4,5-tetrazine, which could not be separated. In this respect, the literature procedure to afford [**1c**]CF_3_SO_3_ (1.4 equiv of Ag(CF_3_SO_3_) and 3,6-di(2-pyridyl)-1,2,4,5-tetrazine in anhydrous CH_2_Cl_2_) was unsuccessful.^50^ It should be noted that the occurrence of side reactions (*e.g.*, formation of the bimetallic compound and incomplete binding of the free ligand) during the preparation of other monometallic complexes of 3,6-di(2-pyridyl)-1,2,4,5-tetrazine was reported (Chart 3).^51^

The title compound is soluble in CH_2_Cl_2_, acetone, and MeOH; moderately soluble in CHCl_3_; and insoluble in diethyl ether. MeOH solutions of [**1c**]PF_6_ are not stable, as suggested by the very rapid color changes. ^1^H NMR (400 MHz, acetone-*d*_6_): δ/ppm = 9.74 (d, ^3^*J*_HH_ = 5.1 Hz, 1H, p1), 9.06–9.00 (m, 2H, p4 + p12), 8.87 (d, ^3^*J*_HH_ = 7.8 Hz, 1H, p9), 8.56 (t, ^3^*J*_HH_ = 7.6 Hz, 1H, p3), 8.24 (t, ^3^*J*_HH_ = 7.3 Hz, 1H, p10), 8.14 (t,* ^3^*J*_HH_ = 6.8 Hz, p2), 7.83 (dd, ^3^*J*_HH_ = 6.9 Hz, ^4^*J*_HH_ = 4.8 Hz, 1H, p11), 6.46 (d, ^3^*J*_HH_ = 6.0 Hz, 1H, a4), 6.38 (d, ^3^*J*_HH_ = 6.1 Hz, 1H, a4′), 6.31 (d, ^3^*J*_HH_ = 6.2 Hz, 1H, a3), 6.13 (d, ^3^*J*_HH_ = 6.1 Hz, 1H, a3′), 3.04 (hept, ^3^*J*_HH_ = 6.9 Hz, 1H, a6), 2.28 (s, 3H, a1), 1.31 (app. t, ^3^*J*_HH_ = 7.4 Hz, 6H, a7 + a7′). *Overlapping with resonances of 3,6-di(2-pyridyl)-1,2,4,5-tetrazine. ^13^C{^1^H} NMR (126 MHz, acetone-*d*_6_): δ/ppm = 166.3 (p6), 164.6 (p7), 157.6 (p1), 152.2 (p12), 151.7 (p5), 149.7 (p8), 141.8 (p3), 138.9 (p10), 131.9 (p2), 128.6 (p11), 127.3 (p4), 126.7 (p9), 110.6 (a5), 105.8 (a2), 92.4 (a4), 90.4 (a4′), 90.0 (a3), 89.2 (a3′), 31.9 (a6), 22.6 (a7), 21.8 (a7′), 18.5 (a1).

##### 4.2.1.4 [RuCl{κ^2^*N*-3-(2-pyridyl)-1,2,4,5-tetrazine}(η^6^-C_6_Me_6_)]PF_6_, [**1d**]PF_6_

**Chart 4:**
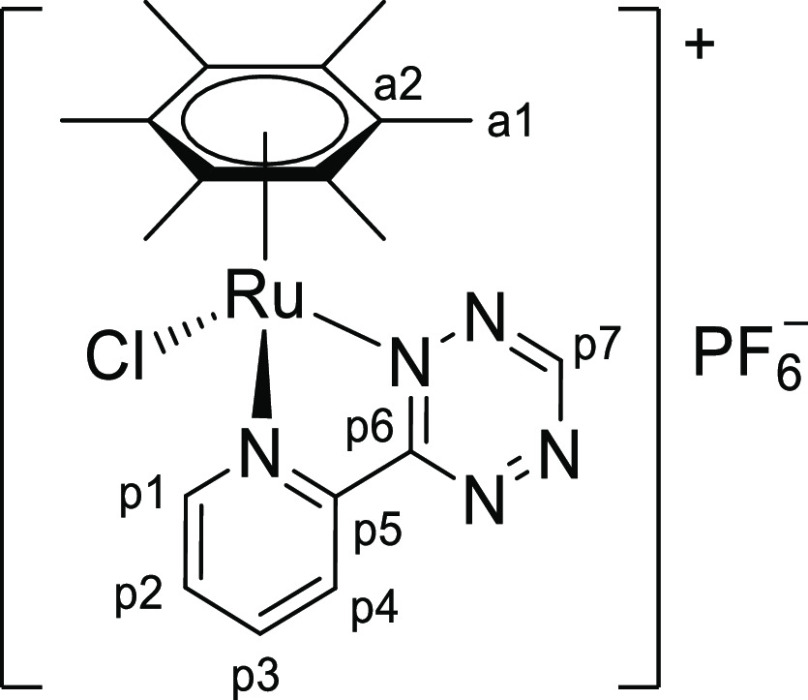
Structure of [**1d**]PF_6_ (Numbering Refers to C Atoms)^49^

Prepared from [RuCl_2_(η^6^-C_6_Me_6_)]_2_ (51 mg, 0.076 mmol) and 3-(2-pyridyl)-1,2,4,5-tetrazine (25 mg, 0.16 mmol). Dark red-purple microcrystalline solid, yield: 79 mg, 86%. Soluble in acetone, moderately soluble in CH_2_Cl_2_, poorly soluble in CHCl_3_, insoluble in Et_2_O, hexane. IR (solid state): ṽ/cm^–1^ = 1607w, 1504w, 1452w, 1419w, 1386w, 1346m, 1290w, 1260w, 1151w, 1073w, 1013w, 949m, 823s (PF_6_), 768m-sh, 744m, 688m, 671w. ^1^H NMR (400 MHz, acetone-*d*_6_): δ/ppm = 10.65 (s, 1H, p7), 9.24 (d, ^3^*J*_HH_ = 5.5 Hz, 1H, p1), 8.93 (d, ^3^*J*_HH_ = 7.8 Hz, 1H, p4), 8.49 (td, ^3^*J*_HH_ = 7.8 Hz, ^4^*J*_HH_ = 1.1 Hz, 1H, p3), 8.16 (ddd, ^3^*J*_HH_ = 7.4, 5.7 Hz, ^4^*J*_HH_ = 1.4 Hz, 1H, p2), 2.28 (s, 18H, a1). ^13^C{^1^H} NMR (101 MHz, acetone-*d*_6_): δ/ppm = 168.8 (p6), 159.3 (p7), 154.8 (p1), 150.0 (p5), 141.2 (p3), 132.1 (p2), 127.3 (p4), 102.0 (a2), 15.9 (a1) (Chart 4).

##### 4.2.1.5 [RuCl(κ^2^*N*-3-(2-pyridyl)-6-methyl-1,2,4,5-tetrazine)(η^6^-C_6_Me_6_)]PF_6_, [**1e**]PF_6_

**Chart 5:**
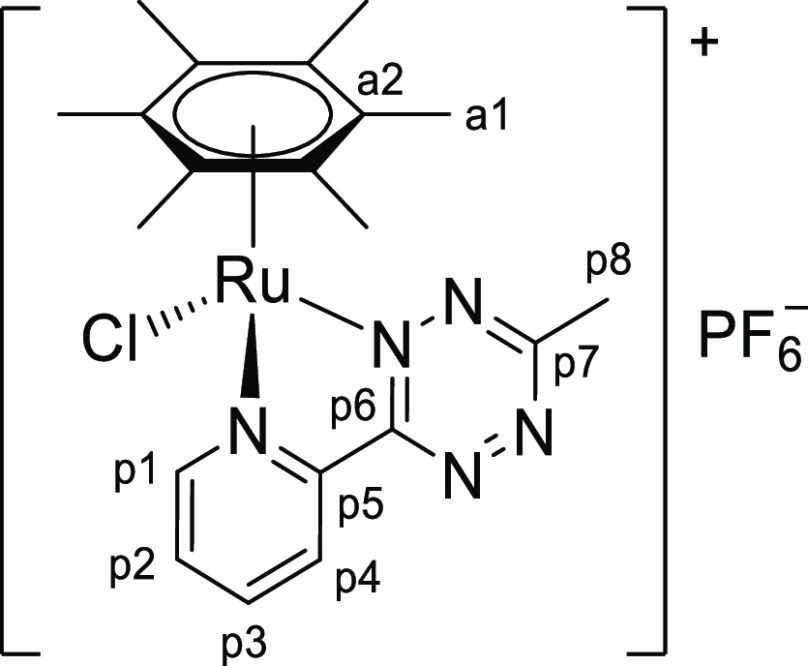
Structure of [**1e**]PF_6_ (Numbering Refers to C Atoms)^49^

Prepared from [RuCl_2_(η^6^-C_6_Me_6_)]_2_ (106 mg, 0.158 mmol) and 3-(2-pyridyl)-6-methyl-1,2,4,5-tetrazine (56 mg, 0.32 mmol). Dark purple/wine red solid, yield: 165 mg, 85%. Soluble in acetone, CH_2_Cl_2_, poorly soluble in CHCl_3_, insoluble in Et_2_O, hexane. IR (solid state): ṽ/cm^–1^ = 3092w, 2976w, 1438w, 1407m, 1371m, 1260w, 1162w, 1138w, 1072w, 1050w, 1021w, 936w, 878w, 835s (PF_6_), 808m-sh, 773m, 742w, 689w. ^1^H NMR (400 MHz, CDCl_3_): δ/ppm = 8.85–8.83 (m, 1H, p1), 8.83–8.81 (m, 1H, p4), 8.20 (td, ^3^*J*_HH_ = 7.7 Hz, ^4^*J*_HH_ = 1.0 Hz, p3), 7.87 (ddd, ^3^*J*_HH_ = = 7.3, 5.7 Hz, ^4^*J*_HH_ = 1.3 Hz, p2), 3.25 (s, 3H, p8), 2.22 (s, 18H, a1). ^1^H NMR (400 MHz, acetone-*d*_6_): δ/ppm = 9.21 (d, ^3^*J*_HH_ = 5.5 Hz, 1H, p1), 8.88 (d, ^3^*J*_HH_ = 7.8 Hz, 1H, p4), 8.47 (td, ^3^*J*_HH_ = 7.8 Hz, ^4^*J*_HH_ = 1.1 Hz, 1H, p3), 8.12 (ddd, ^3^*J*_HH_ = 7.4, 5.7 Hz, ^4^*J*_HH_ = 1.3 Hz, 1H, p2), 3.32 (s, 3H, p8), 2.27 (s, 18H, a1). ^13^C{^1^H} NMR (101 MHz, acetone-*d*_6_): δ/ppm = 170.1 (p7), 166.2 (p6), 154.6 (p1), 150.2 (p5), 141.2 (p3), 131.6 (p2), 126.7 (p4), 101.6 (a2), 21.6 (p8), 15.9 (a1) (Chart 5).

### 4.3 Synthesis and Characterization of Pyridyl–Triazine–Thione Complexes

#### 4.3.1 Procedure A

A solution of [**1a**–**c**]PF_6_ (50 mg, 0.09 mmol) in acetone (10 mL; 5 mg·mL^–1^ Ru) was treated with KSCN (1.0 equiv) and stirred at room temperature in the dark for 3 h. The red-brown reaction mixture rapidly turned to orange. Next, volatiles were removed under vacuum. The residue was suspended in CH_2_Cl_2_ (*ca*. 5 mL) and filtered over celite. The filtrate was taken to dryness under vacuum and triturated in Et_2_O. The suspension was filtered, and the resulting solid was washed with Et_2_O and hexane and dried under vacuum (40 °C). Performing the reactions at a higher concentration (*e.g.*, 13 mg·mL^–1^ Ru) led to small amounts of byproducts with ruthenium-coordinated thiocyanate, as detected by solid-state IR analyses.

#### 4.3.2 Procedure B

A solution of [**1a**–**c**]PF_6_ (31–50 mg) in CH_2_Cl_2_ (10–15 mL) was treated with [Bu_4_N][SCN] (1.0 equiv) and stirred at room temperature in the dark for 24 h. The final mixture was filtered over celite and the filtrate was taken to dryness under vacuum. The solid was washed with Et_2_O and dried under vacuum (40 °C). The ruthenium product was isolated as an inseparable mixture with the co-product [Bu_4_N]PF_6_.

#### 4.3.3 Procedure C

A dark purple/violet solution of [**1d**]PF_6_ and [**1e**]PF_6_ (15 mg, 0.025 mmol) in acetone-*d*_6_ (0.6 mL) was treated with a 0.27 M solution of KSCN in acetone-*d*_6_ (100 μL, 0.027 mmol) at room temperature, with immediate formation of a dark red solution (product not isolated).

##### 4.3.3.1 [RuCl{κ^2^*N*-6-(2-pyridyl)-5-thioxo-1,2,4-triazinide}(η^6^-*p*-cymene)], **2a**

**Chart 6:**
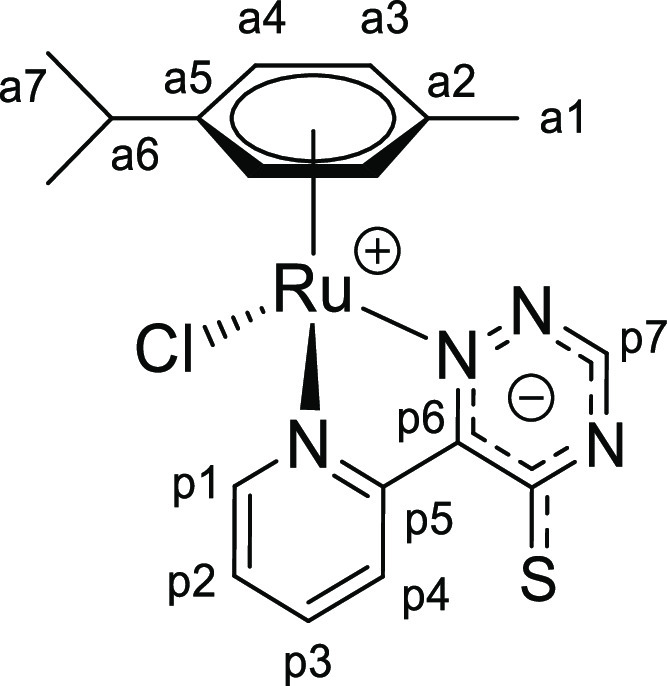
Structure of **2a** (Numbering Refers to C Atoms)^49^

Prepared from [**1a**]PF_6_ (50 mg, 0.087 mmol) and KSCN (9 mg, 0.09 mmol) according to procedure A. Red-orange solid, yield: 37 mg, 92%. Alternatively obtained in admixture with [Bu_4_N]PF_6_ from [**1a**]PF_6_ (30 mg, 0.06 mmol) and [Bu_4_N]SCN (15 mg, 0.06 mmol) following procedure B. Soluble in CH_2_Cl_2_, CHCl_3_, acetone, MeCN, poorly soluble in water, insoluble in diethyl ether and hexane. Anal. calcd for C_18_H_19_ClN_4_RuS: C, 47.00; H, 4.16; N, 12.18; S, 6.97. Found: C, 45.08; H, 4.21; N, 11.33; S, 5.48. IR (solid state): ṽ/cm^–1^ = 3036w-br, 2961w, 2924w, 2870w, 1699w, 1596w, 1492s, 1465m, 1413s, 1369s, 1321m, 1286m, 1241s, 1189s, 1148m, 1072m, 1032m, 993m, 837s, 792s, 757m, 742m, 725m. UV–vis (acetone, 2.2 × 10^–4^ M): λ/nm (ε/M^–1^·cm^–1^) = 405 (4.4 × 10^3^), 565br (1.5 × 10^3^). Λ_m_ (acetone, 3.4 × 10^–3^ M) = 19 S·cm^2^·mol^–1^. ^1^H NMR (500 MHz, acetone-*d*_6_): δ/ppm = 10.88 (d, ^3^*J*_HH_ = 8.3 Hz, 1H, p4), 9.55 (d, ^3^*J*_HH_ = 5.5 Hz, 1H, p1), 8.34 (s, 1H, p7), 8.13 (ddd, ^3^*J*_HH_ = 8.4, 7.5 Hz, ^4^*J*_HH_ = 1.6 Hz, 1H, p3), 7.64 (ddd, ^3^*J*_HH_ = 7.3, 5.7 Hz, ^4^*J*_HH_ = 1.3 Hz, 1H, p2), 6.05 (d, ^3^*J*_HH_ = 6.1 Hz, 1H, a4), 5.99 (d, ^3^*J*_HH_ = 6.2 Hz, 1H, a4′), 5.83 (d, ^3^*J*_HH_ = 6.1 Hz, 1H, a3), 5.78 (d, ^3^*J*_HH_ = 6.3 Hz, 1H, a3′), 2.99–2.84 (m,* a6), 2.24 (s, 3H, a1), 1.15 (d, ^3^*J*_HH_ = 6.9 Hz, 3H, a7 + a7′), 1.12 (d, ^3^*J*_HH_ = 6.9 Hz, 3H, C15′–H). *Over H_2_O resonance. ^13^C{^1^H} NMR (500 MHz, acetone-*d*_6_): δ/ppm = 186.6 (CS), 156.7 (p5), 156.5 (p6), 156.1 (p1), 153.2 (p7), 138.5 (p3), 129.7 (p4), 126.9 (p2), 106.1 (a5), 103.9 (a2), 91.0 (a4), 88.8 (a4′), 87.8 (a3), 87.4 (a3′), 31.7 (a6), 22.4 (a7), 22.0 (a7′), 18.6 (a1). ESI-MS (MeCN): *m*/*z* = 461.0134 Da; calculated base peak for [**2a** + H]^+^: 461.0168 Da (Chart 6).

##### 4.3.3.2 [RuCl{κ^2^*N*-3-methyl-6-(2-pyridyl)-5-thioxo-1,2,4-triazinide}(η^6^-*p*-cymene)], **2b**

**Chart 7:**
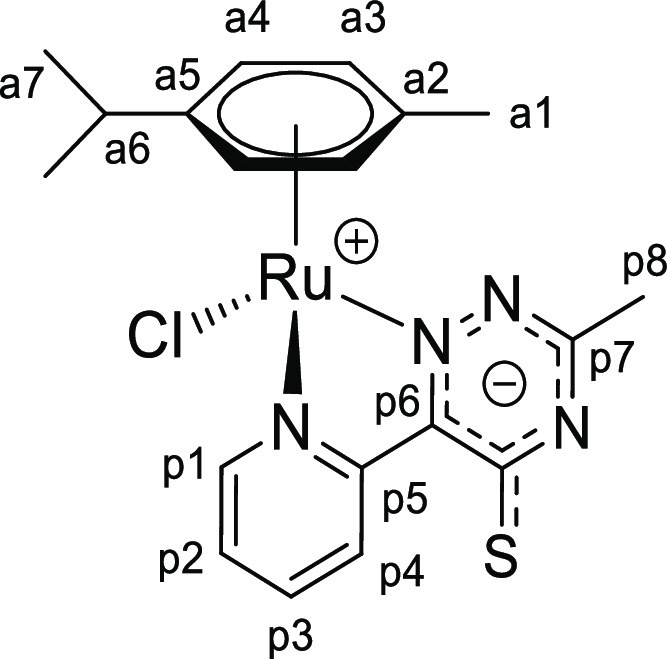
Structure of **2b** (Numbering Refers to C Atoms)^49^

Prepared from [**1b**]PF_6_ (50 mg, 0.085 mmol) and KSCN (8 mg, 0.09 mmol) according to procedure A. Red-orange solid, yield: 38 mg, 92%. Alternatively obtained in admixture with [Bu_4_N]PF_6_ from [**1b**]PF_6_ (50 mg, 0.08 mmol) and [Bu_4_N]SCN (24 mg, 0.08 mmol) following procedure B. Soluble in CH_2_Cl_2_, CHCl_3_, acetone, MeCN, poorly soluble in water, insoluble in diethyl ether and hexane. Anal. calcd for C_19_H_21_ClN_4_RuS: C, 48.15; H, 4.47; N, 11.82; S, 6.77. Found: C, 45.63; H, 4.42; N, 11.17; S, 6.17. IR (solid state): ṽ/cm^–1^ = 3062w, 3034w, 2961w, 2924w, 2870w, 1702w, 1593w, 1470s, 1464s, 1439s, 1408s, 1318s, 1277m, 1256s, 1226m, 1194s, 1157m, 1090s, 1057m, 1032m, 1003m, 938s, 867w, 841m, 794s, 772s, 760s, 742w, 720m, 658m. UV–vis (acetone, 2.0 × 10^–4^ M): λ/nm (ε/M^–1^·cm^–1^) = 404 (3.5 × 10^3^). Λ_m_ (acetone, 3.4 × 10^–3^ M) = 9 S·cm^2^·mol^–1^. ^1^H NMR (500 MHz, acetone-*d*_6_): δ/ppm = 10.89 (d, ^3^*J*_HH_ = 8.2 Hz, 1H, p4), 9.52 (dd, ^3^*J*_HH_ = 5.6 Hz, ^4^*J*_HH_ = 1.0 Hz, 1H, p1), 8.10 (ddd, ^3^*J*_HH_ = 8.4, 7.6 Hz, ^4^*J*_HH_ = 1.6 Hz, 1H, p3), 7.59 (ddd, ^3^*J*_HH_ = 7.3, 5.7 Hz, ^4^*J*_HH_ = 1.5 Hz, 1H, p2), 6.03 (d, ^3^*J*_HH_ = 6.1, 1H, a4), 5.99 (d, ^3^*J*_HH_ = 6.3, 1.2 Hz, 1H, a4′), 5.81 (d, ^3^*J*_HH_ = 6.1 Hz, 1H, a3), 5.77 (d, ^3^*J*_HH_ = 6.3 Hz, 1H, a3′), 2.89–2.79 (m,* 1H, a6), 2.42 (s, 3H, p8), 2.23 (s, 3H, a1), 1.16 (d, ^3^*J*_HH_ = 7.0 Hz, 3H, a7), 1.13 (d, ^3^*J*_HH_ = 6.9 Hz, 3H, a7′); no changes were observed in the ^1^H NMR spectrum after 48 h at room temperature. *Over H_2_O resonance. ^13^C{^1^H} NMR (126 MHz, acetone-*d*_6_): δ/ppm = 186.3 (CS), 162.1 (p7), 156.8 (p5), 155.9 (p1), 153.9 (p6), 138.4 (p3), 129.6 (p4), 126.5 (p2), 105.8 (a5), 103.6 (a2), 91.3 (a4), 88.8 (a4′), 87.7 (a3), 87.3 (a3′), 31.7 (a6), 23.0 (p8), 22.5 (a7), 21.9 (a7′), 18.6 (a1). ESI-MS (MeCN): *m*/*z* = 475.0289 Da; calculated base peak for [**2b** + H]^+^: 475.0324 Da. ^1^H NMR (400 MHz, CD_3_CN): δ/ppm = 10.76 (d, *J* = 8.3 Hz, 1H, p4), 9.32 (d, *J* = 5.2 Hz, 1H, p1), 8.09–8.02 (m, 1H, p3), 7.59–7.51 (m, 1H, p2); 5.82 (app. t, *J* = 6.5 Hz, 2H), 5.63 (d, *J* = 6.2 Hz, 1H), 5.58 (d, *J* = 6.1 Hz, 1H) (a3 + a3′ + a4 + a4′); 2.74 (hept, *J* = 6.9 Hz, 1H, a6), 2.50 (s, 3H, p8), 2.16 (s, 3H, a1), 1.09 (d, *J* = 6.9 Hz, 3H), 1.05 (d, *J* = 6.9 Hz, 3H) (a7 + a7′); 1% *p*-cymene was observed after 24 h at room temperature. Partial decomposition to a dark green-brown solid was observed after 8 months under N_2_ at room temperature; compound **2b** was purified by silica chromatography (eluent: CH_2_Cl_2_/acetone 2:1 v/v) and stored under N_2_ at −20 °C (Chart 7).

##### 4.3.3.3 [RuCl{κ^2^*N*-3,6-di-(2-pyridyl)-5-thioxo-1,2,4-triazinide}(η^6^-*p*-cymene)], **2c**

**Chart 8:**
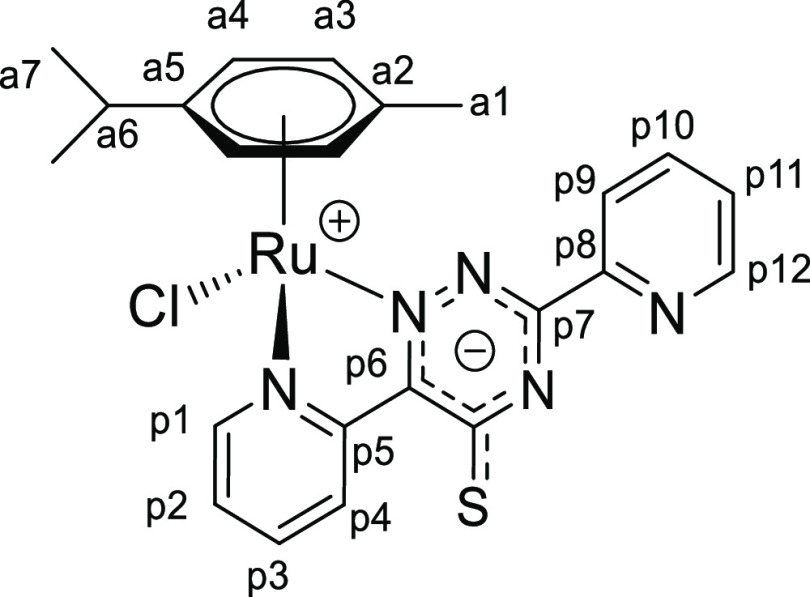
Structure of **2c** (Numbering Refers to C Atoms)^49^

Prepared from [**1c**]PF_6_ (*ca*. 0.15 mmol) and [Bu_4_N]SCN (47 mg, 0.15 mmol) following procedure B. The final dark red solution was charged on a silica column (*d* 2.3 cm, *h* 3 cm). A red band was eluted with tetrahydrofuran (THF) and taken to dryness under vacuum without external heating. The resulting red solid was washed with an Et_2_O/CHCl_3_ 15:1 v/v mixture and dried under vacuum. Yield: 111 mg (in admixture with [Bu_4_N]PF_6_). Compound **2c** was also obtained from [**1c**]PF_6_ and KSCN, according to procedure A, with comparatively higher amounts of byproducts. Soluble in CH_2_Cl_2_, acetone, insoluble in Et_2_O. ^1^H NMR (400 MHz, acetone-*d*_6_): δ/ppm = 10.92 (d, ^3^*J*_HH_ = 8.2 Hz, 1H, p4), 9.64 (dd, ^3^*J*_HH_ = 5.6 Hz, ^4^*J*_HH_ = 0.9 Hz, 1H, p1), 8.81 (d, ^3^*J*_HH_ = 4.6 Hz, 1H, p12), 8.50 (d, ^3^*J*_HH_ = 7.9 Hz, 1H, p9), 8.14 (td, ^3^*J*_HH_ = 8.3 Hz, ^4^*J*_HH_ = 1.4 Hz, 1H p3), 7.99 (td, ^3^*J*_HH_ = 7.8 Hz, ^4^*J*_HH_ = 1.7 Hz, 1H, p10), 7.66 (ddd, ^3^*J*_HH_ = 7.3, 5.7 Hz, ^4^*J*_HH_ = 1.4 Hz, p2), 7.55 (ddd, ^3^*J*_HH_ = 7.5, 4.7 Hz, ^4^*J*_HH_ = 0.9 Hz, 1H, p11), 6.16 (d, ^3^*J*_HH_ = 6.2 Hz, 1H, a4), 6.13 (d, ^3^*J*_HH_ = 6.3 Hz, 1H, a4′), 5.95 (d, ^3^*J*_HH_ = 6.3 Hz, 1H, a3), 5.90 (d, ^3^*J*_HH_ = 6.3 Hz, a3′), 2.95 (hept, ^3^*J*_HH_ = 6.9 Hz, 1H, a6), 2.27 (s, 3H, a1), 1.17 (app. t, ^3^*J*_HH_ = 7.3 Hz, 6H, a7 + a7′). ^13^C{^1^H} NMR (100 MHz, acetone-*d*_6_): δ/ppm = 186.4 (CS), 157.9 (p7), 155.4 (p1 + p5), 155.3 (p6), 154.3 (p8), 150.7 (p12), 138.5 (p3), 137.6 (p10), 129.6 (p4), 126.9 (p11), 126.1 (p2), 125.0 (p9), 106.4 (a5), 103.7 (a2), 91.5 (a4), 88.9 (a4′), 88.1 (a3), 87.5 (a3′), 31.7 (a6), 22.6 (a7), 22.0 (a7′), 18.6 (a1). ESI-MS (MeCN): *m*/*z* = 538.0398 Da; calculated base peak for [**2c** + H]^+^ C_23_H_23_RuN_4_SCl^+^: 538.0433 Da (Chart 8).

##### 4.3.3.4 [RuCl{κ^2^*N*-6-(2-pyridyl)-5-thioxo-1,2,4-triazinide}(η^6^-C_6_Me_6_)], **2d**

**Chart 9:**
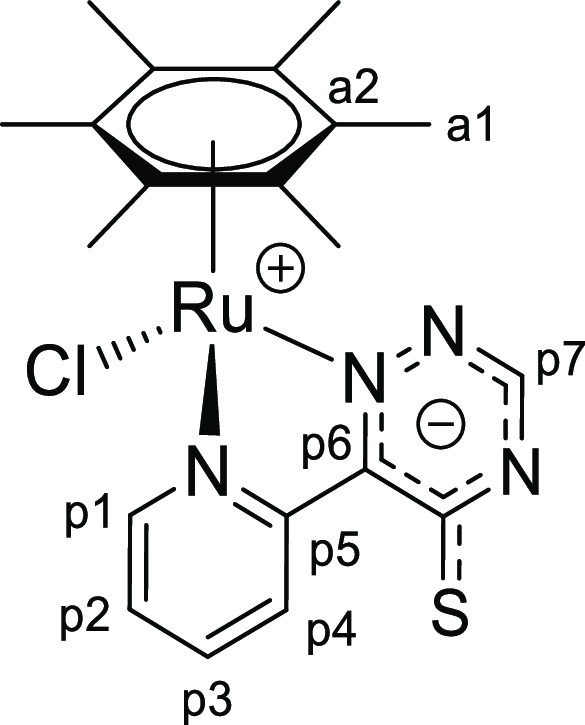
Structure of **2d** (Numbering Refers to C Atoms)^49^

Prepared from [**1d**]PF_6_ according to procedure C. ^1^H NMR (400 MHz, acetone-*d*_6_): δ/ppm = 10.87 (d, ^3^*J*_HH_ = 8.2 Hz, 1H, p4), 9.02 (dd, ^3^*J*_HH_ = 5.6 Hz, 1H, ^4^*J*_HH_ = 0.8 Hz, p1), 8.40 (s, 1H, p7), 8.12–8.05 (m, 1H, p3), 7.70 (ddd, ^3^*J*_HH_ = 7.3, 5.7 Hz, ^4^*J*_HH_ = 1.4 Hz, 1H, p2), 2.14 (s, 18H, a1). ^13^C{^1^H} NMR (101 MHz, acetone-*d*_6_): δ/ppm = 186.0 (CS), 156.8 (p6), 156.1 (p5), 153.9 (p1), 153.1 (p7), 138.0 (p3), 128.8 (p4), 127.0 (p2), 98.5 (a2), 15.5 (a1) (Chart 9).

##### 4.3.3.5 [RuCl{κ^2^*N*-3-methyl-6-(2-pyridyl)-5-thioxo-1,2,4-triazinide}(η^6^-C_6_Me_6_)], **2e**

**Chart 10:**
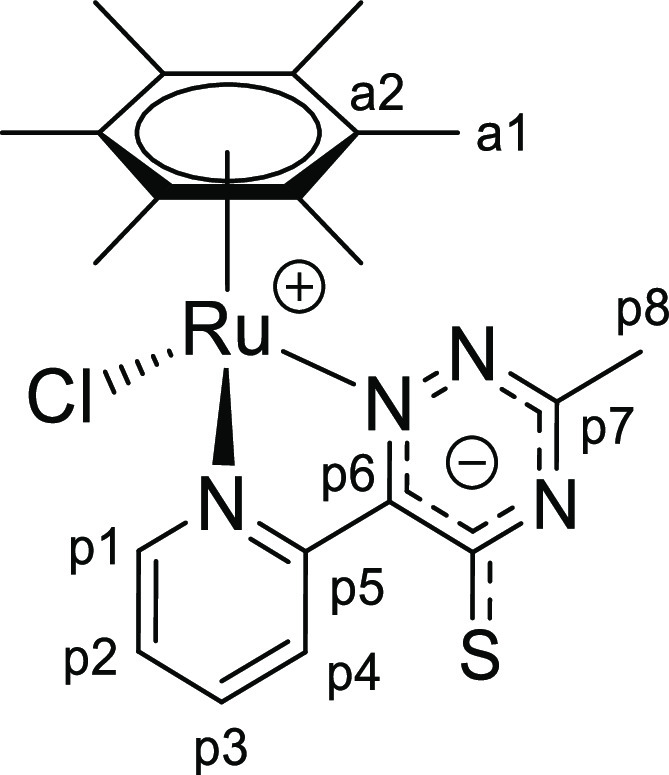
Structure of **2e** (Numbering Refers to C Atoms)^49^

Prepared from [**1e**]PF_6_ according to procedure C. ^1^H NMR (500 MHz, acetone-*d*_6_): δ/ppm = 10.87 (d, ^3^*J*_HH_ = 8.1 Hz, 1H, p4), 9.00 (dd, ^3^*J*_HH_ = 5.7 Hz, ^4^*J*_HH_ 1.0 Hz, 1H, p1), 8.10–8.04 (m, 1H, p3), 7.66 (ddd, ^3^*J*_HH_ = 7.3, 5.7 Hz, ^4^*J*_HH_ = 1.5 Hz, 1H, p2), 2.48 (s, 3H, p8), 2.14 (s, 18H, a1). ^13^C{^1^H} NMR (125 MHz, acetone-*d*_6_): δ/ppm = 185.6 (CS), 161.8 (p7), 156.3 (p5), 154.0 (p6), 153.7 (p1), 137.9 (p3), 128.8 (p4), 126.6 (p2), 98.4 (a2), 22.4 (p8), 15.5 (a1) (Chart 10).

##### 4.3.3.6 [RuCl{κ^2^*N*-3-methyl-6-(2-pyridyl)-1,2,4-triazine-5-thione}(η^6^-*p*-cymene)]X, [**2bH**]X (X = PF_6_, *p*-CH_3_C_6_H_4_SO_3_, CF_3_SO_3_)

**Chart 11:**
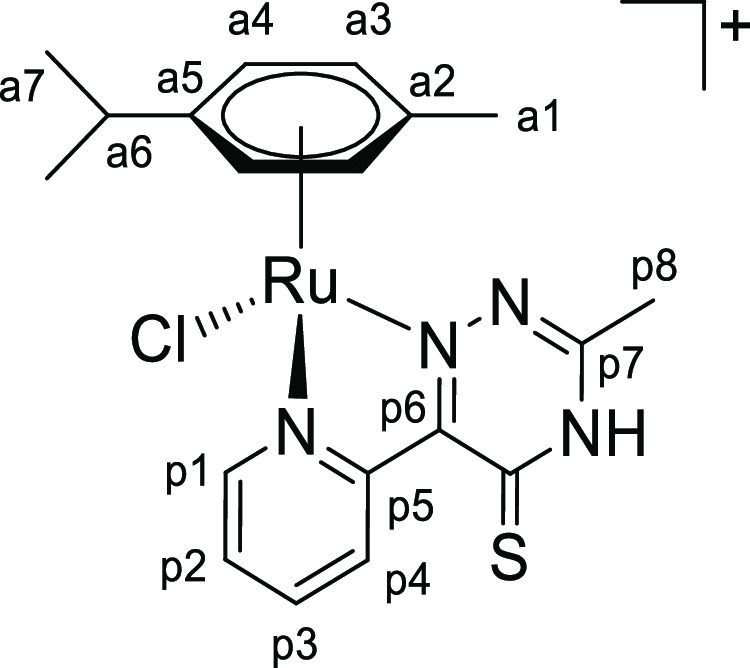
Structure of [**2bH**]^+^ (Numbering Refers to C Atoms)^49^

**[2bH]PF**_**6**_. A dark orange suspension of **2b** and KPF_6_, freshly prepared from [**1b**]PF_6_ (56 mg, 0.094 mmol) and KSCN (9 mg, 0.093 mmol), in MeCN (5 mL) was treated with aqueous HCl (1.0 M, 94 μL, 0.094 mmol) and stirred in the dark at room temperature. After 2 h, the resulting dark red mixture was taken to dryness under vacuum. The residue was suspended in CH_2_Cl_2_ and filtered over celite. The filtrate was taken to dryness under vacuum, affording a red-brown solid. The solid was washed with Et_2_O, hexane, and dried under vacuum (40 °C). Yield: 37 mg, 64%. Soluble in CH_2_Cl_2_, acetone, insoluble in diethyl ether. Anal. calcd for C_19_H_22_ClF_6_N_4_PRuS: C, 36.81; H, 3.58; N, 9.04; S, 5.17. Found: C, 39.05; H, 3.80; N, 9.22; S, 5.18. Λ_m_ (acetone, 1.4 × 10^–3^ M) = 135 S·cm^2^·mol^–1^. UV–vis (acetone, 2.0 × 10^–4^ M): λ/nm (ε/M^–1^·cm^–1^) = 403 (4.0 × 10^3^), 435 (4.7 × 10^3^), 455 (4.6 × 10^3^). IR (solid state): ṽ/cm^–1^ = 3089w, 2963v, 2928w, 2870w, 1561m, 1520m, 1466m, 1420m, 1391m, 1322w, 1283w, 1230m, 1184m, 1132m, 1104m, 1058w, 1034w, 999w, 958m, 876w-sh, 831s (PF_6_), 786s-sh, 770s-sh, 751s-sh, 723m-sh, 657w. ^1^H NMR (500 MHz, CDCl_3_): δ/ppm = 10.83 (d, ^3^*J*_HH_ = 8.3 Hz, 1H, p4), 9.11 (d, ^3^*J*_HH_ = 5.2 Hz, 1H, p1), 7.97 (t, ^3^*J*_HH_ = 7.5 Hz, 1H, p3), 7.44 (t, ^3^*J*_HH_ = 6.2 Hz, 1H, p2), 5.71 (d, ^3^*J*_HH_ = 6.2 Hz, 1H, a4), 5.67 (d, ^3^*J*_HH_ = 6.1 Hz, 1H, a4′), 5.48 (app.t, ^3^*J*_HH_ = 6.8 Hz, 2H, a3 + a3′), 2.79 (hept, ^3^*J*_HH_ = 6.9 Hz, 1H, a6), 2.58 (s, 3H, p8), 2.24 (s, 3H, a1), 1.15 (app.t, ^3^*J*_HH_ = 6.6 Hz, 6H, a7 + a7′). ^1^H NMR (500 MHz, acetone-*d*_6_): δ/ppm = 10.28 (d, ^3^*J*_HH_ = 8.0 Hz, 1H, p4), 9.66 (d, ^3^*J*_HH_ = 4.8 Hz, 1H, p1), 8.27 (t, ^3^*J*_HH_ = 7.7 Hz, 1H, p3), 7.82 (t, ^3^*J*_HH_ = 6.2 Hz, 1H, p2), 6.21 (d, ^3^*J*_HH_ = 5.7 Hz, 1H, a4), 6.11 (d, ^3^*J*_HH_ = 6.0 Hz, 1H, a4′), 5.98 (d, ^3^*J*_HH_ = 5.8 Hz, 1H, a3), 5.96 (d, ^3^*J*_HH_ = 6.3 Hz, 1H, a3′), 5.40 (br, 1H, NH), 2.93 (hept, ^3^*J*_HH_ = 6.8 Hz, 1H, a6), 2.72 (s, 3H, p8), 2.26 (s, 3H, a1), 1.21–1.19 (m, 6H, a7 + a7′). ^13^C{^1^H} NMR (126 MHz, acetone-*d*_6_): δ/ppm = 176.7 (CS), 157.9 (p7), 157.0 (p1), 156.8 (p6), 154.1 (p5), 139.4 (p3), 131.3 (p4), 128.5 (p2), 108.3 (a5), 104.8 (a2), 91.7 (a4), 90.3 (a4′), 89.1 (a3), 88.9 (a3′), 31.8 (a6), 22.6 (a7), 21.9 (a7′), 20.0 (p8), 18.6 (a1). The procedure was repeated with 2 equiv of HCl, at 50 °C for 3 h. The ^1^H NMR spectrum of the resulting red solid in acetone-*d*_6_ showed only resonances due to [**2bH**]PF_6_ (Chart 11).

**[2bH](*****p*****-CH**_**3**_**C**_**6**_**H**_**4**_**SO**_**3**_**)**. A solution of **2b** (32 mg, 0.068 mmol) in CH_2_Cl_2_ (5 mL) was treated with 9.5 × 10^–2^ M *p*-toluenesulfonic acid (TsOH) in CHCl_3_ (0.94 mL, 0.089 mmol) and stirred at room temperature in the dark for 5 h. The final red solution was filtered over celite, and the filtrate was taken to dryness under vacuum. The residue was triturated in a CH_2_Cl_2_/Et_2_O 1:3 v/v solution (Et_2_O washings are not effective in removing excess TsOH). The suspension was stirred overnight and then filtered. The resulting red-brown solid was washed with Et_2_O and hexane and dried under vacuum (40 °C). Yield: 27 mg, 61%. ^1^H NMR (400 MHz, acetone-*d*_6_): δ/ppm = 10.13 (d, *J* = 8.3 Hz, 1H), 9.72 (d, *J* = 5.3 Hz, 1H), 8.27 (t, *J* = 7.7 Hz, 1H), 7.84 (t, *J* = 6.2 Hz, 1H), 7.75 (d, *J* = 7.9 Hz, 2H), 7.19 (d, *J* = 7.6 Hz, 2H), 6.26 (d, *J* = 6.1 Hz, 1H), 6.18 (d, *J* = 6.2 Hz, 1H), 6.01 (app. t, *J* = 6.7 Hz, 2H), 3.8 (br, NH), 2.99–2.89 (m, 1H), 2.79 (s, 3H), 2.34 (s, 3H), 2.24 (s, 3H), 1.20 (d, *J* = 6.9 Hz, 6H).

**[2bH]CF**_**3**_**SO**_**3**_. A solution of **2b** (11 mg, 0.023 mmol) in CH_2_Cl_2_ (5 mL) was treated with 0.23 M trifluoromethanesulfonic acid (CF_3_SO_3_H) in CH_2_Cl_2_ (0.10 mL, 0.023 mmol) and stirred at room temperature in the dark for 4 h. The resulting red solution was filtered over celite and dried under vacuum, affording a dark red solid. Yield: 14 mg, 97%. ^1^H NMR (400 MHz, acetone-*d*_6_): δ/ppm = 10.14 (d, *J* = 8.2 Hz, 1H), 9.70 (d, *J* = 5.2 Hz, 1H), 8.31 (t, *J* = 7.7 Hz, 1H), 7.87 (t, *J* = 6.2 Hz, 1H), 6.26 (d, *J* = 6.1 Hz, 1H), 6.18 (d, *J* = 6.2 Hz, 1H), 6.03 (d, *J* = 6.4 Hz, 1H), 6.01 (d, *J* = 6.3 Hz, 1H), 3.03–2.89 (m, 1H), 2.77 (s, 3H), 2.27 (s, 3H), 1.21 (d, *J* = 6.9 Hz, 6H).

**Titration/deprotonation of [2bH]**^**+**^. A solution of [**2bH**]PF_6_ (2.0 × 10^–4^ M) in acetone was titrated (10 μL additions, up to 160 μL) with an acetone solution of Et_3_N (9.3 × 10^–2^ M), under stirring at room temperature. After each addition, the solution was analyzed by UV–vis (Figure S46). No further changes to the UV–vis spectrum were noticed after the addition of 1.0 equiv of Et_3_N (80 μL). The final solution was taken to dryness under vacuum, and the residue was analyzed by ^1^H NMR, confirming the quantitative formation of **2b**. Similarly, a solution of [**2bH**]PF_6_ in CH_2_Cl_2_ was extracted with H_2_O (3×) and then dried under vacuum. The organic residue was analyzed by ^1^H NMR, indicating the quantitative formation of **2b**. In another experiment, a solution of [**2bH**]PF_6_ in CH_2_Cl_2_ was moved on top of a silica column. An orange band was eluted with an acetone/CH_2_Cl_2_ 1:1 v/v solution containing Et_3_N (1%). Volatiles were removed under vacuum, and the residue was identified as **2b** by ^1^H NMR.

### 4.4 Synthesis and Characterization of Pyridyl–Triazine–Selone Complexes

#### 4.4.1 General Procedure

A red/violet solution of the [**1b**]PF_6_, [**1d**]PF_6_, and [**1e**]PF_6_ (37–80 mg) in anhydrous CH_2_Cl_2_ (10–15 mL) under N_2_ was treated with [Bu_4_N][SeCN] (1.0 equiv) and stirred at room temperature under protection from the light. After 3 h, the final dark red-brown mixture was filtered over celite and the filtrate was taken to dryness under vacuum. The solid was washed with acetone/Et_2_O 1:6 v/v and dried under vacuum (40 °C). The ruthenium product was isolated as an inseparable mixture with the co-product [Bu_4_N]PF_6_.

##### 4.4.1.1 [RuCl{κ^2^*N*-3-methyl-6-(2-pyridyl)-5-selenoxo-1,2,4-triazinide}(η^6^-*p*-cymene)], **3b**

**Chart 12:**
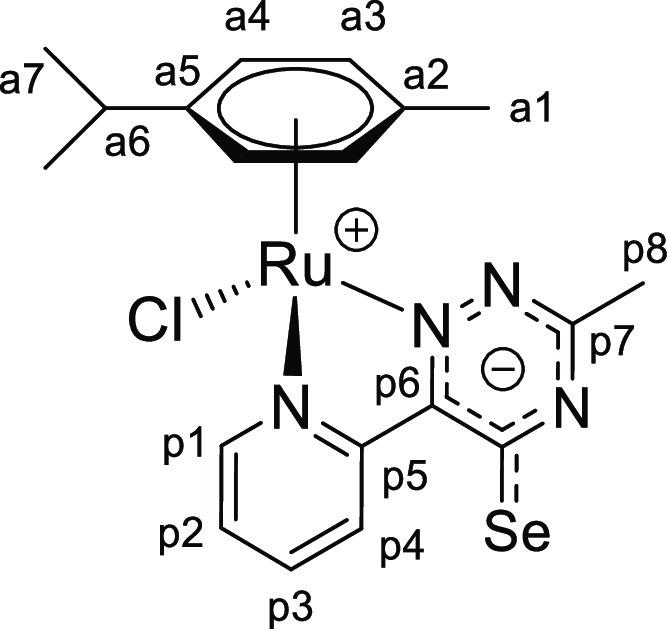
Structure of **3b** (Numbering Refers to C Atoms)^49^

Prepared using [**1b**]PF_6_ (37 mg, 0.063 mmol) and [Bu_4_N]SeCN (22 mg, 0.063 mmol). Dark red solid. The solid product contains [Bu_4_N]PF_6_, *p*-cymene, and minor amounts of unidentified ruthenium *p*-cymene species. ^1^H NMR (400 MHz, acetone-*d*_6_): δ/ppm = 11.26 (d, ^3^*J*_HH_ = 8.3 Hz, 1H, p4), 9.57 (d, ^3^*J*_HH_ = 5.2 Hz, p1), 8.16–8.07 (m, 1H, p3), 7.65 (ddd, ^3^*J*_HH_ = 7.2, 5.7 Hz, ^4^*J*_HH_ = 1.3 Hz, 1H, p2), 6.06 (d, ^3^*J*_HH_ = 6.1 Hz, 1H, a4), 6.00 (d, ^3^*J*_HH_ = 6.1 Hz, 1H, a4′), 5.84 (d, ^3^*J*_HH_ = 6.1 Hz, 1H, a3), 5.79 (d, ^3^*J*_HH_ = 6.3 Hz, 1H, a3′), 2.46 (s, 3H, p8), 2.24 (s, 3H, a2), 1.20 (d, ^3^*J*_HH_ = 6.9 Hz, 3H, a7), 1.16 (d, ^3^*J*_HH_ = 6.9 Hz, 3H, a7′) (Chart 12).

##### 4.4.1.2 [RuCl{κ^2^*N*-6-(2-pyridyl)-5-selenoxo-1,2,4-triazinide}(η^6^-C_6_Me_6_)], **3d**

**Chart 13:**
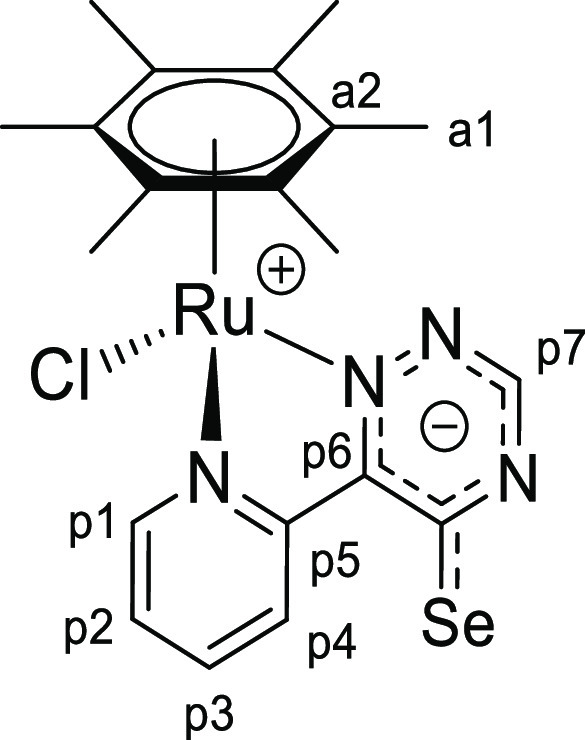
Structure of **3d** (Numbering Refers to C Atoms)^49^

Prepared using [**1d**]PF_6_ (38 mg, 0.063 mmol) and [Bu_4_N]SeCN (22 mg, 0.063 mmol). Red-brown solid, yield: 31 mg (in admixture with [Bu_4_N]PF_6_). Soluble in CH_2_Cl_2_, CHCl_3_, less soluble in acetone, insoluble in Et_2_O. IR (solid state): no bands ascribable to coordinated selenocyanate were detected (2000–2150 cm^–1^ range), see the SI. ^1^H NMR (500 MHz, CDCl_3_): δ/ppm = 11.15 (d, ^3^*J*_HH_ = 8.2 Hz, 1H, p4), 8.75 (d, ^3^*J*_HH_ = 5.7 Hz, 1H, p1), 8.42 (s, 1H, p7), 7.98–7.89 (m, 1H, p3), 7.56 (ddd, ^3^*J*_HH_ = 7.3, 5.7 Hz, ^4^*J*_HH_ = 1.5 Hz, 1H, p2), 2.06 (s, 18H, a1). ^13^C{^1^H} NMR (125 MHz, CDCl_3_): δ/ppm = 183.6 (CSe), 159.0 (p6), 155.2 (p5), 152.1 (p1), 151.9 (p7), 137.4 (p3), 128.7 (p4), 126.6 (p2), 97.6 (a2), 15.5 (a1). ^77^Se NMR (76 MHz, CDCl_3_): δ/ppm = 628.1. ESI-MS (MeCN): *m*/*z* = 536.9889 Da; calculated base peak for [**3d** + H]^+^: 536.9985 Da (Chart 13).

##### 4.4.1.3 [RuCl{κ^2^*N*-3-methyl-6-(2-pyridyl)-5-selenoxo-1,2,4-triazinide}(η^6^-C_6_Me_6_)], **3e**

**Chart 14:**
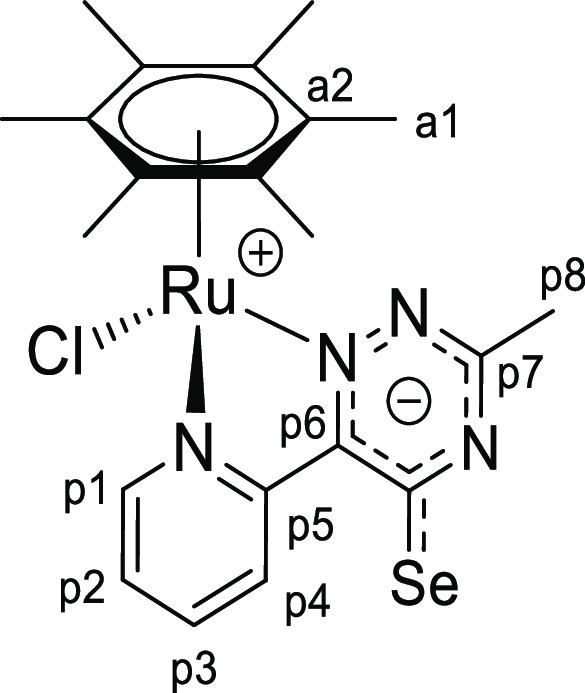
Structure of **3e** (Numbering Refers to C Atoms)^49^

Prepared using [**1e**]PF_6_ (80 mg, 0.130 mmol) and [Bu_4_N]SeCN (45 mg, 0.129 mmol). Red-brown solid. Yield: 72 mg (in admixture with [Bu_4_N]PF_6_). Soluble in CH_2_Cl_2_, CHCl_3_, less soluble in acetone ad insoluble in Et_2_O. IR (solid state): no bands ascribable to coordinated selenocyanate were detected (2000–2150 cm^–1^ range), see the SI. ^1^H NMR (400 MHz, CDCl_3_): δ/ppm = 11.16 (d, ^3^*J*_HH_ = 8.0 Hz, 1H, p4), 8.70 (d, ^3^*J*_HH_ = 5.2 Hz, 1H, p1), 7.96–7.91 (m, 1H, p3), 7.49 (ddd, ^3^*J*_HH_ = 7.3, 5.8 Hz, ^4^*J*_HH_ = 1.4 Hz, 1H, p2), 2.61 (s, 3H, p8), 2.08 (s, 18H, a1). ^13^C{^1^H} NMR (100 MHz, CDCl_3_): δ/ppm = 183.0 (CSe), 161.6 (p7), 156.4 (p6), 155.7 (p5), 151.7 (p1), 137.4 (p3), 128.9 (p4), 125.9 (p2), 97.4 (a2), 22.8 (p8), 15.5 (a1). ^77^Se NMR (76 MHz, CDCl_3_): δ/ppm = 580.3. ESI-MS (MeCN): *m*/*z* = 551.0050 Da, calculated base peak for [**3e** + H]^+^: 551.0141 Da. A minor set of ^1^H NMR signals, resembling that of **3e**, was present both in the reaction crude and in the final product. ^1^H NMR (400 MHz, CDCl_3_): δ/ppm = 11.25 (d, *J* = 9.1 Hz, 1H), 8.60 (d, *J* = 5.7 Hz, 1H), 8.01–7.96 (m, 1H), 7.57 (ddd, *J* = 7.4, 5.8, 1.4 Hz, 1H), 2.53 (s, 3H), 2.12 (s, 18H). During a purification attempt, the reaction crude was moved on top of a silica column (*h* 3 cm, *d* 2.3 cm). A red band was eluted with CH_2_Cl_2_/acetone 4:1 v/v and dried under vacuum. The resulting dark red solid consisted of **3e** and [RuCl{κ^2^*N*-3-(2-pyridyl)-6-methyl-1,2,4-triazine-5-thione}(η^6^-C_6_Me_6_)], **3e**^***O***^, derived from hydrolysis of the C=Se bond (formal Se/O exchange in **3e**). ESI-MS (MeCN): *m*/*z* = 487.0840 Da, calculated base peak for [**3e**^***O***^ + H]^+^: 487.0866 Da (Chart 14).

##### Reversible Protonation of 3e

: A dark red suspension of **3e** and [Bu_4_N]PF_6_ in MeCN (2 mL), freshly prepared from [**1e**]PF_6_ (15 mg, 0.024 mmol) and [Bu_4_N]SeCN (7.3 mg, 0.024 mmol), was treated with aqueous HCl (1.0 M, 25 μL, 0.025 mmol) and stirred at room temperature for 1 h. Volatiles were removed under vacuum, and the residue was analyzed by ^1^H NMR, showing the quantitative conversion of **3e** and a new set of signals related to its protonated derivative, [**3eH**]^+^. ^1^H NMR (500 MHz, acetone-*d*_6_): δ/ppm = 10.73 (d, ^3^*J*_HH_ = 4.5 Hz, 1H, p4), 9.08 (d, ^3^*J*_HH_ = 5.8 Hz, 1H, p1), 8.16 (t, ^3^*J*_HH_ = 8.2 Hz, 1H, p3), 7.81 (t, ^3^*J*_HH_ = 6.5 Hz, 1H, p2), 2.80 (s, 3H, p8), 2.20 (s, 18H, a1). Next, excess Et_3_N (5 μL, 0.036 mmol) was added to the NMR tube, leading to the re-formation of **3e** (^1^H NMR).

### 4.5 Tetrazine/Cyanate Reactivity

#### 4.5.1 Reaction of [**1a**]PF_6_ with [Bu_4_N][OCN]

Compound [**1a**]PF_6_ (15 mg, 0.026 mmol) and [Bu_4_N][OCN] (7 mg, 0.026 mmol) were dissolved in acetone-*d*_6_ (0.6 mL) and transferred into an NMR tube. The ^1^H NMR spectrum was recorded within 30 min from mixing, showing quantitative conversion. Two major sets of signals for ruthenium *p*-cymene complexes were identified, along with a trace of *p*-cymene. ^1^H NMR (500 MHz, acetone-*d*_6_): δ/ppm = 10.73 (br), 9.98 (br), 9.62 (d, *J* = 5.3 Hz), 9.31 (d, *J* = 7.8 Hz) (2H), 8.94 (br, 0.6H), 8.59 (br), 8.41 (s) (1H), 8.12 (m-br), 7.66–7.61 (m) (1.4H), 6.48 (br), 6.44 (br), 6.37 (br), 6.27 (br), 6.10 (d, *J* = 5.8 Hz), 6.02 (d, *J* = 5.6 Hz), 5.87 (d, *J* = 5.8 Hz), 5.80 (d, *J* = 5.5 Hz) (4H), 2.30 (br), 2.24 (s) (3H), 1.31 (br), 1.16 (d, *J* = 6.9 Hz), 1.13 (d, *J* = 6.9 Hz) (6H). Next, volatiles were removed under vacuum and the IR spectrum of the resulting dark yellow-green solid was recorded, showing no bands ascribable to ruthenium-coordinated cyanate (2100–2300 cm^–1^ region—see the SI). Further investigations were hampered by the instability of the products in solution.

#### 4.5.2 Reaction of [**1b**]PF_6_ with [Bu_4_N][OCN] and Subsequent Protonation

A solution of [**1b**]PF_6_ (12 mg, 0.021 mmol) in anhydrous CH_2_Cl_2_ (2 mL) was treated with [Bu_4_N][OCN] (6 mg, 0.022 mmol) and stirred at room temperature under N_2_ in the dark for 24 h. The solvent was removed under reduced pressure affording a dark yellow-green solid. Quantitative conversion and the presence of two major sets of signals for ruthenium *p*-cymene complexes were assessed by ^1^H NMR.

^1^H NMR (400 MHz, acetone-*d*_6_): δ/ppm = 10.04 (m-br), 9.54 (d, *J* = 5.4 Hz) (1H), 9.31 (d, *J* = 8.1 Hz), 8.91 (br) (1H), 8.48 (br), 8.06 (t, *J* = 7.7 Hz), 7.54 (t, *J* = 6.5 Hz) (2H), 6.44 (m-br), 6.38 (m-br), 6.04 (d, *J* = 6.0 Hz), 5.98 (d, *J* = 6.2 Hz), 5.82 (d, *J* = 6.1 Hz), 5.73 (d, *J* = 6.2 Hz) (4H), 2.93–2.75 (m-br, 4H), 2.38, 2.23 (s, 3H), 1.29 (br), 1.16 (d, *J* = 6.9 Hz), 1.16 (d, *J* = 6.9 Hz) (6H).

Next, the solid was dissolved in CH_2_Cl_2_ (2 mL) and treated with a 9.5 × 10^–2^ M TsOH solution in CHCl_3_ (0.25 mL, 0.024 mmol). The reaction mixture was stirred at room temperature for 2 h, then the volatiles were removed under vacuum. The resulting red-brown solid was analyzed by ^1^H NMR, showing three sets of signals for ruthenium *p*-cymene complexes. ^1^H NMR (400 MHz, acetone-*d*_6_): δ/ppm = 9.79 (d, *J* = 4.3 Hz), 9.74–9.65 (m) (1H), 9.17–9.09 (m), 8.84 (d, *J* = 7.7 Hz) (1H), 8.45 (t, *J* = 7.6 Hz), 8.31–8.23 (m) (1H), 8.09–8.02 (m), 7.87–7.79 (m) (1H), 7.68 (d, *J* = 7.8 Hz, TsO), 7.13 (d, *J* = 7.5 Hz, TsO), 6.40–6.19 (m), 6.15 (d, *J* = 5.9 Hz), 6.05–5.97 (m, 4H), 3.24, 2.75, 2.74 (s, 3H), 2.30, 2.26, 2.24 (s, 3H), 1.31 (d, *J* = 6.8 Hz), 1.27 (d, *J* = 6.9 Hz), 1.25–1.18 (m) (6H).

### 4.6 Tetrazine/Chalcogenocyanate Reactivity: UV–Vis Monitoring and Kinetics Analysis

A freshly prepared solution of [**1a**]PF_6_ (2.2 × 10^–4^ M) or [**1b**]PF_6_ (2.0 × 10^–4^ M) in acetone (5 mL) was analyzed by UV–vis (*t*_0_), then treated with an equimolar amount of [Bu_4_N][ECN] (E = O, S, Se) solution in acetone (0.17 M; 7 μL for [**1a**]^+^, 6 μL for [**1b**]^+^). The solution was stirred for a few seconds, transferred into a 1 mL quartz cuvette, and monitored by UV–vis during the next 4 h at room temperature (21 ± 1 °C). The final spectrum was recorded after 4 h (*t*_∞_). Analogous experiments were carried out with [**1a**]PF_6_ (2.2 × 10^–4^ M) and [**1b**]PF_6_ (3.5 × 10^–4^ M) solutions in acetone treated with KSCN (10 μL of a 0.85 M solution in water). In parallel, solutions of [**1a**]PF_6_ (5.2 × 10^–4^ M) or [**1b**]PF_6_ (5.9 × 10^–4^ M) in acetone were kept at room temperature and monitored by UV–vis for 17 or 48 h, respectively. Solvent-subtracted UV–vis spectra are reported in Figures S66–S73, S76, and S77. An isosbestic point was detected in the 440–450 nm range for all experiments involving thiocyanate or selenocyanate. Assuming a simple reacting system (reactant → product), complete conversion at *t*_∞_ and unchanged total concentration during the experiment (*c*_0_ = *c*_R_ + *c*_P_, wherein *c*_0_ is the initial concentration and R represents the tetrazine-based reagent), the conversion *y* = (*c*_0_ – *c*_R_)/*c*_0_ was calculated as *y* = (*A*_t_ – *A*_0_)/(*A*_∞_ – *A*_0_), where *A_t_* is the absorbance at a given time, and *A*_0_ and *A*_∞_ are the absorbances in the initial (*t*_0_) and final (*t*_∞_) spectra, respectively. Conversion/time profiles are reported in Figures S74 and S75. Under these conditions, *c*_R_ = *c*_0_·[(*A*_t_ – *A*_∞_)/(*A*_0_ – *A*_∞_)]. By combining this expression into the integrated rate-law for a second-order reaction (1/*c*_R_(*t*) – 1/*c*_0_ = *k*_2_·*t* since the two reactants are equimolar),^52^ we obtain *w*(*t*) = 1 + *k*_2_·*c*_0_·*t*, wherein *w*(*t*) = [(*A*_0_ – *A*_∞_)/(*A*_t_ – *A*_∞_)]. Least-squares linear regression of (*w*(*t*); *t*) data gave an equation of the type *w*(*t*) = *a*·*t* + *b*; therefore, the rate constant was calculated as *k*_2_ = *a*/*c*_0_. Plots are shown in Figures S78–S81 and *k*_2_ data are compiled in Table 1. The wavelength of the maximum absorbance change in the 350–450 nm range was used for the calculations in the thio- and selenocyanate reactions; 575 and 760 nm for the decomposition of [**1a**]^+^ and [**1b**]^+^, respectively. The UV–vis spectra of the reactions between [**1a**,**b**]PF_6_ and [Bu_4_N][OCN] revealed a more complex evolution over time that did not fit the simple model adopted; conversion/time profiles based on the very rapid absorbance change at 370 nm were calculated for comparative purposes.

### 4.7 Tetrazine/Chalcogenocyanate Reactivity: Control Experiments

Under an Ar atmosphere, a tetrazine-based reagent (5–10 mg) was dissolved in anhydrous CH_2_Cl_2_ (4 mL) and treated with an equimolar amount of a chalcogenocyanate derivative. The solution was stirred at room temperature for 24 h under protection from the light. Therefore, volatiles were removed under vacuum and the residue was analyzed by ^1^H NMR (CDCl_3_). The following combinations were tested: 3-(2-pyridyl)-1,2,4,5-tetrazine + [Bu_4_N][ECN] (E = O, S) or [Et_3_NH][ECN] (E = S, Se); 3-(2-pyridyl)-6-methyl-1,2,4,5-tetrazine + [Bu_4_N][ECN] (E = O, S); 3,6-diphenyl-1,2,4,5-tetrazine + [Et_3_NH][ECN] (E = S, Se); [**1a**]PF_6_ + phenyl isocyanate or isopropyl isothiocyanate. Similarly, a solution of 3-(2-pyridyl)-6-methyl-1,2,4,5-tetrazine (*ca.* 7 mg) and an equimolar amount of K[ECN] (E = S, Se) in acetone (10 mL) was stirred at reflux temperature overnight under protection from the light, then treated as above. No reactivity of the tetrazine-based reagent was observed in each case, except for a minor, unselective degradation of 3-(2-pyridyl)-1,2,4,5-tetrazine that also occurred with organic solutions containing the tetrazine alone.

### 4.8 Synthesis and Characterization of Bipyridine Chalcogenocyanato Complexes

#### 4.8.1 General Procedure

A solution of [RuCl(2,2′-bipyridine)(η^6^-*p*-cymene)]PF_6_ (40–100 mg) in acetone (10 mL) was treated with K[ECN] (E = S, Se; 1.0 equiv) and stirred at room temperature overnight (*ca*. 14 h). The resulting suspension was filtered over celite and the filtrate was taken to dryness under vacuum. The solid was washed with Et_2_O and hexane, dried under vacuum (40 °C), and kept under N_2_ (long-term storage). Note: a slightly different procedure was used with Na[OCN] (see below) since its insolubility in acetone prevented any reaction from occurring. Next, a fraction of the compound (*ca*. 20 mg) was suspended in deaerated EtOH (8 mL) under N_2_ and stirred at reflux up to 14 h. Volatiles were removed under vacuum, and the residue was analyzed by ^1^H and ^13^C NMR.

##### 4.8.1.1 [Ru(OCN)(2,2′-bipyridine)(η^6^-*p*-cymene)]PF_6_, [**4**]PF_6_

**Chart 15:**
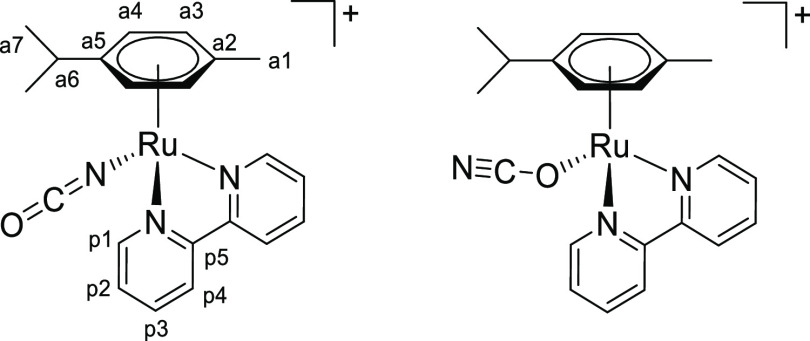
Structures of [**4**^***N***^]^+^ (Left) and [**4**^***O***^]^+^ (Right) Isomers (Numbering Refers to C Atoms)^49^

A suspension of [RuCl(2,2′-bipyridine)(η^6^-*p*-cymene)]PF_6_ (53 mg, 0.093 mmol) in acetone (5 mL) was treated with an aqueous solution of NaOCN (1.9 × 10^–2^ mol·L^–1^, 5 mL, 0.095 mmol) and stirred at room temperature. After 24 h, the resulting light-yellow solution was taken to dryness under vacuum. The residue was suspended in CH_2_Cl_2_ and filtered over Celite. Volatiles were removed under vacuum from the filtrate solution, affording a yellow, hygroscopic solid, which was washed with diethyl ether and dried under vacuum. Yield: 47 mg, 87%. Isomer (κ*N*/κ*O*) ratio (^1^H NMR, acetone-*d*_6_): 1.2. No changes in the isomer ratio were observed after the treatment in refluxing EtOH (14 h). IR (solid state): ṽ/cm^–1^ = 3380w-br (H_2_O-moisture), 3076w, 2968w, 2928w, 2874w, 2217m (CN), 1605m, 1497w, 1470m, 1446m, 1388w, 1377w, 1364w, 1327m-sh, 1316m (κ*N*-CO), 1280w, 1243w, 1200w, 1174w, 1160w, 1137w (κ*O*-CO), 1124w, 1109w, 1092w, 1073w, 1057w, 1033w, 880m-sh, 831s (PF6), 805 s-sh, 766s, 727s, 695m, 675m (Chart 15).

[**4**^***N***^]^+^. ^1^H NMR (400 MHz, acetone-*d*_6_): δ/ppm = 9.62 (d, ^3^*J*_HH_ = 5.4 Hz, 2H, p1), 8.61 (d, ^3^*J*_HH_ = 8.1 Hz, 2H, p4), 8.30 (t, ^3^*J*_HH_ = 7.8 Hz, 2H, p3), 7.83–7.78 (m, 2H, p2), 6.25 (d, ^3^*J*_HH_ = 6.2 Hz, 2H, a4), 5.99 (d, ^3^*J*_HH_ = 6.2 Hz, 2H, a3), 2.81–2.68 (m, 1H,* a6), 2.30 (s, 3H, a1), 1.08 (d, *J* = 6.9 Hz, 6H,* a7). *Superimposed to the corresponding resonances of [**4**^***O***^]^+^. ^13^C{^1^H} NMR (400 MHz, acetone-*d*_6_): δ/ppm = 156.7 (p1), 155.8 (p5), 140.8 (p3), 128.5 (p2), 127.8 (CO), 124.6 (p4), 105.9 (a5), 104.7 (a2), 87.6 (a4), 85.4 (a3), 31.8 (a6), 22.2 (a7), 18.8 (a1).

[**4**^***O***^]^+^. ^1^H NMR (400 MHz, acetone-*d*_6_): δ/ppm = 9.68 (d, ^3^*J*_HH_ = 5.3 Hz, 2H, p1), 8.66 (d, ^3^*J*_HH_ = 8.0 Hz, 2H, p4), 8.36 (t, ^3^*J*_HH_ = 7.8 Hz, 2H, p3), 7.88–7.83 (m, 2H, p2), 6.32 (d, ^3^*J*_HH_ = 6.2 Hz, 2H, a4), 6.08 (d, ^3^*J*_HH_ = 6.2 Hz, 2H, a3), 2.81–2.68 (m, 1H,* a6), 2.27 (s, 3H, a1), 1.09 (d, ^3^*J*_HH_ = 7.0 Hz, 6H,* a7). *Superimposed to the corresponding resonances of [**4**^***N***^]^+^. ^13^C{^1^H} NMR (400 MHz, acetone-*d*_6_): δ/ppm = 156.5 (p1), 155.8 (p5), 141.2 (p3), 128.8 (p2), 128.1 (CO), 124.9 (p4), 106.1 (a5), 105.4 (a2), 88.2 (a4), 85.0 (a3), 31.9 (a6), 22.3 (a7), 18.8 (a1).

##### 4.8.1.2 [Ru(SCN)(2,2′-bipyridine)(η^6^-*p*-cymene)]PF_6_, [**5**]PF_6_

**Chart 16:**
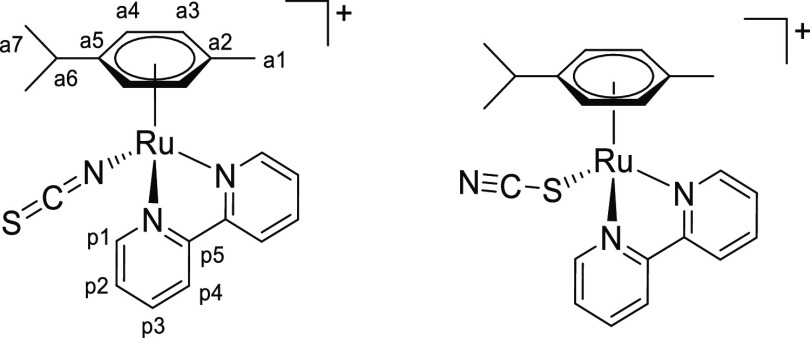
Structures of [**5**^***N***^]^+^ (Left) and [**5**^***S***^]^+^ (Right) Isomers (Numbering Refers to C Atoms)^49^

Prepared from [RuCl(2,2′-bipyridine)(η^6^-*p*-cymene)]PF_6_ (41 mg, 0.072 mmol) and KSCN (7 mg, 0.072 mmol). Previously reported using a different synthetic procedure.^39b^ Orange, hygroscopic solid; yield: 35 mg, 82%. Isomer (κ*N*/κ*S*) ratio (^1^H NMR, acetone-*d*_6_) ≥15: in one case, no trace of the κ*S* isomer was detected. No changes in the isomer ratio were observed in the ^1^H NMR spectrum after 6 h at room temperature. Following the treatment in refluxing EtOH: orange solid. Isomer (κ*N*/κ*S*) ratio (^1^H NMR, acetone-*d*_6_): 0.64 (6 h); 0.23 (14 h). A third, unidentified and previously unreported^39b^ species was also obtained, [**5**^***X***^]^+^ (20–30% with respect to [**5**^***S***^]^+^ + [**5**^***N***^]^+^) (Chart 16).

IR (solid state, κ*N*/κ*S* ratio 15): ṽ/cm^–1^ = 3435w-br (H_2_O-moisture), 3112w, 3053w, 2965w, 2926w, 2871w, 2049s (κ*N*-SCN), 1702w, 1604m, 1495w-sh, 1469m, 1444s, 1387–1364w, 1312w, 1277w, 1243w, 1224w, 1157w, 1107w, 1072w, 1059w, 1032w, 876s-sh, 830s (PF_6_), 764s, 727s, 674 m. IR (solid state, κ*N*/κ*S* ratio 0.23): ṽ/cm^–1^ = 3078w, 2956m, 2924m, 2868–2855m, 2103m (κ*S*-SCN), 2051m-sh (κ*N*-SCN), 1730w, 1703w, 1605w, 1495w-sh, 1467m, 1446m, 1424w, 1388w, 1378w, 1365w, 1314w, 1278w, 1245w, 1224w, 1160w, 1123w, 1109w, 1073w, 1057w, 1033w, 878w-sh, 829s (PF6), 764s, 741s-sh, 725s, 700m, 677m.

[**5**^***N***^]^+^. ^1^H NMR (400 MHz, acetone-*d*_6_): δ/ppm = 9.65 (d, ^3^*J*_HH_ = 5.4 Hz, 2H, p1), 8.63 (d, ^3^*J*_HH_ = 7.9 Hz, 2H, p4), 8.31 (t, ^3^*J*_HH_ = 7.8 Hz, 2H, p3), 7.86–7.79 (m, 2H, p2), 6.28 (d, ^3^*J*_HH_ = 6.2 Hz, 2H, a4), 6.01 (d, ^3^*J*_HH_ = 6.1 Hz, 2H, a3), 2.78 (hept, ^3^*J*_HH_ = 7.0 Hz, 2H, a6), 2.30 (s, 3H, a1), 1.09 (d, ^3^*J*_HH_ = 6.9 Hz, 6H, a7). ^13^C{^1^H} NMR (100 MHz, acetone-*d*_6_): δ/ppm = 156.8 (p1), 155.6 (p5), 140.7 (p3), 138.4 (CS), 128.6 (p2), 124.6 (p4), 105.7 (a5), 104.5 (a2), 87.6 (a4), 85.3 (a3), 31.7 (a6), 22.2 (a7), 18.9 (a1).

[**5**^***S***^]^+^. ^1^H NMR (400 MHz, acetone-*d*_6_): δ/ppm = 9.46 (d, ^3^*J*_HH_ = 5.3 Hz, 2H, p1), 8.69 (d, ^3^*J*_HH_ = 8.0 Hz, 2H, p4), 8.34 (t, ^3^*J*_HH_ = 8.0 Hz, 2H, p3), 7.86–7.81 (m, p2), 6.34 (d, ^3^*J*_HH_ = 6.2 Hz, 2H, a4), 6.08 (d, ^3^*J*_HH_ = 6.2 Hz, 2H, a3), 2.78 (hept, ^3^*J*_HH_ = 6.6 Hz, a6), 2.34 (s, 3H, a1), 1.10 (d, ^3^*J*_HH_ = 6.9 Hz, a7).

^13^C{^1^H} NMR (100 MHz, acetone-*d*_6_): δ/ppm = 157.1 (p1), 156.0 (p5), 141.0 (p3), 128.9 (p2), 125.1 (p4), 118.0 (CS), 109.3 (a5), 105.9 (a2), 89.6 (a4), 87.2 (a3), 31.8 (a6), 22.3 (a7), 18.4 (a1).

[**5**^***X***^]^+^. ^1^H NMR (400 MHz, acetone-*d*_6_): δ/ppm = 9.71 (d, *J* = 5.3 Hz, 2H), 8.40 (t, *J* = 7.7 Hz, 2H), 7.94–7.88 (m, 2H), 6.44 (d, *J* = 6.1 Hz, 2H), 6.22 (d, *J* = 6.2 Hz, 2H), 2.29 (s, 3H).

##### 4.8.1.3 [Ru(SeCN)(2,2′-bipyridine)(η^6^-*p*-cymene)]PF_6_, [**6**]PF_6_

**Chart 17:**
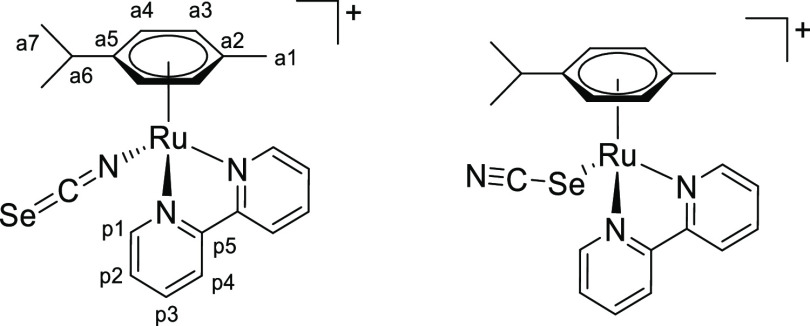
Structures of [**6**^***N***^]^+^ (Left) and [**6**^***Se***^]^+^ (Right) Isomers (Numbering Refers to C Atoms)^49^

Prepared from [RuCl(2,2′-bipyridine)(η^6^-*p*-cymene)]PF_6_ (100 mg, 0.175 mmol) and KSeCN (25 mg, 0.18 mmol). Orange-red hygroscopic solid; yield: 101 mg, 90%. Isomer (κ*N*/κ*Se*) ratio (^1^H NMR, acetone-*d*_6_): 7.0 (freshly prepared solution), 3.0 (after 14 h at room temperature). Following the treatment in refluxing EtOH: orange-red solid. Isomer (κ*N*/κ*Se*) ratio (^1^H NMR, acetone-*d*_6_): 0.85 (14 h) (Chart 17).

IR (solid state, κ*N*/κ*Se* ratio *ca*. 24): ṽ/cm^–1^ = 3414w-br, 3112w, 3052w, 2964w, 2925w, 2872w, 2058s (κ*N*-SeCN), 1704m, 1604m, 1494w, 1468m, 1444s, 1362m, 1312m, 1277w, 1245w, 1223w, 1157w, 1106w, 1092w, 1072w, 1056w, 1032w, 877m-sh, 836s (PF_6_), 805m-sh, 764s, 725s.

IR (solid state, κ*N*/κ*Se* ratio 0.85): ṽ/cm^–1^ = 3081w, 2957m, 2924m, 2869–2857m, 2113m (κ*Se*-SCN), 2070w-br (κ*N*-SeCN), 1729w, 1704w, 1667w, 1605m, 1494w-sh, 1467m, 1446m, 1422m-sh, 1387w, 1379w, 1365w, 1314w, 1277w, 1260w, 1244w, 1223w, 1160w, 1123w, 1108w, 1091w, 1073w, 1057w, 1032w, 877m-sh, 829s (PF_6_), 763s, 741s, 725s.

[**6**^***N***^]^+^. ^1^H NMR (400 MHz, acetone-*d*_6_): δ/ppm = 9.62 (d, ^3^*J*_HH_ = 5.4 Hz, 2H, p1), 8.62 (d, ^3^*J*_HH_ = 8.1 Hz, 2H, p4), 8.31 (td, ^3^*J*_HH_ = 7.9 Hz, ^4^*J*_HH_ = 1.4 Hz, 2H, p3), 7.82 (ddd, ^3^*J*_HH_ = 7.1, 5.7 Hz, ^4^*J*_HH_ = 1.3 Hz, 2H, p2), 6.25 (d, ^3^*J*_HH_ = 6.3 Hz, 2H, a4), 6.00 (d, ^3^*J*_HH_ = 6.3 Hz, 2H, a3), 2.78 (hept, ^3^*J*_HH_ = 6.9 Hz, 1H, a6), 2.30 (s, 3H, a1), 1.08 (d, ^3^*J*_HH_ = 7.0 Hz, 6H, a7). ^13^C{^1^H} NMR (100 MHz, acetone-*d*_6_): δ/ppm = 156.7 (p1), 155.8 (p5), 140.8 (p3), 131.0 (CSe), 128.6 (p2), 124.6 (p4), 105.9 (a5), 104.7 (a2), 87.7 (a4), 85.4 (a3), 31.8 (a6), 22.3 (a7), 18.9 (a1). ^77^Se NMR (76 MHz, acetone-*d*_6_): δ/ppm = −303.

[**6**^***Se***^]^+^. ^1^H NMR (400 MHz, acetone-*d*_6_): δ/ppm = 9.42 (d, ^3^*J*_HH_ = 5.3 Hz, 2H, p1), 8.69 (d, ^3^*J*_HH_ = 8.1 Hz, 2H, p4), 8.32 (td, ^3^*J*_HH_ = 8.0 Hz, ^4^*J*_HH_ = 1.3 Hz, 2H,* p3), 7.84–7.78 (m, 2H,* p2), 6.29 (d, ^3^*J*_HH_ = 6.4 Hz, 2H, a4), 6.07 (d, ^3^*J*_HH_ = 6.4 Hz, 2H, a3), 2.80–2.75 (m, 1H,* a6), 2.39 (s, 3H, a1), 1.11 (d, ^3^*J*_HH_ = 5.7 Hz, 6H, a7). *Superimposed to the corresponding resonances of [**6**^***N***^]^+^. ^13^C{^1^H} NMR (100 MHz, acetone-*d*_6_): δ/ppm = 157.3 (p1), 155.9 (p5), 140.7 (p3), 128.7 (p2), 125.1 (p4), 109.7 (a5), 105.0 (a2), 102.6 (CSe), 89.0 (a4), 87.0 (a3), 31.9 (a6), 22.3 (a7), 18.7 (a1). ^77^Se NMR (76 MHz, acetone-*d*_6_): δ/ppm = −106.

### 4.9 Computational Details

Preliminary geometry optimizations were performed using the PBEh-3c method, which is a reparametrized version of PBE0^53^ (with 42% HF exchange) that uses a split-valence double-ζ basis set (def2-mSVP), with ECP on Ru^54,55^ and adds three corrections considering dispersion, basis set superposition, and other basis set incompleteness effects.^56−58^ The viability of the PBEh-3c method toward transition-metal complexes was recently highlighted.^59^ Further refinement of the structures was carried out with the hybrid meta-GGA DFT functional TPSS0,^60^ with 25% HF exchange in combination with Ahlrichs’ def-2 TZVP basis set, with relativistic pseudopotential on Ru.^54,55^ The C-PCM implicit solvation model was added to all calculations, considering acetone as continuous medium.^61,62^ IR simulations were carried out using the harmonic approximation, from which zero-point vibrational energies and thermal corrections (*T* = 298.15 K) were obtained. The stationary points were characterized by verifying the presence of zero (ground states) or one (transition states) imaginary frequencies.^63^ The software used was ORCA version 5.0.3.^64^ The output was elaborated using MultiWFN, version 3.8.^65,66^ Cartesian coordinates of the DFT-optimized structures are collected in a separate .xyz file.

### 4.10 X-ray Crystallography

Crystal data and collection details for [RuCl(2,2′-bipyridine)(η^6^-*p*-cymene)]PF_6_ are reported in Table S3, and a view of the structure of the organometallic cation is given in Figure S79. A polymorph of the present structure^67^ and the crystal structure of a methanol solvate ([RuCl(2,2′-bipyridine)(η^6^-*p*-cymene)]PF_6_·1/2MeOH)^68^ were previously published.

Data were recorded on a Bruker APEX II diffractometer equipped with a PHOTON2 detector using Mo Kα radiation. The structures were solved by direct methods and refined by full-matrix least-squares based on all data using *F*^2^.^69^ Hydrogen atoms were fixed at calculated positions and refined using a riding model.

## Supporting Information Available

Synthesis and characterization of alkylammonium chalcogenocyanates; IR, NMR, ESI-MS, and UV–vis spectra of pyridyl tetrazine, pyridyl–triazine–thione, pyridyl–triazine–selone Ru complexes and related tetrazine/chalcogenocyanate reactions; X-ray characterization of [RuCl(2,2′-bipyridine)(η^6^-*p*-cymene)]PF_6_ and IR/NMR spectra of 2,2′-bipyridine chalcogenocyanato Ru complexes; comparison of IR/NMR data for tetrazine, triazine, and chalcogenocyanato complexes (PDF)
Cartesian coordinates of the DFT-optimized structures (XYZ)

## Supplementary Material

ic3c00459_si_001.pdf

ic3c00459_si_002.xyz
