# Supplementary material for: Triazine Chalcogenones from Thiocyanate or Selenocyanate Addition to Tetrazine Ligands in Ruthenium Arene Complexes
Source: Inorg Chem. 2023 May 11;62(20):7814–33. doi: 10.1021/acs.inorgchem.3c00459 (PMC10263068; doi:10.1021/acs.inorgchem.3c00459)
Supplement: Supplementary file 1 — ic3c00459_si_001.pdf [file ic3c00459_si_001.pdf]

Supporting Information

Triazine-Chalcogenones from Thiocyanate or Selenocyanate addition to Tetrazine Ligands in Ruthenium Arene Complexes

Lorenzo Bonaldi,[a] Marco Bortoluzzi,[b] Stefano Zacchini,[c] Guido Pampaloni,[a] Fabio Marchetti,[a] Lorenzo Biancalana [a*]*

[a] University of Pisa, Department of Chemistry and Industrial Chemistry, Via G. Moruzzi 13, I-56124 Pisa, Italy

[b] Ca' Foscari University of Venice, Dipartimento di Scienze Molecolari e Nanosistemi, Via Torino 155, I-30175 Mestre, Venezia, Italy

[c] Universita di Bologna, Dipartimento di Chimica Industriale "Toso Montanari", Viale del Risorgimento 4, I-40136 Bologna, Italy

* Email: lorenzo.biancalana@unipi.it, fabio.marchetti@unipi.it

Table of contents Page(s)

1. Preparation and characterization of alkylammonium chalcogenocyanates (**Chart S1**, **Figure S1**) S3-S5

2. ^{1}H NMR spectra and stability of pyridyl-tetrazines (**Chart S2**, **Figures S2-S3**) S6-S7

3. IR and NMR spectra of ruthenium pyridyl-tetrazine complexes (**Figures S4-S16**) S8-S14

4. IR, NMR and ESI-MS spectra of pyridyl-triazine-thione complexes (**Figures S17-S46**) S15-S29

5. IR, NMR and ESI-MS spectra of pyridyl-triazine-selone complexes (**Figures S47-S62**). S30-S37

6. Reactivity of pyridyl-tetrazine complexes with cyanate: IR and NMR spectra (**Figures S63-S65**). S38-S39

7. Tetrazine/chalcogenocyanate reactivity: comparison of NMR data (**Tables S1-S2**) S40-S41

8. Tetrazine/chalcogenocyanate reactivity: UV-Vis monitoring and kinetic analysis (**Figures S66-S81**) S42-S46

9. Preparation and X-Ray characterization of [RuCl(2,2'-bipyridine)(η^6-*p*-cymene)]PF_6 (**Chart S3**, **Figure S79**, **Table S3**) S47-S49

10. Characterization of bipyridine chalcogenocyanato complexes (**Figures S83-S98**, **Table S4**) S50-S58

11. References S59

Preparation and characterization of alkylammonium chalcogenocyanates

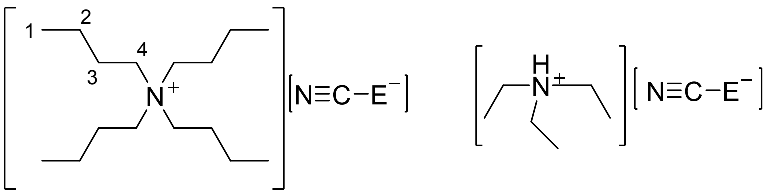

Chart S1. Structures of $[Bu_4N][ECN]$ and $[Et_3NH][ECN]$, E = O, S, Se (numbering refers to C atoms).

[Bu$_4$N][OCN]. A suspension of $[Bu_4N]Br$ (1.55 g, 4.80 mmol) and NaOCN (315 mg, 4.84 mmol) in water (20 mL) was stirred at room temperature for 24 h. The resulting colorless solution was extracted with CH_2Cl_2 (4 x 20 mL) and the combined organic solutions were taken to dryness under vacuum, affording a colorless solid. Yield 1.20 g, 88 %. Previously prepared from $[Bu_4N]Br$ and K[OCN] using an anion-exchange resin[1a] or by metathesis in water or ethanol using $[Bu_4N]BF_4$ and K[OCN].[1b] Λ_m (acetone, $1.0{\cdot}10^{-3}$ M) = 165 $S{\cdot}cm^2{\cdot}mol^{-1}$. IR (solid state): $\tilde{\upsilon}/cm^{-1}$ = 3411vw, 2959s, 2874m, 2192w, 2141vs (CN), 1633vw, 1473m, 1464m, 1396vw, 1380w, 1364vw, 1275vw, 1240vw, 1166vw, 1109w, 1067w, 1058w, 1031w, 992vw, 959vw, 922w, 897w, 882m, 800w, 738w. $^{13}C\{^1H\}$ NMR (400 MHz, acetone/C_6D_6): δ/ppm = 129.0 (CN), 59.9 (C^4), 25.1 (C^3), 20.9 (C^2), 14.5 (C^1).

[Bu$_4$N][SCN]. A solution of $[Bu_4N]Br$ (1.7 g, 5.2 mmol) in acetone (15 mL) was treated with KSCN (510 mg, 5.2 mmol) and stirred for 3 h at room temperature. The resulting suspension was filtered over celite and the filtrate was taken to dryness under vacuum, affording a colorless solid. Yield: 1.6 g, 99 %. Previously obtained by CH_2Cl_2 extraction from an aqueous solution of the same reagents.[2] Λ_m (acetone, $1.0{\cdot}10^{-3}$ M) = 206 $S{\cdot}cm^2{\cdot}mol^{-1}$. IR (solid state): $\tilde{\upsilon}/cm^{-1}$ = 3402vw, 2960s, 2935m, 2874m, 2791vw, 2054vs (CN), 2013wv, 1635vw, 1469s, 1382m, 1320vw, 1245vw, 1168w, 1111w, 1066w, 1037w, 1010vw, 929w, 879m, 802vw, 734m. $^{13}C\{^1H\}$ NMR (acetone/C_6D_6): δ/ppm = 131.7 (CN), 59.8 (C^4), 25.0 (C^3), 20.9 (C^2), 14.4 (C^1).

[Bu_4N][SeCN]. Prepared as described for [Bu_4N][SCN] using [Bu_4N]Br (1.1 g, 3.5 mmol) and KSeCN (510 mg, 3.5 mmol). The resulting waxy solid was triturated in Et_2O/hexane 1:1 v/v and the suspension was filtered. The solid (powder) was washed with hexane, dried under vacuum (40 °C) and stored under N_2. Colorless solid with a faint pink shade, yield: 1.1 g, 91 %. Previously obtained by precipitation from an EtOH solution of the same reagents.[3] Λ_m (acetone, $1.0·10^{-3}$ M) = 207 $S·cm^2·mol^{-1}$. IR (solid state): $\tilde{\upsilon}$/cm^{-1} = 2959s, 2932m, 2873m, 2066s (CN), 2022w, 1470 m, 1382w, 1319w, 1245w, 1170w, 1111w, 1066w, 1038w, 995w, 929w, 878m, 807w, 738m. $^{13}C\{^1H\}$ NMR (acetone/C_6D_6): δ/ppm = 118.0 (CN), 59.9 (C^4), 25.0 (C^3), 20.9 (C^2), 14.4 (C^1). ^{77}Se NMR (acetone/C_6D_6): δ/ppm = − 300.3.

[Et_3NH][SCN]. A suspension of [Et_3NH]Cl (710 mg, 5.16 mmol) in acetone (15 mL) was treated with KSCN (500 mg, 5.14 mmol) and stirred for 4 h at room temperature. The resulting light orange suspension was filtered over Celite and the filtrate was taken to dryness under vacuum. The resulting pale orange hygroscopic solid was dried under vacuum over P_2O_5 to and stored under N_2. Yield: 800 mg, 97 %. IR (solid state): $\tilde{\upsilon}$/cm^{-1} = 3428w-br (H_2O – moisture), 2985w, 2949w, 2776w, 2684m, 2502w, 2048s (CN), 1634w, 1474m, 1449m, 1396m, 1359w, 1316w, 1293w, 1179w, 1161w, 1063w, 1033m, 1017m, 943w, 898w, 837w, 794w, 739w.

[Et_3NH][SeCN]. Prepared as described for [Et_3NH][SCN] using [Et_3NH]Cl (420 mg, 3.1 mmol) and KSeCN (440 mg, 3.1 mmol). Pale pink hygroscopic solid, stored under N_2. Yield: 590 mg, 92 %. IR (solid state): $\tilde{\upsilon}$/cm^{-1} = 2981m, 2946w, 2780w-sh, 2680m, 2634m, 2533m-sh, 2504m, 2055s (CN), 1474m, 1447m, 1397m, 1355w, 1319w, 1289w, 1170m, 1065w, 1035m, 896w, 835m, 798m, 740w.

Figure S1. Solid-state IR spectra (650-4000 cm^{-1}) of $[Bu_4N][ECN]$ (E = O, dark red line; E = S, yellow line; E = Se, orange line).

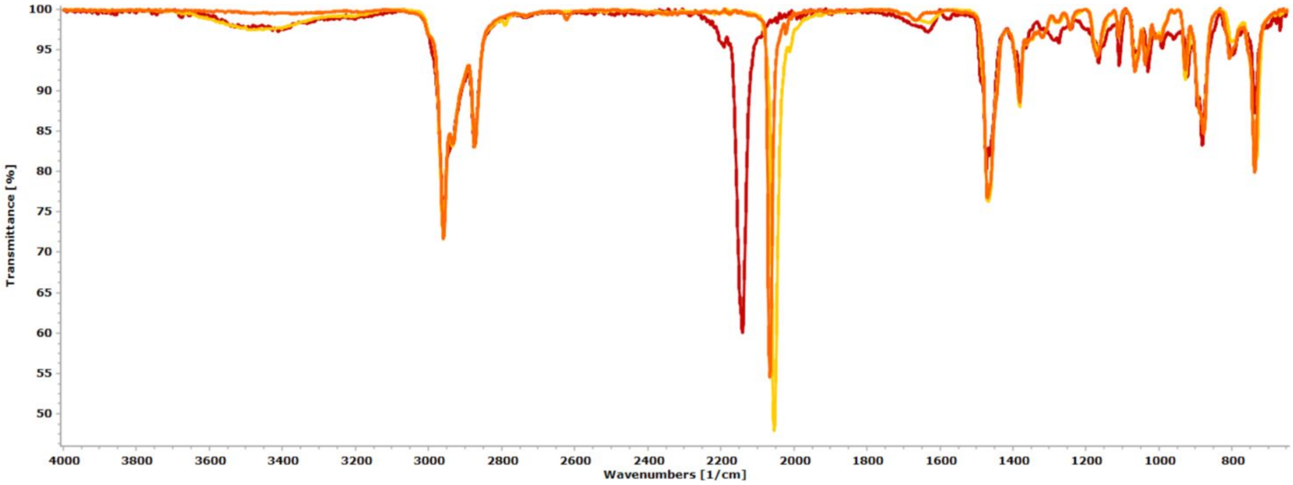

^{1}H NMR spectra and stability of pyridyl-tetrazines

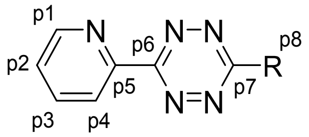

Chart S2. Structure of 3-(2-pyridyl)-1,2,4,5-tetrazine (R = H) and 3-(2-pyridyl)-6-methyl-1,2,4,5-tetrazine (R = Me). Numbering refers to C atoms.

3-(2-pyridyl)-1,2,4,5-tetrazine. Violet solid, best stored at − 20°C under N_2. A sample stored for 4-5 months at 4 °C became a dark red-violet sticky solid and was purified by silica chromatography (eluent: hexane/EtOAc 1:1 *V*/*V*). ^{1}H NMR (400 MHz, $CDCl_3$): δ/ppm = 10.37 (s, 1H, p7), 8.98 (d, $^3J_{HH}$ = 4.5 Hz, 1H, p1), 8.70 (d, $^3J_{HH}$ = 7.4 Hz, 1H, p4), 8.02 (td, $^3J_{HH}$ = 7.8 Hz, $^4J_{HH}$ = 1.7 Hz, 1H, p3), 7.60 (ddd, $^3J_{HH}$ = 7.6, 4.8 Hz, $^4J_{HH}$ = 0.9 Hz, 1H, p2).

3-(2-pyridyl)-6-methyl-1,2,4,5-tetrazine. Violet solid, stored at 4 °C for months with no evidence of decomposition. ^{1}H NMR (400 MHz, $CDCl_3$): δ/ppm = 8.95 (d, $^3J_{HH}$ = 4.3 Hz, 1H, p1), 8.65 (d, $^3J_{HH}$ = 7.9 Hz, 1H, p4), 7.98 (td, $^3J_{HH}$ = 7.8 Hz, $^4J_{HH}$ = 1.7 Hz, 1H, p3), 7.56 (ddd, $^3J_{HH}$ = 7.5, 4.7 Hz, $^4J_{HH}$ = 0.9 Hz, 1H, p2), 3.16 (s, 3H, p8).

Figure S2. ^{1}H NMR spectrum (400 MHz, $CDCl_3$) of 3-(2-pyridyl)-1,2,4,5-tetrazine.

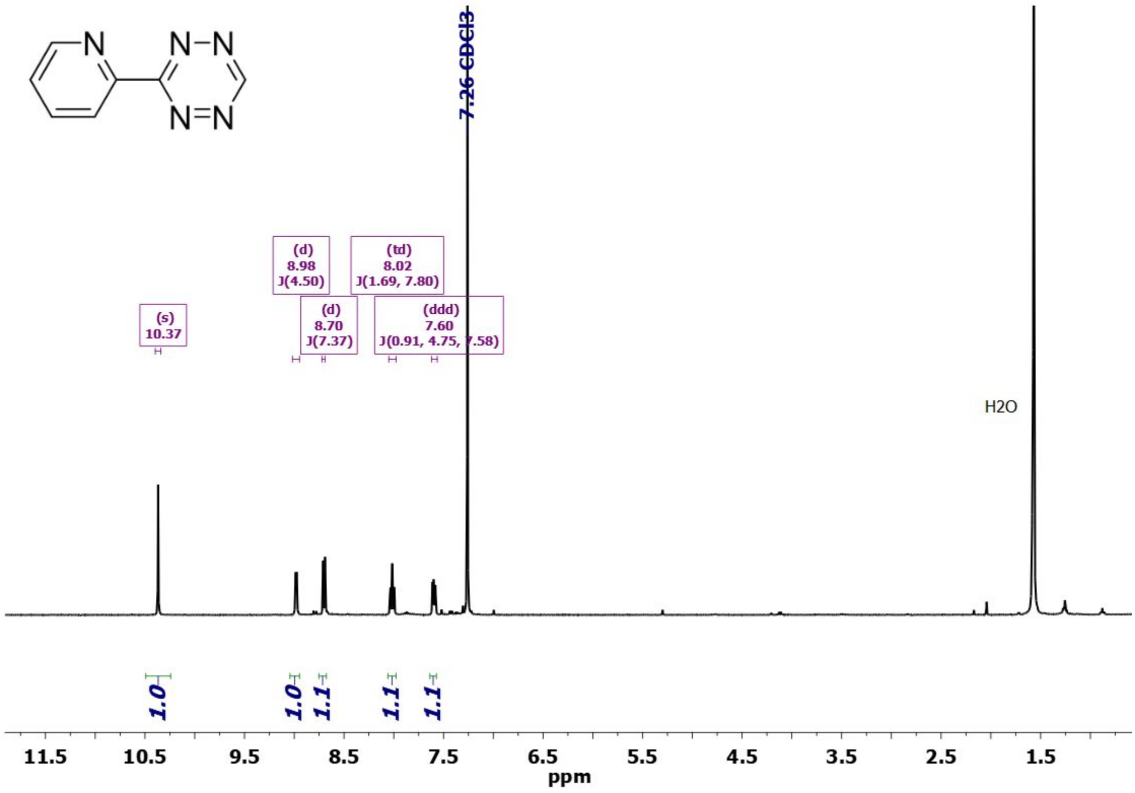

Figure S3. ^{1}H NMR spectrum (400 MHz, $CDCl_3$) of 3-(2-pyridyl)-6-methyl-1,2,4,5-tetrazine.

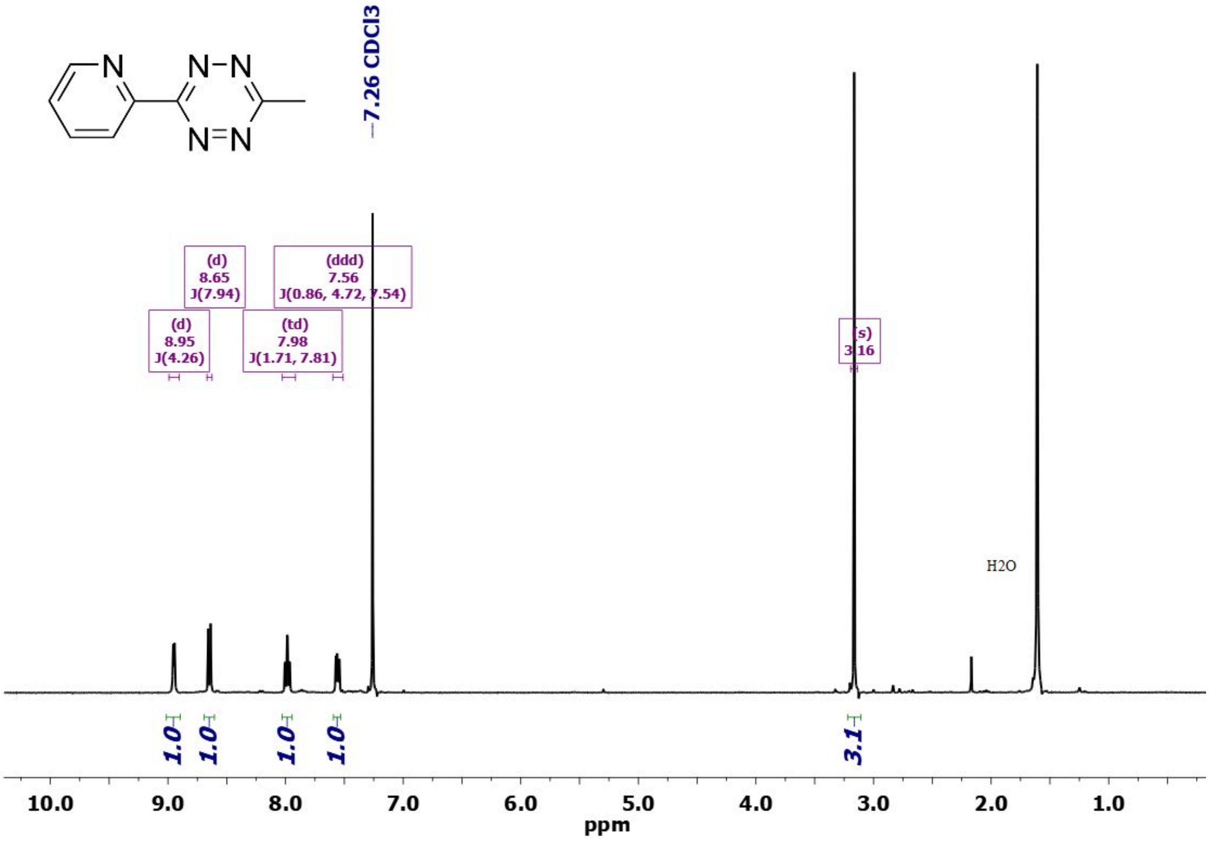

IR and NMR spectra of ruthenium pyridyl-tetrazine complexes

Figure S4. Solid-state IR spectrum (650-4000 cm^{-1}) of [RuCl{κ^2*N*-3-(2-pyridyl)-1,2,4,5-tetrazine}(η^6-*p*-cymene)]PF_6, [**1a**]PF_6.

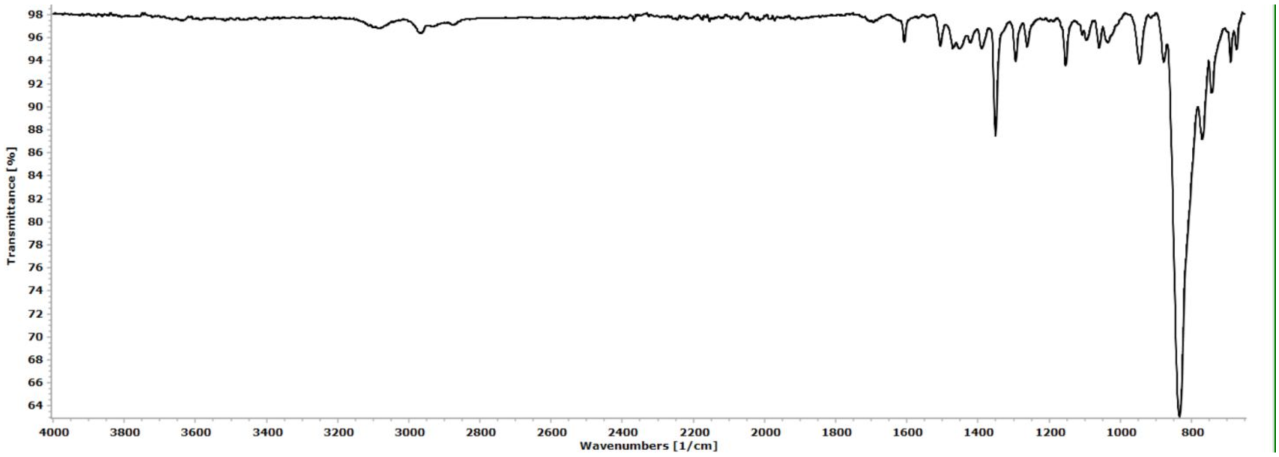

Figure S5. ^{1}H NMR spectrum (400 MHz, acetone-d_6) of [**1a**]PF_6.

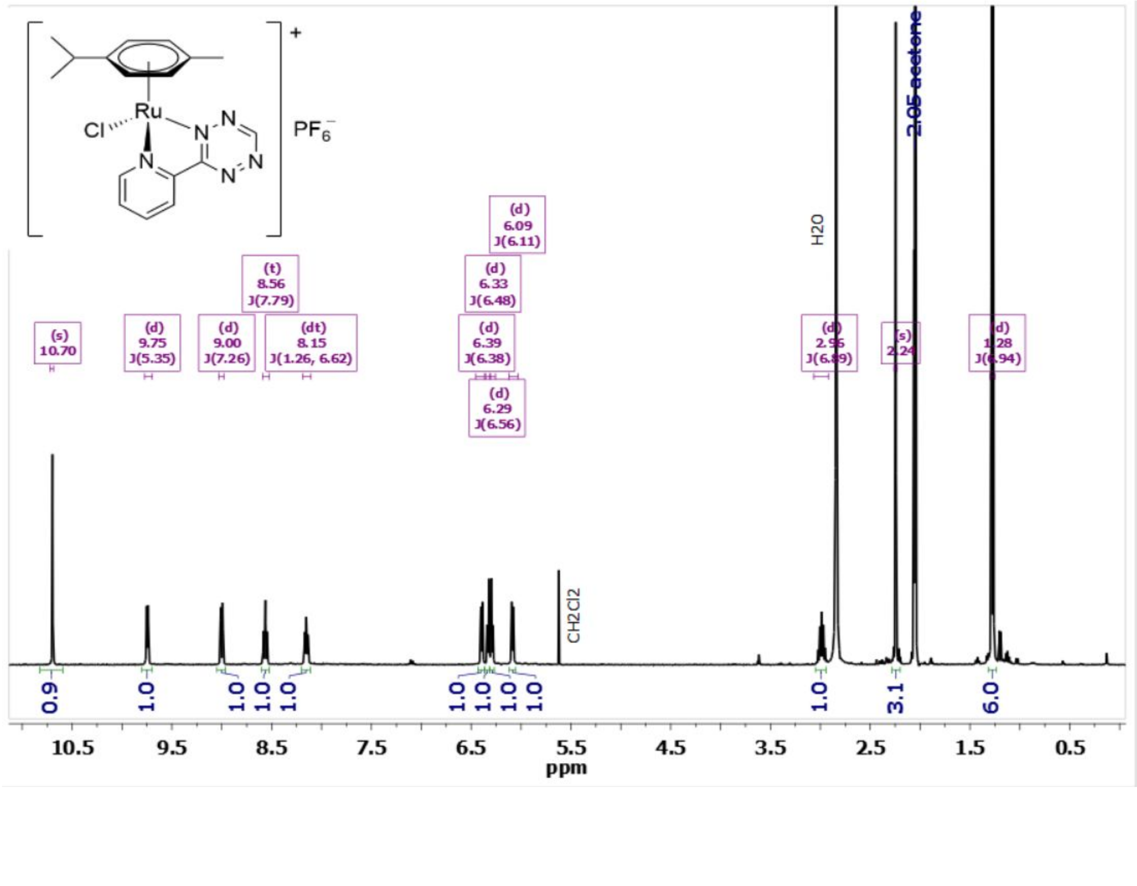

Figure S6. $^{13}C\{^{1}H\}$ NMR spectrum (126 MHz, acetone-d_6) of [**1a**]PF_6 (50-180 ppm range).

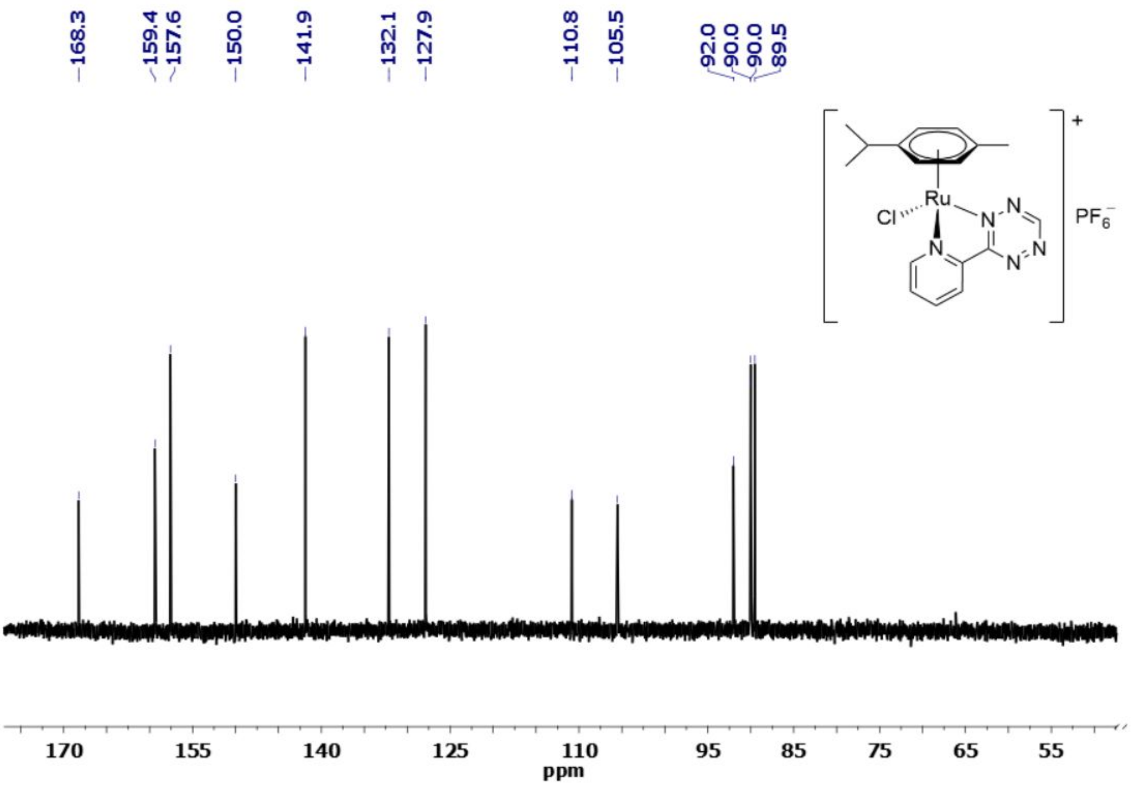

Figure S7. Solid-state IR spectrum (650-4000 cm^{-1}) of [RuCl{κ^2*N*-3-(2-pyridyl)-6-methyl-1,2,4,5-tetrazine}(η^6-p-cymene)]PF_6, [**1b**]PF_6.

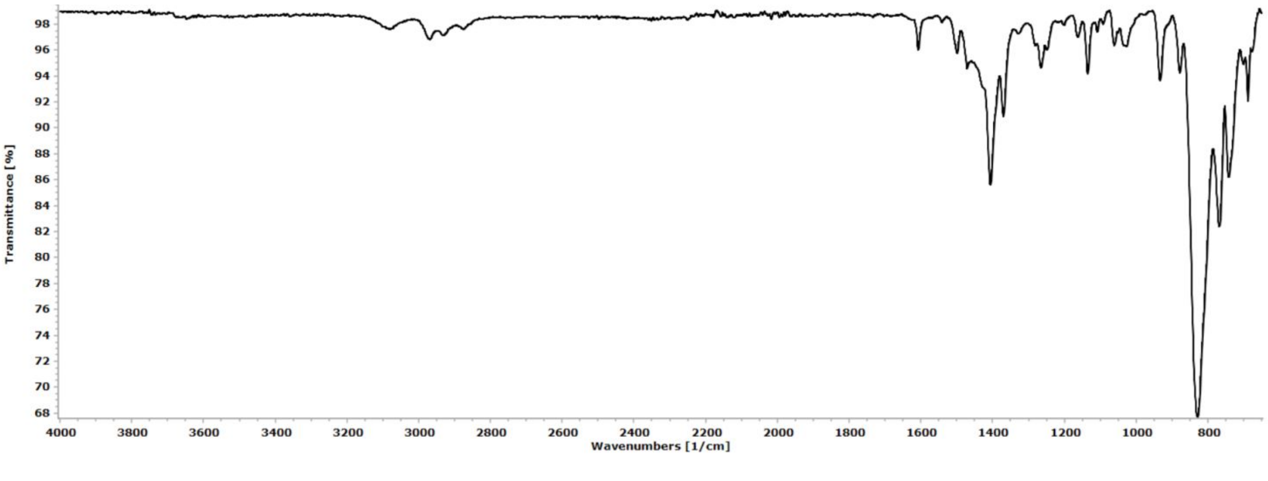

Figure S8. ^{1}H NMR spectrum (500 MHz, acetone-d_6) of [**1b**]PF_6.

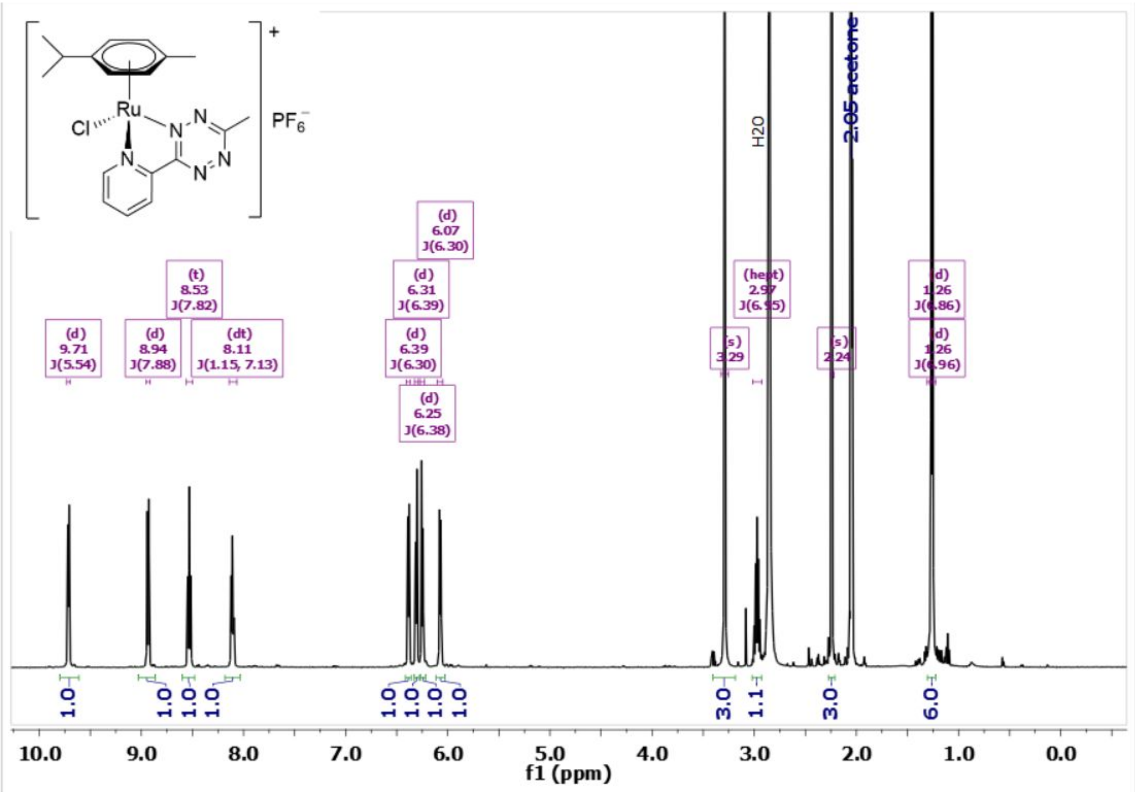

Figure S9. $^{13}C\{^{1}H\}$ NMR spectrum (126 MHz, acetone-d_6) of [**1b**]PF_6.

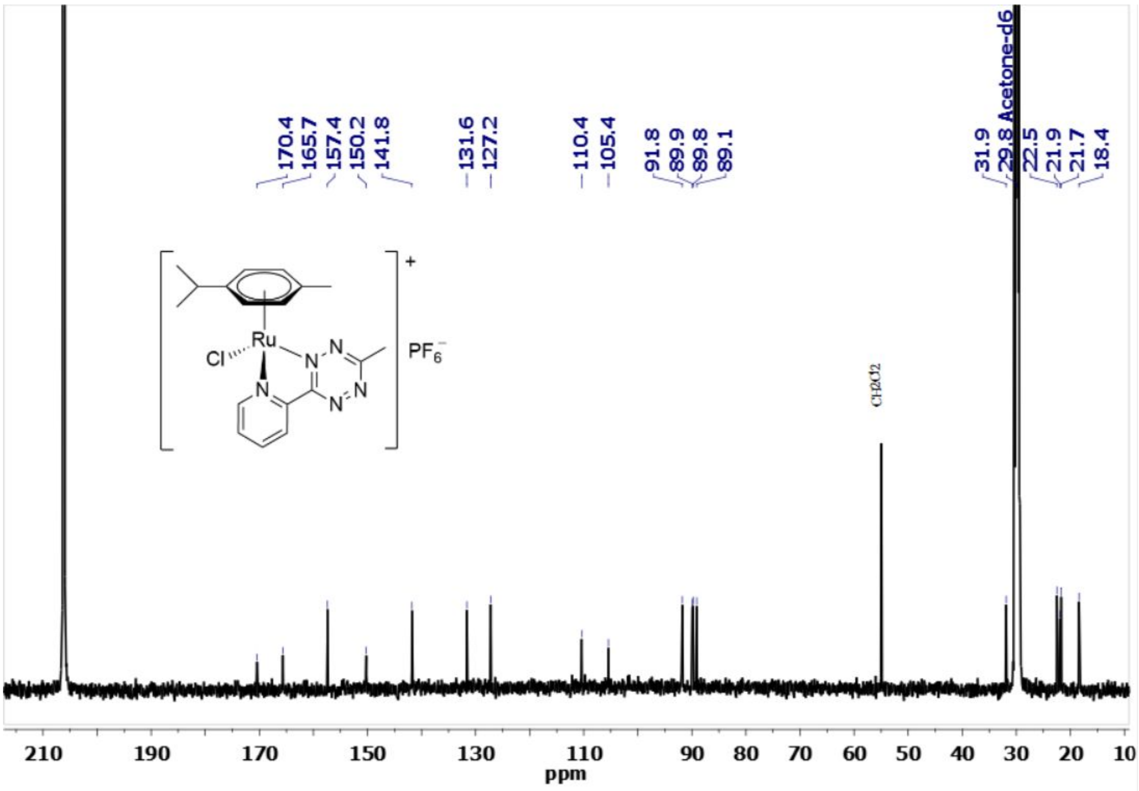

Figure S10. ^{1}H NMR spectrum (400 MHz, acetone-d$_6$) of [**1c**]PF$_6$. Resonances due to 3,6-di(2-pyridyl)-1,2,4,5-tetrazine are marked with asterisk (*).

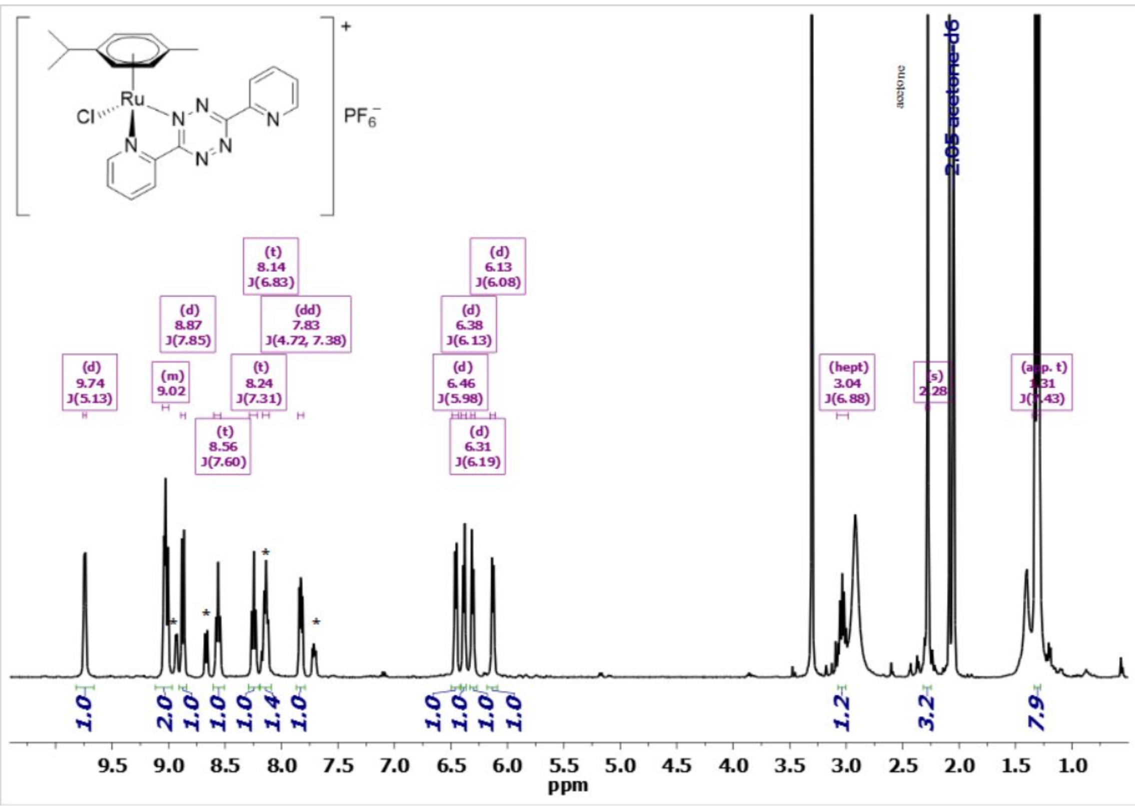

Figure S11. Solid-state IR spectrum (650-4000 cm^{-1}) of [RuCl{κ^2*N*-3-(2-pyridyl)-1,2,4,5-tetrazine}(η^6-C$_6$Me$_6$)]PF$_6$, [**1d**]PF$_6$.

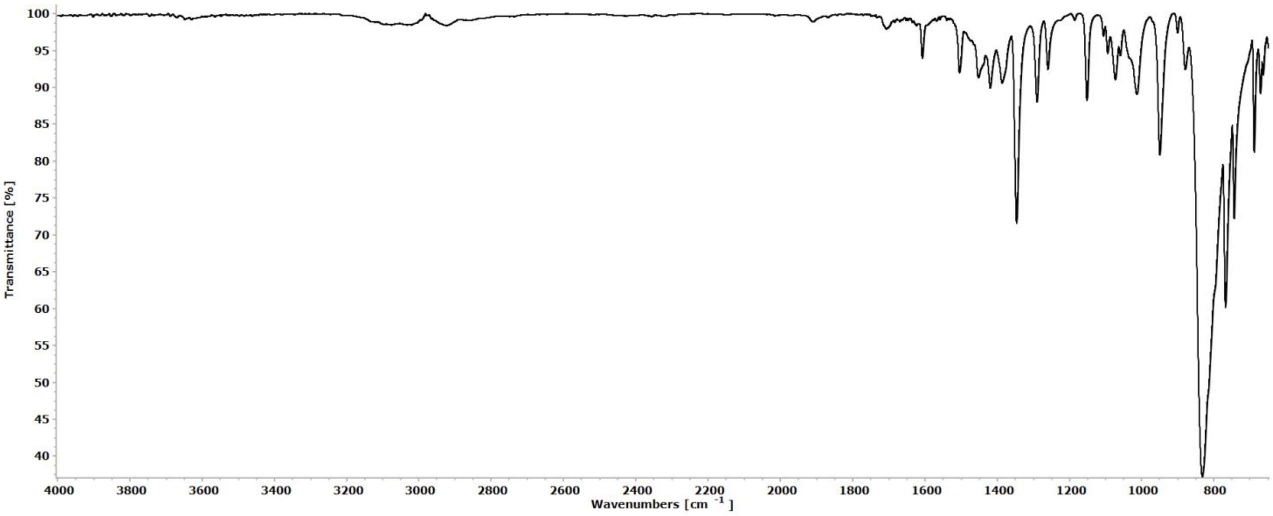

Figure S12. ^{1}H NMR spectrum (400 MHz, acetone-d_6) of [**1d**]PF_6.

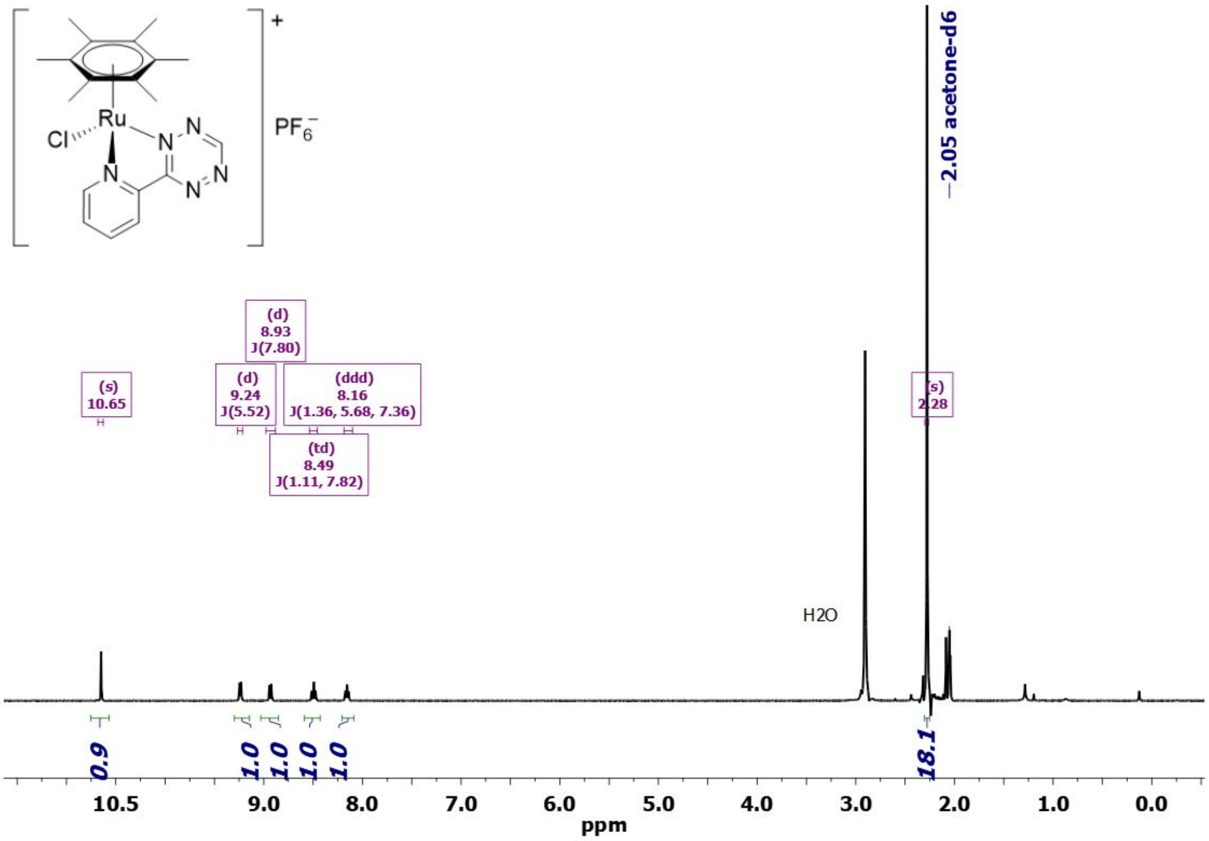

Figure S13. $^{13}C\{^{1}H\}$ NMR spectrum (101 MHz, acetone-d_6) of [**1d**]PF_6.

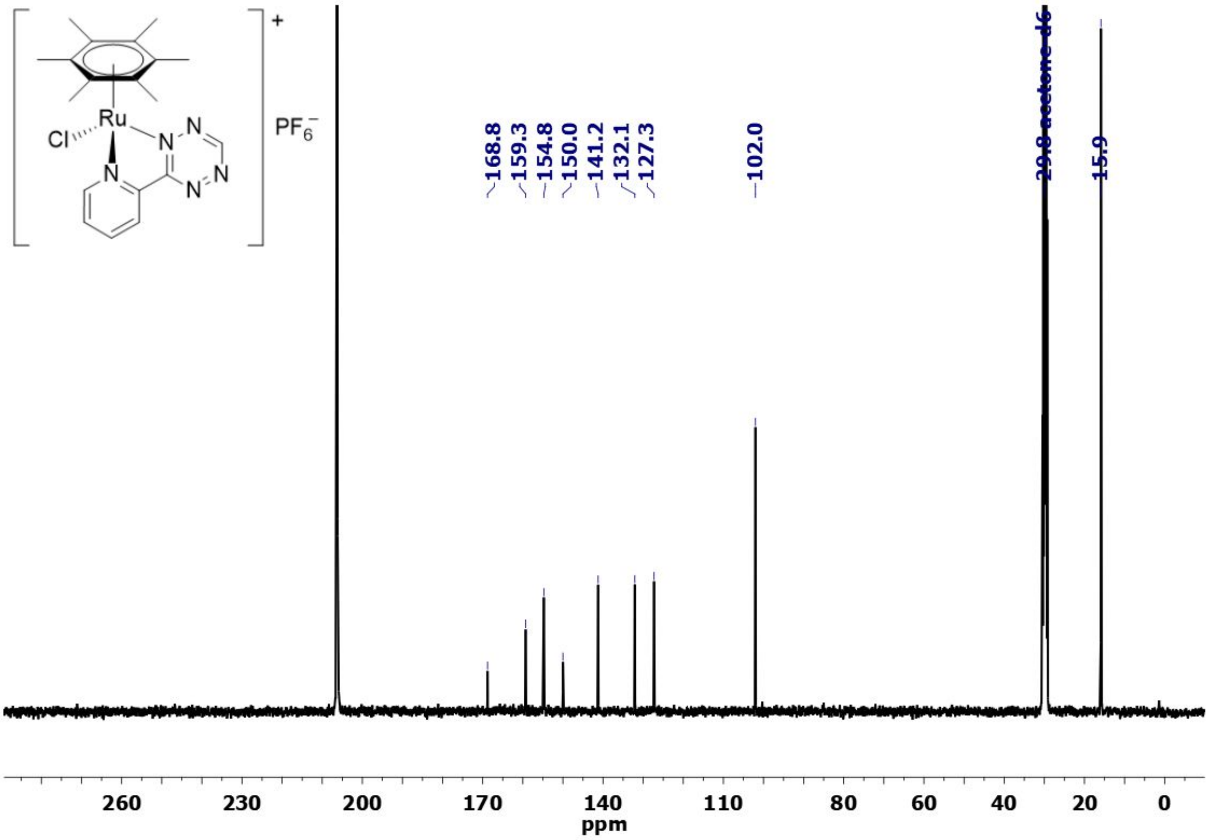

Figure S14. Solid-state IR spectrum (650-4000 cm^{-1}) of [RuCl{ κ^2*N*-3-(2-pyridyl)-6-methyl-1,2,4,5-tetrazine}(η^6-C_6Me_6)]PF_6, [**1e**]PF_6.

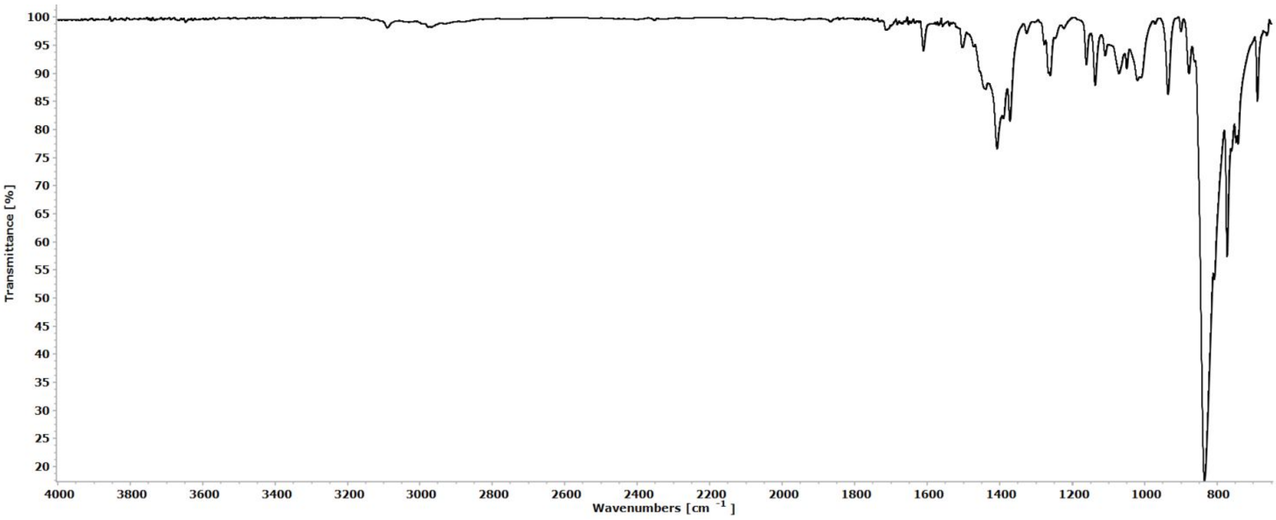

Figure S15. ^{1}H NMR spectrum (400 MHz, acetone-d$_6$) of [**1e**]PF_6.

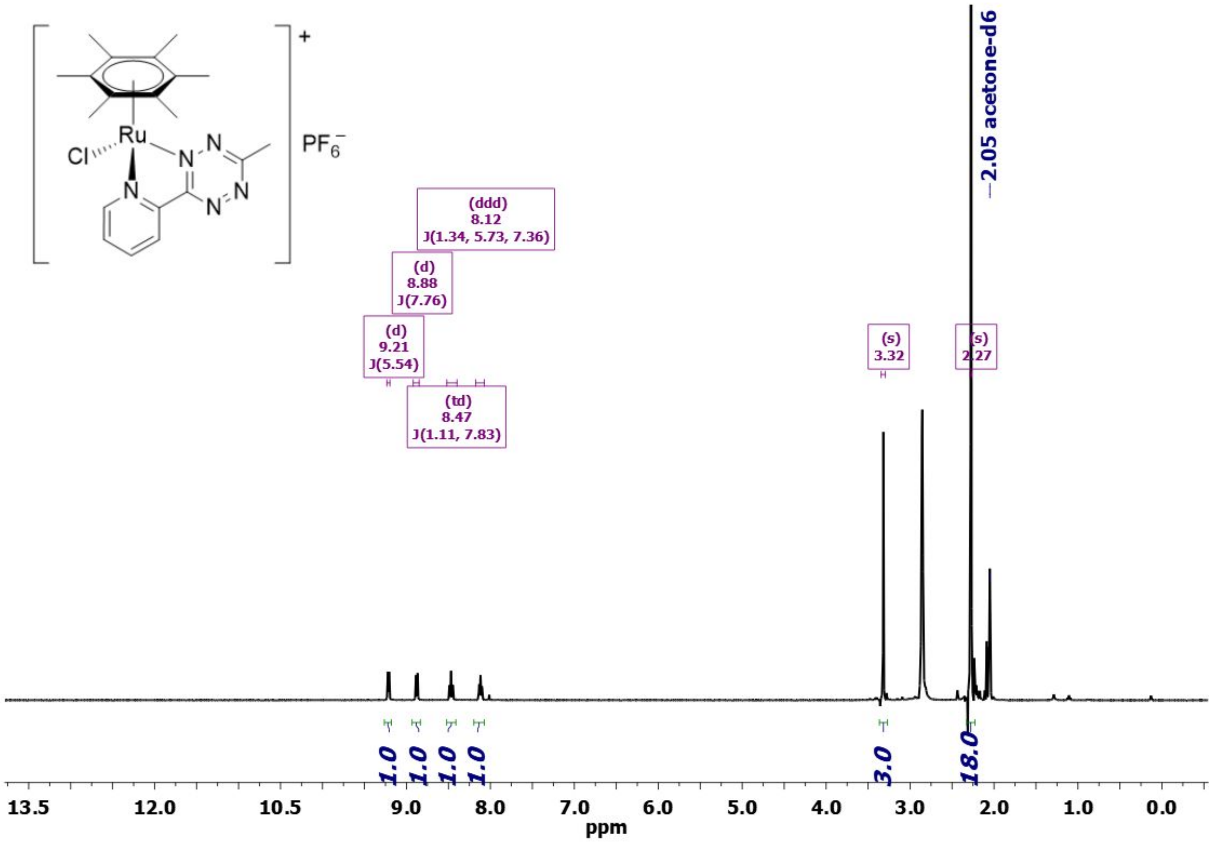

Figure S16. $^{13}C\{^1H\}$ NMR spectrum (101 MHz, acetone-d$_6$) of [**1e**]PF_6.

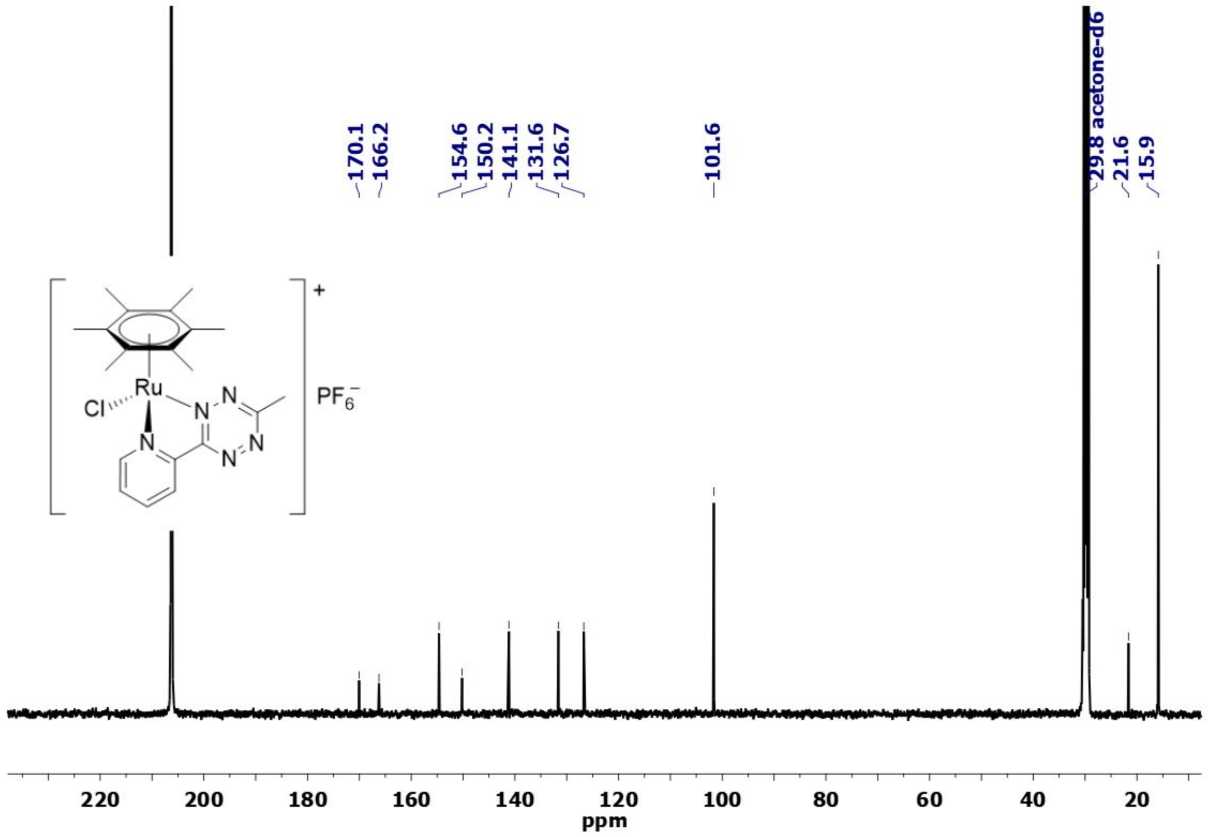

IR, NMR and ESI-MS spectra of pyridyl-triazine-thione complexes

Figure S17. Solid-state IR spectrum (650-4000 cm^{-1}) of [RuCl{κ^2*N*-6-(2-pyridyl)-5-thioxo-1,2,4-triazinide}(η^6-*p*-cymene)], **2a**.

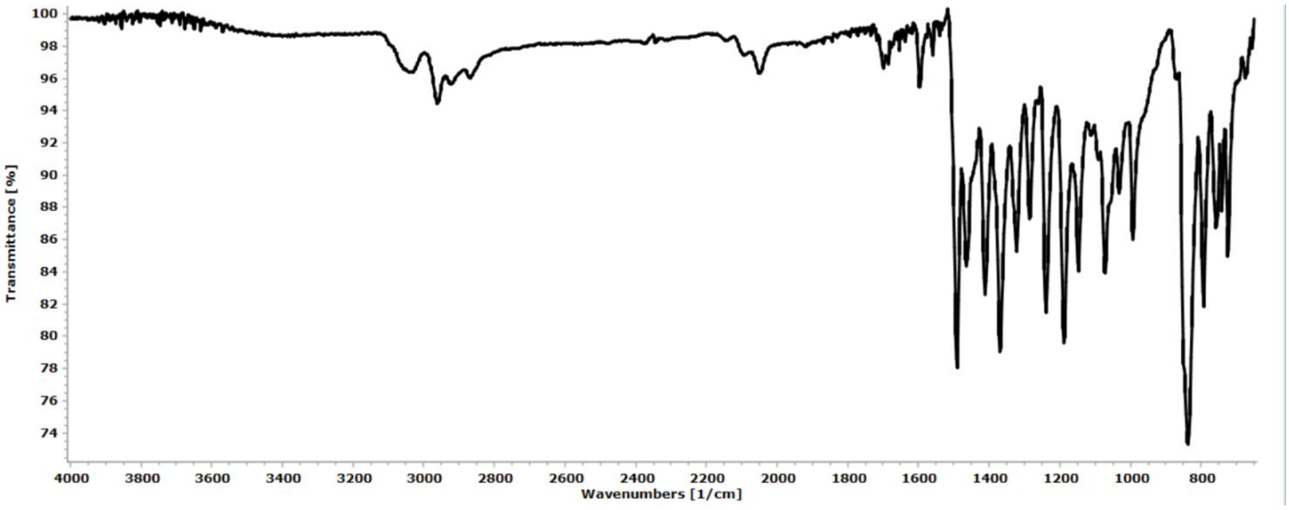

Figure S18. ^{1}H NMR spectrum (500 MHz, acetone-d$_6$) of **2a**.

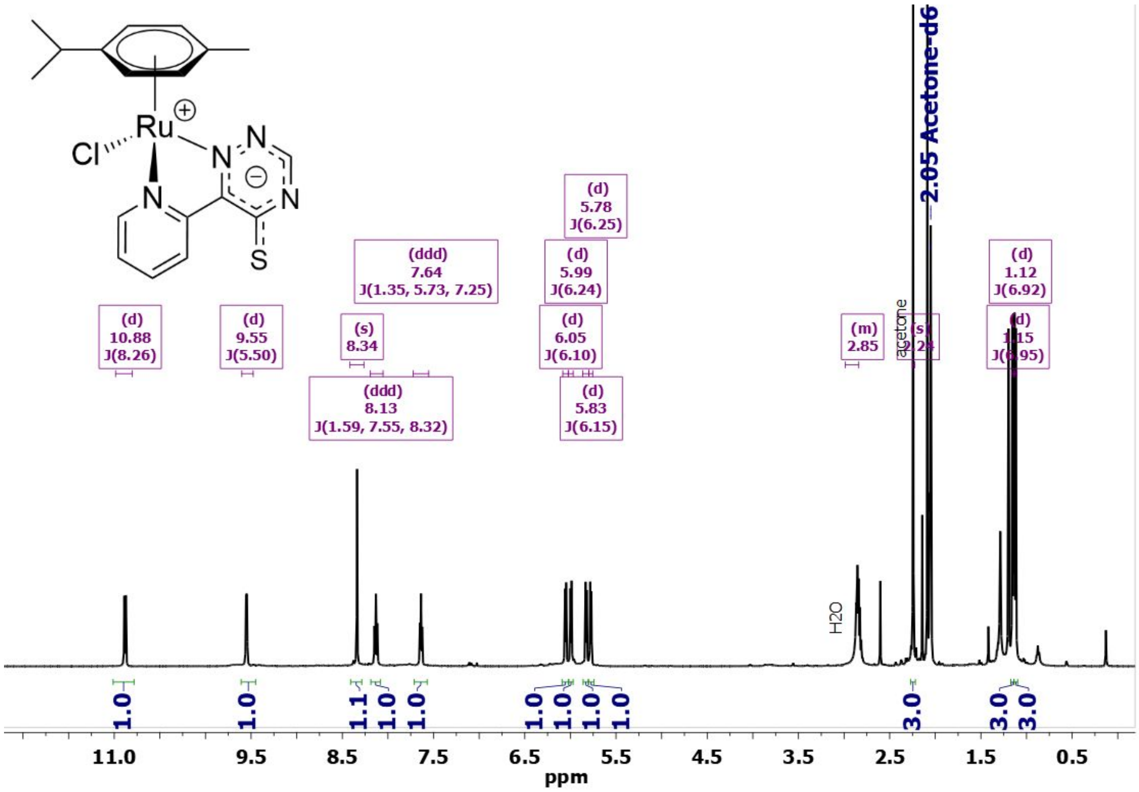

Figure S19. $^{13}C\{^{1}H\}$ NMR spectrum (126 MHz, acetone-d_6) of **2a**.

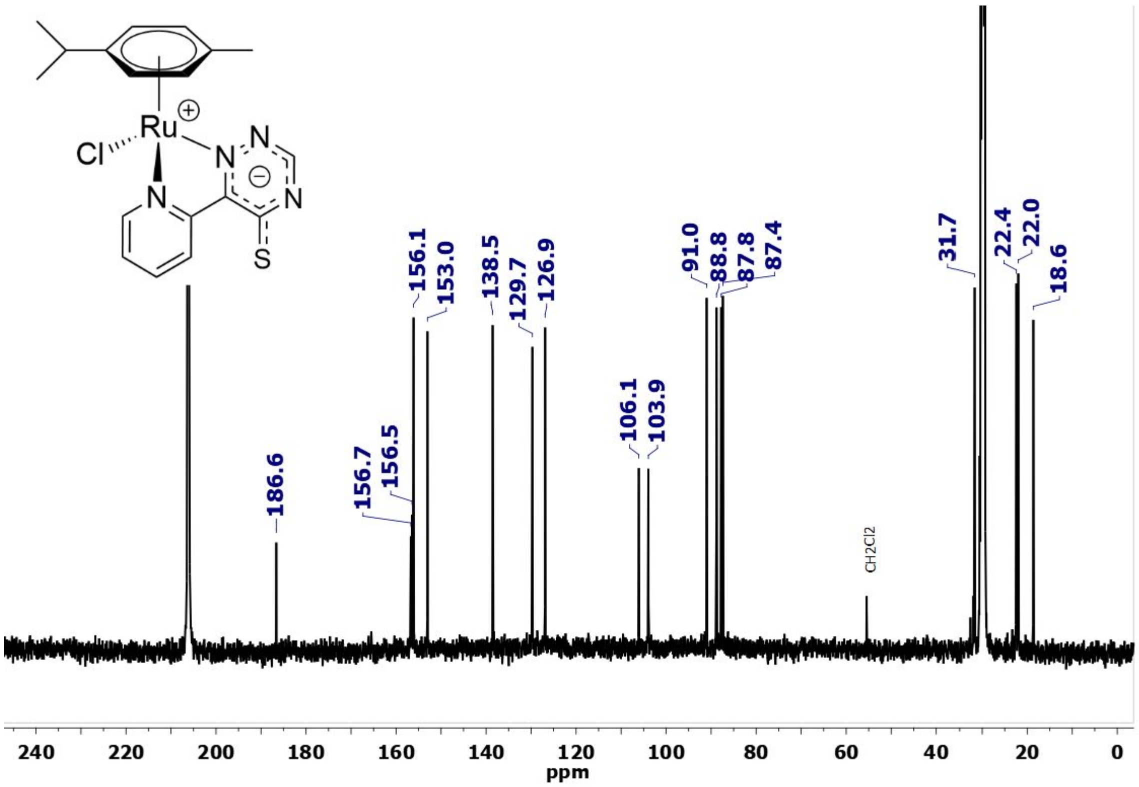

Figure S20. Section of the ^{1}H-^{13}C HSQC NMR spectrum (acetone-d_6) of **2a**, related to triazine and *p*-cymene resonances.

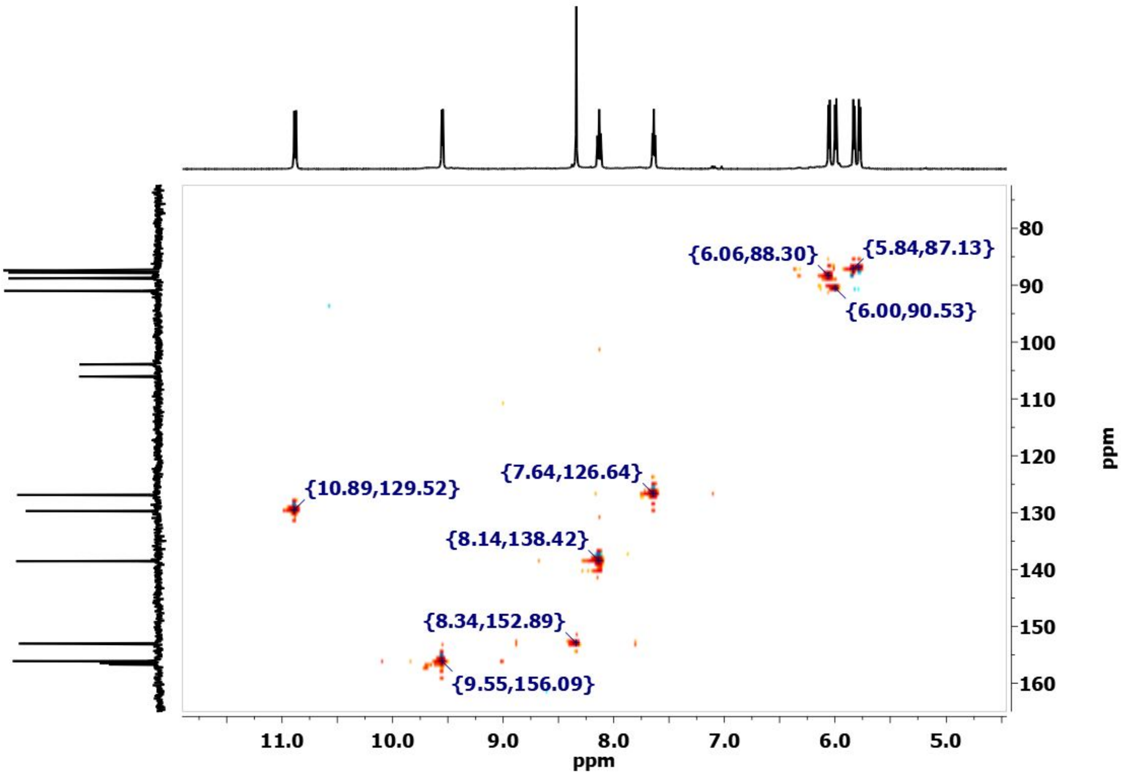

Figure S21. Section of the ^{1}H-^{13}C HMBC NMR spectrum (acetone-d$_6$; long range J = 8 Hz, Δ_2 = 62.5 ms) of **2a**, related to triazine resonances, showing the correlation between the triazinyl proton (8.34 ppm) and the CS carbon (186.4 ppm).

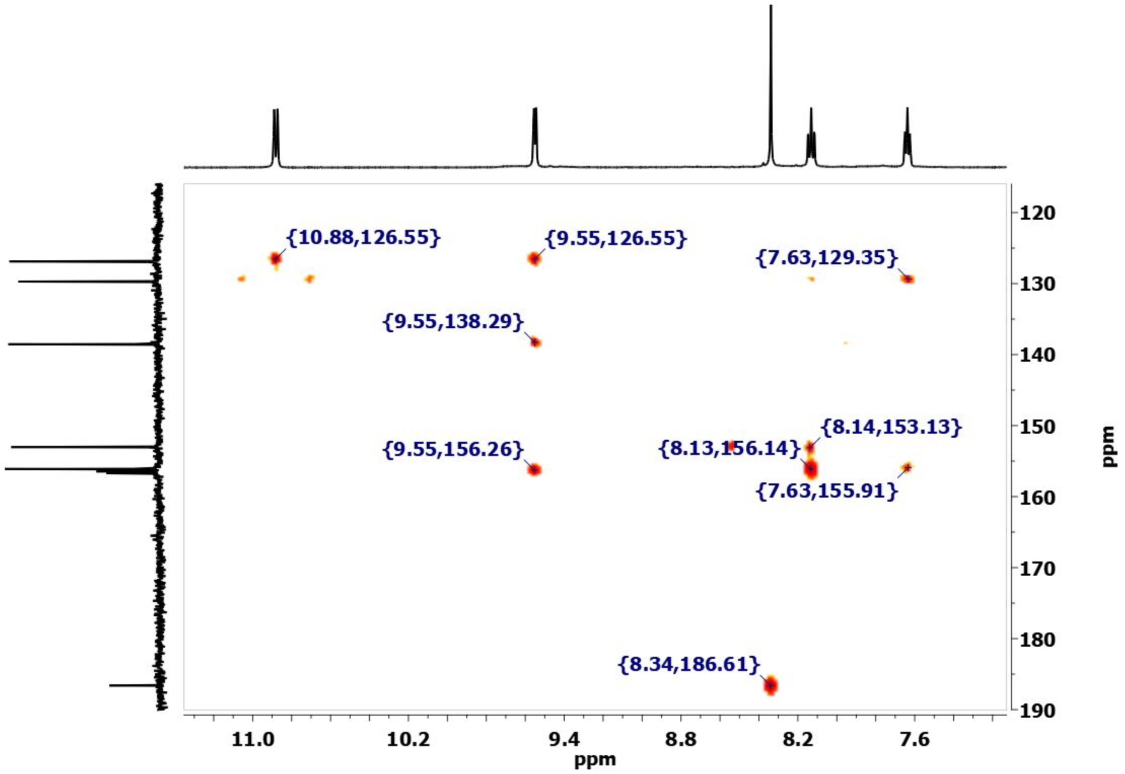

Figure S22. FIA-ESI(+)-MS spectrum of **2a** in MeCN. Calcd. base peak for [**2a**+H]$^+$ ($C_{18}H_{20}ClN_4RuS$): 461.0168 Da.

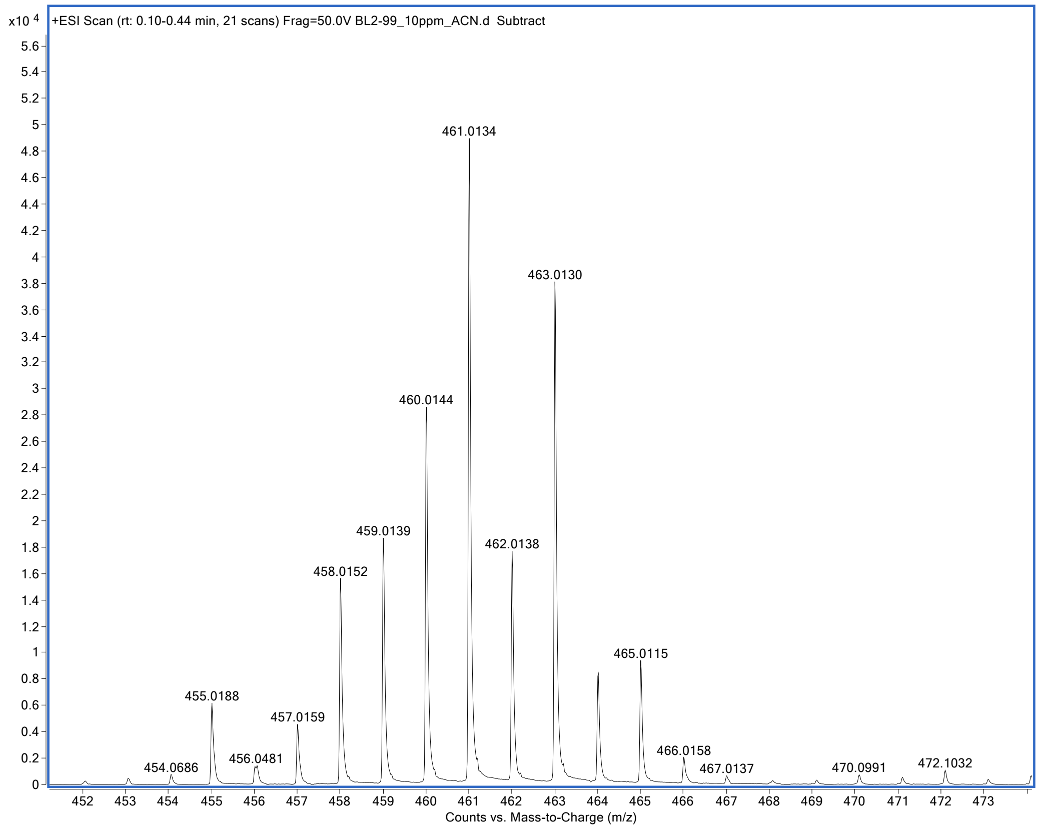

Figure S23. Solid-state IR spectrum (650-4000 cm^{-1}) of [RuCl{κ^2*N*-3-methyl-6-(2-pyridyl)-5-thioxo-1,2,4-triazinide}(η^6-*p*-cymene)], **2b**.

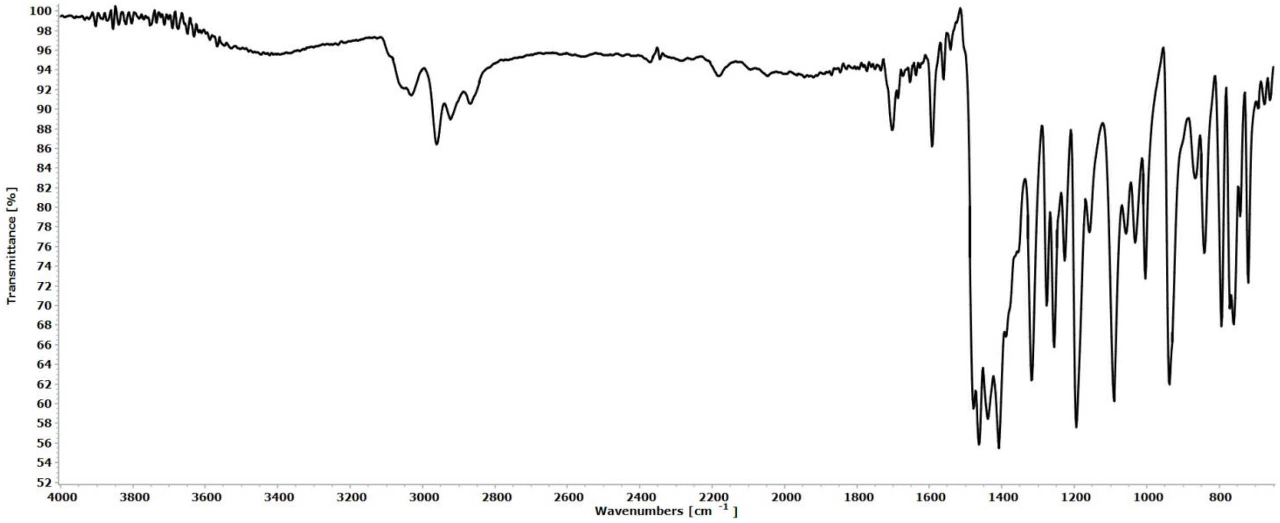

Figure S24. ^{1}H NMR spectrum (500 MHz, acetone-d$_6$) of **2b**.

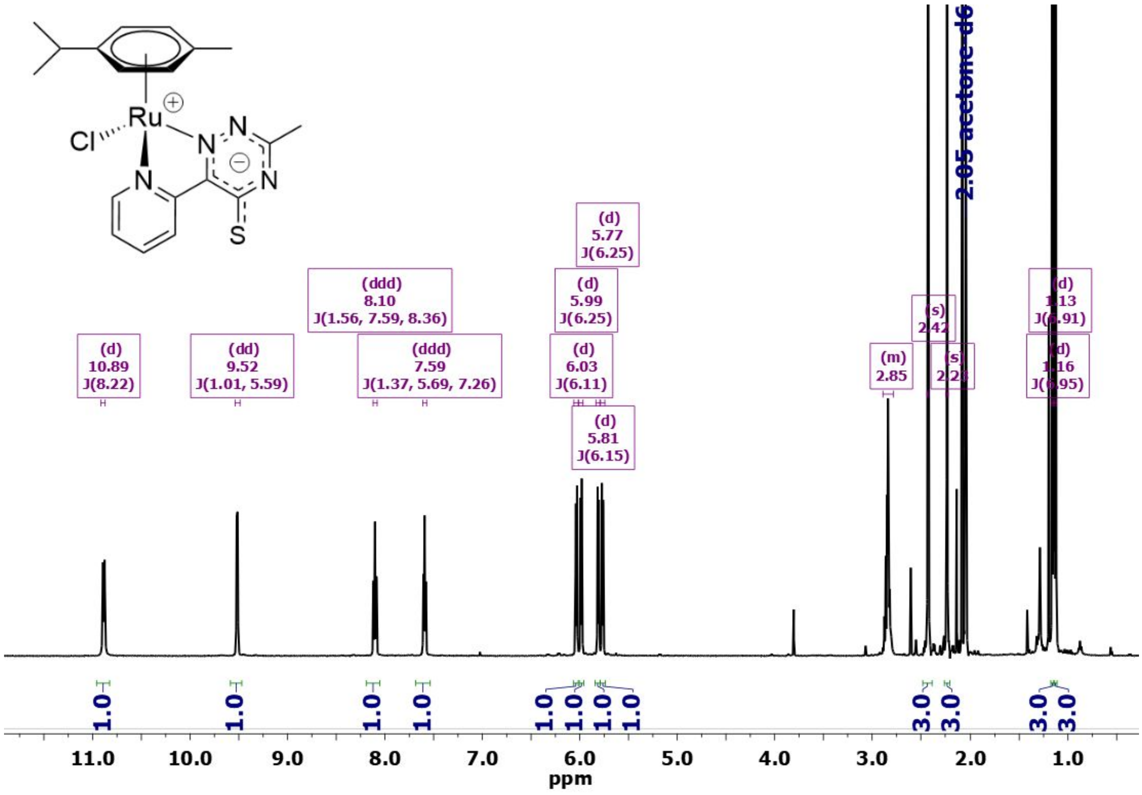

Figure S25. $^{13}C\{^{1}H\}$ NMR spectrum (126 MHz, acetone-d_6) of **2b**.

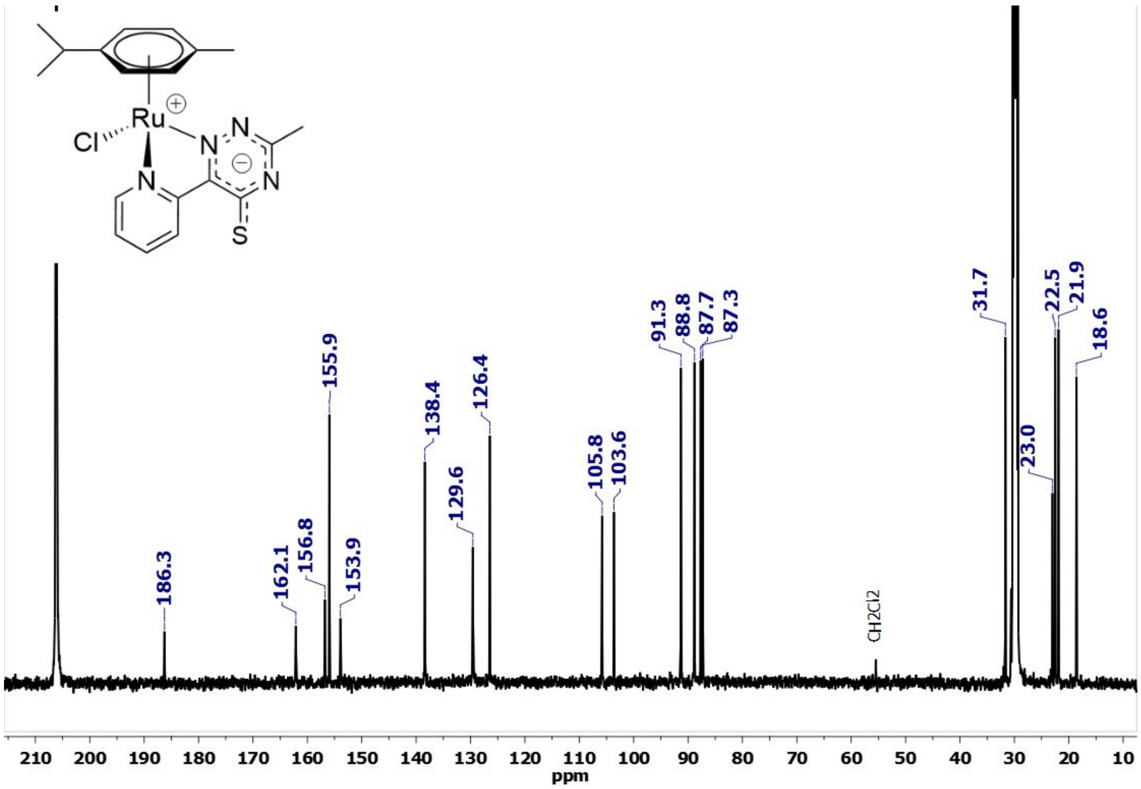

Figure S26. Section of the ^{1}H-^{13}C HSQC NMR spectrum (acetone-d_6) of **2b**, related to triazine and *p*-cymene resonances.

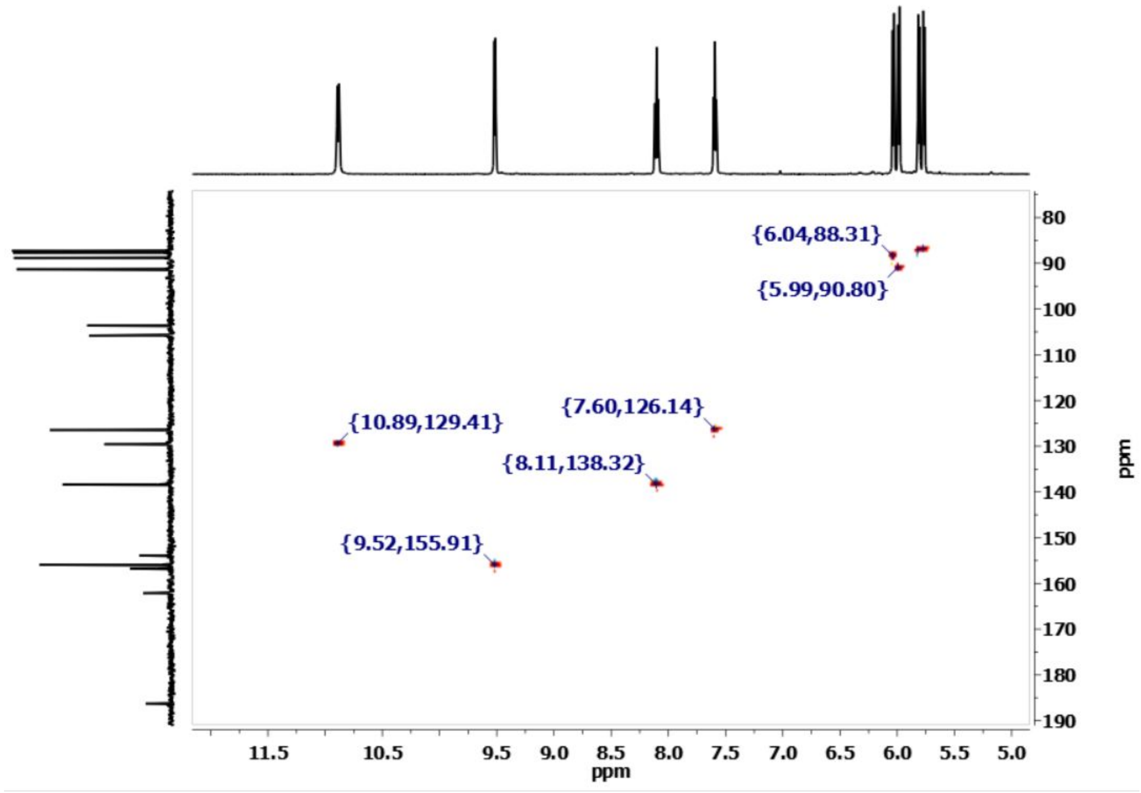

Figure S27. Section of the ^{1}H-^{13}C HMBC NMR spectrum (acetone-d_6; long range J = 8 Hz, Δ_2 = 62.5 ms) of **2b**, related to triazine resonances, showing no correlation between the triazinyl methyl (2.42 ppm) and the CS carbon (186.3 ppm).

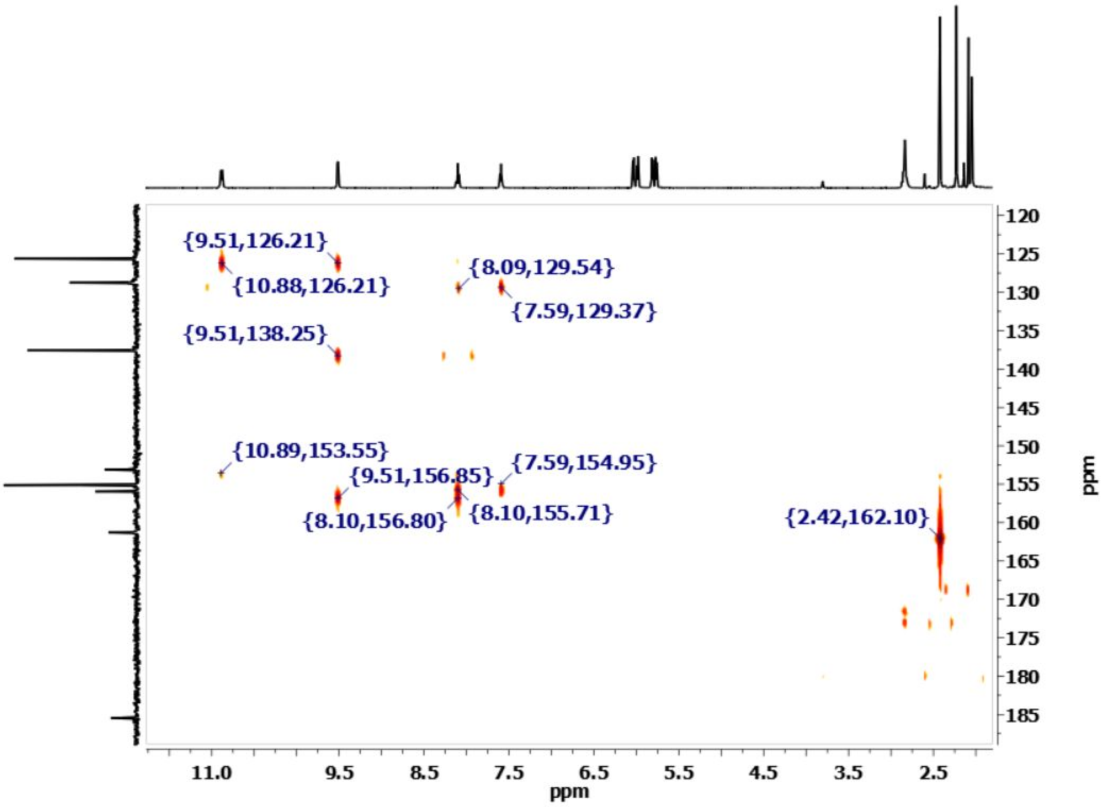

Figure S28. FIA-ESI(+)-MS spectrum of **2b** in MeCN. Calcd. base peak for [**2b**+H]$^{+}$ ($C_{19}H_{22}ClN_4RuS$): 475.0324 Da.

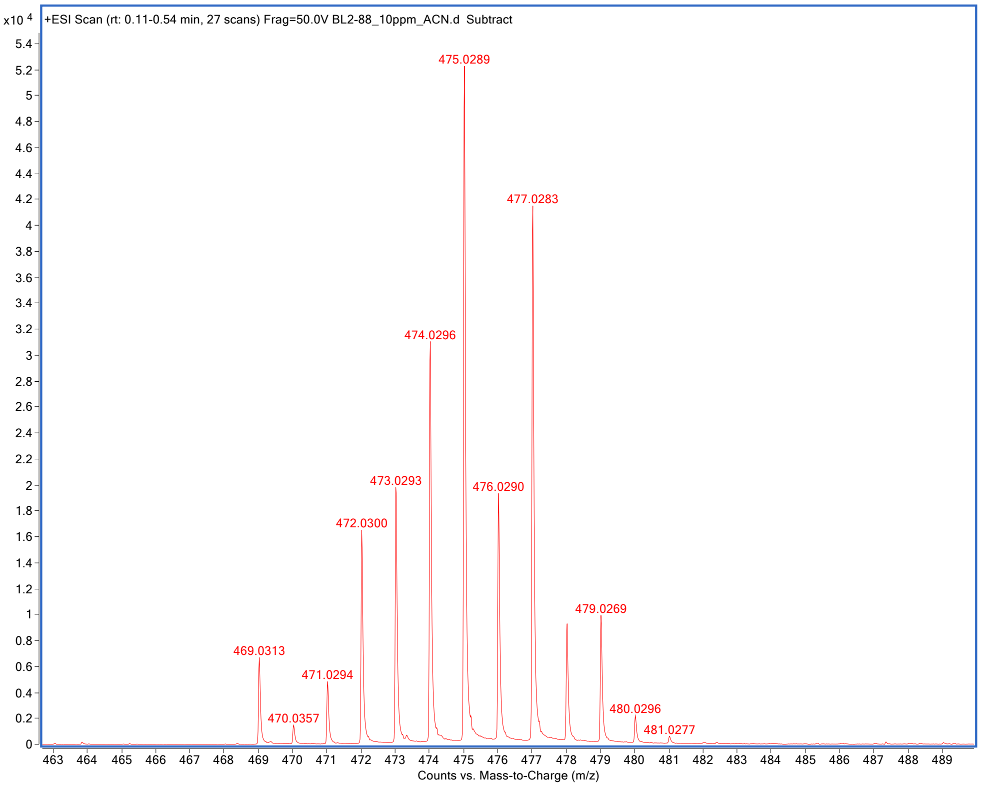

Figure S29. ^{1}H NMR spectrum (400 MHz, acetone-d_6) of [RuCl{κ^2*N*-3,6-di-(2-pyridyl)-5-thioxo-1,2,4-triazinide}(η^6-*p*-cymene)], **2c** in admixture with $[Bu_4N]PF_6$. Signals due to BHT (nonvolatile radical inhibitor of commercial THF) are marked with asterisk.

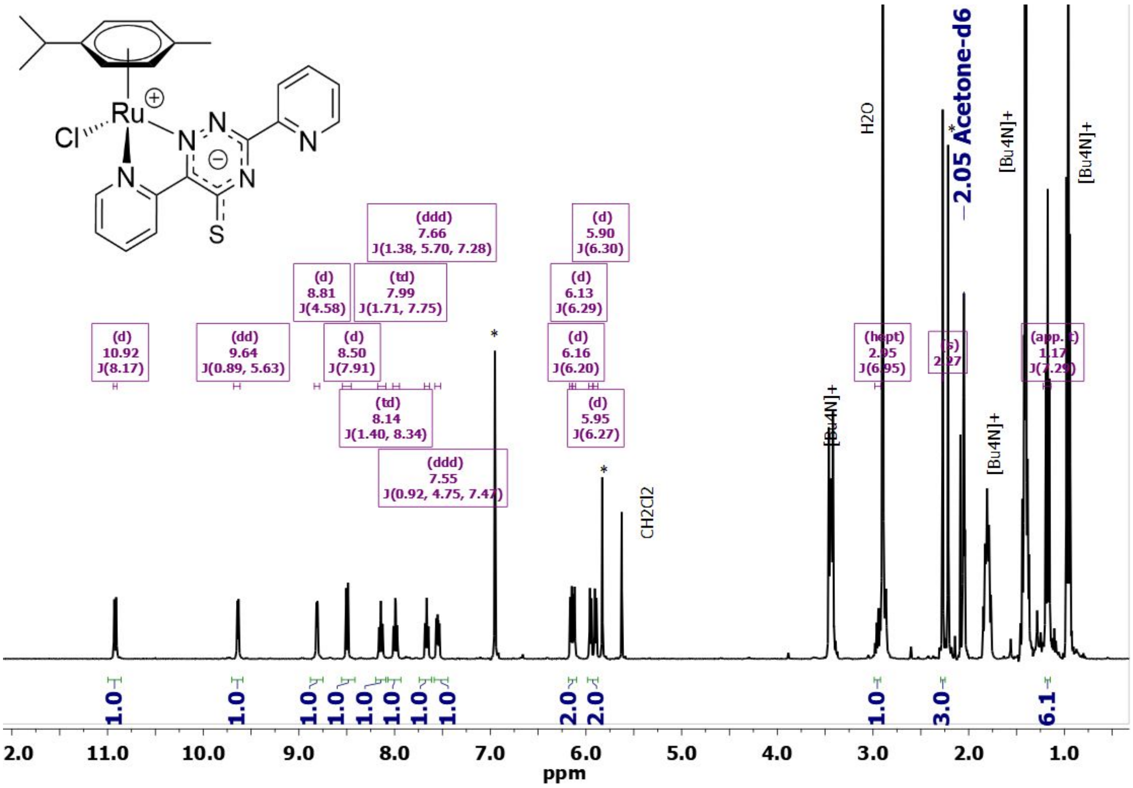

Figure S30. $^{13}C\{^1H\}$ NMR spectrum (100 MHz, acetone-d_6) of **2c** in admixture with $[Bu_4N]PF_6$. Signals due to BHT are marked with asterisk.

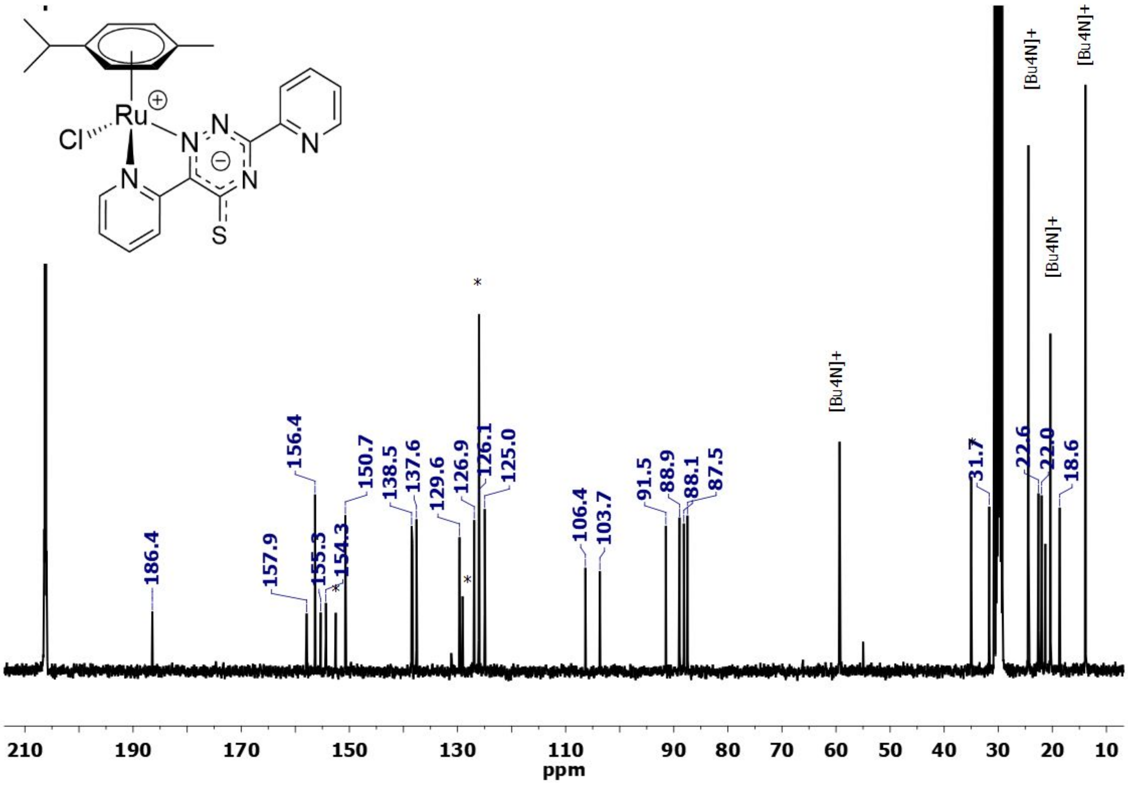

Figure S31. FIA-ESI(+)-MS spectrum of **2c** in MeCN. Calcd. base peak for [**2c**+H]$^+$ ($C_{23}H_{23}ClN_5RuS$): 538.0433 Da.

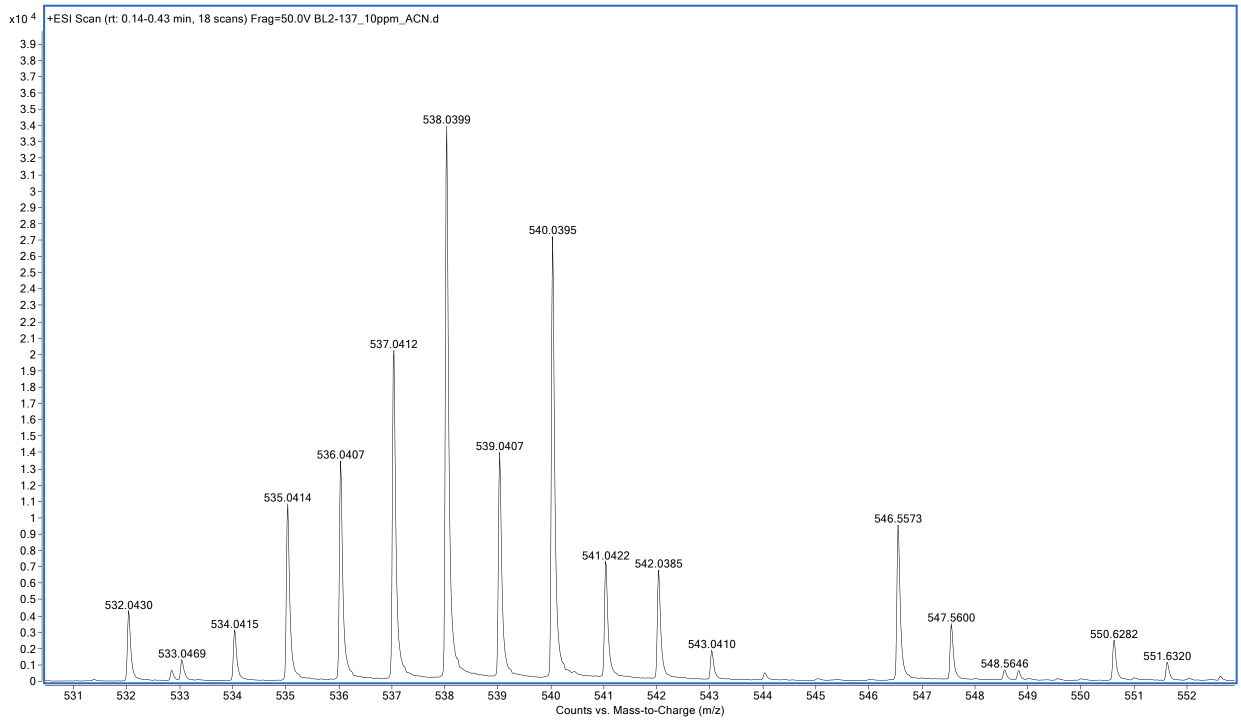

Figure S32. ^{1}H NMR spectrum (500 MHz, acetone-d$_6$) of [RuCl{κ^2*N*-6-(2-pyridyl)-5-thioxo-1,2,4-triazinide}(η^6-C$_6$Me$_6$)], **2d**.

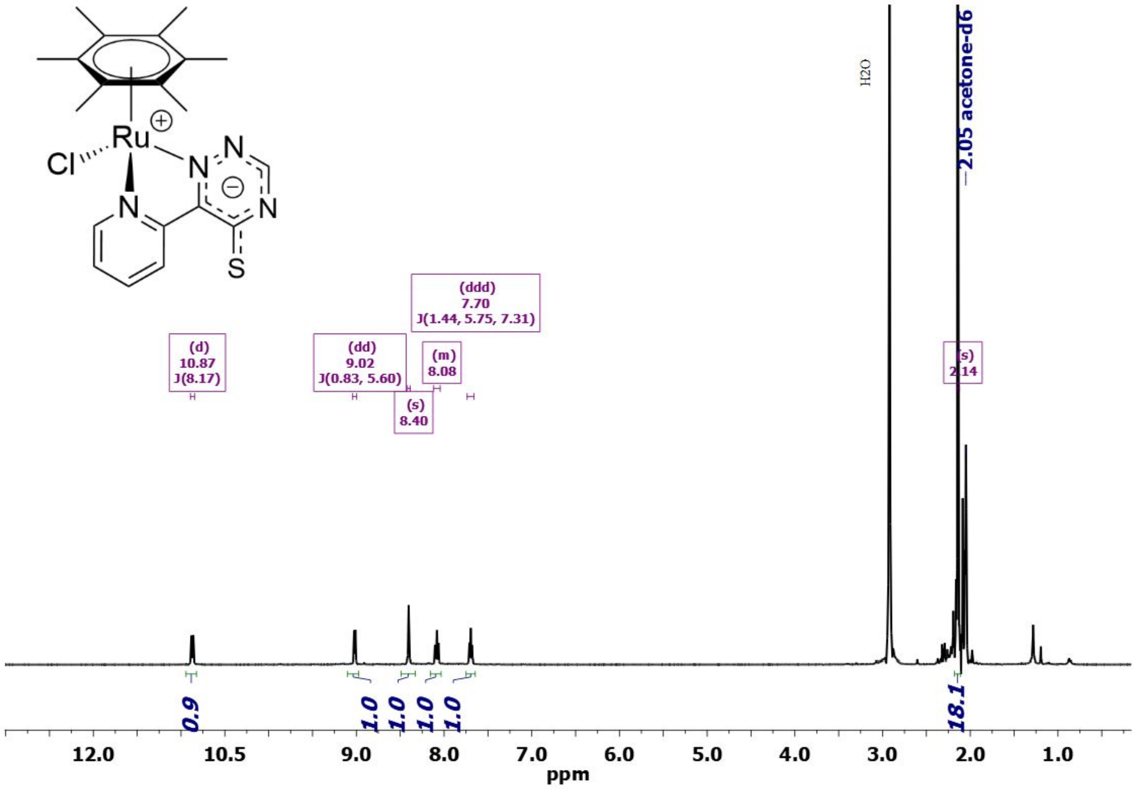

Figure S33. $^{13}C\{^{1}H\}$ NMR spectrum (126 MHz, acetone-d$_6$) of **2d**.

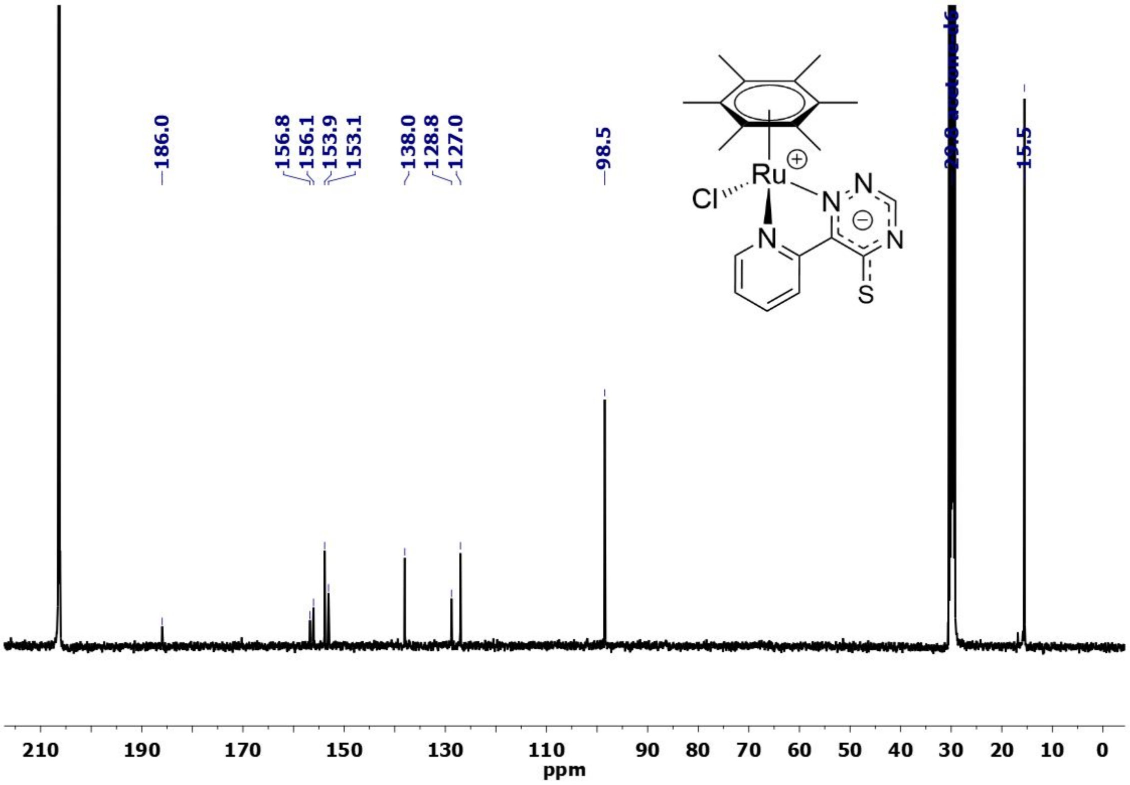

Figure S34. Section of the ^{1}H-^{13}C HMBC NMR spectrum (acetone-d$_6$; long range J = 8 Hz, Δ_2 = 62.5 ms) of **2d**, related to triazine resonances, showing the correlation between the triazinyl proton (8.40 ppm) and the CS carbon (186.0 ppm).

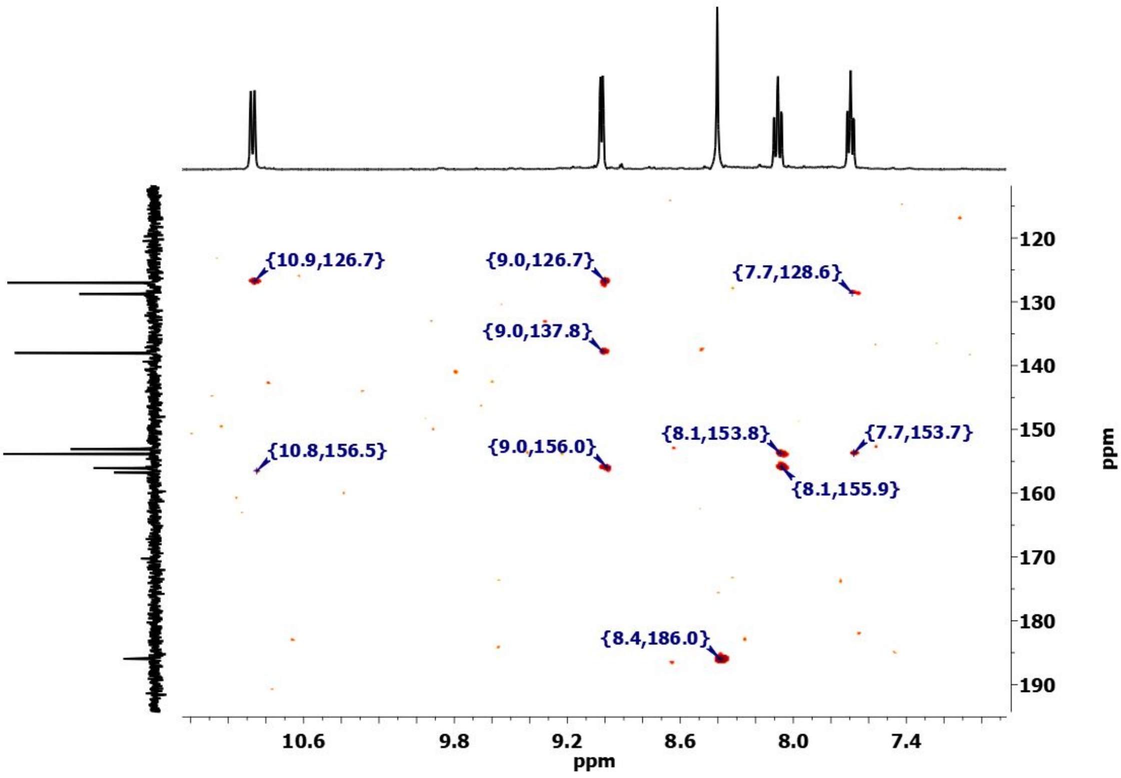

Figure S35. ^{1}H NMR spectrum (400 MHz, acetone-d_6) of [RuCl{κ^2*N*-3-methyl-6-(2-pyridyl)-5-thioxo-1,2,4-triazinide}(η^6-C_6Me_6)], **2e**.

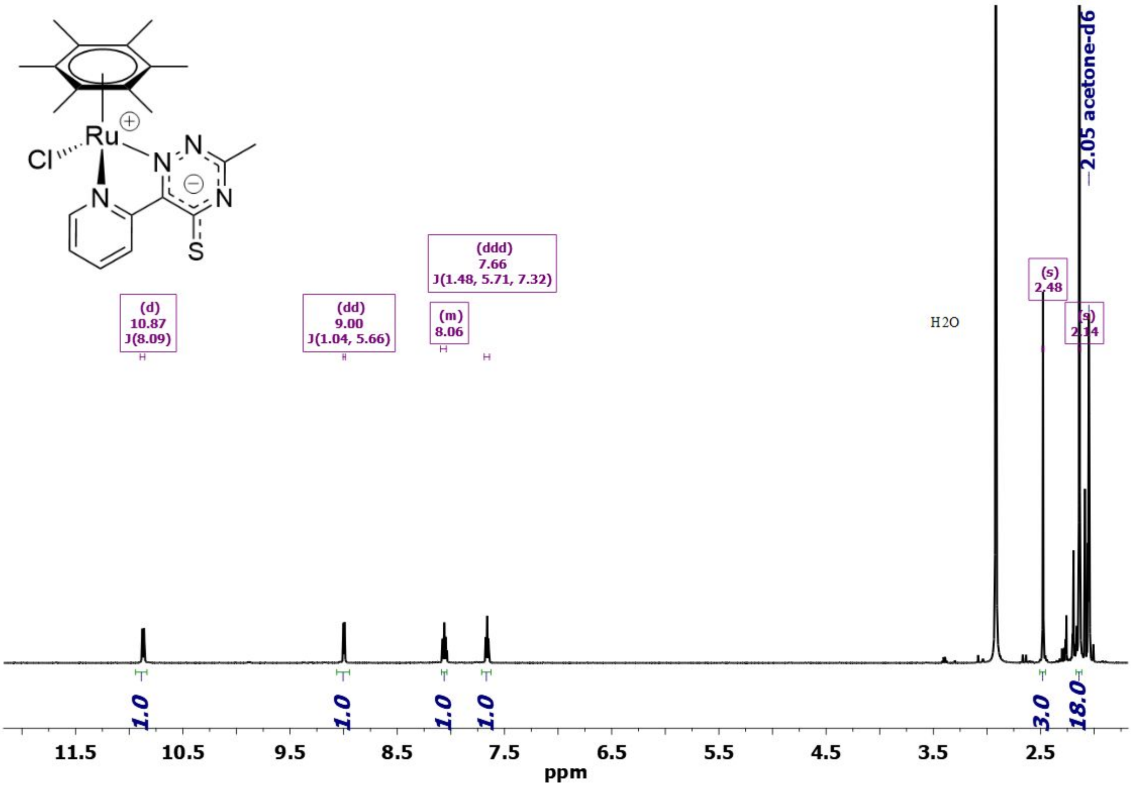

Figure S36. ^{13}C{^{1}H} NMR spectrum (100 MHz, acetone-d_6) of **2e**.

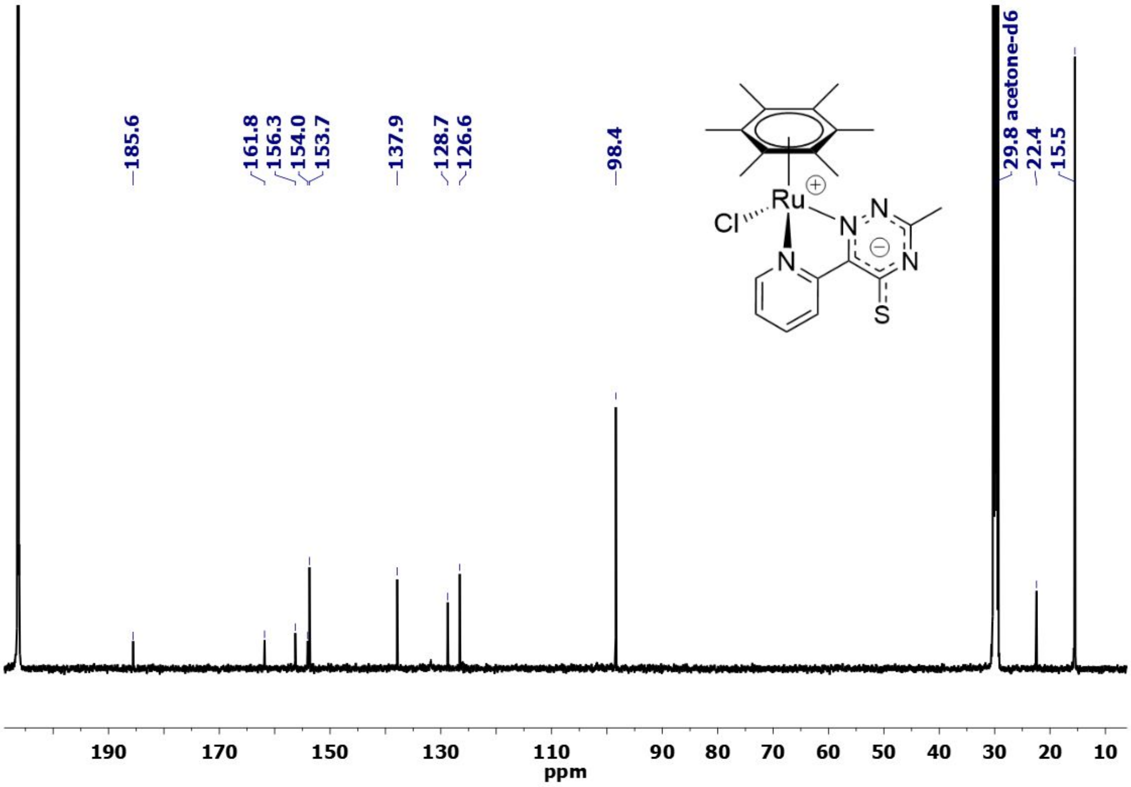

Figure S37. Section of the ^{1}H-^{13}C HSQC NMR spectrum (acetone-d_6) of **2e**, related to triazine resonances.

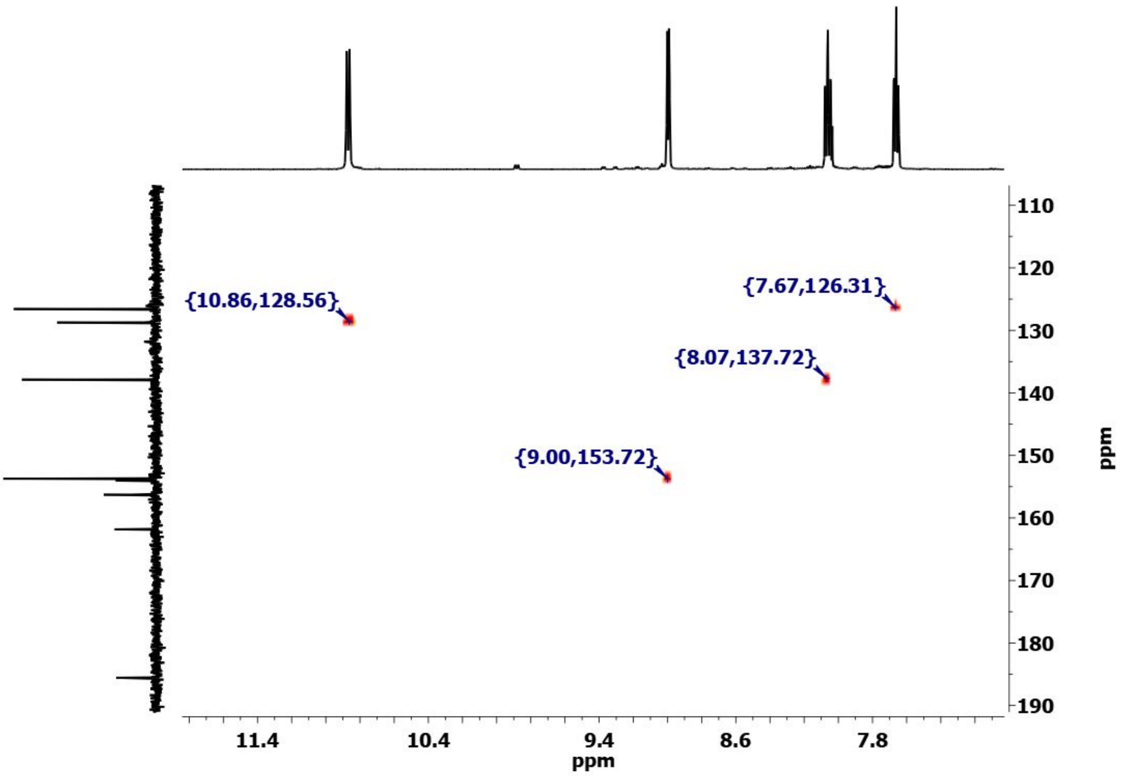

Figure S38. Section of the ^{1}H-^{13}C HMBC NMR spectrum (acetone-d_6; long range J = 8 Hz, Δ_2 = 62.5 ms) of **2e**, related to triazine resonances, showing no correlation between the triazinyl methyl (2.48 ppm) and the CS carbon (185.6 ppm).

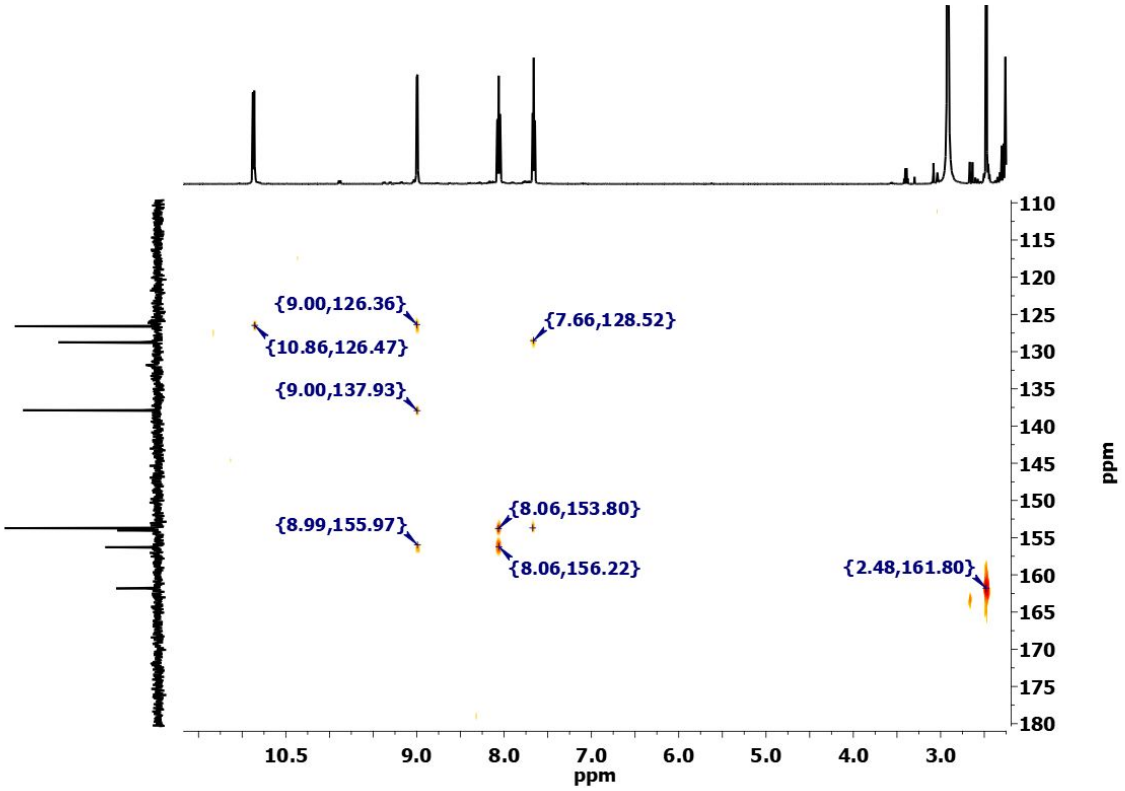

Figure S39. Solid-state IR spectrum (650-4000 cm^{-1}) of [**2bH**]PF_6.

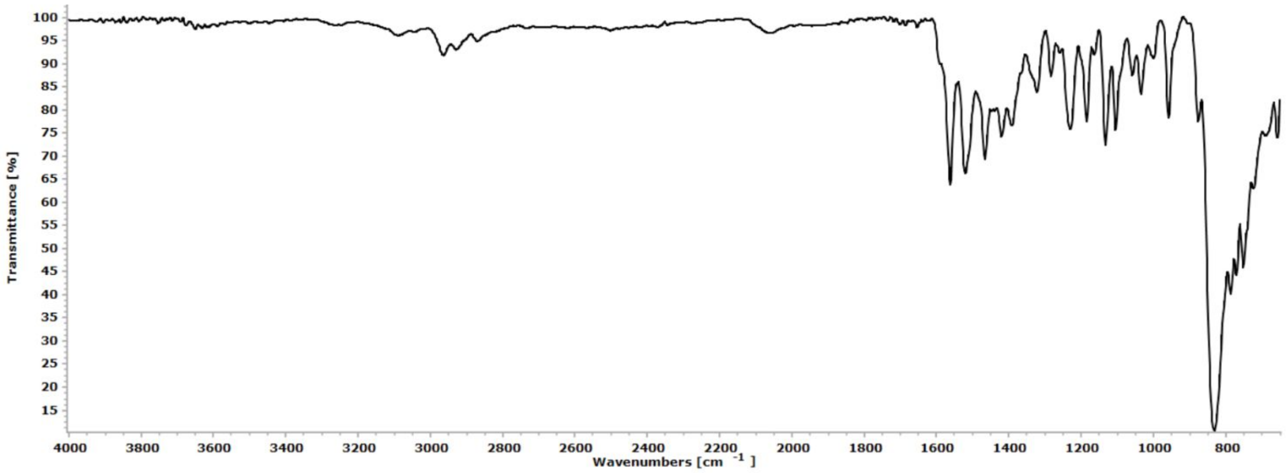

Figure S40. ^{1}H NMR spectrum (500 MHz, acetone-d_6) of [**2bH**]PF_6.

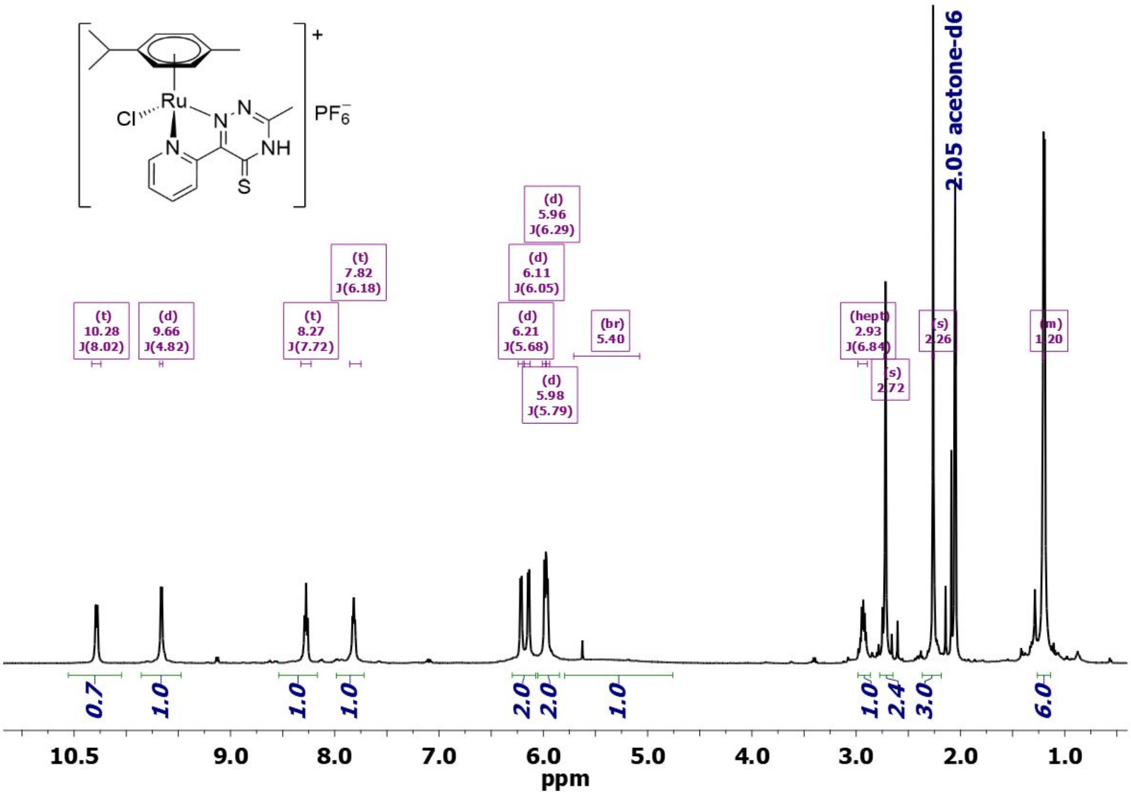

Figure S41. ^{1}H NMR spectrum (400 MHz, $CDCl_3$) of [**2bH**]PF_6 acquired with a longer delay time, showing the correct integral values for all resonances.

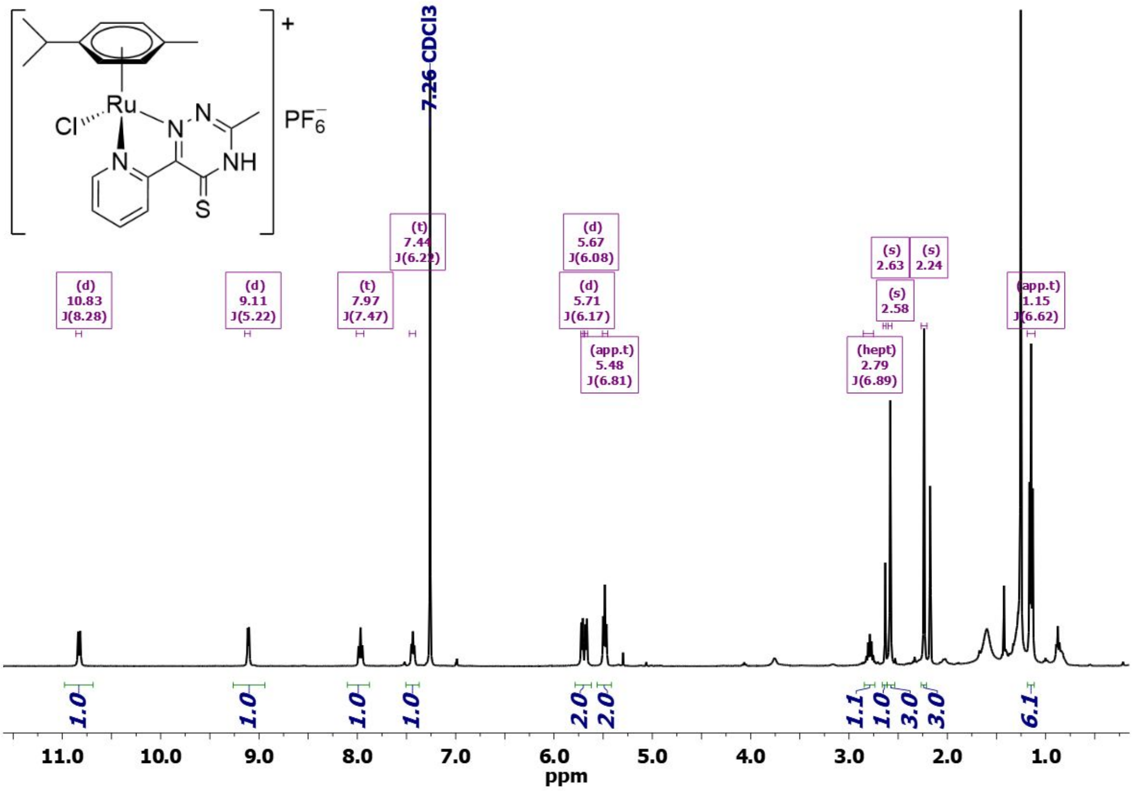

Figure S42. ^{1}H NMR spectra (400 MHz, acetone-d_6; 6-10 ppm region) of [**2bH**]$^+$ as PF_6^- (blue line), *p*-$CH_3C_6H_4SO_3^-$ (green line) or $CF_3SO_3^-$ (dark red line) salt.

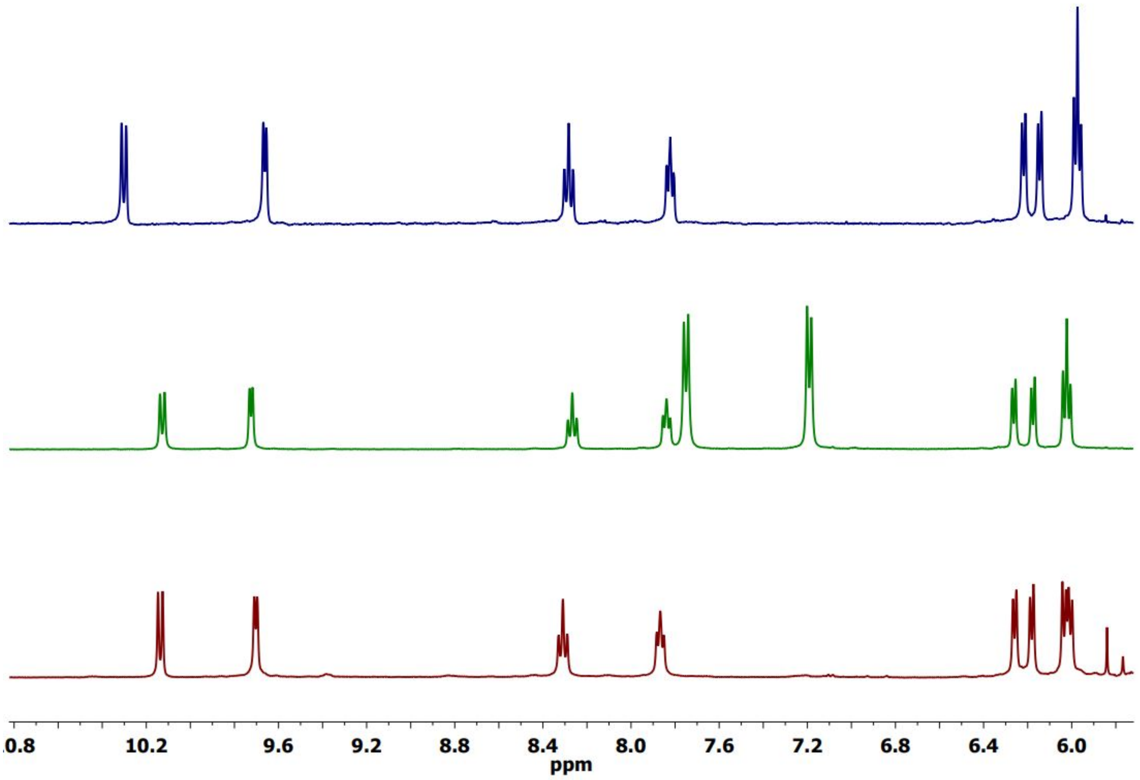

Figure S43. $^{13}C\{^1H\}$ NMR spectrum (126 MHz, acetone-d_6) of [**2bH**]PF_6.

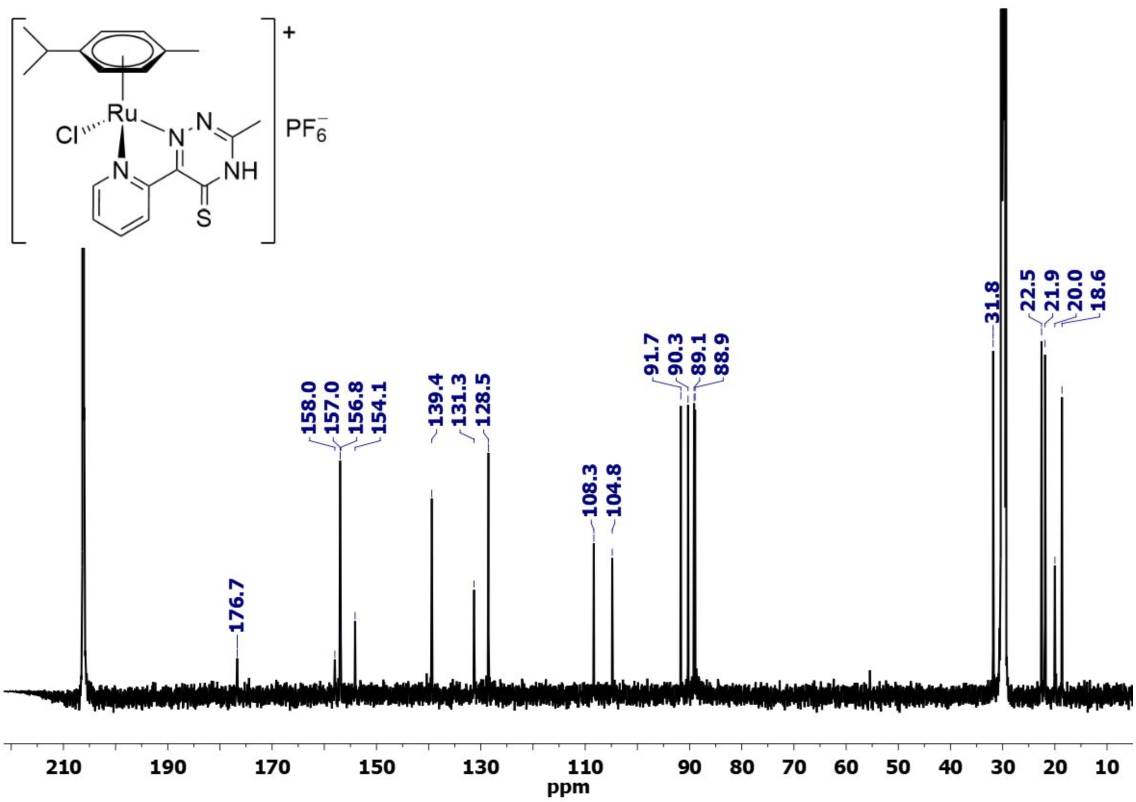

Figure S44. Section of the ^1H-^{13}C HSQC NMR spectrum (acetone-d_6) of [**2bH**]PF_6, related to triazine and *p*-cymene resonances.

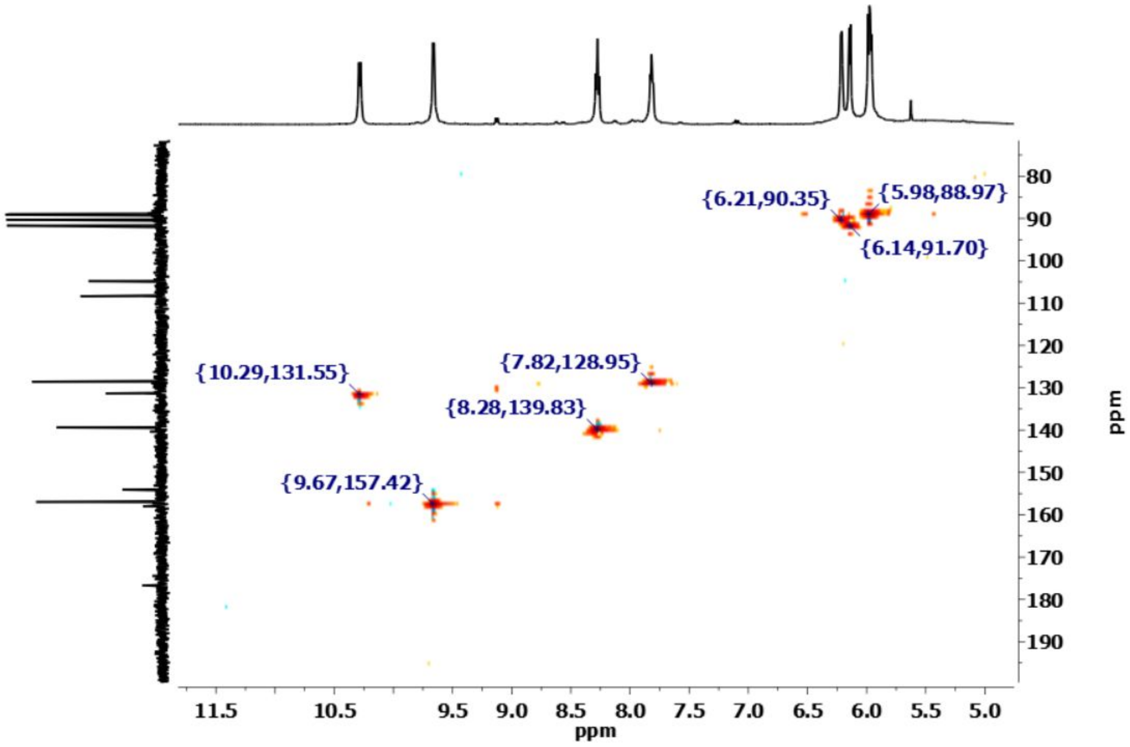

Figure S45. Section of the ^{1}H-^{13}C HMBC NMR spectrum (acetone-d_6; long range J = 8 Hz, Δ_2 = 62.5 ms) of [**2bH**]PF_6, related to triazine resonances.

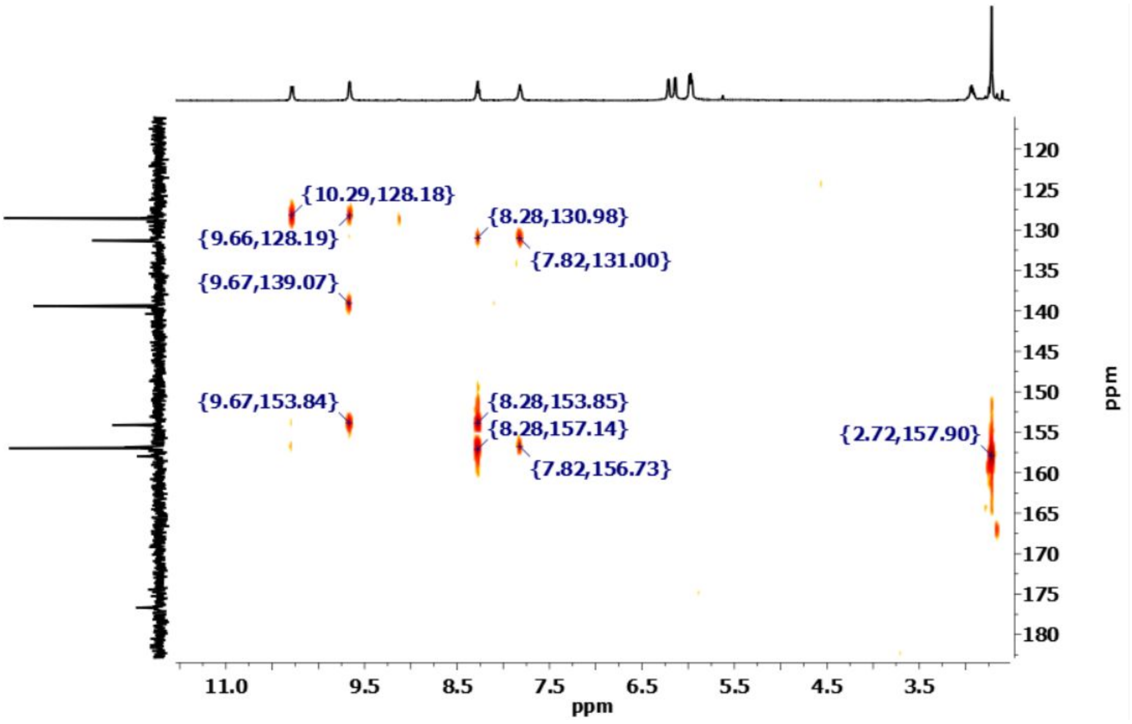

Figure S46. UV-Vis spectra of [**2bH**]PF_6 ($2 \cdot 10^{-4}$ M) in acetone (red line) and after progressive additions of Et_3N (red to blue gradient). The final spectrum (blue line) corresponds to **2b** (≥ 1.0 equivalents of base added).

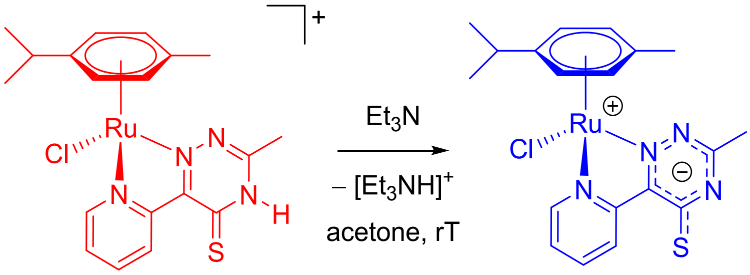

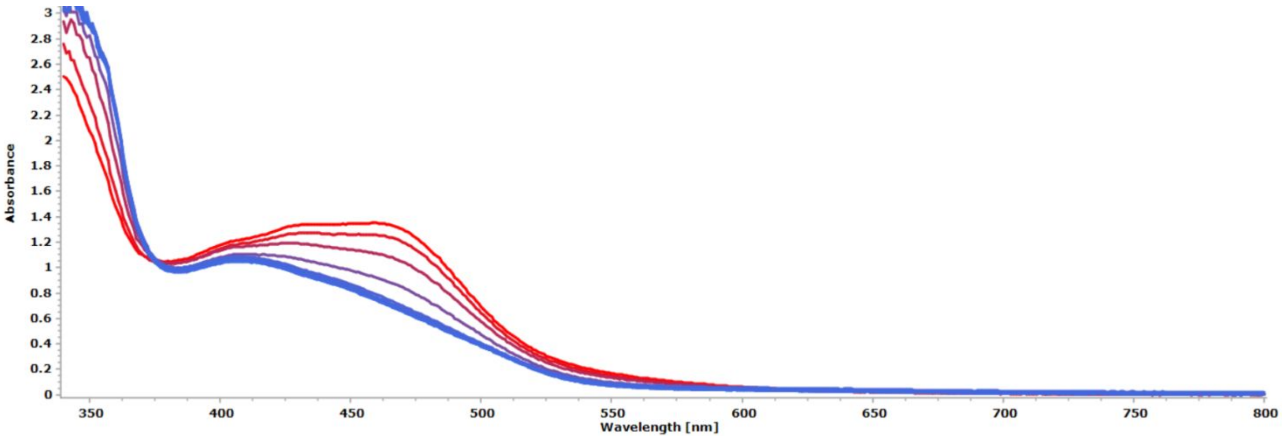

IR, NMR and ESI-MS spectra of pyridyl-triazine-selone complexes

Figure S47. ^{1}H NMR spectrum (500 MHz, acetone-d$_6$) of [RuCl{κ^2*N*-3-methyl-6-(2-pyridyl)-5-selenoxo-1,2,4-triazinide}(η^6-*p*-cymene)], **3b**, in admixture with [Bu$_4$N]PF$_6$, *p*-cymene and other by-products.

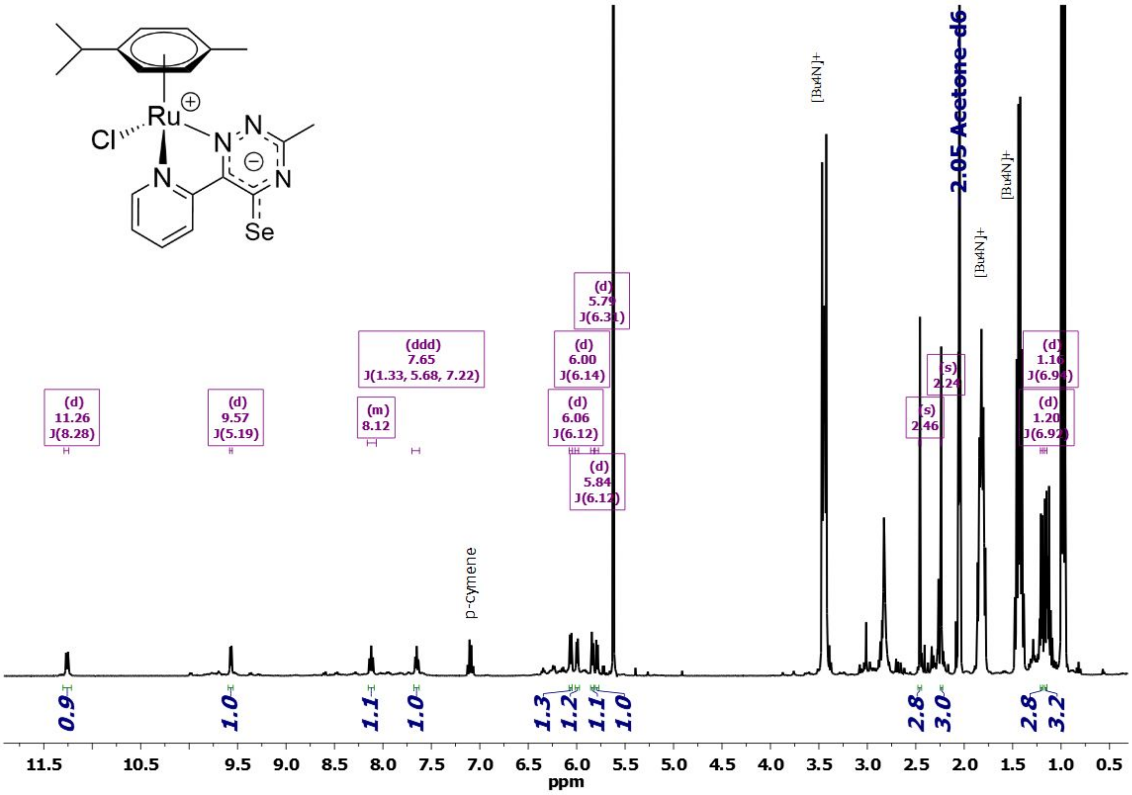

Figure S48. Solid-state IR spectrum (650-4000 cm^{-1}) of [RuCl{κ^2*N*-6-(2-pyridyl)-5-selenoxo-1,2,4-triazinide}(η^6-C$_6$Me$_6$)], **3d**, in admixture with [Bu$_4$N]PF$_6$, showing no bands of Ru-coordinated selenocyanate.

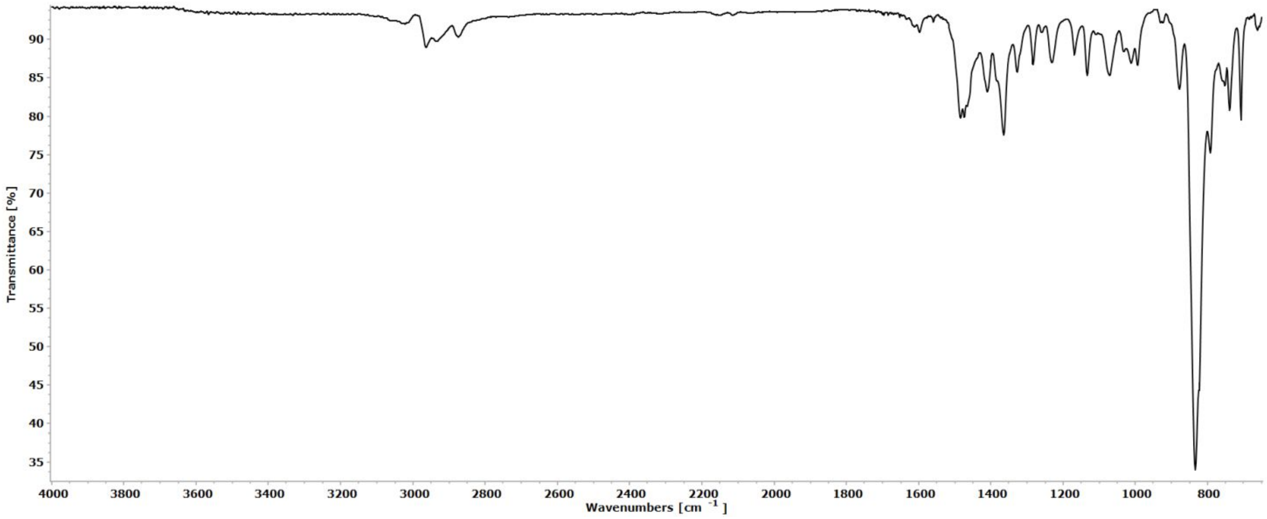

Figure S49. ^{1}H NMR spectrum (500 MHz, $CDCl_3$) of **3d** (in admixture with $[Bu_4N]PF_6$).

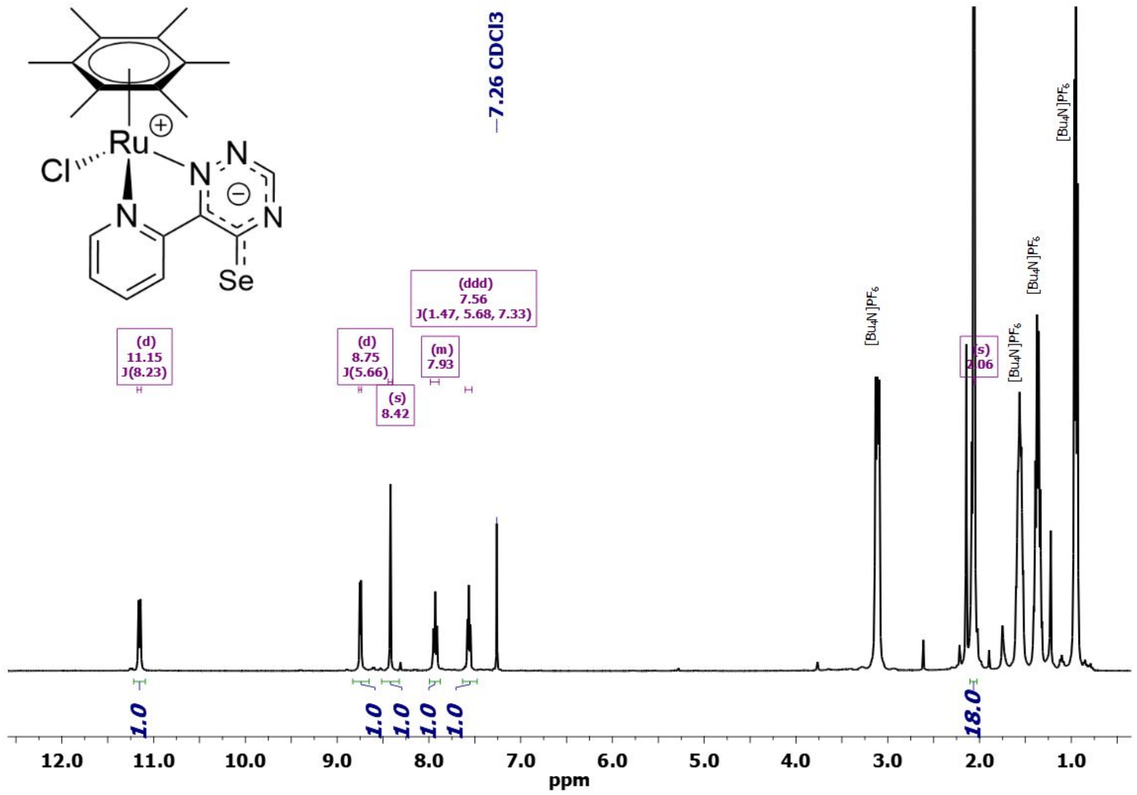

Figure S50. $^{13}C\{^1H\}$ NMR spectrum (126 MHz, $CDCl_3$) of **3d** (in admixture with $[Bu_4N]PF_6$).

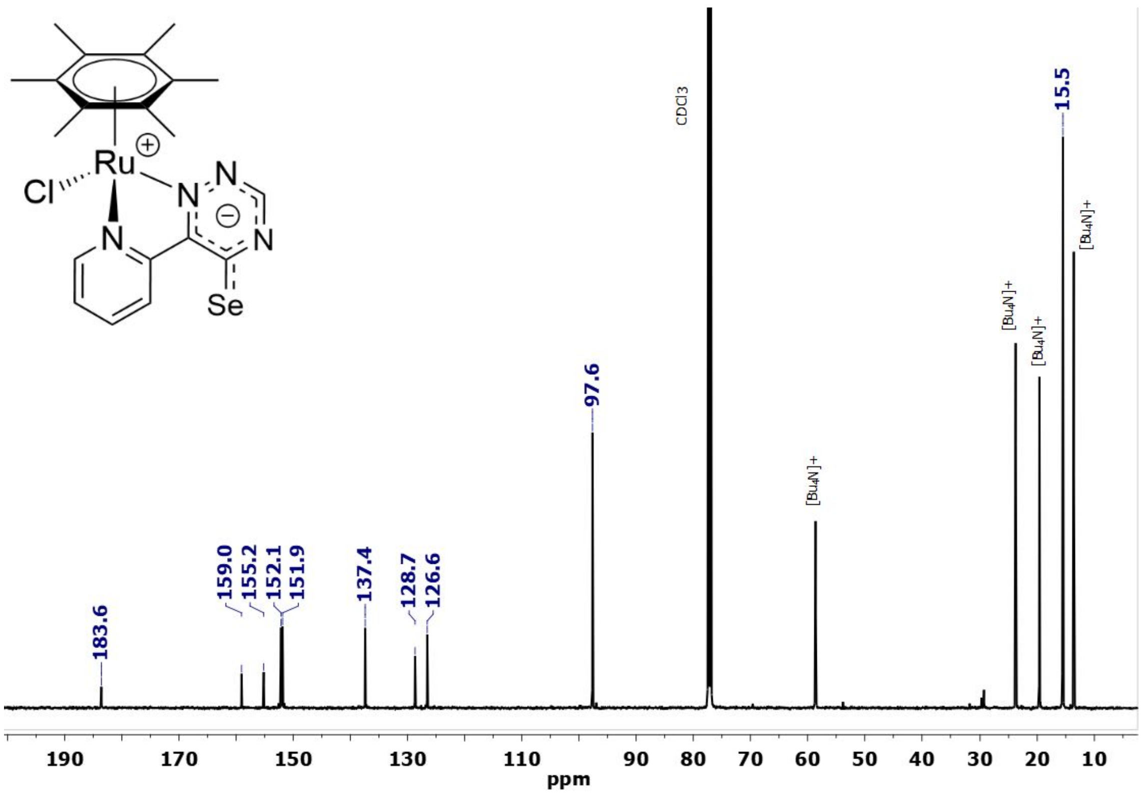

Figure S51. Section of the ^{1}H-^{13}C HSQC NMR spectrum ($CDCl_3$) of **3d**, related to triazine resonances.

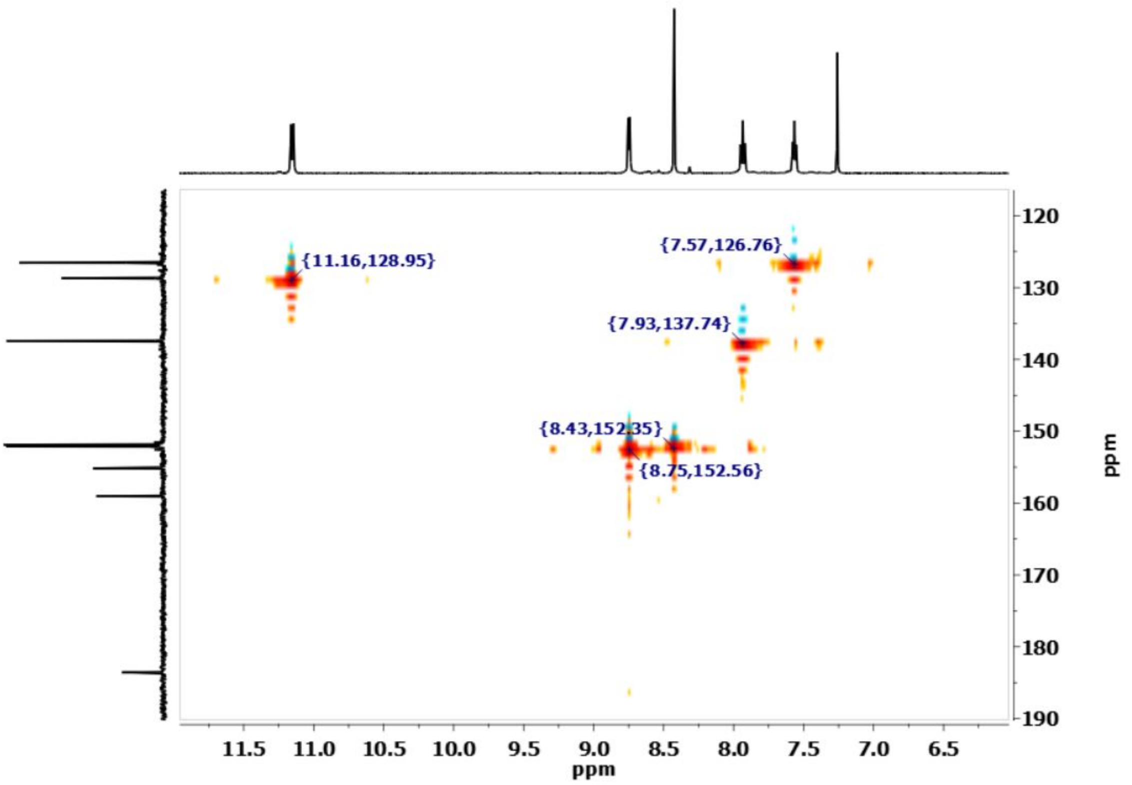

Figure S52. Section of the ^{1}H-^{13}C HMBC NMR spectrum ($CDCl_3$; long range J = 8 Hz, Δ_2 = 62.5 ms) of **3d**, related to triazine resonances, showing the correlation between the triazinyl proton (8.43 ppm) and the CSe carbon (183.6 ppm).

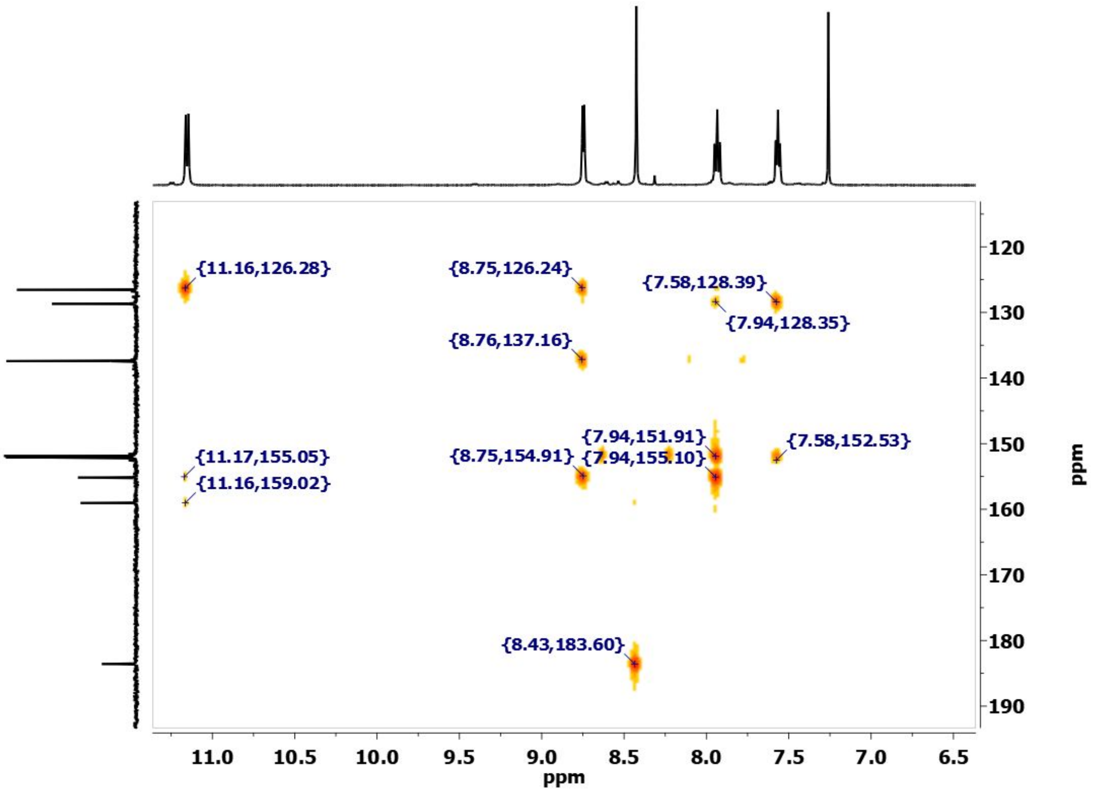

Figure S53. ^{77}Se NMR spectrum (76 MHz, $CDCl_3$) of **3d**.

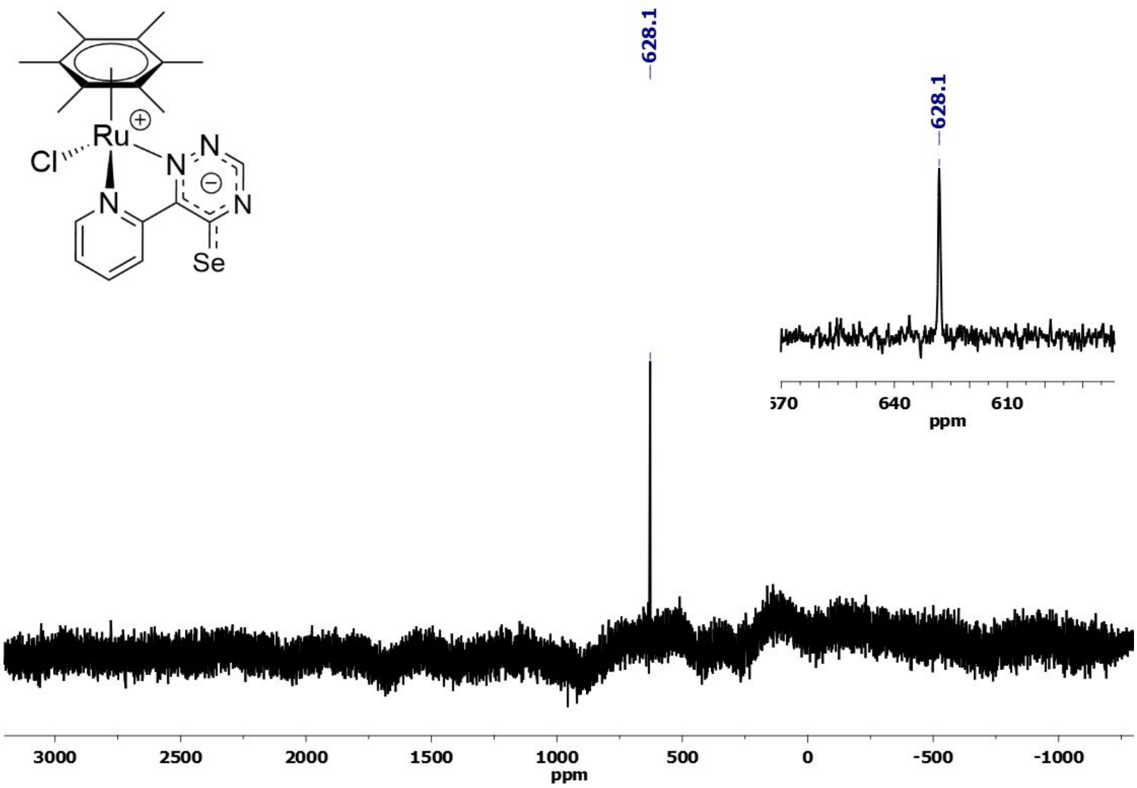

Figure S54. FIA-ESI(+)-MS spectrum of **3d** in MeCN. Calcd. base peak for [**3d**+H]$^+$ ($C_{20}H_{24}ClN_4RuSe$): 536.9985.

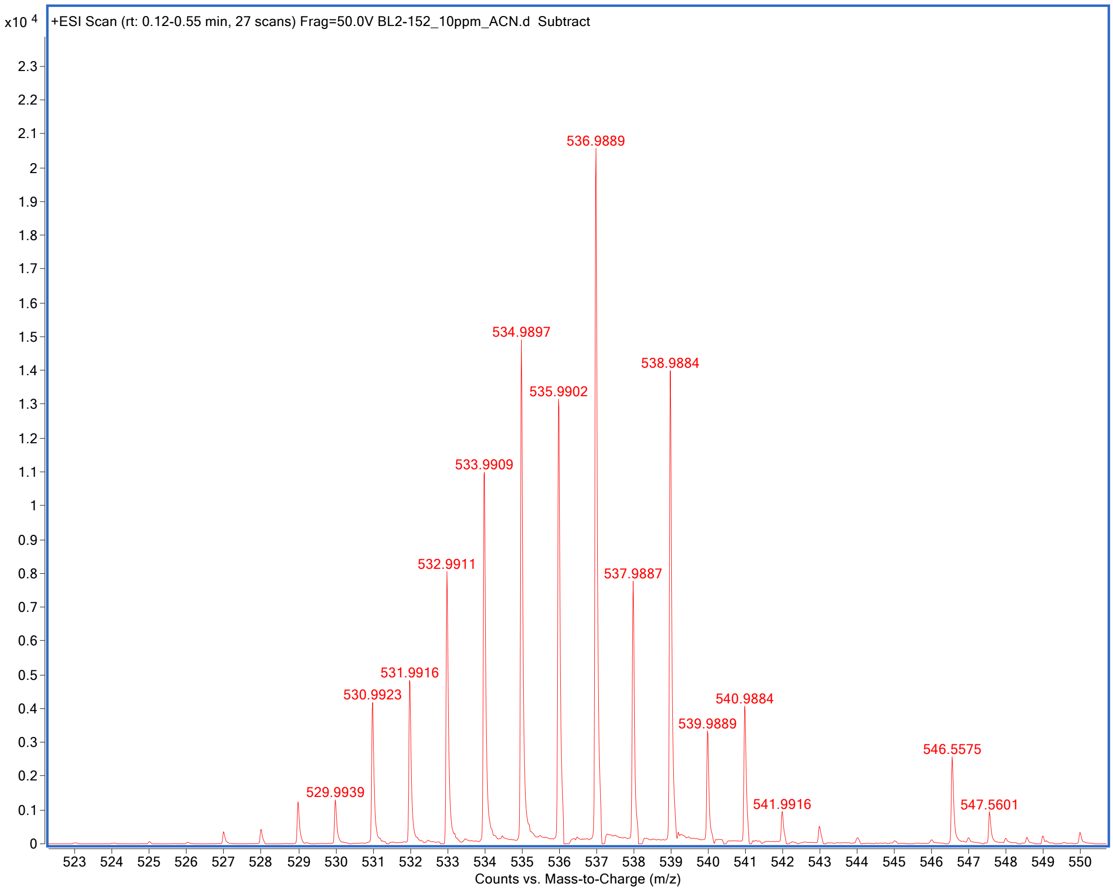

Figure S55. Solid-state IR spectrum (650-4000 cm^{-1}) of [RuCl{κ^2*N*-3-methyl-6-(2-pyridyl)-5-selenoxo-1,2,4-triazinide}(η^6-C_6Me_6)], **3e**, in admixture with [Bu_4N]PF_6, showing no bands of Ru-coordinated selenocyanate.

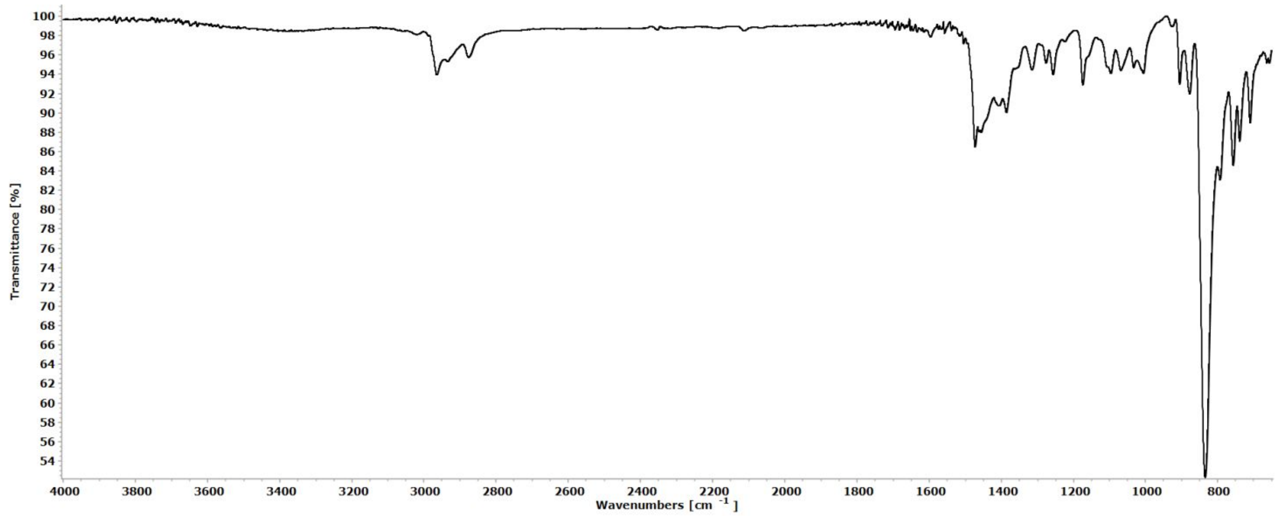

Figure S56. ^{1}H NMR spectrum (400 MHz, $CDCl_3$) of **3e** (in admixture with [Bu_4N]PF_6). The set of signals possibly ascribable to the oxygenated derivative **3e^O** is marked with asterisk (*)

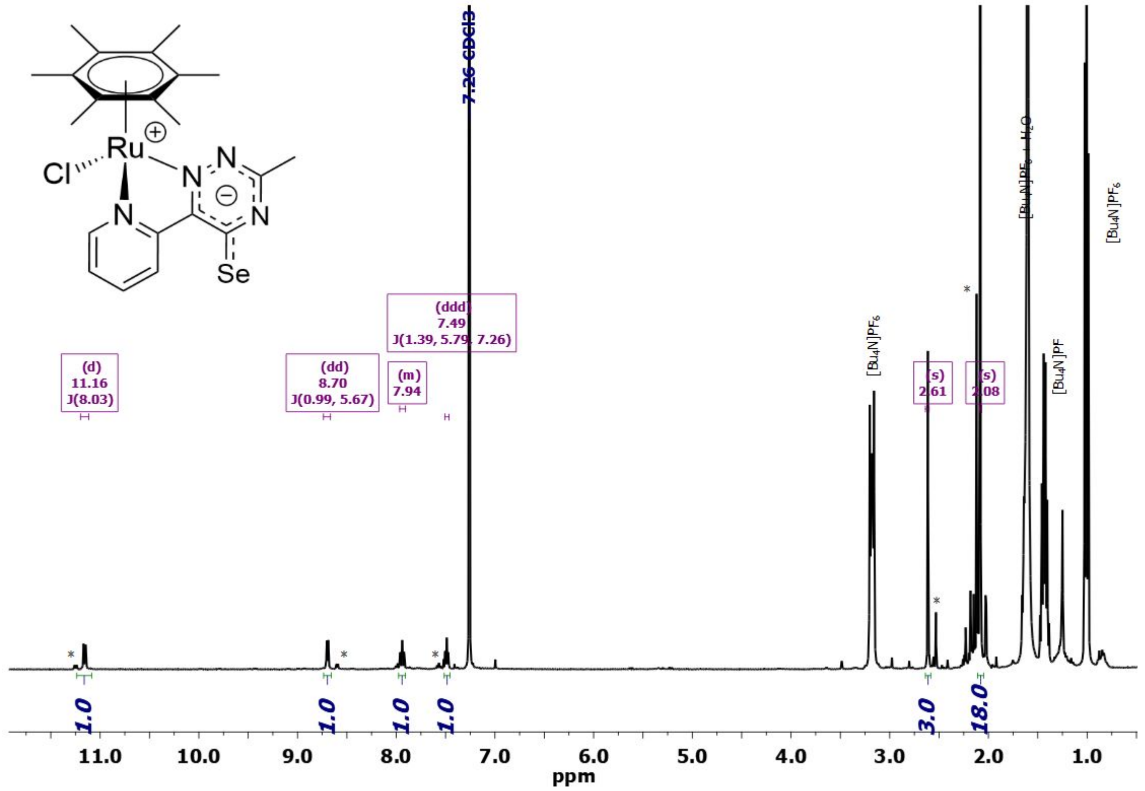

Figure S57. $^{13}C\{^1H\}$ NMR spectrum (100 MHz, $CDCl_3$) of **3e** (in admixture with $[Bu_4N]PF_6$).

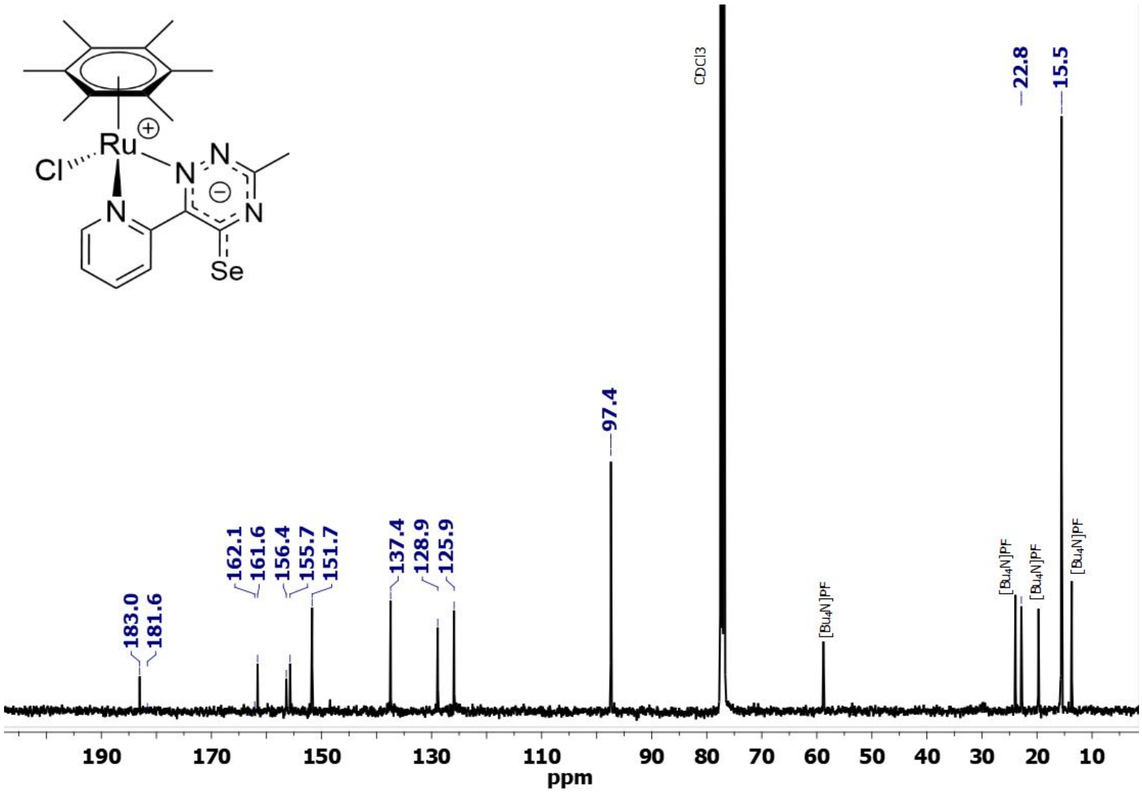

Figure S58. Section of the ^1H-^{13}C HSQC NMR spectrum ($CDCl_3$) of **3e**, related to triazine resonances.

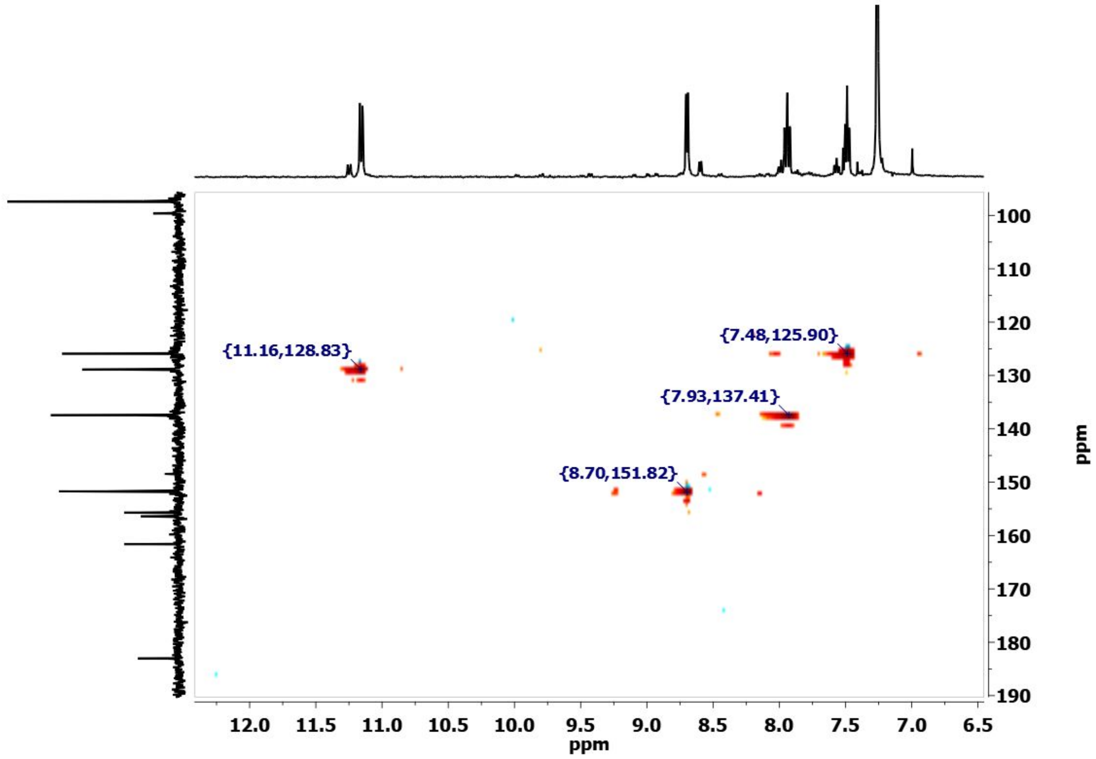

Figure S59. Section of the ^{1}H-^{13}C HMBC NMR spectrum ($CDCl_3$; long range J = 8 Hz, Δ_2 = 62.5 ms) of **3e**, related to triazine resonances, showing no correlation between the triazinyl methyl (2.60 ppm) and the CSe carbon (183.0 ppm).

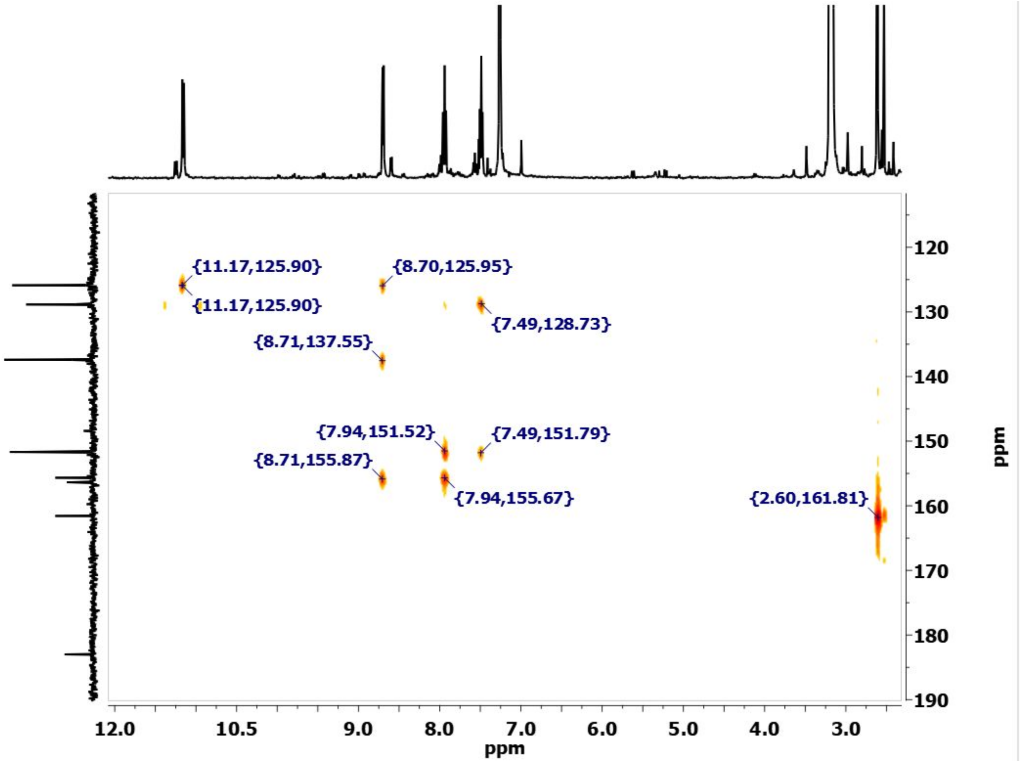

Figure S60. ^{77}Se NMR spectrum (76 MHz, $CDCl_3$) of **3e**.

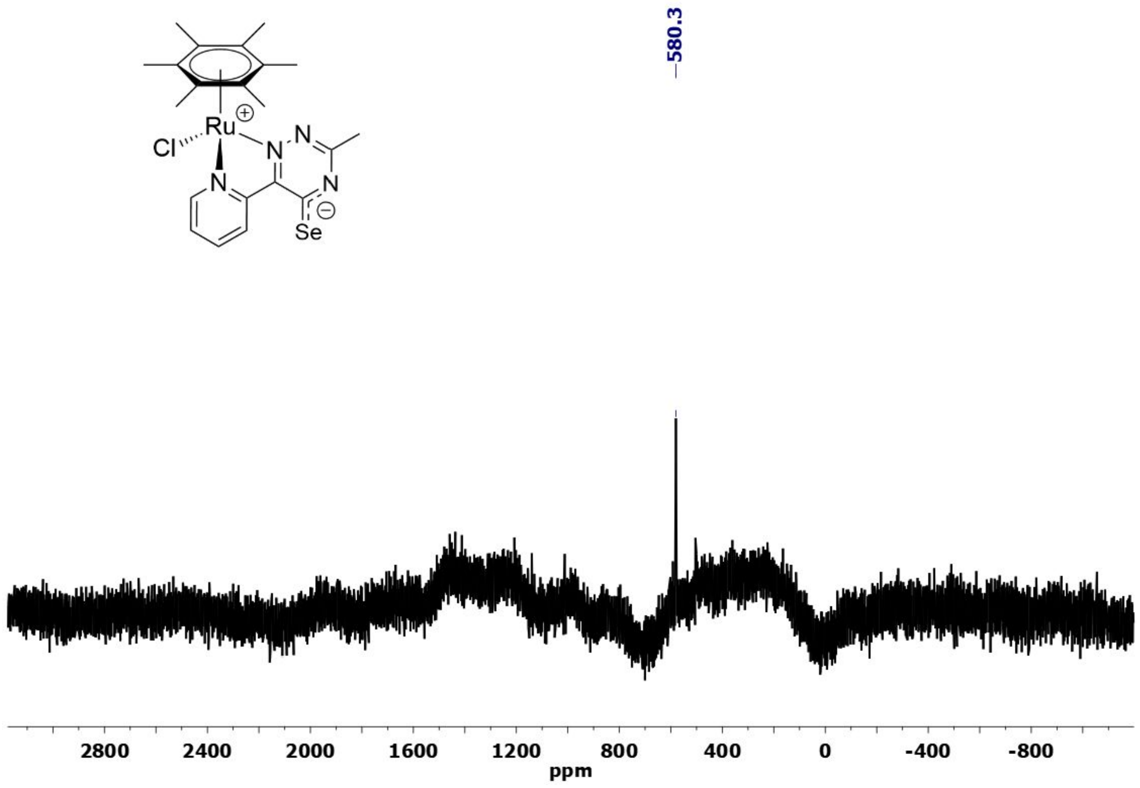

Figure S61. FIA-ESI(+)-MS spectrum of **3e** in MeCN. Calcd. base peak for [**3e**+H]$^+$ ($C_{21}H_{26}ClN_4RuSe$): 551.0141 Da.

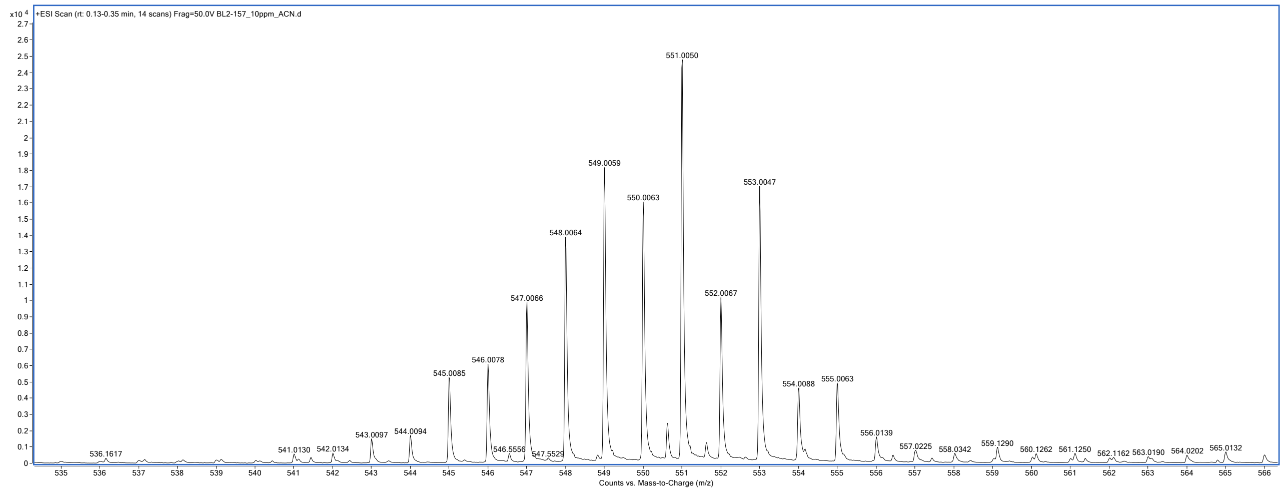

Figure S62. Structure and FIA-ESI(+)-MS spectrum of **3e*^{O}*** in MeCN, formed by hydrolysis of **3e** on silica column. Calcd. base peak for [**3e*^{O}***+H]$^+$: 487.0866 Da.

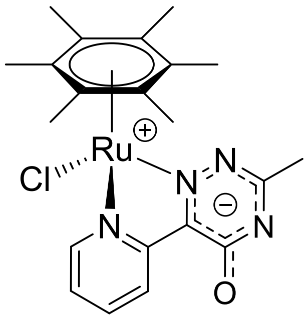

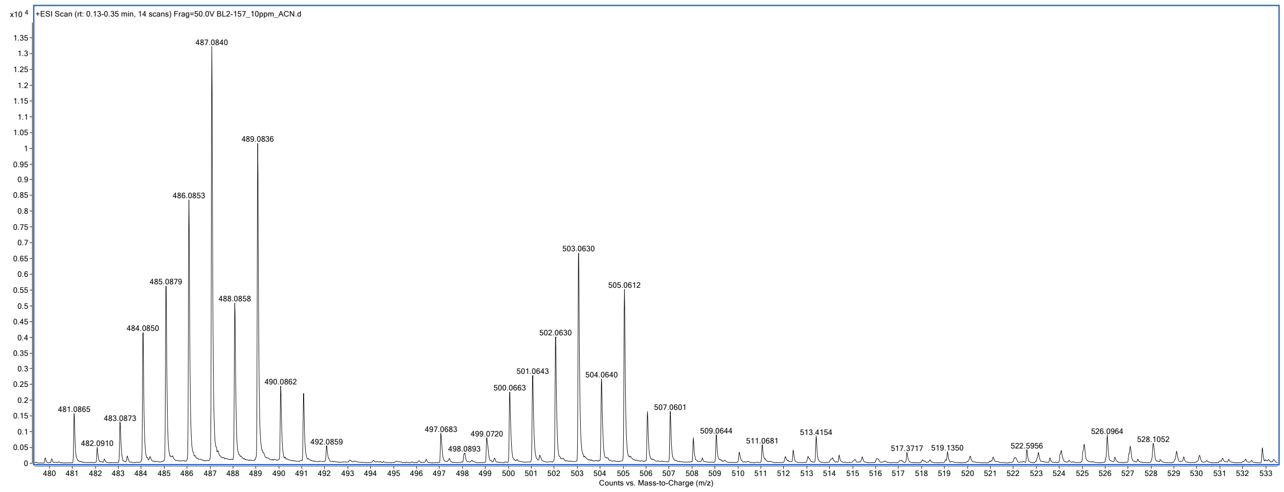

Reactivity of pyridyl-tetrazine complexes with cyanate: IR and NMR spectra

Figure S63. ^{1}H NMR spectra (400 MHz, acetone-d_6, 5-13 ppm) of [**1a**]PF_6 (top) and after addition of $[Bu_4N][OCN]$ (bottom).

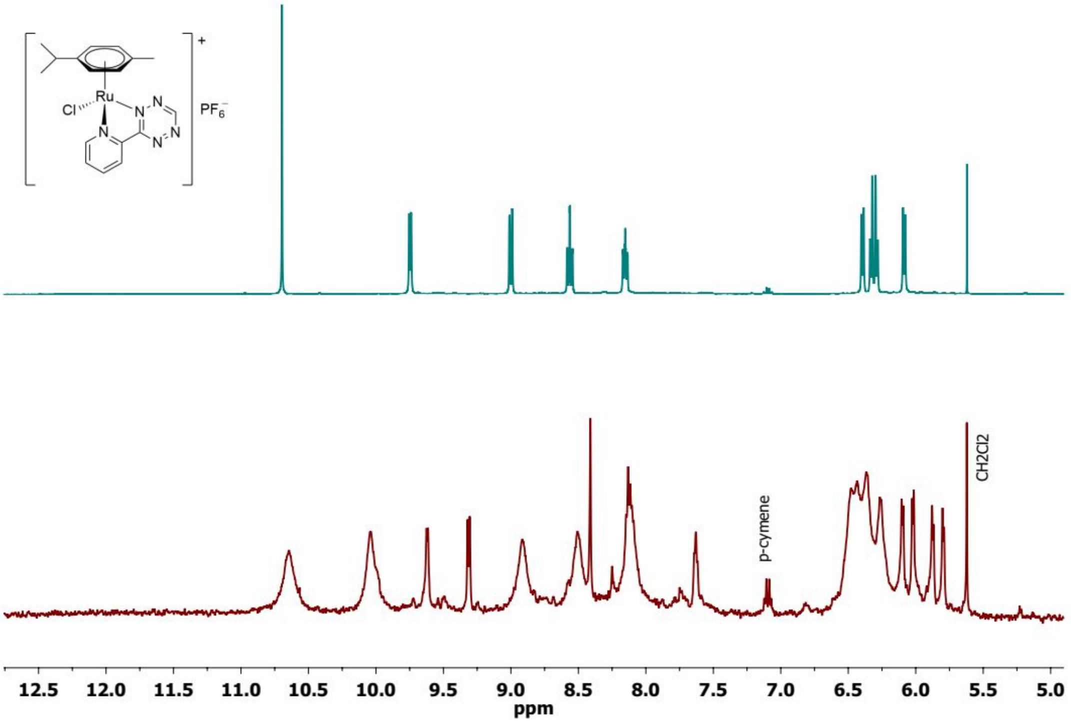

Figure S64. ^{1}H NMR spectra (400 MHz, acetone-d_6, 5-13 ppm) of [**1b**]PF_6 (**a**), the mixture of products resulting from its reaction with $[Bu_4N][OCN]$ (**b**) and after subsequent addition of *p*-toluenesulfonic acid (**c**).

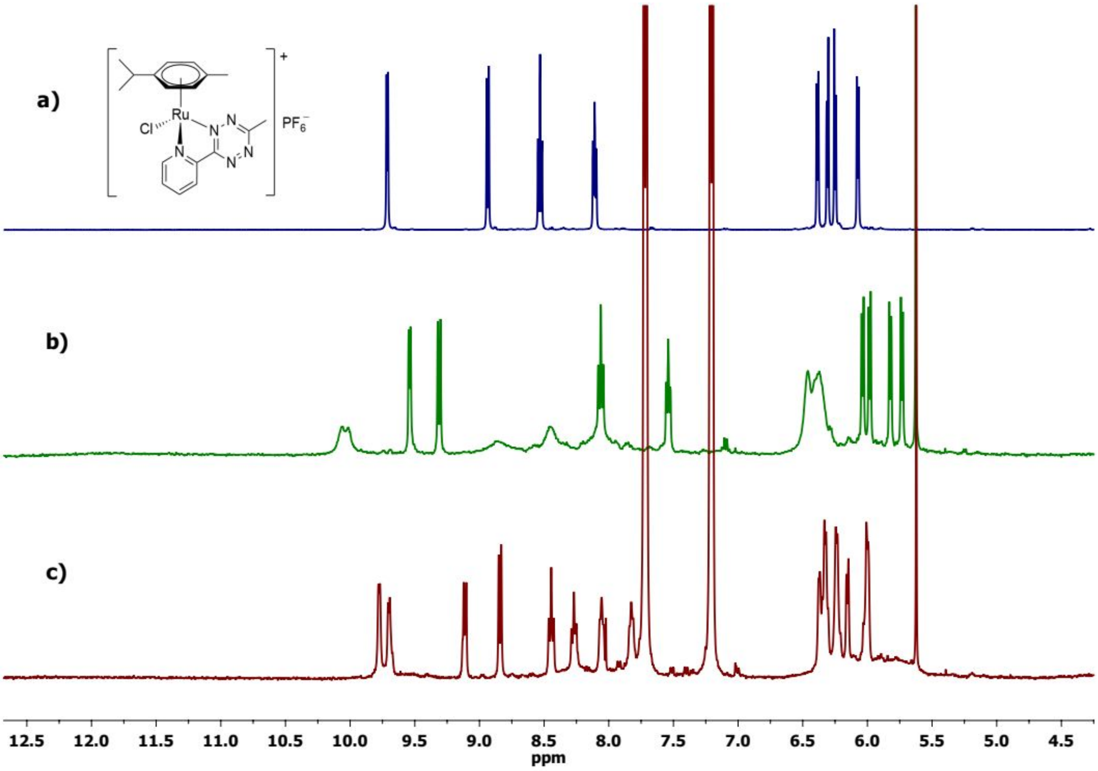

Figure S65. Solid-state IR spectrum (650-4000 cm^{-1}) of the mixture of products obtained by the reaction of [**1a**]PF_6 and [Bu_4N][OCN], showing absence of Ru-coordinated cyanate.

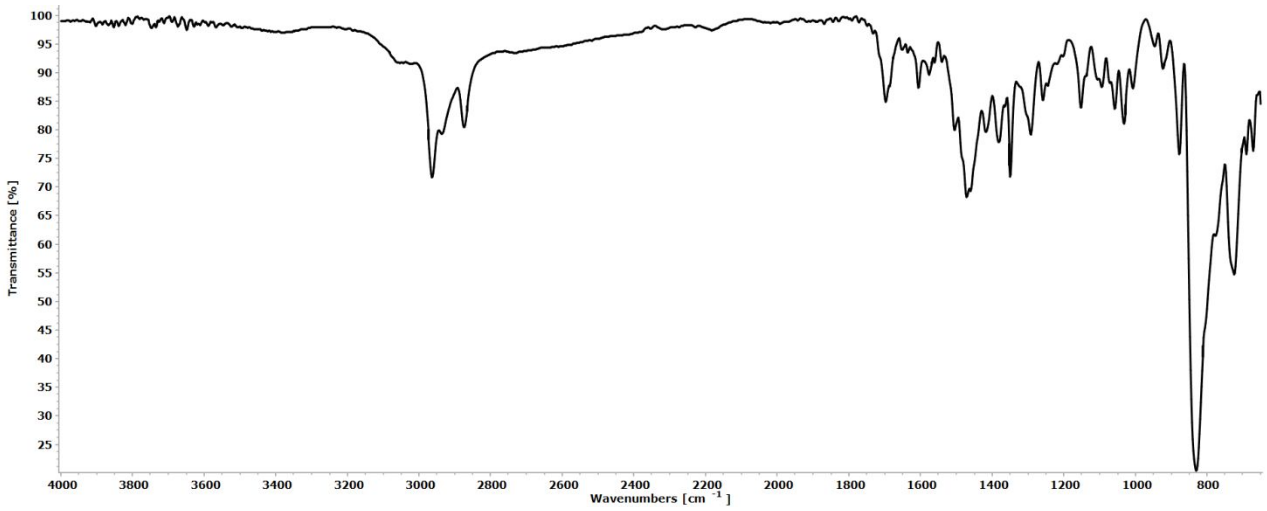

Tetrazine/chalcogenocyanate reactivity: comparison of NMR data

Table S1. Comparison of ^{1}H NMR data (δ/ppm)[a] for the *N*-heterocyclic and arene ligands in tetrazine and triazine complexes (H atom numbering is given below). Chemical shift variations (Δ) due to reaction with thiocyanate / selenocyanate (yellow background) or protonation (green background) are given in blue (negative, shielding) or in red (positive, deshielding).

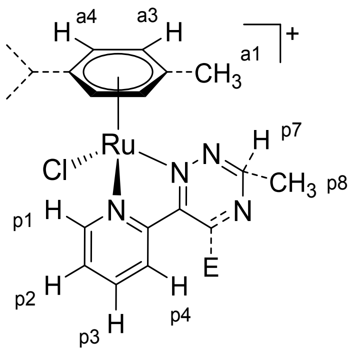

Cpd.	p1	p2	p3	p4	p7/p8	a4	a4’	a3	a3’
[1a]PF_6	9.75	8.15	8.56	9.00	10.7	6.39	6.33	6.29	6.09
2a	9.55	7.64	8.13	10.88	8.34	6.05	5.99	5.83	5.78
Δ(2a/1a$^+$)	− 0.2	− 0.51	− 0.43	+ 1.88	− 2.36	− 0.34	− 0.34	− 0.46	− 0.31
[1b]PF_6	9.71	8.11	8.53	8.94	3.29	6.39	6.31	6.25	6.07
2b	9.52	7.59	8.10	10.89	2.42	6.03	5.99	5.81	5.77
Δ(2b/1b$^+$)	− 0.19	− 0.52	− 0.43	+ 1.95	− 0.87	− 0.36	− 0.32	− 0.44	− 0.3
3b	9.57	7.65	8.11	11.26	2.46	6.06	6.00	5.84	5.79
Δ(3b/1b$^+$)	− 0.14	− 0.46	− 0.42	+ 2.32	− 0.83	− 0.33	− 0.31	− 0.41	− 0.28
[2bH]PF_6	9.66	7.82	8.27	10.28	2.72	6.21	6.11	5.98	5.96
Δ(2bH$^+$/2b)	+ 0.14	+ 0.23	+ 0.17	-0.61	+ 0.30	+ 0.18	+ 0.12	+ 0.17	+ 0.19
[1c]PF_6	9.74	8.14	8.56	9.03	-	6.46	6.38	6.31	6.13
2c	9.64	7.66	8.14	10.92	-	6.16	6.13	5.95	5.90
Δ(2c/1c$^+$)	− 0.10	− 0.48	− 0.42	+ 1.89	-	− 0.30	− 0.25	− 0.36	− 0.23

Cpd.	p1	p2	p3	p4	p7/p8	a1
[1d]PF_6	9.25	8.17	8.50	8.94	10.66	2.29
2d	9.02	7.70	8.08	10.87	8.40	2.14
Δ(2d/1d$^+$)	− 0.23	− 0.47	− 0.42	+ 1.93	− 2.26	− 0.15
3d [b]	8.75	7.56	7.93	11.15	8.42	2.06
Δ(3d/1d$^+$)	− 0.50	− 0.61	− 0.57	+ 2.21	− 2.24	− 0.23
[1e]PF_6 [b]	8.84	7.87	8.20	8.82	3.25	2.22
3e	9.00	7.66	8.07	10.87	2.48	2.14
Δ(3e/1e$^+$)	+ 0.16	− 0.21	− 0.13	+ 2.05	− 0.77	− 0.08
3e [b]	8.70	7.49	7.93	11.16	2.61	2.07
Δ(3e/1e$^+$)	− 0.14	− 0.38	− 0.27	+ 2.34	− 0.64	− 0.15
[3eH]PF_6	9.08	7.81	8.16	10.73	2.80	2.20
Δ(3eH$^+$/3e)	+ 0.38	+ 0.32	+ 0.23	− 0.43	+ 0.19	+ 0.13

[a] NMR data refer to acetone-d_6 solutions, unless otherwise stated. [b] $CDCl_3$ solution.

Table S2. Comparison of $^{13}C\{^1H\}$ NMR data (δ/ppm)[a] for the *N*-heterocyclic and arene ligands in tetrazine and triazine complexes (C atom numbering is given below). Chemical shift variations (Δ) due to reaction with thiocyanate / selenocyanate (yellow background) or protonation (green background) are given in blue (negative, shielding) or in red (positive, deshielding).

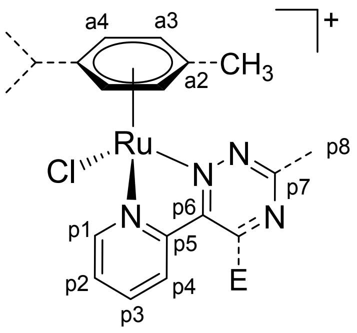

Cpd.	p1	p2	p3	p4	p5	p6	p7	p8	a4	a4’	a3	a3’
**[1a]PF$_6$**	157.6	132.1	141.9	127.9	150.0	168.3	159.4	-	92.0	90.03	90.01	89.5
**2a**	156.1	126.9	138.5	129.7	156.7	156.5	153.2	-	91.0	88.8	87.8	87.4
**Δ(2a/1a$^+$)**	**− 1.5**	**− 5.2**	**− 3.4**	**+ 1.8**	**+ 6.7**	**− 11.8**	**− 6.2**	-	**− 1.0**	**− 1.2**	**− 2.2**	**− 2.1**
**[1b]PF$_6$**	157.4	131.6	141.8	127.2	150.2	165.7	170.4	21.9	91.8	89.9	89.8	89.1
**2b**	155.9	126.5	138.4	129.6	156.8	153.9	162.1	23.0	91.3	88.8	87.7	87.3
**Δ(2b/1b$^+$)**	**− 1.5**	**− 5.1**	**− 3.4**	**+ 2.4**	**+ 6.6**	**− 11.8**	**− 8.3**	**+ 1.1**	**− 0.5**	**− 1.1**	**− 2.1**	**− 1.8**
**[2bH]PF$_6$**	157.0	128.5	139.4	131.3	154.1	156.8	157.9	20.0	91.7	90.3	89.1	88.9
**Δ(2bH$^+$/2b)**	**+ 1.1**	**+ 2.0**	**+ 1.0**	**+ 1.7**	**− 2.7**	**+ 2.9**	**− 4.2**	**− 3.0**	**+ 0.4**	**+ 1.5**	**+ 1.4**	**+ 1.6**
**[1c]PF$_6$**	157.6	131.9	141.8	127.3	151.7	166.3	164.6	149.7	92.4	90.4	90.0	89.2
**2c**	155.4	126.1	138.5	129.6	155.4	155.3	157.9	154.3	91.5	88.9	88.1	87.5
**Δ(2c/1c$^+$)**	**− 2.2**	**− 5.8**	**− 3.3**	**+ 2.3**	**+ 3.7**	**− 11.0**	**− 6.7**	**+ 4.6**	**− 0.9**	**− 1.5**	**− 1.9**	**− 1.7**

Cpd.	p1	p2	p3	p4	p5	p6	p7	p8	a2
**[1d]PF$_6$**	154.8	132.1	141.2	127.3	150.0	168.8	159.3	-	102.0
**2d**	153.9	127.0	138.0	128.8	156.1	156.8	153.1	-	98.5
**Δ(2d/1d$^+$)**	**− 0.9**	**− 5.1**	**− 3.2**	**+ 1.5**	**+ 6.1**	**− 12**	**− 6.2**		**− 3.5**
**3d** [b]	152.1	126.6	137.4	128.7	155.2	159.0	151.9	-	97.6
**Δ(3d/1d$^+$)**	**− 2.7**	**− 5.5**	**− 3.8**	**+ 1.4**	**+ 5.2**	**− 9.8**	**− 7.4**	-	**− 4.4**
**[1e]PF$_6$**	154.6	131.6	141.2	126.7	150.2	166.2	170.1	21.6	101.6
**2e**	153.7	126.6	137.9	128.8	156.3	154.0	161.8	22.4	98.4
**Δ(2e/1e$^+$)**	**− 0.9**	**− 5**	**− 3.3**	**+ 2.1**	**+ 6.1**	**− 12.2**	**− 8.3**	**+ 0.8**	**− 3.2**
**3e** [b]	151.7	125.9	137.4	128.9	155.7	156.4	161.6	22.8	97.4
**Δ(3e/1e$^+$)**	**− 2.9**	**− 5.7**	**− 3.8**	**+ 2.2**	**+ 5.5**	**− 9.8**	**− 8.5**	**+ 1.2**	**− 4.2**

[a] NMR data refer to acetone-d_6 solutions, unless otherwise stated. [b] $CDCl_3$ solutions.

Tetrazine/chalcogenocyanate reactivity: UV-Vis monitoring and kinetic analysis

Figure S66. Solvent-subtracted UV-Vis spectra (320-800 nm) of [**1a**]PF_6 in acetone ($2.2 \cdot 10^{-4}$ M; blue line) and after addition of $[Bu_4N][OCN]$ (1.0 equivalents; yellow to red gradient). Arrows indicate the change in absorbance over time.

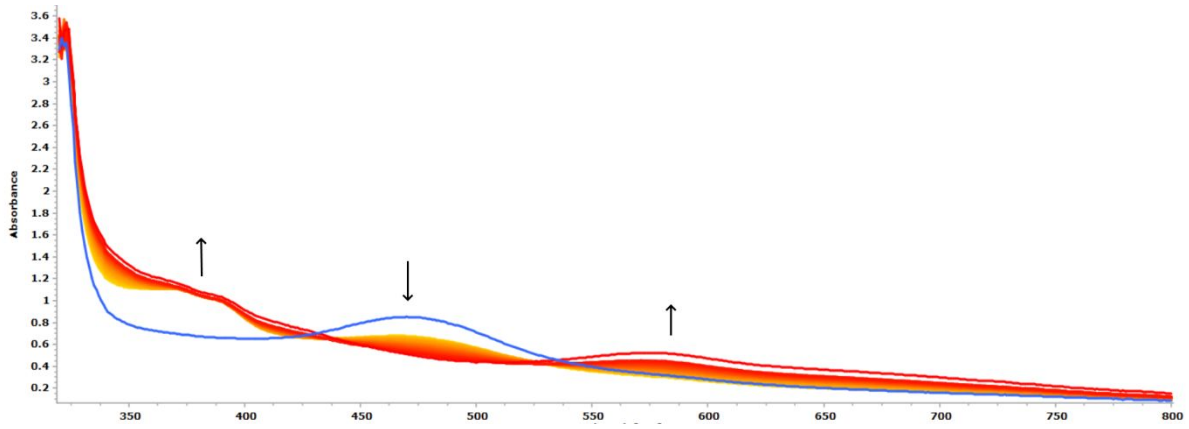

Figure S67. Solvent-subtracted UV-Vis spectra (320-800 nm) of [**1a**]PF_6 in acetone ($2.2 \cdot 10^{-4}$ M; blue line) and after addition of $[Bu_4N][SCN]$ (1.0 equivalents; yellow to red gradient). Arrows indicate the change in absorbance over time.

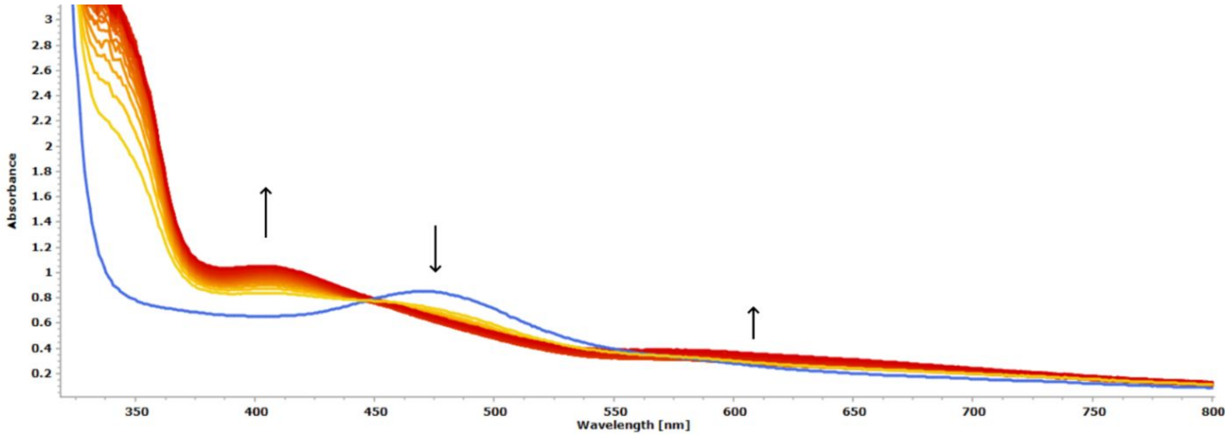

Figure S68. Solvent-subtracted UV-Vis spectra (330-800 nm) of [**1a**]PF_6 in acetone ($2.2 \cdot 10^{-4}$ M; blue line) and after addition of K[SCN] (1.0 equivalents; yellow to red gradient). Arrows indicate the change in absorbance over time.

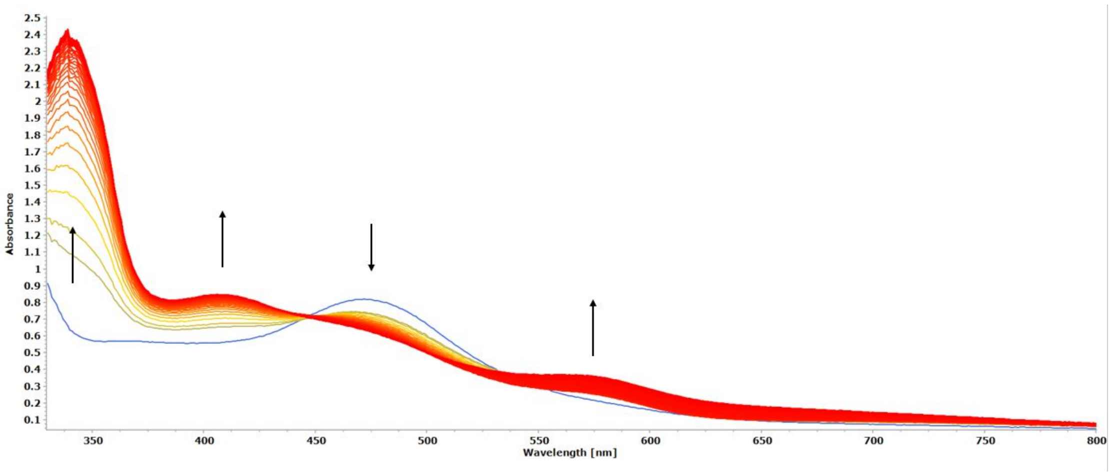

Figure S69. Solvent-subtracted UV-Vis spectra (320-800 nm) of [**1a**]PF_6 in acetone ($2.2 \cdot 10^{-4}$ M; blue line) and after addition of [Bu_4N][SeCN] (1.0 equivalents; blue-yellow-red gradient). Arrows indicate the change in absorbance over time.

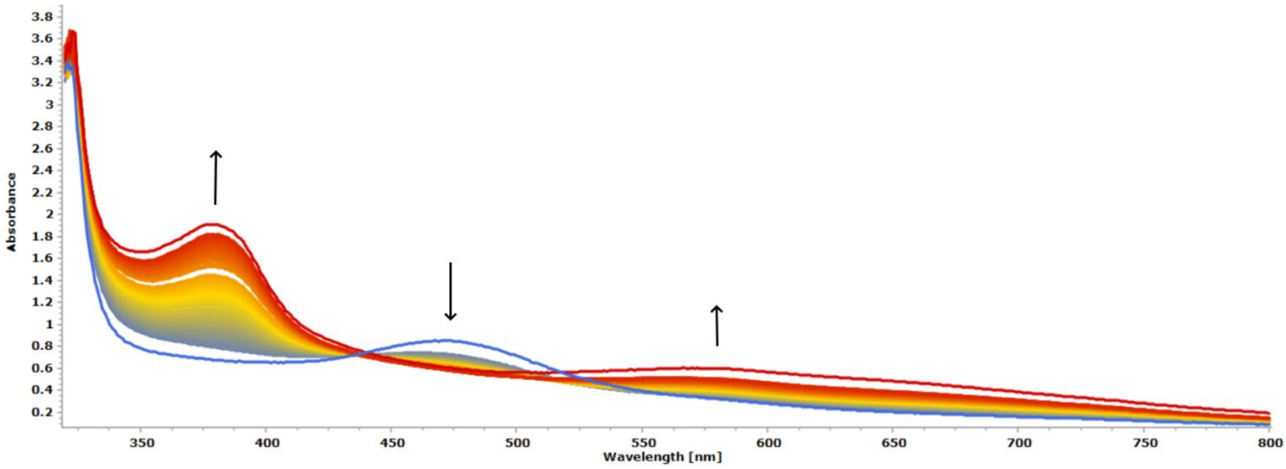

Figure S70. Solvent-subtracted UV-Vis spectra (320-800 nm) of [**1b**]PF_6 in acetone ($2.0 \cdot 10^{-4}$ M; blue line) and after addition of [Bu_4N][OCN] (1.0 equivalents; yellow to red gradient). Arrows indicate the change in absorbance over time.

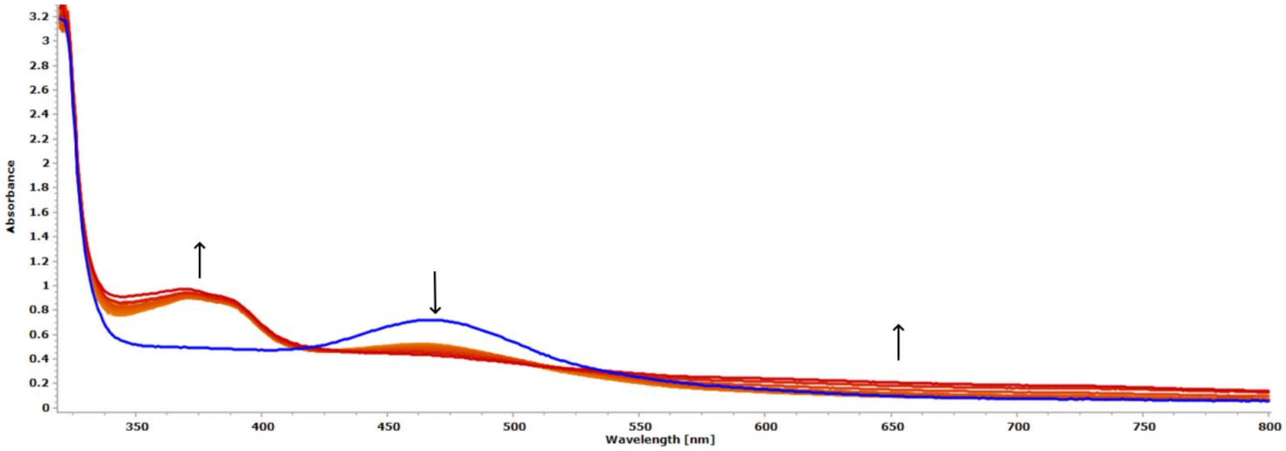

Figure S71. Solvent-subtracted UV-Vis spectra (320-800 nm) of [**1b**]PF_6 in acetone ($2.0 \cdot 10^{-4}$ M; blue line) and after addition of [Bu_4N][SCN] (1.0 equivalents; blue-yellow-red gradient). Arrows indicate the change in absorbance over time.

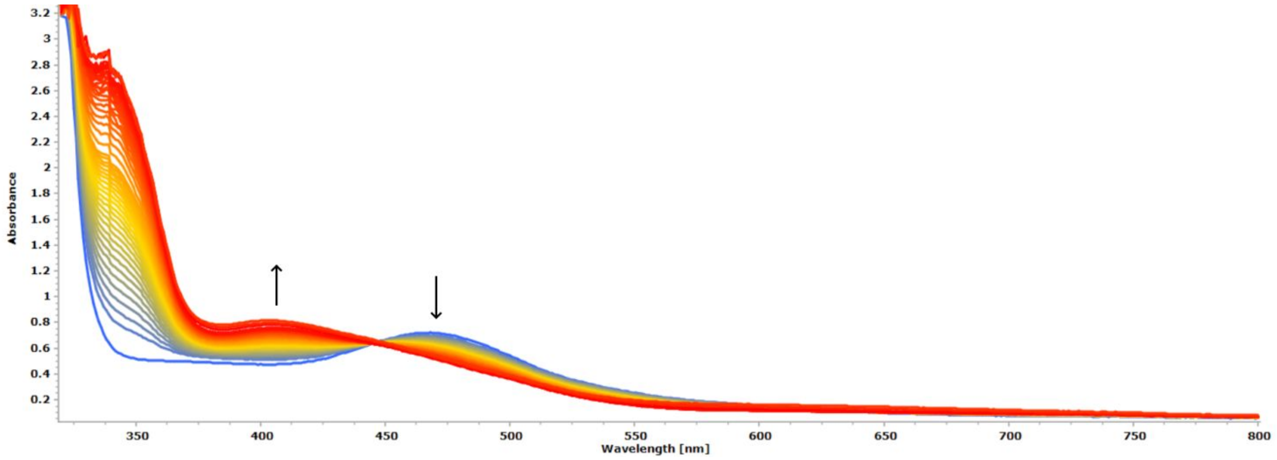

Figure S72. Solvent-subtracted UV-Vis spectra (320-800 nm) of [**1b**]PF_6 in acetone ($3.5\cdot10^{-4}$ M; blue line) and after addition of K[SCN] (1.0 equivalents; blue-yellow-red gradient). Arrows indicate the change in absorbance over time.

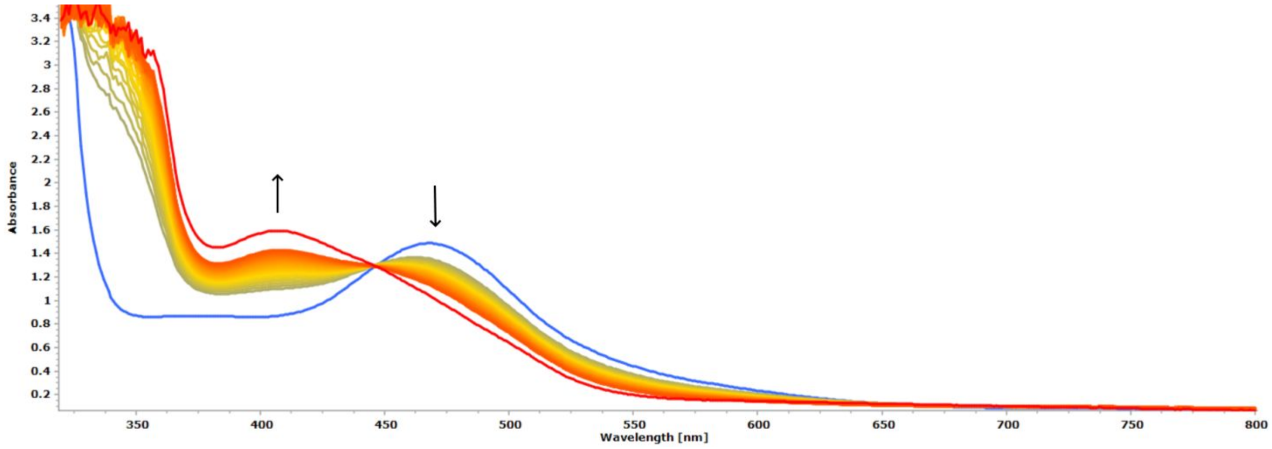

Figure S73. Solvent-subtracted UV-Vis spectra (320-800 nm) of [**1b**]PF_6 in acetone ($2.0\cdot10^{-4}$ M; blue line) and after addition of [Bu_4N][SCN] (1.0 equivalents; blue-yellow-red gradient). Arrows indicate the change in absorbance over time.

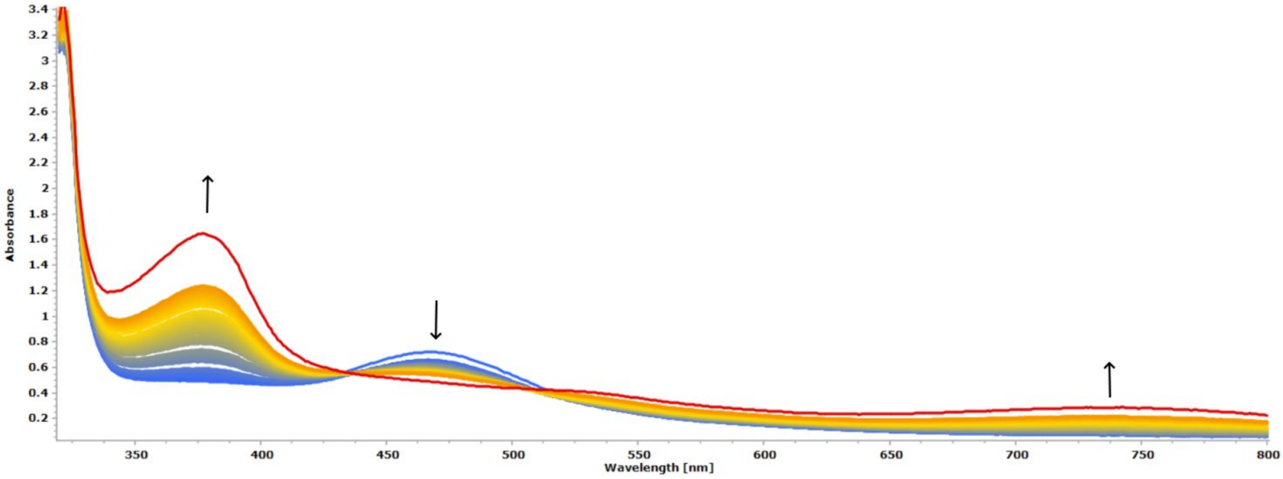

Figure S74. Conversion/time profiles for the reactions of [**1a**]PF_6 and [Bu_4N][ECN] (E = O, yellow-orange line; E = S, blue line; E = Se, red line) or KSCN (green line) in acetone (1:1 ratio, $2.2\cdot10^{-4}$ M). Data treatment is described in the main text.

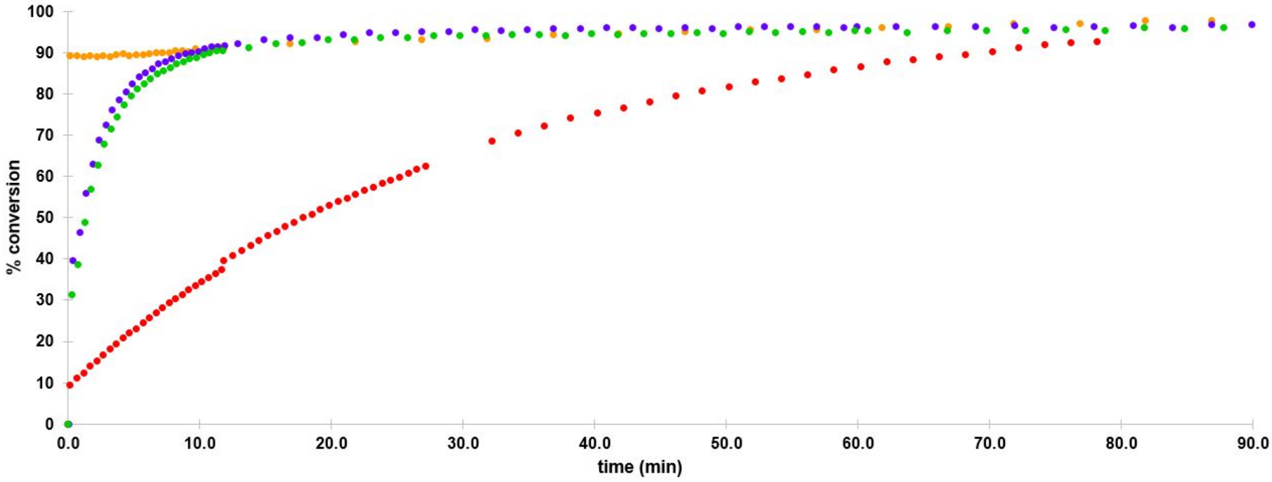

Figure S75. Conversion/time profiles for the reactions of [**1b**]PF_6 and [Bu_4N][ECN] (E = O, yellow-orange line; E = S, blue line; E = Se, red line; $2.0 \cdot 10^{-4}$ M) or KSCN (green line; $3.5 \cdot 10^{-4}$ M) in acetone (1:1 ratio). Data treatment is described in the main text.

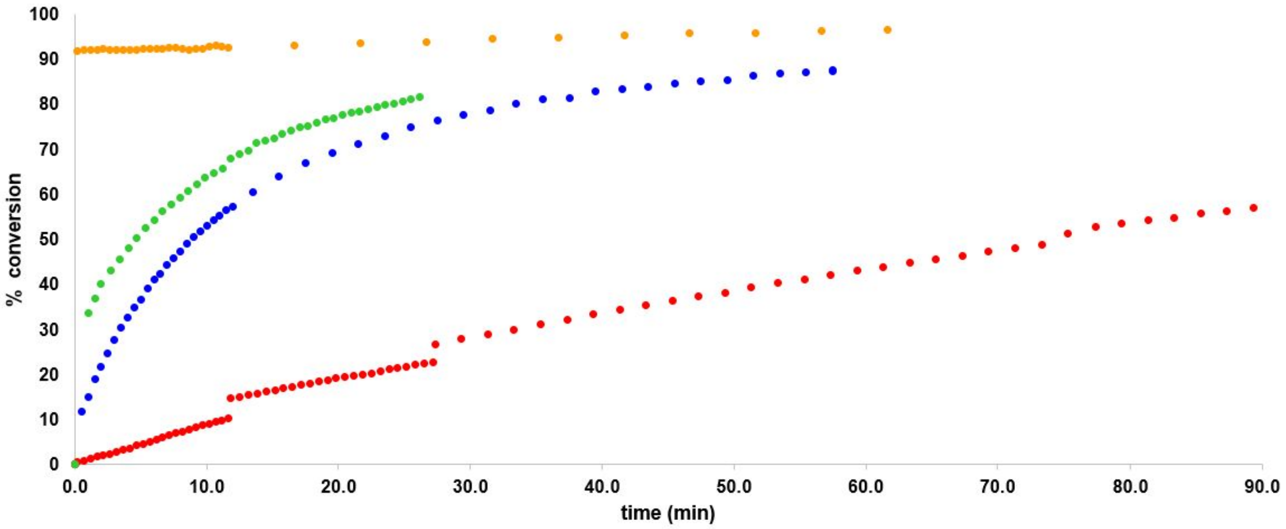

Figure S76. Solvent-subtracted UV-Vis spectra (320-800 nm) of a solution of [**1a**]PF_6 in acetone ($5.2 \cdot 10^{-4}$ M) at room temperature for 17 h (blue to yellow gradient); final spectrum in red. Arrows indicate the change in absorbance over time.

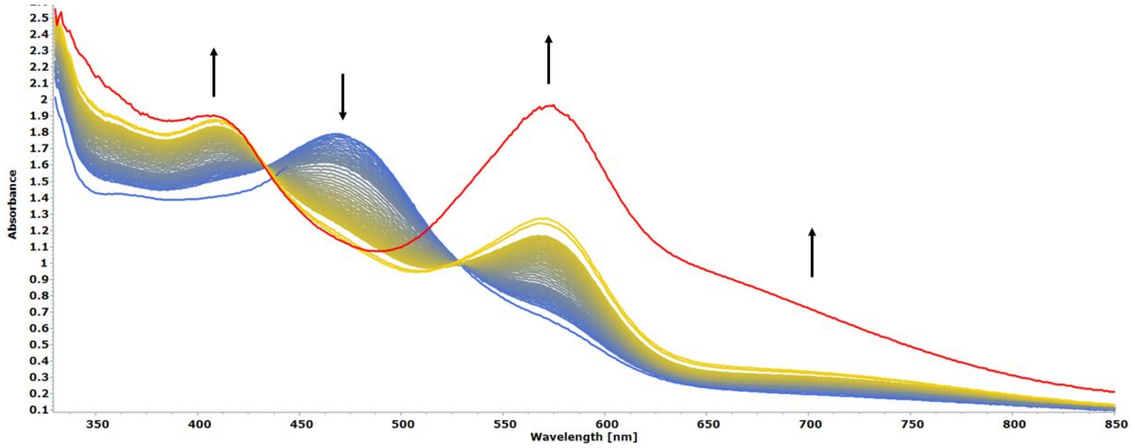

Figure S77. Solvent-subtracted UV-Vis spectra (320-800 nm) of a solution of [**1b**]PF_6 in acetone ($5.9 \cdot 10^{-4}$ M) at room temperature for 48 h (blue-yellow-red gradient); final spectrum in red. Arrows indicate the change in absorbance over time.

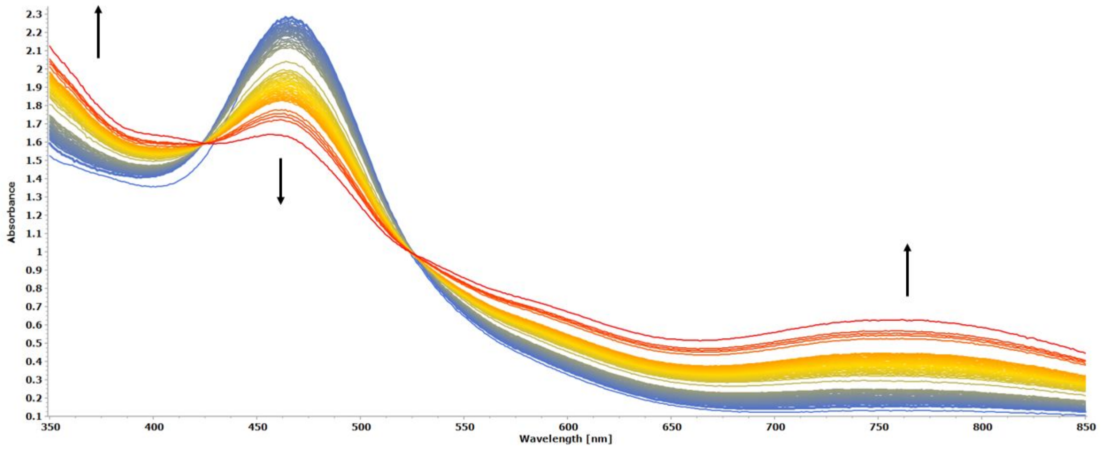

Figure S78. Least square linear regression for the reactions of [**1a**]PF_6 (left) or [**1b**]PF_6 (right) with [Bu_4N][SCN] in acetone. Data treatment is described in the main text.

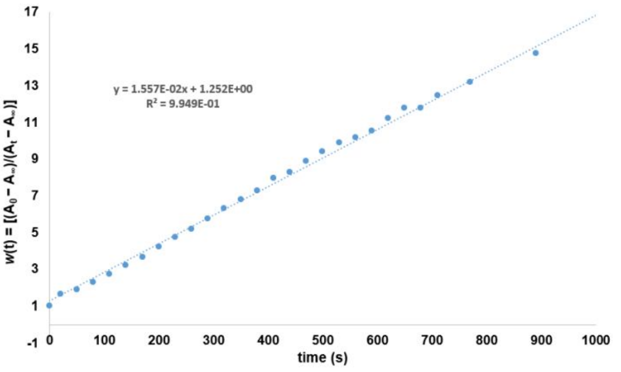

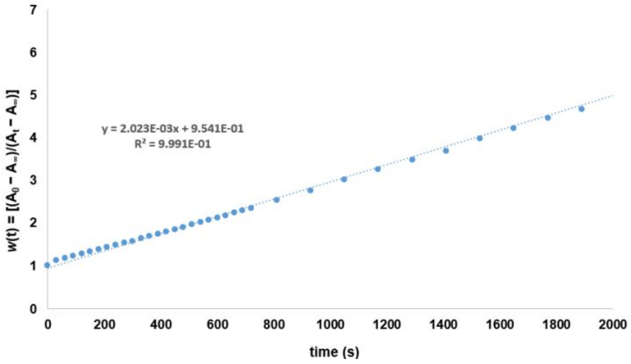

Figure S79. Least square linear regression for the reactions of [**1a**]PF_6 (left) or [**1b**]PF_6 (right) with K[SCN] in acetone. Data treatment is described in the main text.

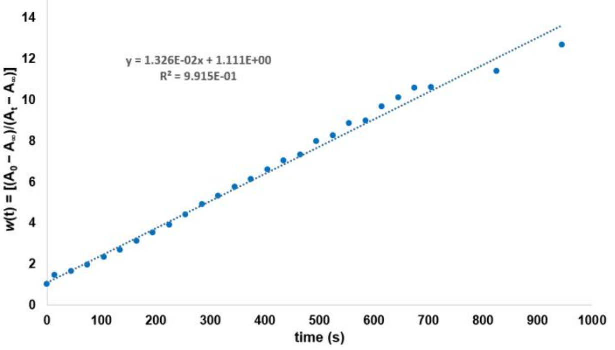

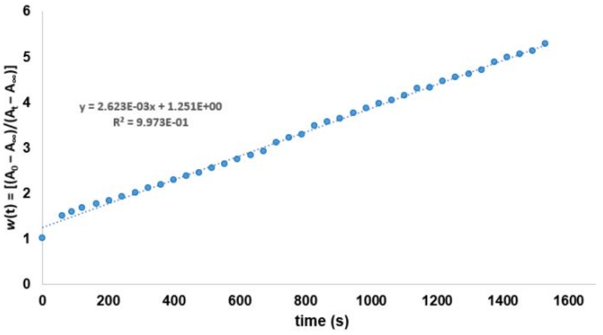

Figure S80. Least square linear regression for the reactions of [**1a**]PF_6 (left) or [**1b**]PF_6 (right) with [Bu_4N][SeCN] in acetone. Data treatment is described in the main text.

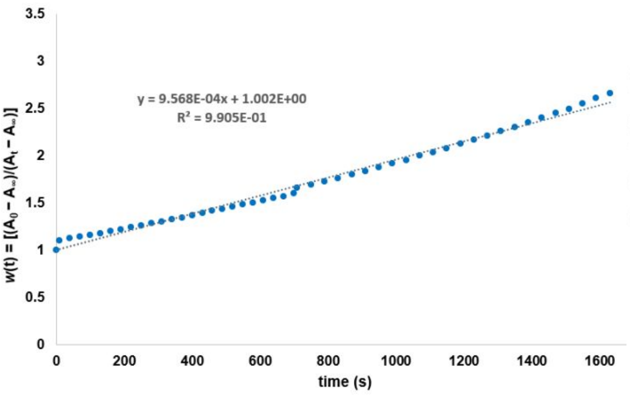

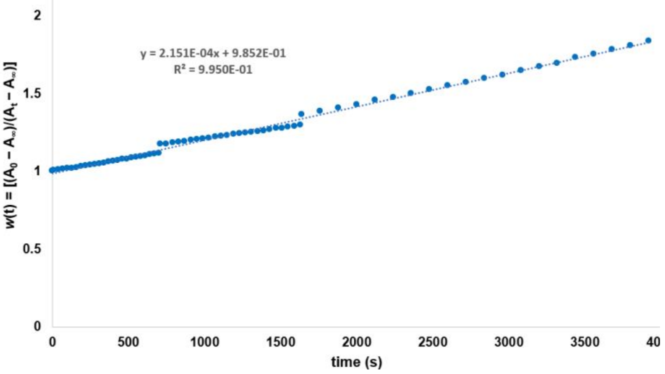

Figure S81. Least square linear regression for the decomposition of [**1a**]PF_6 (left) and [**1b**]PF_6 (right) in acetone solution. Data treatment is described in the main text.

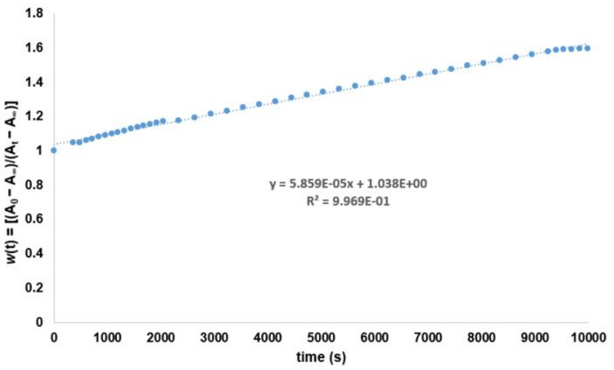

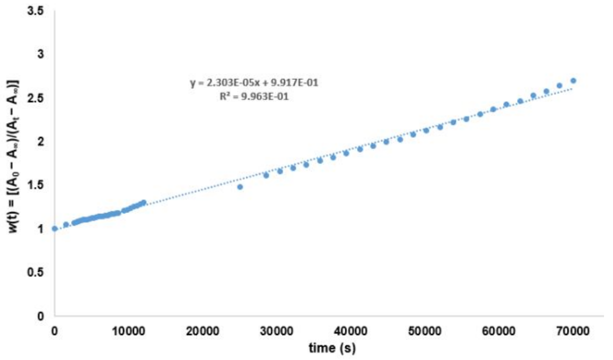

Preparation and X-Ray characterization of [RuCl(2,2'-bipyridine)(η^6-*p*-cymene)]PF_6

The compound was prepared according to a slightly modified literature procedure.[4] An orange suspension of [$RuCl_2$(η^6-*p*-cymene)]$_2$ (150 mg, 0.245 mmol) in MeCN (3 mL) was treated with NH_4PF_6 (84 mg, 0.52 mmol) and stirred at room temperature for 3 h. The resulting suspension was filtered over a celite pad to remove a colorless precipitate (NH_4Cl) and the filtrate was taken to dryness under vacuum. The residue was dissolved in CH_2Cl_2 (5 mL) and treated with 2,2'-bipyridine (77 mg, 0.49 mmol). The solution was stirred at room temperature for 2.5 h then filtered over a celite pad and taken to dryness under vacuum. The resulting yellow solid was washed with diethyl ether, hexane and dried under vacuum (40°C). Yield 271 mg, 97%. X-ray quality crystals of [RuCl(2,2'-bipyridine)(η^6-*p*-cymene)]PF_6 were obtained from an acetone solution layered with Et_2O and settled aside at – 20 °C. A polymorph of the present structure[4b] and the crystal structure of a methanol solvate ([RuCl(2,2'-bipyridine)(η^6-*p*-cymene)]PF_6·½MeOH)[5] were previously published.

^{1}H NMR (400 MHz, $CDCl_3$): δ/ppm = 9.57 (d, $^3J_{HH}$ = 5.2 Hz, 2H, p1), 8.28 (d, $^3J_{HH}$ = 7.8 Hz, 2H, p4), 8.08 (t, $^3J_{HH}$ = 7.8 Hz, 2H, p3), 7.72 (t, $^3J_{HH}$ = 6.4 Hz, 2H, p2), 6.10 (d, $^3J_{HH}$ = 6.2 Hz, 2H, a4), 5.94 (d, $^3J_{HH}$ = 6.2 Hz, 2H, a3), 2.70 (hept, $^3J_{HH}$ = 6.9 Hz, 1H, a6), 2.28 (s, 3H, a1), 1.07 (d, $^3J_{HH}$ = 6.9 Hz, 6H, a7). ^{1}H NMR (400 MHz, acetone-d$_6$): δ/ppm = 9.60 (d, $^3J_{HH}$ = 5.3 Hz, 2H, p1), 8.60 (d, $^3J_{HH}$ = 8.1 Hz, 2H, p4), 8.31 (td, $^3J_{HH}$ = 7.9, 1.3 Hz, 2H, p3), 7.81 (ddd, $^3J_{HH}$ = 7.2, 5.8 Hz, $^4J_{HH}$ = 1.1 Hz, 2H, p2), 6.23 (d, $^3J_{HH}$ = 6.3 Hz, 2H, a4), 5.98 (d, $^3J_{HH}$ = 6.3 Hz, 2H, a3), 2.77 (hept, $^3J_{HH}$ = 6.9 Hz, 1H, a6), 2.30 (s, 3H, a1), 1.08 (d, $^3J_{HH}$ = 6.9 Hz, 6H, a7).

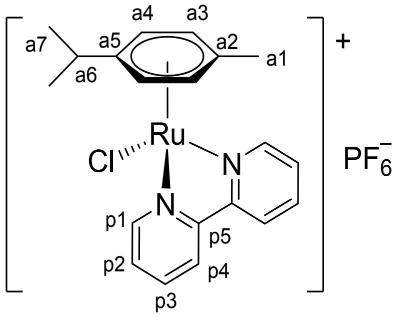

Chart S3. Structure of [RuCl(2,2'-bipyridine)(η^6-*p*-cymene)]PF_6 (numbering refers to C atoms).

.

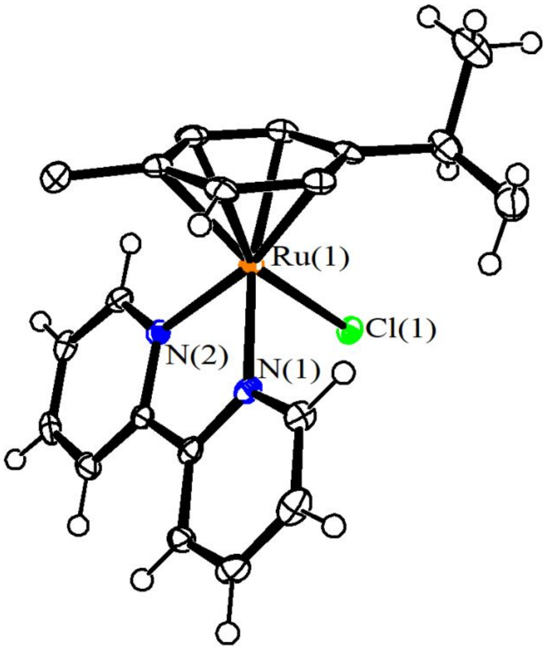

Figure S82. View of the organometallic cation in the X-Ray structure of [RuCl(2,2'-bipyridine)(η^6-*p*-cymene)]PF_6. Displacement ellipsoids are at the 50% probability level. Main bond distances (Å) and angles (°): Ru(1)-(η^6-p-cymene)$_{average}$ 2.200(7), Ru(1)-Cl(1) 2.4007(6), Ru(1)-N(1) 2.085(2), Ru(1)-N(2) 2.078(2), N(1)-Ru(1)-N(2) 77.13(9), Cl(1)-Ru(1)-N(1) 86.44(6), Cl(1)-Ru(1)-N(2) 84.36(7).

Table S3. Crystal data and measurement details for [RuCl(2,2'-bipyridine)(η^6-*p*-cymene)]PF_6.

Formula	$C_{20}H_{22}ClF_6N_2PRu$
FW	571.88
T, K	100(2)
λ, Å	0.71073
Crystal system	Orthorhombic
Space group	$P2_12_12_1$
a, Å	11.6499(5)
b, Å	12.2728(5)
c, Å	15.1603(6)
Cell Volume, $Å^3$	2167.57(15)
Z	4
D_c, g·cm^{-3}	1.752
μ, mm^{-1}	0.982
F(000)	1144
Crystal size, mm	0.16×0.14×0.11
θ limits,°	2.135–27.000
Reflections collected	33892
Independent reflections	4728 [R_{int} = 0.0291]
Data / restraints /parameters	4728 / 0 / 284
Goodness on fit on F^2	1.136
R_1 ($I > 2\sigma(I)$)	0.0182
wR_2 (all data)	0.0428
Largest diff. peak and hole, e Å^{-3}	0.351 / –0.463

Characterization of bipyridine chalcogenocyanato complexes

Figure S83. Solid-state IR spectra (650-4000 cm^{-1}) of [Ru(OCN)(bpy)(η^6-*p*-cymene)]PF_6, [**4**]PF_6 (κ*N*/κ*O* isomer ratio 1.2).

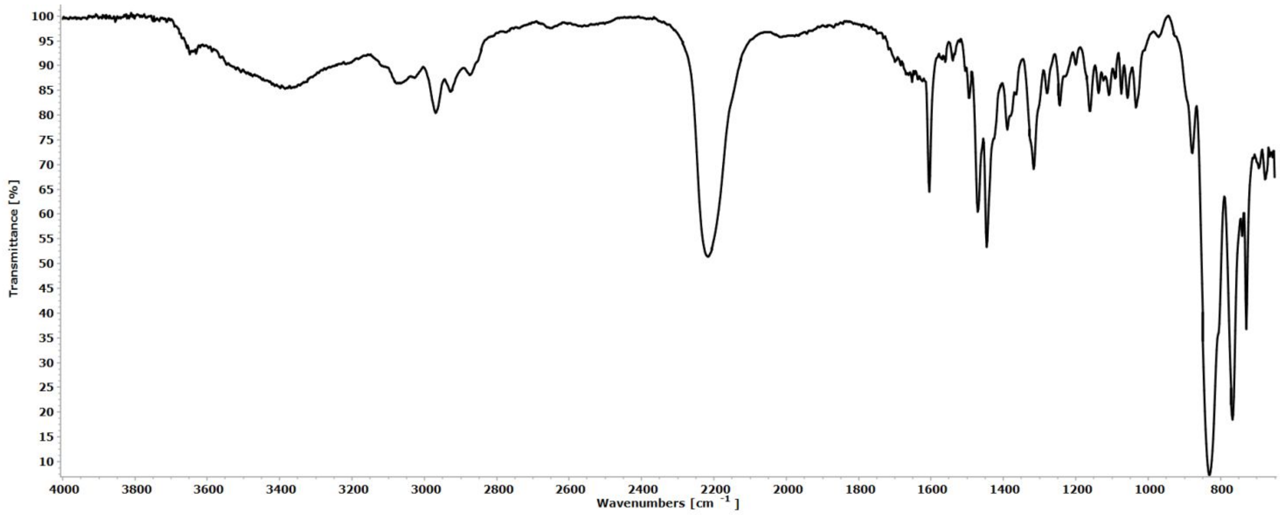

Figure S84. ^{1}H NMR spectrum (400 MHz, $CDCl_3$) of [**4**]PF_6 (κ*N*/κO isomer ratio 1.2). Resonances attributed to [$\mathbf{4}^{N}$]$^+$ are highlighted.

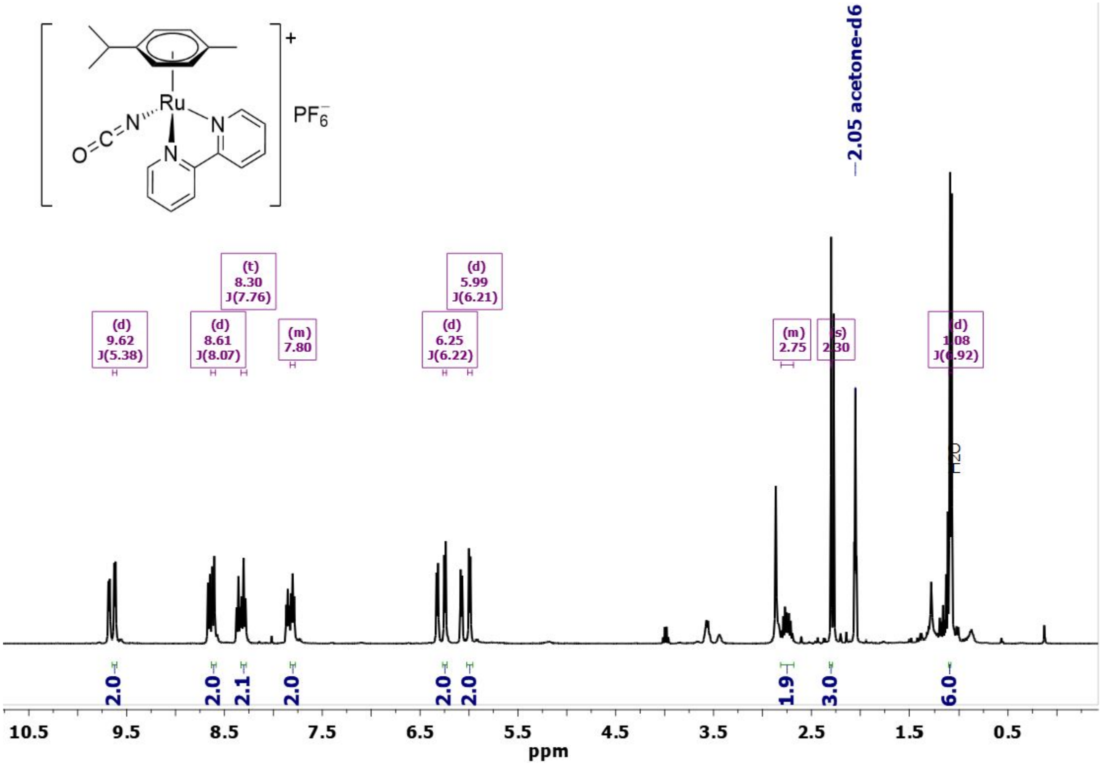

Figure S85. ^{1}H NMR spectrum (400 MHz, $CDCl_3$) of [**4**]PF_6 (κ*N*/κO isomer ratio 1.2). Resonances attributed to [$\mathbf{4}^{O}$]$^{+}$ are highlighted.

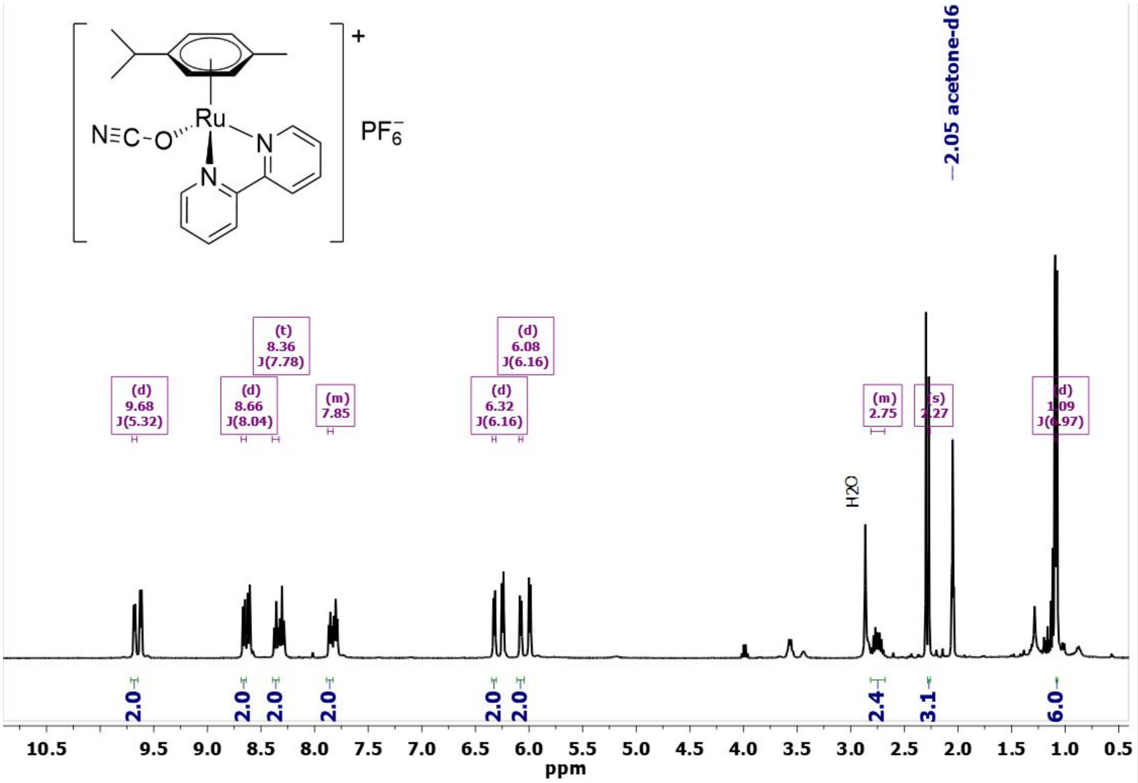

Figure S86. $^{13}C\{^{1}H\}$ NMR spectrum (100 MHz, $CDCl_3$) of [**4**]PF_6 (κ*N*/κO isomer ratio 1.2). Resonances attributed to [$\mathbf{4}^{N}$]$^{+}$ are highlighted.

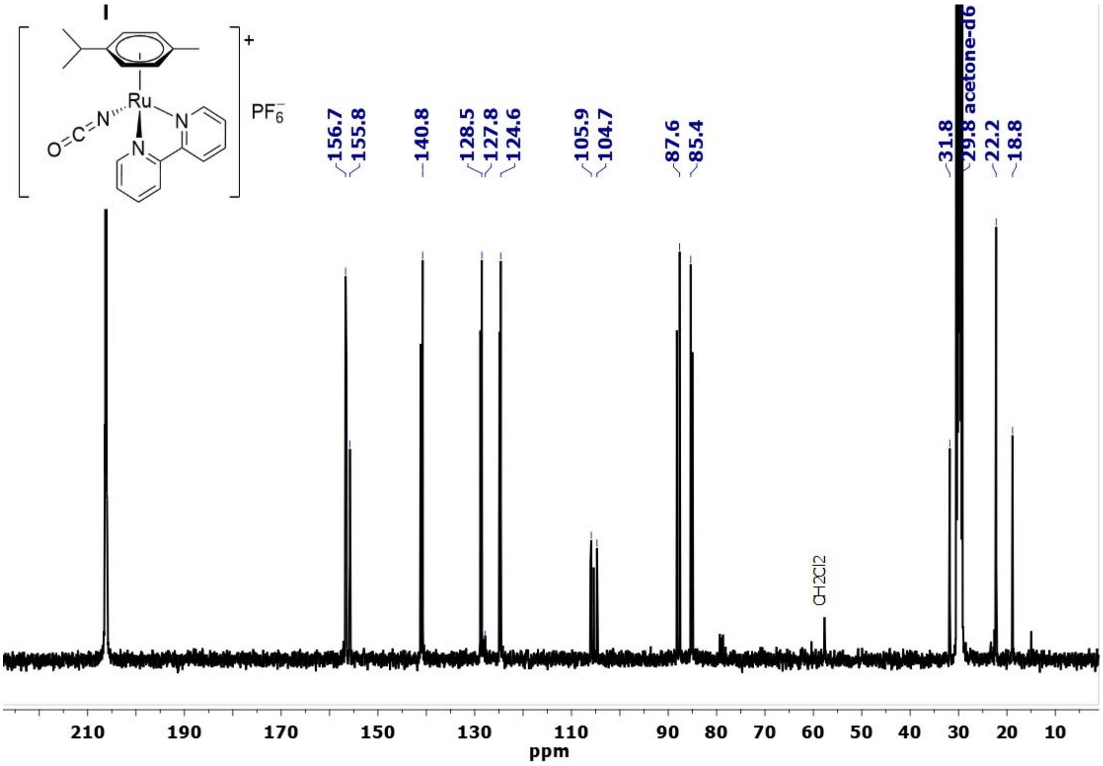

Figure S87. $^{13}C\{^1H\}$ NMR spectrum (100 MHz, $CDCl_3$) of [**4**]PF_6 (κ*N*/κO isomer ratio 1.2). Resonances attributed to [$\mathbf{4^O}$]$^+$ are highlighted.

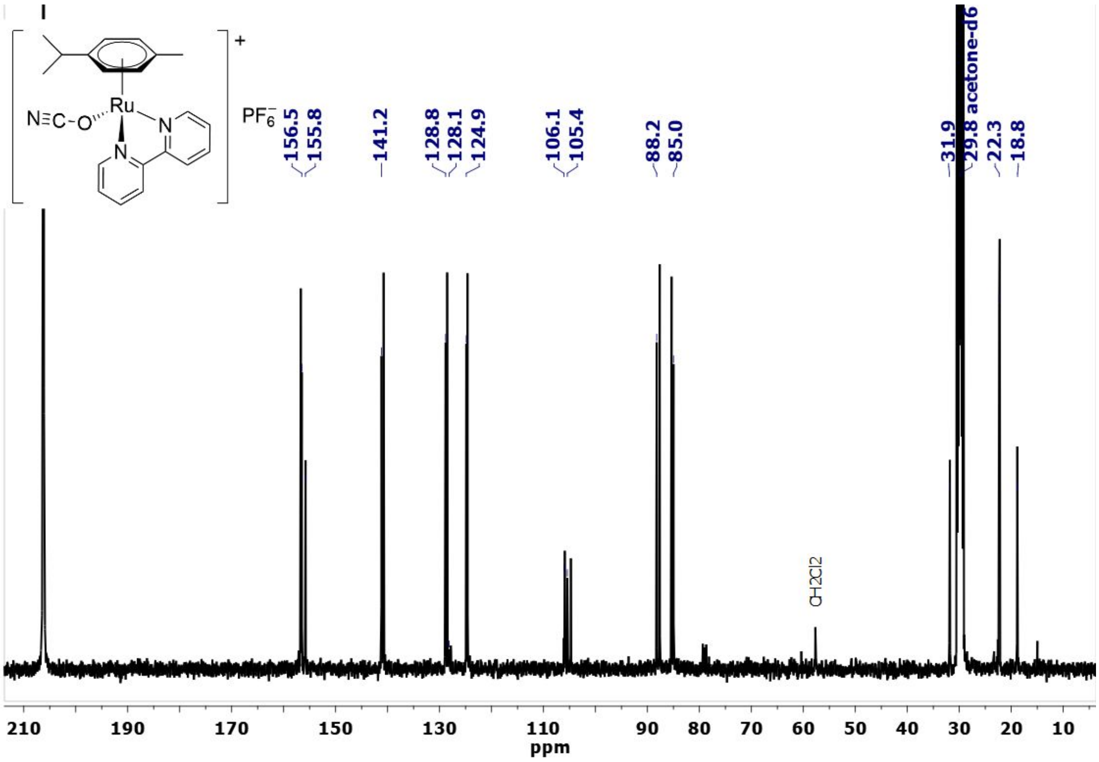

Figure S88. Solid-state IR spectra (650-4000 cm^{-1}) of samples of [Ru(SCN)(bpy)(η^6-*p*-cymene)]PF_6, [**5**]PF_6, with predominance of the κ*N* isomer (κ*N*/κ*S* ratio 15, blue line) or the κ*S* isomer (κ*N*/κ*S* ratio 0.23, orange line).

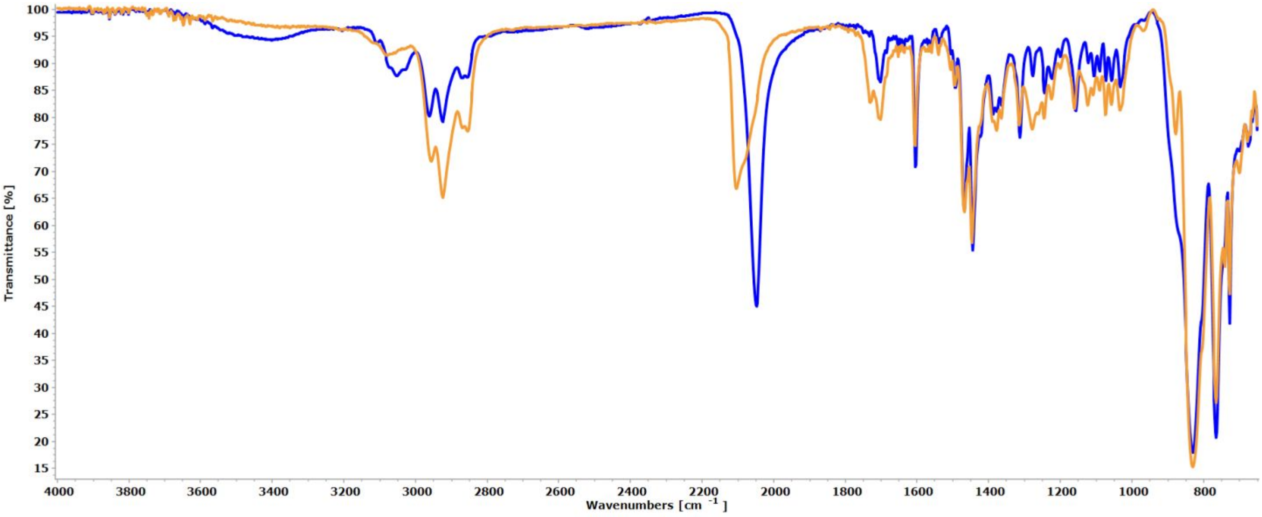

Figure S89. ^{1}H NMR spectrum (400 MHz, acetone-d$_6$) of [**5**]PF$_6$ (κ*N*/κ*S* isomer ratio > 15). Resonances attributed to [**5*N***]$^+$ are highlighted.

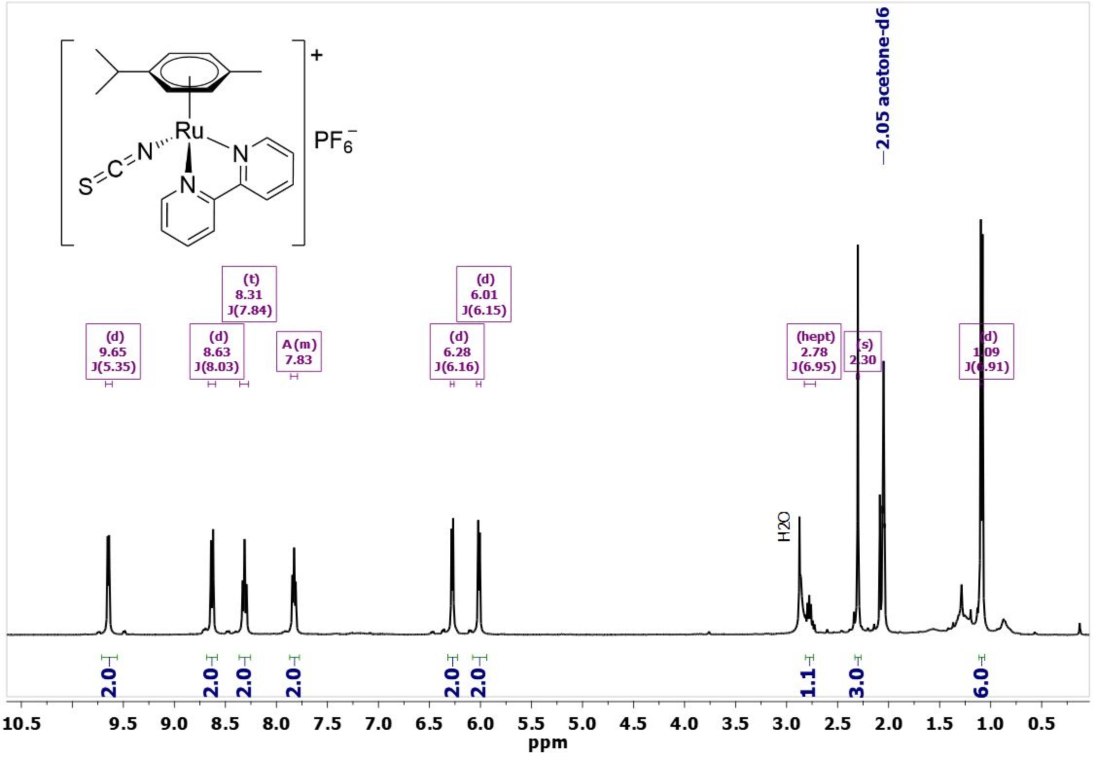

Figure S90. ^{1}H NMR spectrum (400 MHz, acetone-d$_6$) of [**5**]PF$_6$ (κ*N*/κ*S* isomer ratio 0.23 + a third species, [**5*X***]$^+$ in the main text). Resonances attributed to [**5*S***]$^+$ are highlighted.

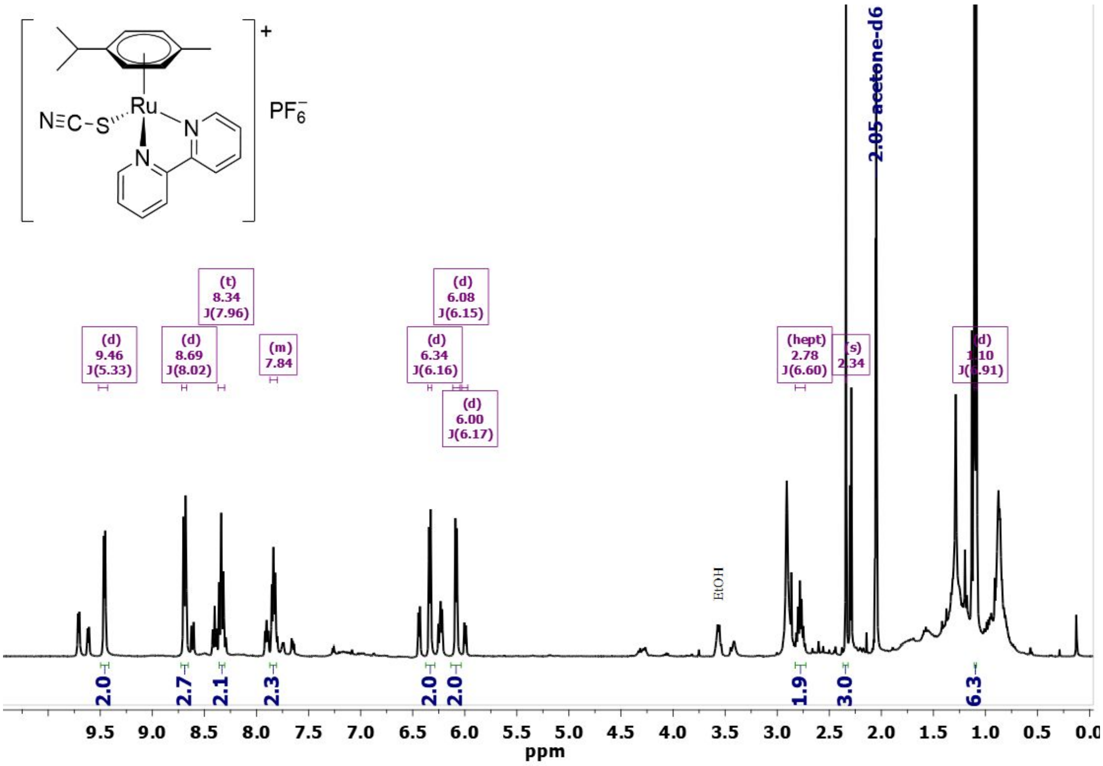

Figure S91. $^{13}C\{^1H\}$ NMR spectrum (125 MHz, acetone-d$_6$) of [**5**^{N}]PF_6 (pure κ*N* isomer).

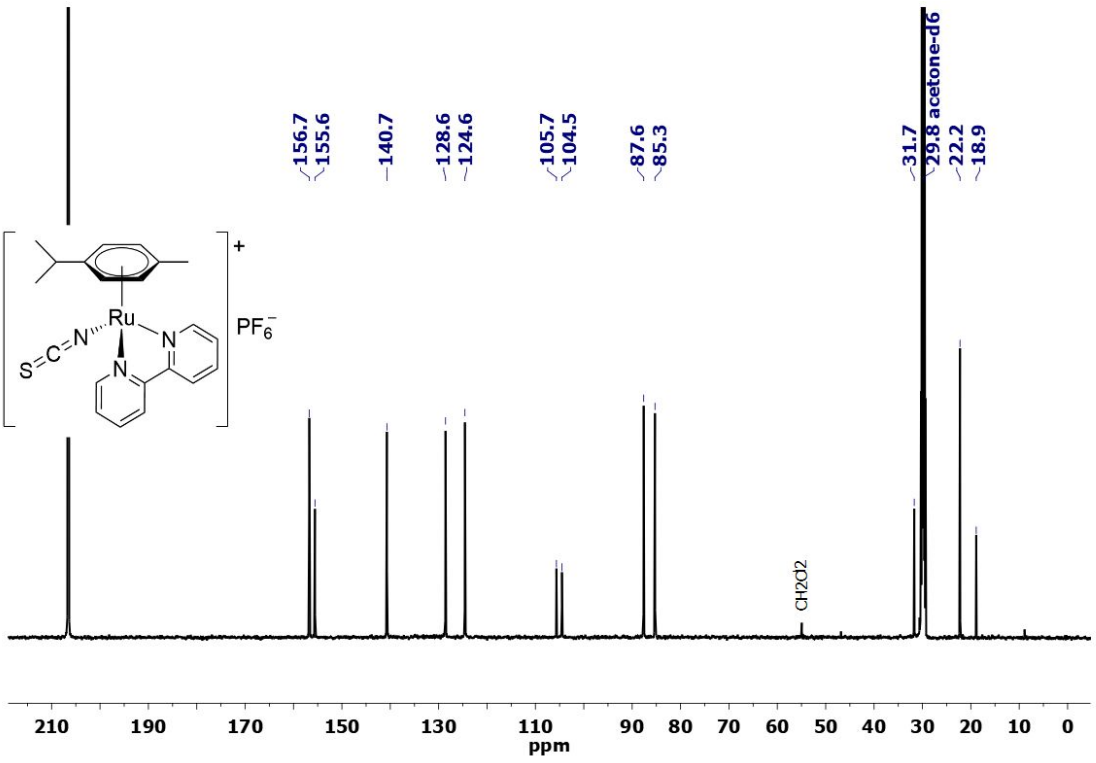

Figure S92. $^{13}C\{^1H\}$ NMR spectrum (100 MHz, acetone-d$_6$) of [**5**]PF_6 (κ*N*/κ*S* isomer ratio 0.64 + a third species, [**5**^{X}]$^+$ in the main text). Resonances attributed to [**5**^{S}]$^+$ are highlighted. The CS resonance of [**5**^{N}]$^+$ at 138 ppm is also indicated.

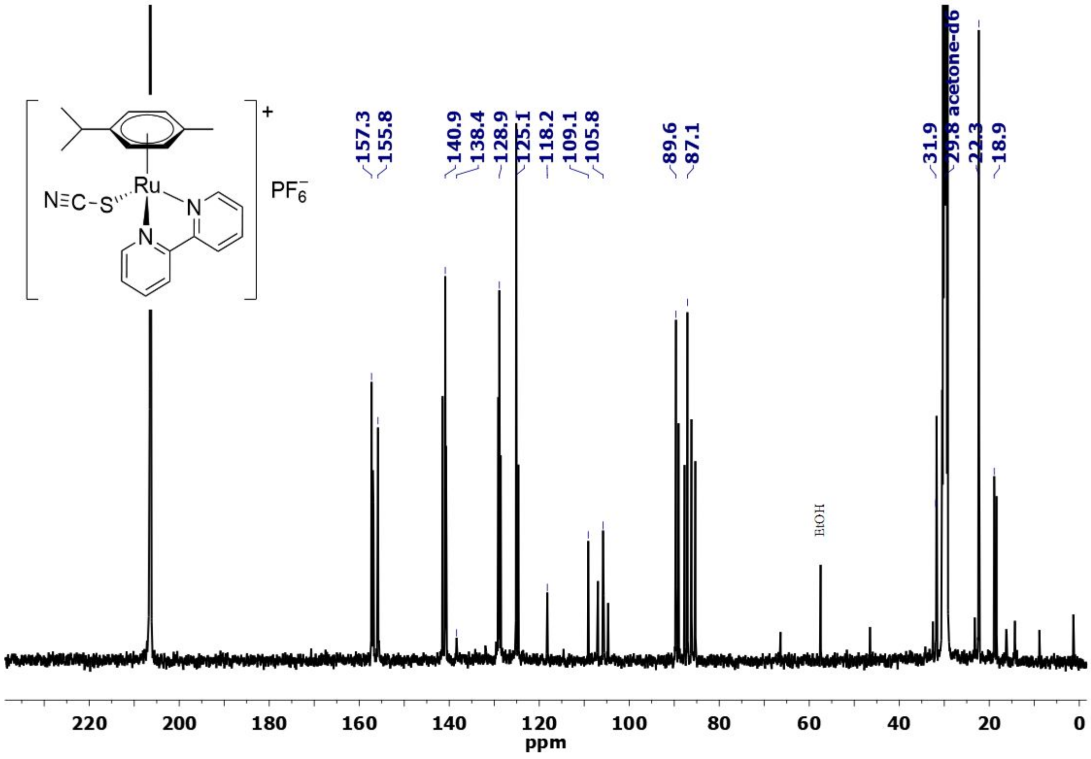

Figure S93. Solid-state IR spectra (650-4000 cm^{-1}) of samples of [Ru(SeCN)(bpy)(η^6-*p*-cymene)]PF_6, [**6**]PF_6, with predominance of the κ*N* isomer (κ*N*/κ*Se* ratio 7.0, blue line) or the κ*Se* isomer (κ*N*/κ*Se* ratio 0.85, orange line).

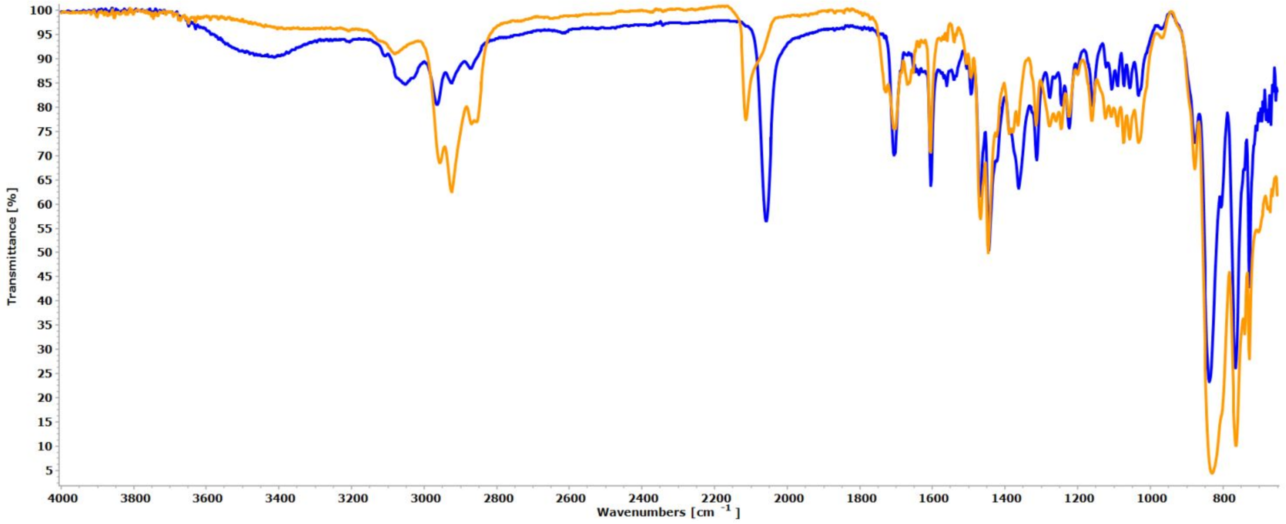

Figure S94. ^1H NMR spectrum (400 MHz, acetone-d_6) of [**6**]PF_6 (κ*N*/κ*Se* isomer ratio 3.0). Resonances attributed to [**6**^{N}]$^+$ are highlighted.

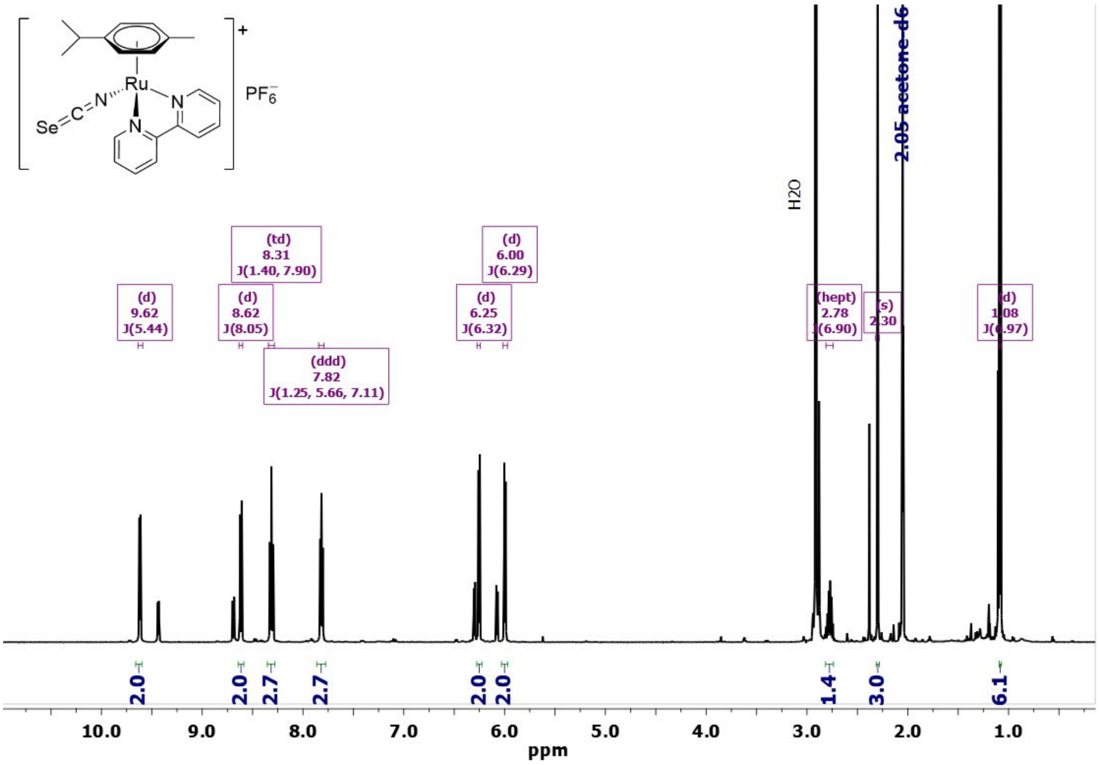

Figure S95. ^{1}H NMR spectrum (400 MHz, acetone-d_6) of [**6**]PF_6 (κ*N*/κ*Se* isomer ratio 0.85). Resonances attributed to [$\mathbf{6^{Se}}$]$^+$ are highlighted.

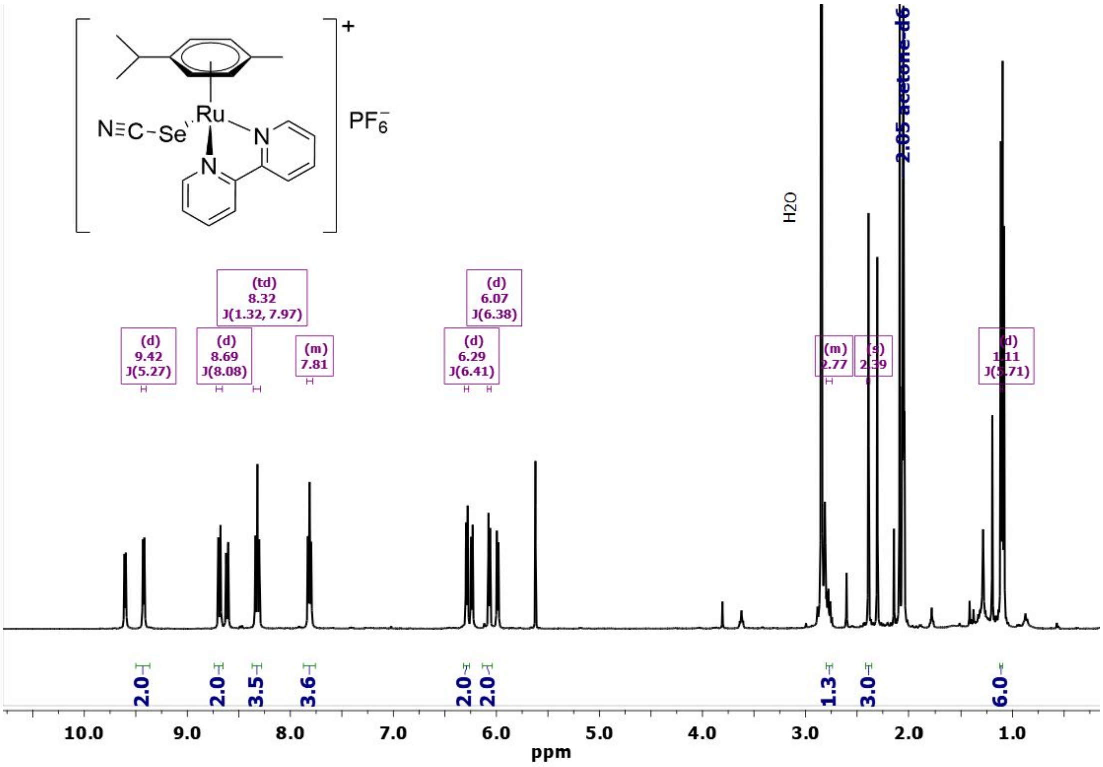

Figure S96. $^{13}C\{^{1}H\}$ NMR spectrum (100 MHz, acetone-d_6) of [**6**]PF_6 (κ*N*/κ*Se* isomer ratio 3.0). Resonances attributed to [$\mathbf{6^{N}}$]$^+$ are highlighted.

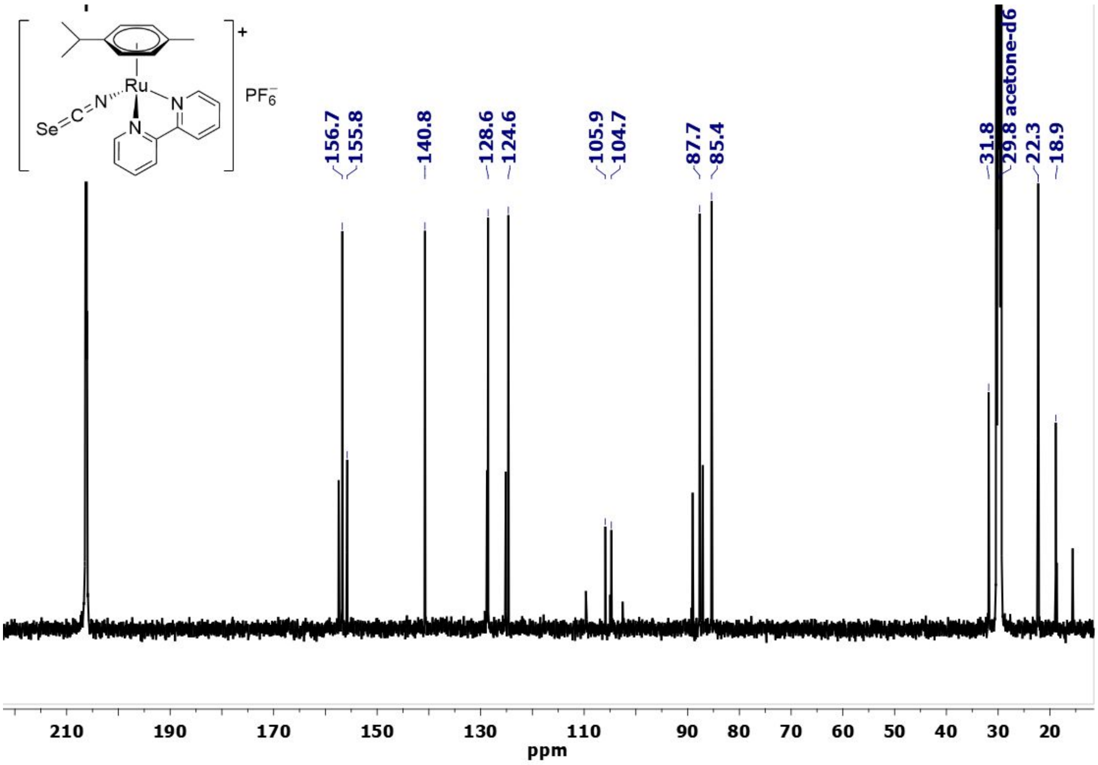

Figure S97. $^{13}C\{^1H\}$ NMR spectrum (100 MHz, acetone-d$_6$) of [**6**]PF$_6$ (κ*N*/κ*Se* isomer ratio 0.85). Resonances attributed to [$\mathbf{6}^{Se}$]$^+$ are highlighted.

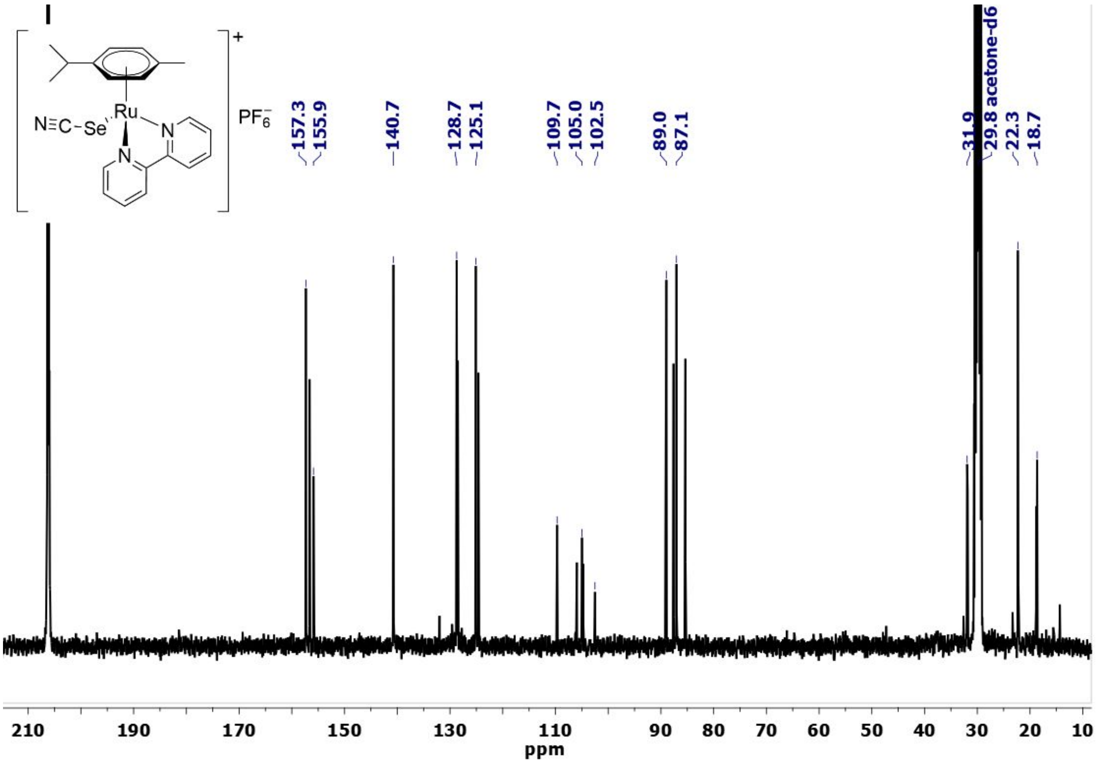

Figure S98. ^{77}Se NMR spectra (76 MHz, acetone-d$_6$) of [**6**]PF$_6$ (κ*N*/κ*Se* isomer ratio 3.0; blue line) and after thermal treatment in refluxing EtOH (κ*N*/κ*Se* isomer ratio 0.85; red line).

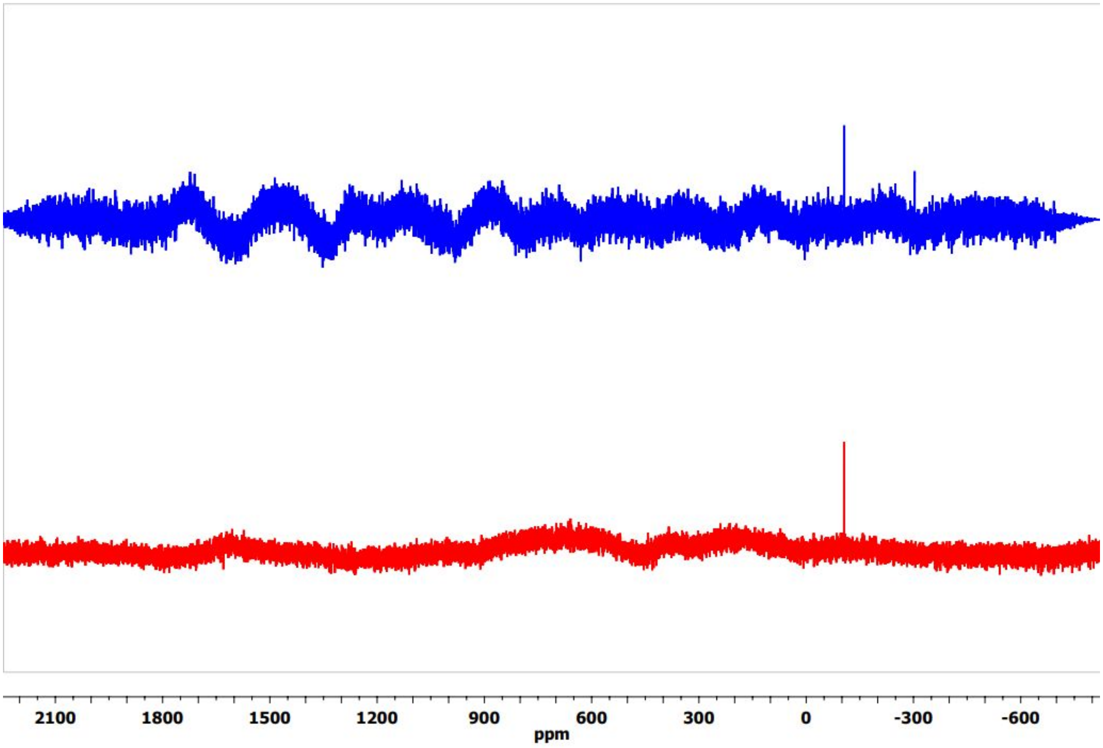

Table S4. Comparison of ^{13}C, ^{77}Se NMR (acetone-d_6 or acetone/C_6D_6 solutions) and solid-state IR data for the ECN moiety (E = O, S, Se) in their tetrabutylammonium salts and in their ruthenium arene 2,2'-bipyridyl complexes. Isomer ratios for [Ru(ECN)(bpy)(η^6-*p*-cymene)]$^+$ complexes in solution (^1H NMR, acetone-d_6).

Cpd	Chemical sketch	IR (C≡N) ũ / cm^{-1}	IR (C-O) ũ / cm^{-1}	^{13}C NMR (ECN) δ / ppm	^{77}Se NMR δ / ppm	κ*N*/κ*E* ratio at room T	κ*N*/κ*E* ratio after thermal treatment
**[Bu$_4$N][OCN]**	$^-$O−C≡N	2141	n.d.	129.0	-	-	-
**[4^N]PF$_6$**	Ru−N=C=O	2217br.	1316	127.8	-	1.2	1.2
**[4°]PF$_6$**	Ru−O−C≡N		1137	128.1	-		
**[Bu$_4$N][SCN]**	$^-$S−C≡N	2054	-	131.7	-	-	-
**[5^N]PF$_6$**	Ru−N=C=S	2049	-	138.4	-	≥ 15 [a][b]	0.64 (6 h)
**[5^S]PF$_6$**	Ru−S-C≡N	2103	-	118.0	-		0.64 (14 h)
**[Bu$_4$N][SeCN]**	$^-$Se−C≡N	2066	-	118.0	− 300	-	-
**[6^N]PF$_6$**	Ru−N=C=Se	2058	-	131.0	− 303	7.0 [c]	0.85 (14 h)
**[6^{Se}]PF$_6$**	Ru−Se−C≡N	2113	-	102.6	− 106		

[a] No isomerization was observed at room T during the next 14 h. [b] In one case, no trace of the κ*S* isomer was detected by ^1H NMR. [c] Measured in the freshly-prepared solution, changed to 3.0 after 14 h at room T.

References

1 (a) Galliart, A.; Brown, T. M. Ammonium and Tetraalkylammonium Cyanates, *Synth. React. Inorg. Met.-Org. Chem.*, **1972**, *2:4*, 273-275. (b) Kobler, A. H.; Munz, R.; Gasser, G. A.; Simchen, G. Eine einfache Synthese von Tetraalkylammoniumsalzen mit funktionellen Liebigs *Ann. Chem.* **1978**, 1937-1945.

2 Dehmlow, E. V.; Vehre, B.; Broda, W. A novel method for the preparation of tetra-n-butyl- to tetra-n-octylammonium hydrogen sulfates, *Synthesis* **1985**, *5*, 508-509

3 Burmeister, J. L.; Williams, L. E. Coordination Complexes of the Selenocyanate Ion, *Inorg. Chem.* **1966**, *5*, 1113-1117.

4 (a) Kaim, W.; Reinhardt, R.; Sieger, M. Chemical and Electrochemical Generation of Hydride-Forming Catalytic Intermediates (bpy)M(CnRn): M = Rh, Ir (n = 5); M = Ru, Os (n = 6). Coordinatively Unsaturated Ground State Models of MLCT Excited States? *Inorg. Chem.* **1994**, *33*, 4453-4459. (b) Colina-Vegas, L.; Villarreal, W.; Navarro, M.; Rodrigues de Oliveira, C.; Graminha, A. E.; Ivo da S. Maia, P.; Deflon, V. M.; Ferreira, A. G.; Cominetti, M. R.; Batista, A. A. Cytotoxicity of Ru(II) piano–stool complexes with chloroquine and chelating ligands against breast and lung tumor cells: Interactions with DNA and BSA, *J. Inorg. Biochem.* **2015**, *153*, 150–161.

5 Wu, X.; Liu, J.; Di Tommaso, D.; Iggo, J. A.; Catlow, C. R. A.; Bacsa, J.; Xiao, J.; A Multilateral Mechanistic Study into Asymmetric Transfer Hydrogenation in Water, *Chem. Eur. J.* **2008**, *14*, 7699–7715.
